# Beta and Gamma Amino Acid-Substituted Benzenesulfonamides as Inhibitors of Human Carbonic Anhydrases

**DOI:** 10.3390/ph15040477

**Published:** 2022-04-13

**Authors:** Benas Balandis, Tomas Šimkūnas, Vaida Paketurytė-Latvė, Vilma Michailovienė, Aurelija Mickevičiūtė, Elena Manakova, Saulius Gražulis, Sergey Belyakov, Visvaldas Kairys, Vytautas Mickevičius, Asta Zubrienė, Daumantas Matulis

**Affiliations:** 1 Department of Organic Chemistry, Kaunas University of Technology, Radvilėnų pl. 19, LT-50254 Kaunas, Lithuania; benas.balandis@ktu.edu (B.B.); vytautas.mickevicius@ktu.lt (V.M.); 2 Department of Biothermodynamics and Drug Design, Institute of Biotechnology, Life Sciences Center, Vilnius University, Saulėtekio 7, LT-10257 Vilnius, Lithuania; tomas.simkunas@gmc.stud.vu.lt (T.Š.); vaida.paketuryte@gmc.vu.lt (V.P.-L.); vilma.michailoviene@bti.vu.lt (V.M.); aurelija.mickeviciute@bti.vu.lt (A.M.); asta.zubriene@bti.vu.lt (A.Z.); 3 Department of Protein–DNA Interactions, Institute of Biotechnology, Life Sciences Center, Vilnius University, Saulėtekio al. 7, LT-10257 Vilnius, Lithuania; elena.manakova@bti.vu.lt (E.M.); saulius.grazulis@bti.vu.lt (S.G.); 4 Laboratory of Physical Organic Chemistry, Latvian Institute of Organic Synthesis, Aizkraukles 21, LV-1006 Riga, Latvia; serg@osi.lv; 5 Department of Bioinformatics, Institute of Biotechnology, Life Sciences Center, Vilnius University, Saulėtekio al. 7, LT-10257 Vilnius, Lithuania; visvaldas.kairys@bti.vu.lt

**Keywords:** carbonic anhydrase inhibitor, benzenesulfonamide, fluorescent thermal shift assay, X-ray crystallography, intrinsic thermodynamics of binding

## Abstract

A series of novel benzenesulfonamide derivatives were synthesized bearing *para*-*N* β,γ-amino acid or *para*-*N* β-amino acid and thiazole moieties and their binding to the human carbonic anhydrase (CA) isozymes determined. These enzymes are involved in various illnesses, such as glaucoma, altitude sickness, epilepsy, obesity, and even cancer. There are numerous compounds that are inhibitors of CA and used as pharmaceuticals. However, most of them bind to most CA isozymes with little selectivity. The design of high affinity and selectivity towards one CA isozyme remains a significant challenge. The beta and gamma amino acid-substituted compound affinities were determined by the fluorescent thermal shift assay and isothermal titration calorimetry for all 12 catalytically active human carbonic anhydrase isozymes, showing the full affinity and selectivity profile. The structures of several compounds were determined by X-ray crystallography, and the binding mode in the active site of CA enzyme was shown.

## 1. Introduction

Carbonic anhydrases (CAs, EC 4.2.1.1) are zinc-containing metalloenzymes that catalyze the reversible hydration of CO_2_ to bicarbonate and maintain the acid–base balance in tissues and blood and, thus, play a crucial physiological role [1,2,3,4,5]. Human CAs belong to the α-family, which consists of 12 catalytically active (CAI, CAII, CAIII, CAIV, CAVA, CAVB, CAVI, CAVII, CAIX, CAXII, CAXIII, CAXIV) and three non-catalytic isozymes (CAVIII, CAX, and CAXI) that are also called carbonic anhydrase-related proteins (CARPs) [6,7]. Due to their different distribution in the body, the functions performed vary widely [8]. Therefore, disruption of normal function results in illnesses associated with only one or several CAs [9]. Thus, CAs are drug targets for a variety of disorders. There is still a significant challenge to design compounds possessing both high affinity and high selectivity for a specific isozyme, due to the structural similarity of the active sites of CAs [10,11,12]. Sulfonamides were discovered in 1940 as CA inhibitors by Mann and Keilin [13], who also reported some side effects of the newly discovered 4-aminobenzenesulfonamide. This was followed by a series of discoveries of a wide variety of compounds [14,15,16,17,18,19,20,21,22,23,24,25], some of which were approved as drugs. However, the desired properties and selectivity for all targeted CAs have not been achieved, and research in this field is ongoing.

The 5-oxopyrrolidine moiety is a part of a variety of compounds of natural or synthetic origin [26,27]. Recently, we have synthesized and examined the binding properties of 3-substituted 1-(4-sulfamoylphenyl)-5-oxopyrrolidine derivatives [28,29,30]. The different halo-substituents at the 4-sulfamoylphenyl moiety improved the binding properties of the compounds and allowed us to obtain several molecules that selectively bind to CAIX. In addition, we also investigated the binding properties of *N*-sulfamoylphenyl- and *N*-sulfamoylphenyl-*N*-thiazolyl-β-alanines and their derivatives [31], which showed better binding results as compared to compounds with a 5-oxopyrrolidine moiety as a linker. With the aim of further investigating the *N*-thiazolyl-β-alanine residue, we synthesized some analogous thiazole derivatives with the aliphatic substituents at C-4 and C-5 of the thiazole ring and studied their binding to CA. Moreover, to our knowledge, there were no CA inhibition studies of benzenesulfonamides with a β,γ-amino acid moiety as a linker. Therefore, we decided to explore this idea and incorporated the γ-aminobutyric acid moiety into the *N*-sulfamoylphenyl-β-alanine structure.

## 2. Results and Discussion

### 2.1. Organic Synthesis

**Figure 1:**
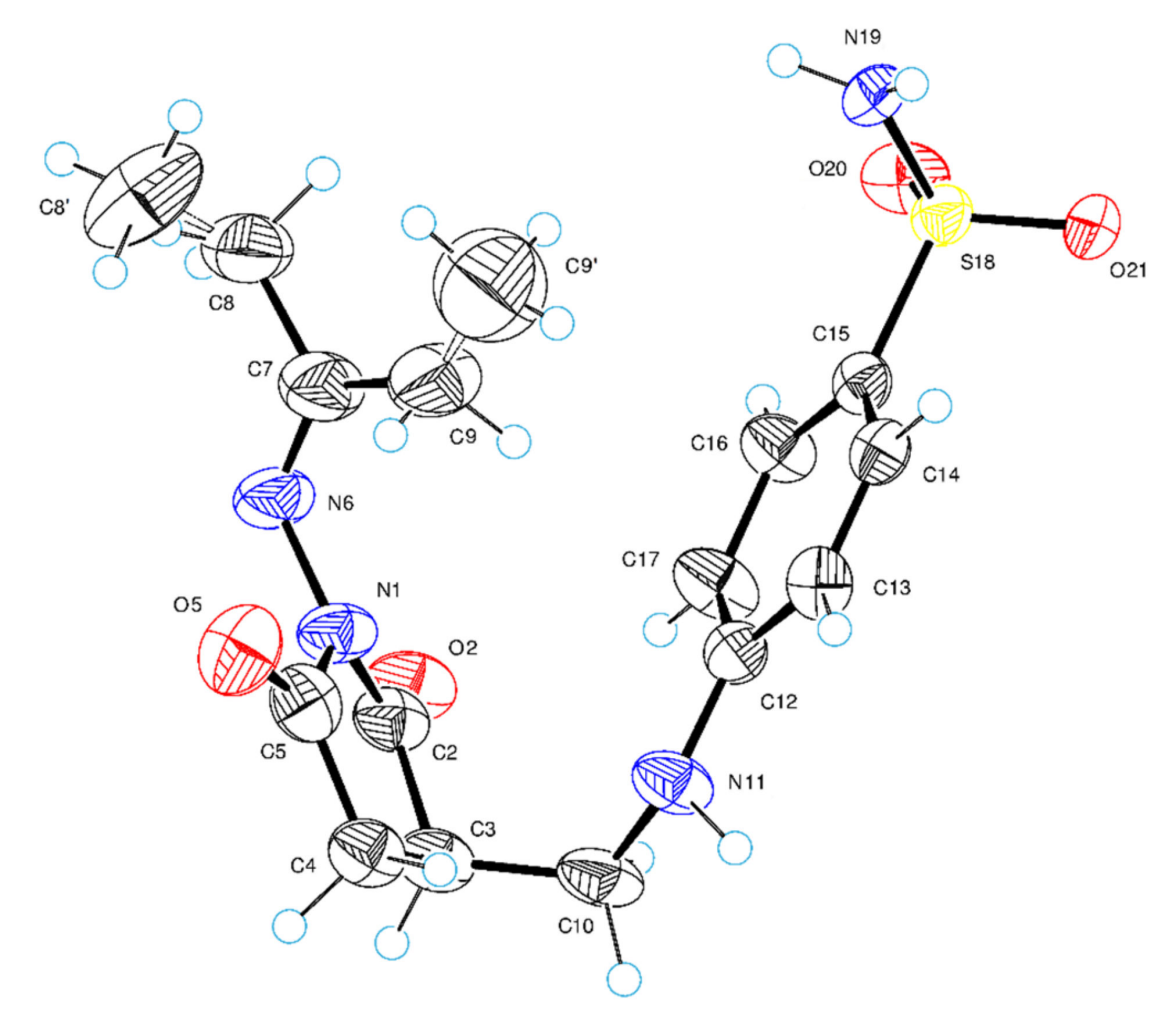
ORTEP (Oak Ridge Thermal-Ellipsoid Plot Program) molecular structure of compound **4a** showing the atom-numbering scheme. The thermal displacement ellipsoids are drawn at the 50% probability level and hydrogen atoms are shown as small spheres of arbitrary radii.

**Scheme 1:**
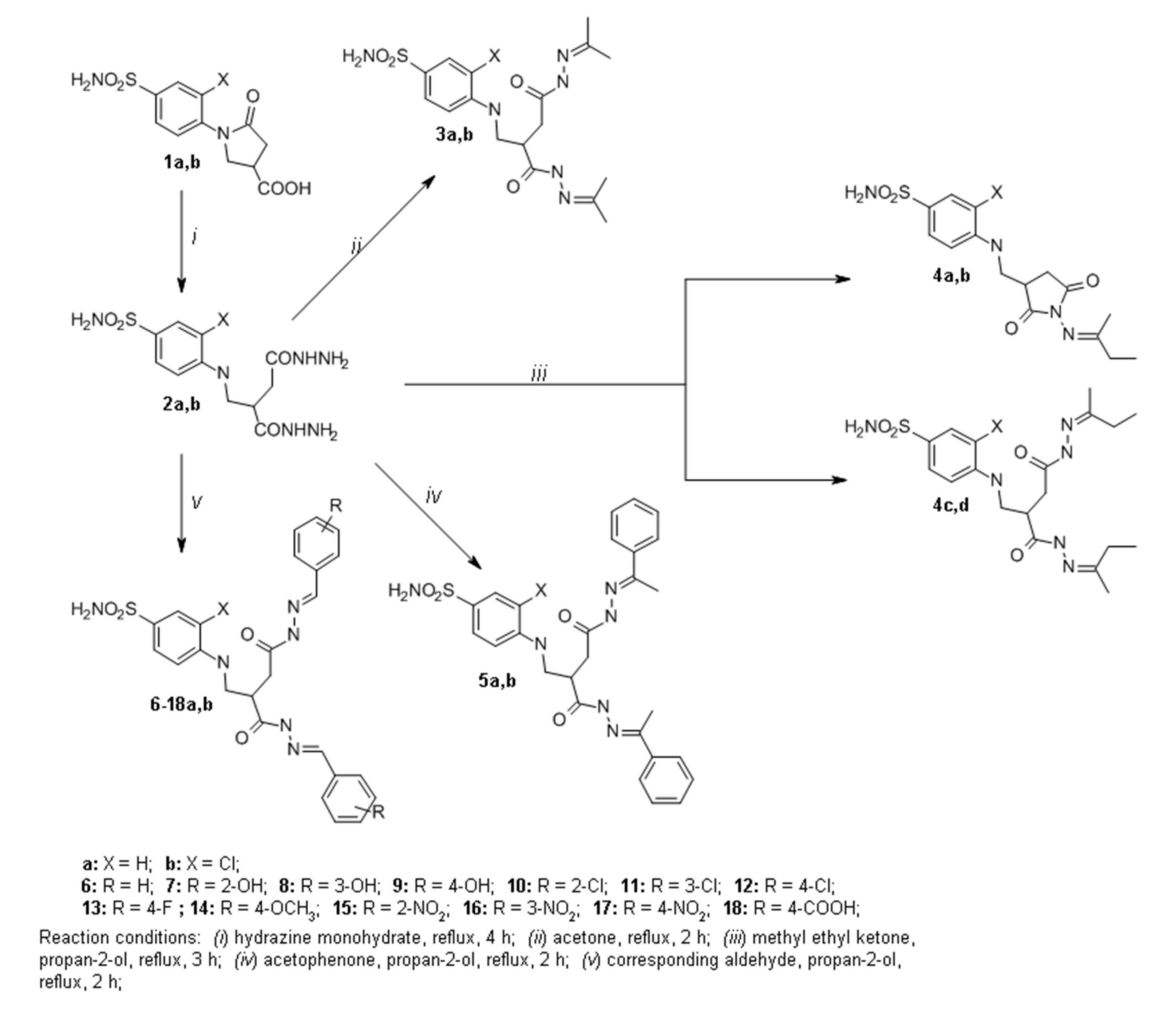
Synthesis of compounds **2**–**18a,b**.

Pyrrolidinones **1a**,**b**, which were used in this work as precursors, were synthesized according to the methods described in previous works [29,30]. Compounds 4-((2-(hydrazinecarbonyl)-4-hydrazineyl-4-oxobutyl)amino)benzenesulfonamide (**2a**) and 3-chloro-4-((2-(hydrazinecarbonyl)-4-hydrazineyl-4-oxobutyl)amino)benzenesulfonamide (**2b**) were synthesized by nucleophilic ring-opening reactions of pyrrolidones **1a**,**b** by heating them under reflux in an excess of hydrazine monohydrate [32] (Scheme 1). The structures of **2a**,**b** and all of the other compounds have been confirmed by FT-IR, ^1^H and ^13^C NMR spectroscopy, and mass spectrometry or elemental analysis data. The triplet signals of NH group at 6.39 ppm (**2a**) and 6.05 ppm (**2b**) in the ^1^H NMR spectra of compounds **2a**,**b** confirmed the existence of an open pyrrolidone ring structure. Subsequently, β,γ-amino acid derivatives **2a**,**b** were used as the precursors for the synthesis of a series of hydrazone derivatives. Reactions between compounds **2a**,**b** and ketones (acetone and acetophenone) at reflux temperature provided corresponding hydrazones **3a**,**b** and **5a**,**b**, whereas the ones with corresponding benzaldehydes yielded hydrazones **6a**,**b**–**18a**,**b** (Scheme 1). ^1^H NMR spectra for compounds **3a**,**b** and **5a**,**b** display double sets of resonances for each of the CO-NH group protons, due to the restricted rotation around the amide bond. Similar duplication of CO-NH and N=CH group protons signals was also observed in ^1^H NMR spectra of compounds **6**–**18a**,**b**. Since compounds **6**–**18a**,**b** possess two isomerism centers, existing in each side chain, 10 isomers of these compounds can form. Therefore, CO-NH or N=CH group proton resonances were observed as the sets of multiplets. For instance, in the ^1^H NMR spectrum for **6a**, multiplets at 7.94–8.23 ppm and 11.21–11.65 ppm were assigned to N=CH and CO-NH group protons, respectively. This splitting of the proton resonances indicates that in DMSO-*d_6_* solution hydrazones **3a**,**b**, **5a**,**b,** and **6**–**18a**,**b** exist as a mixture of *Z*/*E* isomers. However, in the attempt to synthesize analogous dihydrazones from compounds **2a**,**b** with methyl ethyl ketone, dioxopyrrolidine derivatives **4a**,**b** were obtained instead of target compounds **4c**,**d** (Scheme 1). The structure of compound **4a** was determined by X-ray structural analysis data (Figure 1), thereby confirming the empirical formula—C_15_H_20_N_4_O_4_S. In addition, in the ^1^H NMR spectrum of **4a**, the multiplet at 2.58–3.14 ppm was assigned to pyrrolidine CH_2_ group protons, while the multiplet at 3.25–3.59 ppm to CH and NCH_2_ group protons.

The multiplet at 6.45–6.58 ppm has been ascribed to protons of NH group. Furthermore, the multiplet at 0.82–1.24 ppm and a singlet at 1.70 ppm integrated for three protons of methyl groups, respectively. Similar spectral data were observed in compound **4b** ^1^H and ^13^C NMR spectra.

**Scheme 2:**
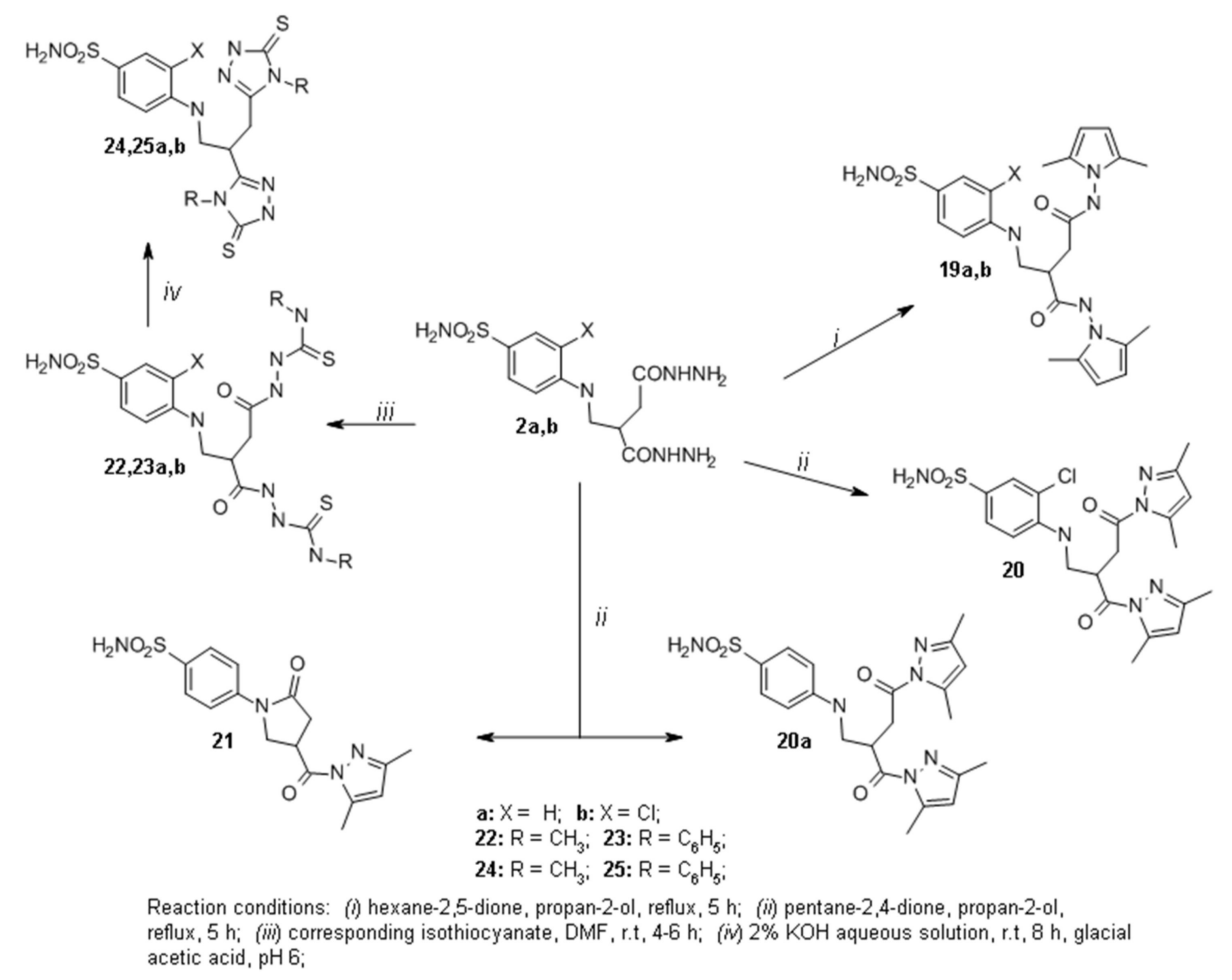
Synthesis of compounds **19**–**25**.

Hydrazides **2a**,**b** were used for the synthesis of a series of azole derivatives (Scheme 2). Pyrrole or pyrazole derivatives can be synthesized by the condensation reaction of carboxylic acid hydrazides with aliphatic diketones [33,34]. Pyrroles **19a**,**b** were obtained from hydrazides **2a**,**b** by the reactions with hexane-2,5-dione in propan-2-ol at the reflux temperature of the reaction mixture. For instance, in the ^1^H NMR spectrum for **19b**, singlets at 1.91, 1.94, and 1.98 ppm were assigned to the protons of CH_3_ groups, and each of the singlets at 5.61 and 5.63 ppm integrated for two protons of CH groups. These proton resonances have confirmed the presence of 2,5-dimethylpyrrole moiety. The singlets at 10.72 and 10.87 ppm have been ascribed to protons of NH groups. Similar signals are found in the compound **19a** ^1^H NMR spectrum as well. Furthermore, there was an attempt to synthesize 3,5-dimethylpyrrazoles **20** and **20a** using the same method. Unfortunately, only one of the desired products (compound **20**) was obtained. In the ^1^H NMR spectrum for **20**, four singlets integrated for 12 protons of CH_3_ groups at 2.18, 2.25, 2.35, and 2.43 ppm, and two singlets assigned to the protons in the CH groups at 6.16 and 6.24 ppm have proven the presence of 3,5-dimethylpyrazole moiety in the molecule. Moreover, the triplet at 6.52 ppm assigned to the proton of NH group proved an open 2-pyrrolidone ring structure of compound **20**. To obtain compound **20** non-chlorinated analog, previously synthesized pyrazole **21** [30] was isolated from the reaction mixture. The ^1^H and ^13^C NMR spectra data for **21** were very similar, as was described in previous work [30], which confirmed a closed 2-pyrrolidone ring structure in **21**. We have proposed a hypothesis that, due to the acidic properties of pentane-2,4-dione and high temperature, the 2-pyrrolidone ring closed during the reaction. These conditions were suitable for compound **20** synthesis. We proposed that some steric hindrance, which occurs due to the chlorine substitute at C-2 of the phenyl ring, prevents the closure of the 2-pyrrolidone ring. In order to synthesize the desired product **20a**, we have introduced several changes in reaction conditions. When the reaction was carried out at room temperature with NaOAc (to reduce the acidity of pentane-2,4-dione), we failed to detect new products by TLC after 24 h. Then we raised the reaction temperature to 50 °C, and after that we observed compound **21** by TLC.

Reactions of hydrazides **2a**,**b** with methyl isothiocyanate or its phenyl analog in DMF led to the formation of carbothioamides **22a**,**b** and carbothioamides **23a**,**b**, respectively (Scheme 2). Further condensation reactions of **22a**,**b** and **23a**,**b** in the alkaline medium resulted in the formation of methyl triazolethiones **24a**,**b** and phenyl triazolethiones **25a**,**b** correspondingly. In the ^1^H NMR spectra for **24a** and **24b**, singlets at 3.33, 3.39 ppm (**24a**) and 3.36, 3.40 ppm (**24b**) have been assigned to the protons of CH_3_ groups. In the ^13^C NMR spectrum for **24a**, two signals at 166.49 and 166.74 ppm have been attributed to C=S carbon in triazolethione ring. Similar carbon resonances were observed in compounds **24b** and **25a**,**b** ^13^C NMR spectra.

**Scheme 3:**
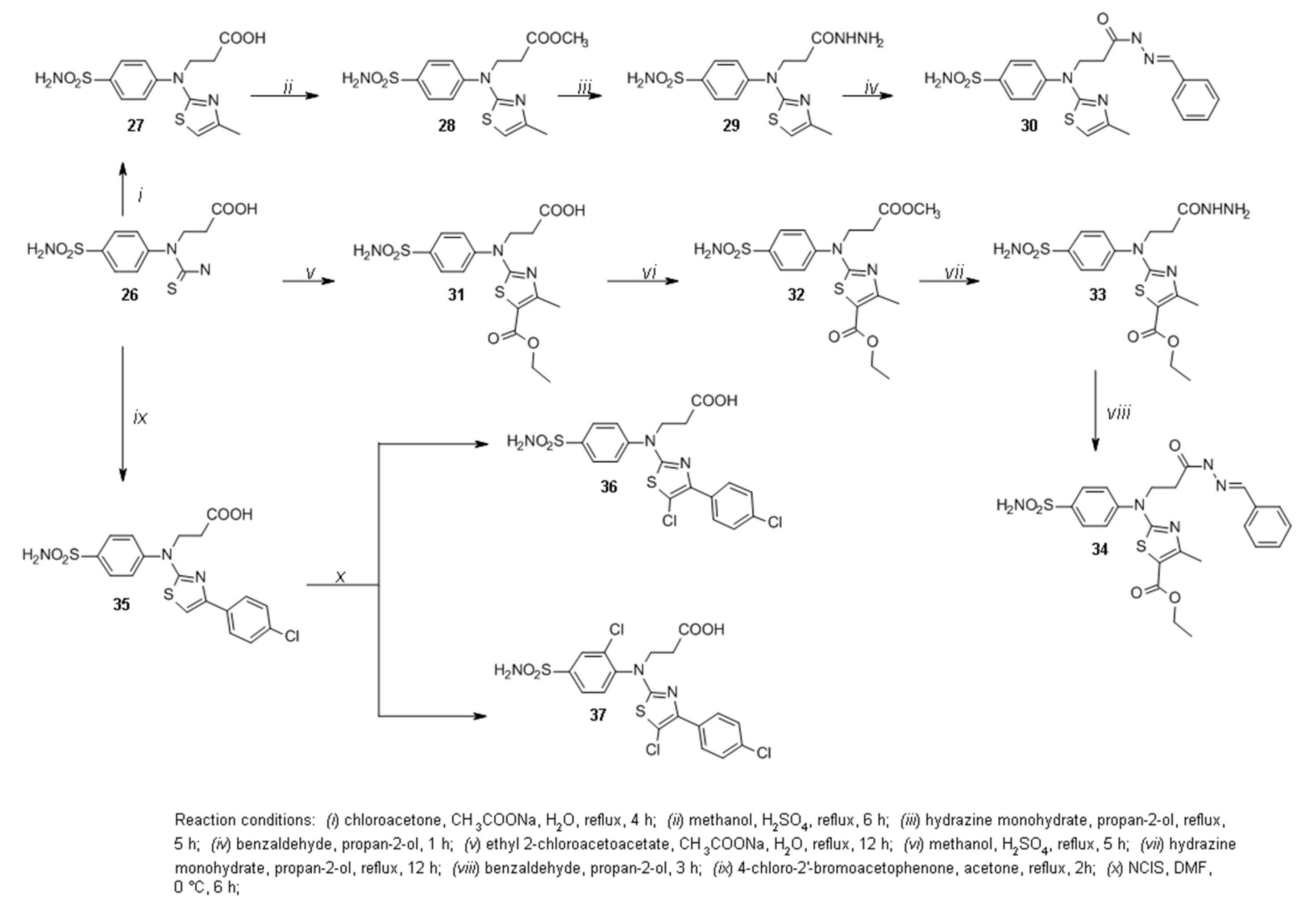
Synthesis of compounds **26**–**37**.

In the next stage of this work, thioureido acid **26** was synthesized by the procedure [31] and used for the synthesis of new functionalized aminothiazoles. Compound **26** was treated with an excess of chloroacetone in water, and sodium acetate was used as a base to obtain thiazole **27** (Scheme 3). In the ^1^H NMR spectra for **27**, singlets at 2.19 and 6.44 ppm have been assigned to the protons of CH_3_ and CH group, respectively. These proton resonances have confirmed the presence of 4-methylthiazol moiety. Later, ester **28** was synthesized by an esterification reaction of thiazole **27** with an excess of methanol at reflux in the presence of sulfuric acid as a catalyst. Hydrazine hydrate reaction with ester **28** in propan-2-ol at reflux temperature yielded hydrazide **29**, which was treated with benzaldehyde in propan-2-ol at reflux to afford hydrazone **30**. The ^1^H NMR spectra **30** display double sets of resonances for the CO-NH and N=CH group protons due to the restricted rotation around the amide bond. The splitting of the proton resonances is also observed for carbon chain protons—two triplets at 2.58 and 3.00 ppm were assigned to the protons of CH_2_ group, while the multiplet at 4.06–4.36 ppm region to protons of NCH_2_ group. This proton splitting indicates that in DMSO-*d_6_* solution hydrazone **30** exists as a mixture of *Z*/*E* isomers, and, in most cases, the *Z* isomer predominates [33].

The reaction of carboxylic acid **26** with ethyl 2-chloroacetoacetate in water at reflux gave thiazole **31**. Sodium acetate was used as a base (Scheme 3). In the ^1^H NMR spectra for **31**, a triplet at 2.19 and a singlet at 2.52 ppm have been assigned to the protons of CH_3_ groups. In the ^13^C NMR spectrum for **31**, two signals at 14.23 and 17.31 ppm have been attributed to CH_3_ group carbons and the signal at 60.22 to the carbon of ester CH_2_ group. These proton resonances have confirmed the formation of desired product **31**. Furthermore, thiazole **31** was treated with an excess of methanol in the presence of sulfuric acid as a catalyst to afford ester **32**, which was then used in the reaction with hydrazine hydrate to obtain hydrazide **33**. The reaction of sulfonamide **33** with benzaldehyde in propan-2-ol at reflux yielded hydrazone **34**. Double sets of resonances are also observed in the ^1^H NMR spectra for **34** similar to those in the spectra for **30**. Therefore, this indicates that hydrazone **34** also exists as a mixture of *Z*/*E* isomers in DMSO-*d_6_* solution.

Previously synthesized compound **35** [31] was obtained by stirring thioureido acid **26** with 4-chloro-2′-bromoacetophenone in acetone at reflux temperature. Chlorine was introduced into the phenyl ring at the C-3 position of the benzenesulfonamide moiety to determine the halogenation effect on the binding affinity toward carbonic anhydrases. Thiazole **35** was treated with *N*-chlorosuccinimide in DMF at 0 °C. In the ^1^H NMR spectra for **36**, the doublets at 7.54 and 7.72 ppm were attributed to the protons of the aromatic ring of 4-sulfamoylphenyl moiety. Moreover, there was no signal observed at the 7.38 ppm region as was described in previous work [31], which was assigned to the proton of CH group in the thiazole ring. This data confirmed that electrophilic substitution only occurred in the thiazole ring, which led to the formation of 5-chlorothiazole **36** but not the desired product **37**. To synthesize compound **37**, some changes were made to the reaction conditions. Performing the reaction at room temperature in DMF with NClS and monitoring the course of the reaction by TLC, after 1 h the formation of compound **36** was observed. It was then decided to raise the temperature to 80 °C [35], and the reaction was reperformed, but after the 20 h the only compound observed by TLC was thiazole **36**. Furthermore, chlorination of compound **35** was attempted in 6 M aqueous HCl solution with hydrogen peroxide overnight [29], although the only product isolated from the reaction mixture was 5-chlorothiazole **36**.

### 2.2. Crystal Structure of Compound ***4a***

The structure of **4a** was examined in more detail. Figure 1 shows a perspective view of molecule **4a** with thermal ellipsoids and the atom-numbering scheme followed in the text. The newly obtained 2,5-dioxopyrrolidine ring is planar and forms with the CH_2_ (C10) group a dihedral angle of 104.6(7). In the molecular structure, the 2,5-dioxopyrrolidine is almost perpendicular to both the hydrazone fragment (dihedral angle is 115.0(8)) and the benzenesulfonamide system (dihedral angle is equal to 113.3(8)).

### 2.3. Compound Binding to Carbonic Anhydrases (CA)

**Figure 2:**
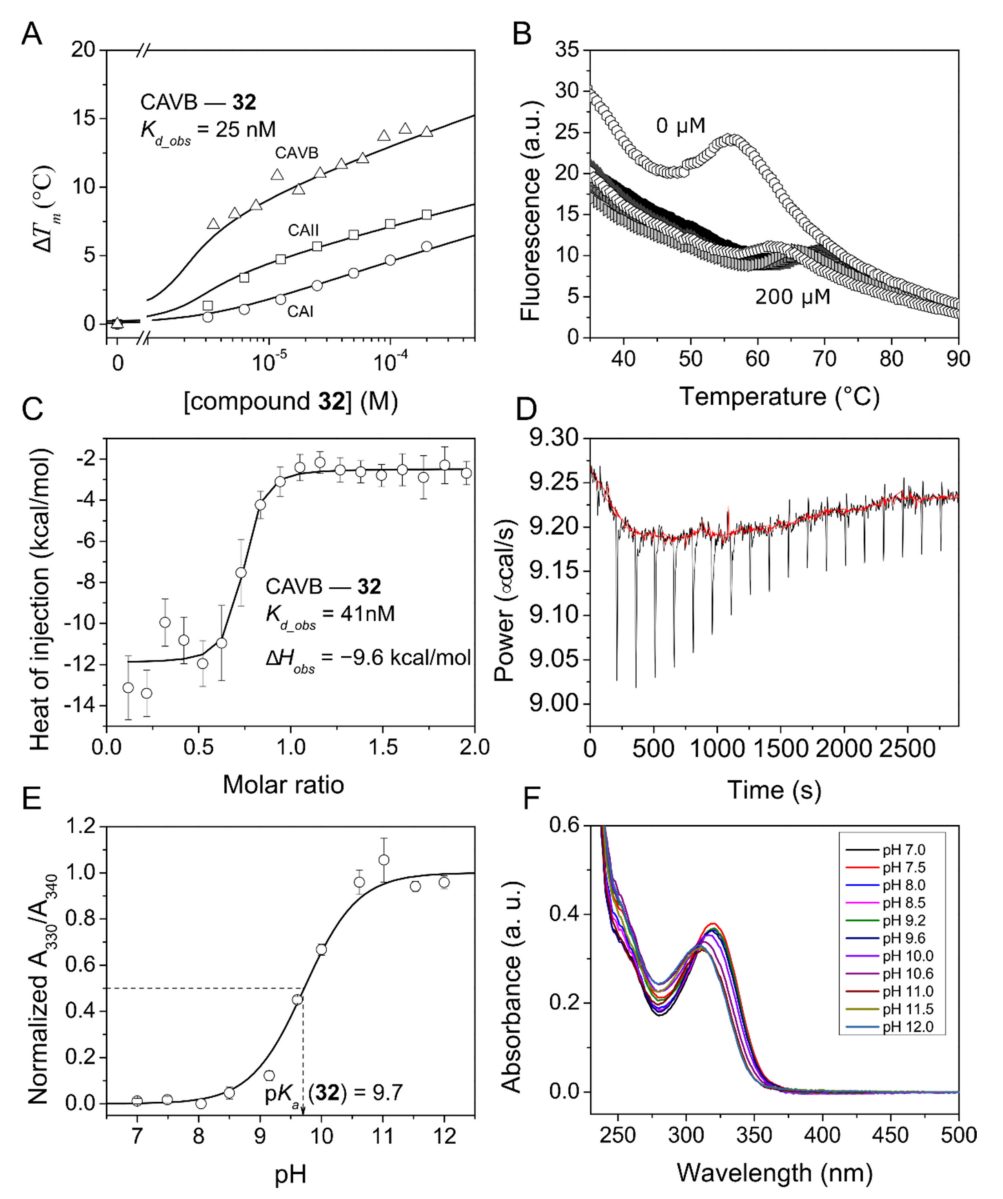
Fluorescent thermal shift assay (FTSA) data of compound **32** binding to CAI, CAII, and CAVB (panels (**A**,**B**), raw data only of CAVB) and isothermal titration calorimetry (ITC) data of compound **32** binding to CAVB (panels (**C**,**D**)). Dissociation constant, *K_d_obs_*, values at 37 °C and pH 7.0 in 50 mM sodium phosphate buffer containing 100 mM NaCl are shown near the corresponding graphs in panels (**A**,**C**). To calculate the intrinsic dissociation constants, the p*K_a_* values of compound sulfonamide group were measured spectrophotometrically at 37 °C (panels (**E**,**F**)). The normalized absorbances at selected wavelengths were plotted as a function of pH and fitted to the Henderson–Hasselbalch equation.

The dissociation constants of all synthesized compounds were measured for five CA isozymes, CAI, CAII, CAIX, CAXII, and CAXIII (Table 1), by the fluorescent thermal shift assay (FTSA, also referred to as TSA and DSF—differential scanning fluorimetry [36,37,38,39]) (Figure 2).

**Figure 3:**
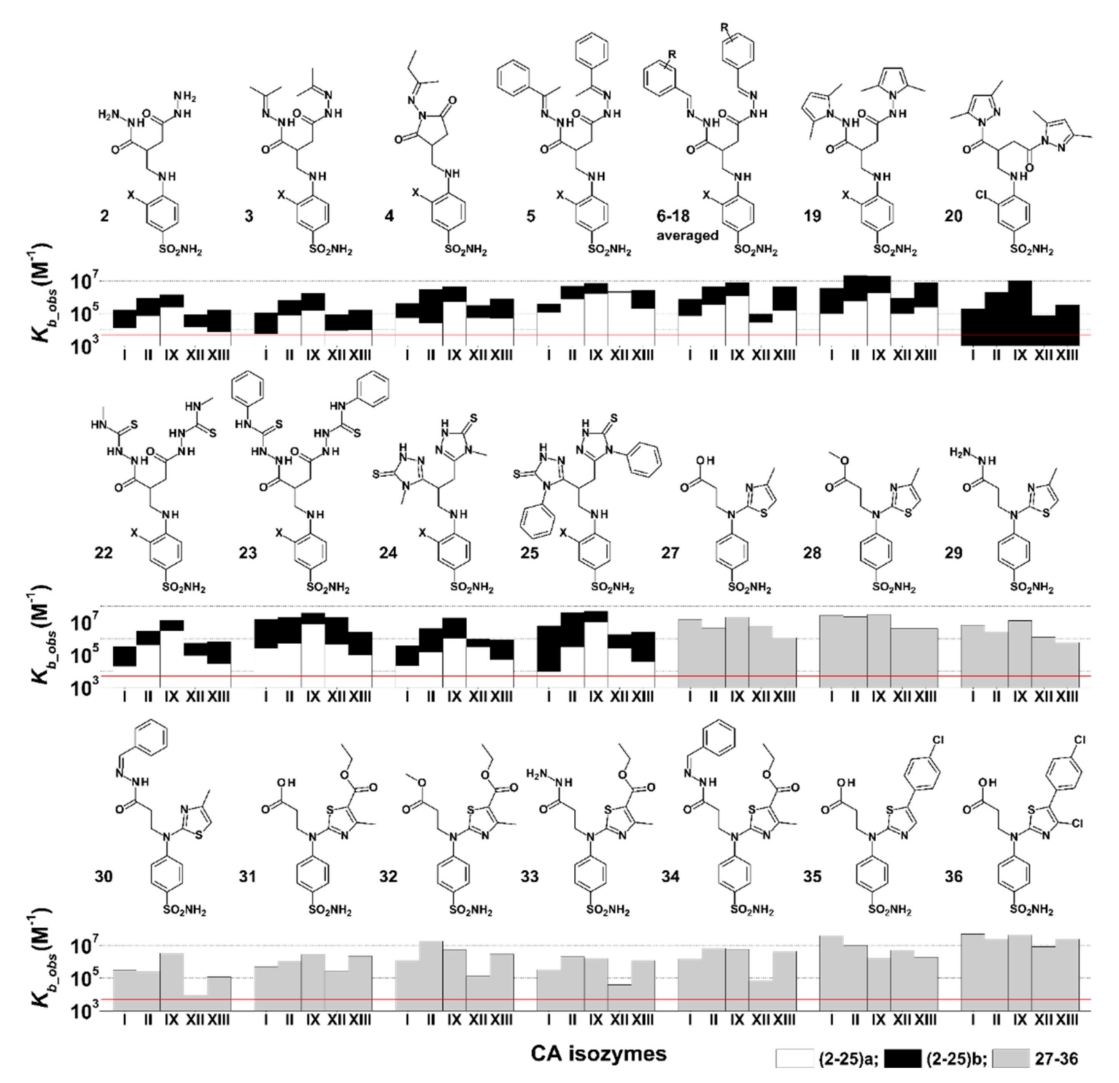
The observed binding constants (*K_b_obs_*) of compounds **2**(**a**,**b**)**–19**(**a**,**b**), **20**, **22**(**a**,**b**)**–25**(**a**,**b**), and **27**–**36** interaction with CAI, CAII, CAIX, CAXII, and CAXIII. Compound chemical structures and numbers are provided above the bar charts showing the *K_b_obs_*. For compounds **2**–**25**, X denotes –H for **a** compounds (white bars) and –Cl for **b** compounds (black bars, always higher than the white ones). The red line shows the *K_b_obs_* value of 5000 M^−1^, the lowest detectable *K_b_obs_* by the FTSA experiment.

The binding affinities of 19 compounds for all 12 catalytically active human CA isozymes were measured to test the selectivity (Table 2 and Figure S1). For clarity we divided our compounds in two groups according to chemical structure: *para*-*N* β,γ-amino acid (**2a**,**b**, **3a**,**b**, **5a**,**b**–**25a**,**b**) and *para*-*N* β-amino acid and thiazol (compounds **27**–**36**) bearing benzenesulfonamide derivatives. The binding properties of both groups will be described separately. The affinity of the compounds for five CA isozymes is shown in Figure 3.

**Figure 4:**
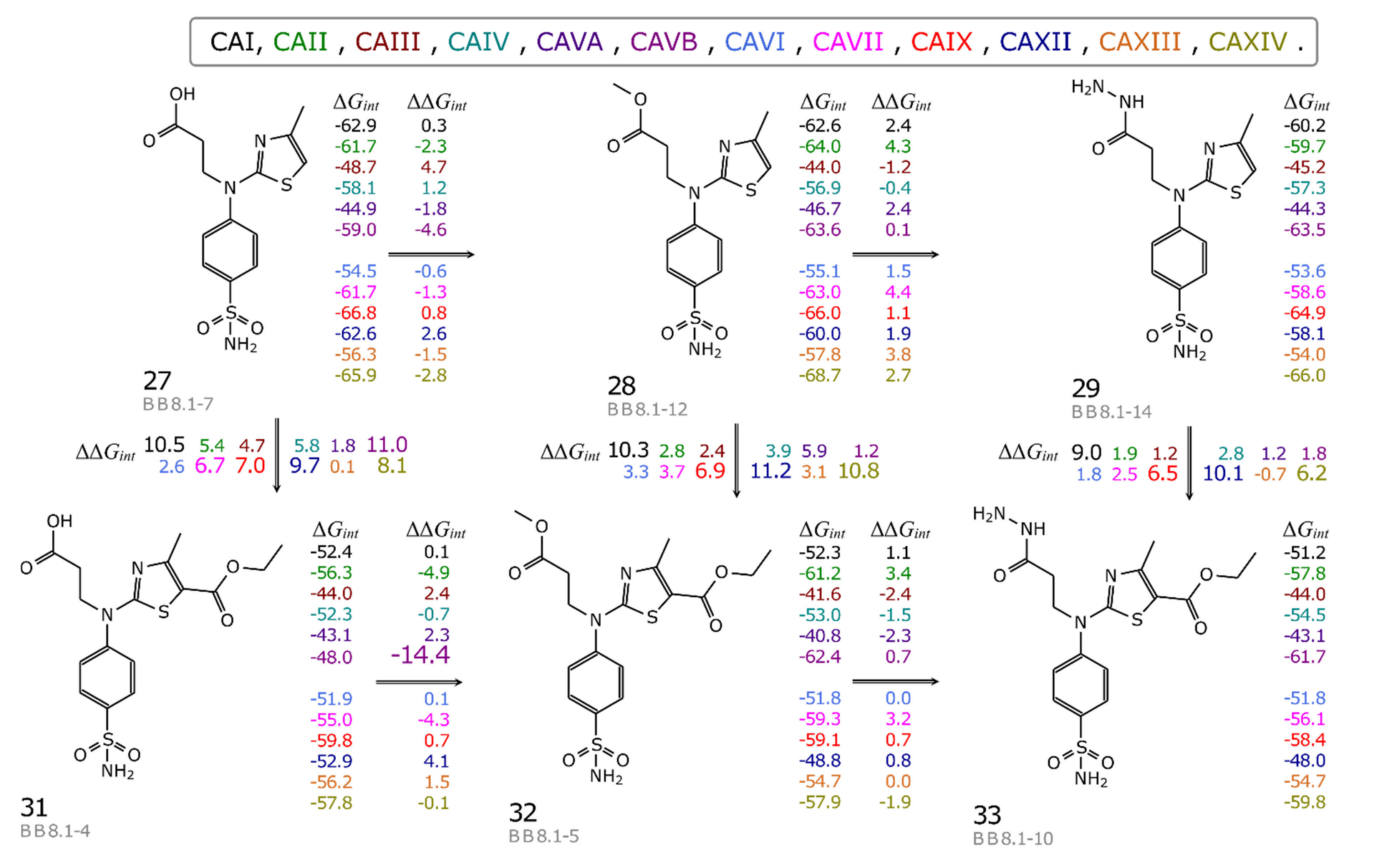
The map of correlation between compound chemical structures and the changes of the standard intrinsic Gibbs energies of binding (kJ/mol). The energies for compound binding to each CA isozyme (colored according to the list above the map) are listed on the right of each compound, while the differences between the values of neighboring compounds are listed above and below the connecting arrows.

For some compounds, the p*K_a_* values for the sulfonamide group were measured and used to calculate the intrinsic, pH-independent dissociation constants or changes in Gibbs energy [40] (Figure 4).

**Figure 5:**
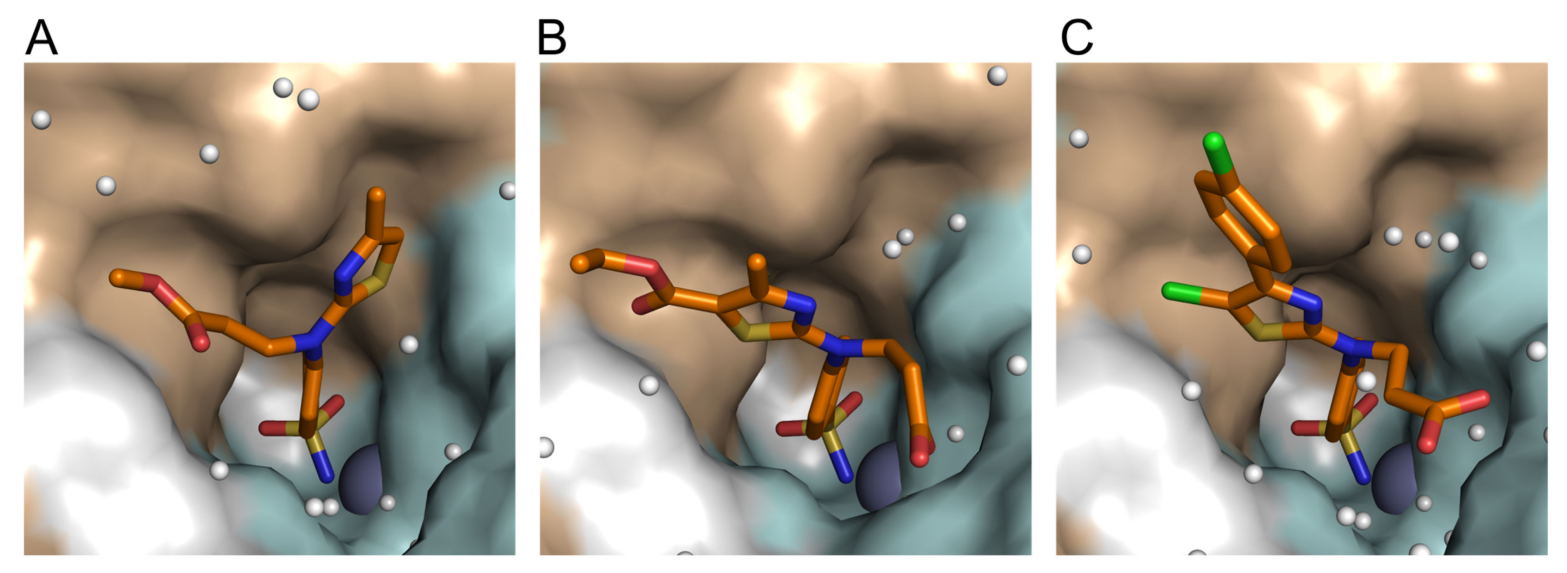
The X-ray crystallographic structures of (**A**) compound **28** (BB8.1-12) (PDB ID: 7QGZ); (**B**) compound **31** (BB8.1-4) (PDB ID: 7QGY); and (**C**) compound **36** (BB8.3-3) (PDB ID: 7QGX) in the active site of CAII. The zinc ion is shown as a grey sphere and water molecules as small white spheres. The protein surface of CAII is colored brown for hydrophobic residues (A, G, V, I, L, F) and cyan for the residues with polar side chains (R, K, H, E, D, N, Q, T, S, C). The refinement statistics are provided in the Supplementary Materials.

In addition, the binding strength of several compounds was confirmed by isothermal titration calorimetry [41]. The binding data of compounds **28**, **31**, and **36** to CA II and compound **32** binding to CA VB were in good agreement; the *K_d___obs_* values measured by both methods differed by less than three-fold. Structural characterization of three compounds’ (**28**, **31**, and **36**) binding was performed by determining their X-ray crystal structures in complexes with CAII (Figure 5).

#### 2.3.1. *para*-*N* β,γ-Amino Acid-Substituted Benzenesulfonamide Derivatives (**2**–**25a,b**) Binding to CA

Tested compounds **2**–**20a**,**b** and **22**–**25a**,**b** can be grouped into pairs differing by a chlorine atom at *meta*- position on the benzene ring: compounds **b**—chlorinated and compounds **a**—non-chlorinated. Chlorinated compounds exhibited significantly greater *observed* affinity towards most CA isozymes (Figure 3 or Table 1), with on average a 40-fold increase in affinity for CAI and CAXIII, 15–20-fold for CAII, CAVII, and CAXIV, and marginally increased affinity toward CAIII, CAIV, CAIX, and CAXII.

Compounds **2a** and **2b**, which are precursors to compounds **3a**,**b**–**25a**,**b**, exhibited weak interaction with CAI, CAII, CAIX, CAXII, and CAXIII (*K_d_obs_* 0.83–130 μM). Compound **2b** had a moderately strong interaction with CAIX (*K_d_obs_* = 0.83 μM). The size and hydrophobicity of substituents increases in the direction **3a**→**6a** and **3b**→**6b**, resulting in the increment of affinity toward CAI, CAII, CAIX, CAXII, and CAXIII. However, observed affinity for CAXII of compounds **5a**→**6a** decreased 50 times. Ligand flexibility could also play a role; ligands **5a** and **5b** having an extra rotatable bond might allow better interaction of the benzene ring with hydrophobic amino acids of CAXII, compared to **6a** and **6b**. Compound **6b** was also tested with all other catalytically active CA isozymes (Table 2); it exhibited weak interaction with CAIII, CAIV, CAVA, and CAXII (*K_d_obs_* ≥ 200,000 nM, 4200 nM, 20,000 nM, and 7900 nM, respectively), moderately strong interaction with CAI and CAVI, and strong interaction with CAII, CAVB, CAVII, CAIX, CAXIII, and CAXIV (*K_d_obs_* in the range of 100–290 nM). Although compounds **4a** and **4b** contain closed dioxopyrrolidine ring structure (Figure 3), the affinities for CAI, CAII, CAIX, CAXII, and CAXIII did not differ from the other compounds described in this section. No remarkable selectivity toward any CA isozyme was observed for compounds **2a**,**b**, **3a**,**b**, **5a**,**b**, **6a**,**b**, nor even **4a**,**b**, but usually there was an increase in observed affinity (up to 10–100 times) for CAIX compared to other tested CAs.

Ligands **7**–**18a** and **7**–**18b** are structurally similar to **6a** and **6b**, only differing by symmetric substituents on the phenyl ring. Small substituents such as -OH, -Cl, -F, -OCH_3_, -COOH, and -NO_2_ as well as their positions in the phenyl ring did not significantly affect the binding affinity towards CAs. Compounds **7**–**18a** exhibited low affinity for tested CA isozymes, with no significant selectivity for a particular CA. The *K_d_* ranges for CAI were 5.0–36 μM, CAII—1.3–5.0 μM, CAIX—0.5–3.3 μM, CAXII—20–100 μM, and for CAXIII—1.7–23 μM. Chlorinated compounds **7**–**18b** proved to be of more interest, with a generally increased observed affinity toward CAII, CAIX, and CAXIII compared to analogous non-chlorinated compounds **7**–**18a** (Figure 3). Compounds **7**–**9b**, having -OH substituent in, respectively, *ortho-*, *meta-*, and *para-*positions on the benzene ring, interacted similarly with CAI, II, IX, XII, and XIII as compound **6b**. Compound **14b**, having OCH_3_ substitute in *para-*position on the benzene ring, exhibited the strongest interaction with CAII and CAXIII (*K_d_obs_* 59 nM and 66 nM, respectively), although binding was not selective, due to moderately strong interaction with CAI and CAIX *(K_d_obs_* 510 nM and 290 nM, respectively).

Thiourea derivatives **22**–**25a**,**b** (Figure 3) exhibited similar binding patterns to CAI, CAII, CAIX, CAXII, and CAXIII, showing increases in observed binding affinity going from CAI to CAIX. Compounds **22a**–**25a** had a trend of decreasing affinity toward CA isozymes going from CAIX to CAXIII. A similar trend was not observed for their chlorinated analogs **22**–**25b**, which showed a stronger affinity for CAIX but did not have significant differences in binding strength for CAXII and CAXIII (Table 1). Compounds **22a**,**b**, **23b**, and **25b** were also tested with all catalytically active CA isozymes, and no selectivity was observed. Compound **22a** exhibited weak binding to all tested CA isozymes (micromolar *K_d_obs_* values). This is consistent with a trend of non-chlorinated compounds having weaker observed binding affinity to CAs than chlorinated analogs. On the other hand, **22a** interacted with a stronger affinity with CAIX (*K_d_obs_* 330 nM). Compound **22b** bound CA isozymes more than three times stronger than **22a** and showed the highest affinities for CAIX and CAXIV (*K_d_obs_* values of 100 nM and 130 nM, respectively). The *K_d_obs_* decreased in the direction **22b**→**23b**→**25b** for most CAs, especially for CAII (400 nM→50 nM→25 nM), CAIX (100 nM→33 nM→25 nM), and CAXIV (from 130 nM→50 nM→25 nM). Compound **25b** also had an unusually strong interaction with CAIII (*K_d_obs_* 5000 nM, Table 2), despite having large substituents, which usually contribute to steric hindrance and weak binding to CAIII.

#### 2.3.2. *para*-*N* β-Amino Acid and Thiazol Bearing Benzenesulfonamide Derivatives (**27**–**36**) Binding to CA

Compounds **27–6** had greater asymmetry than compounds **2**–**25a**,**b** (Figure 3). Compounds **27**–**34** and **36** were tested with all catalytically active CA isozymes. In general, compounds from this group exhibited weak interaction with CAIII and CAVA (Table 2), with the *K_d_obs_* values ranging from 17 µM to ≥200 µM (200 µM being the weakest detectable *K_d_obs_* value). Compounds **27**–**30** differed by substitutes on β-aminoacid carbonyl group, increasing in size (–OH, –OCH_3_, –NHNH_2_, –NHN=CPh). Carboxylic acid **27** strongly bound to CAI and CAIX (*K_d_obs_* 67 nM and 48 nM, respectively) but also had moderately strong interactions with CAII, CAVII, CAXII, and CAXIV (*K_d_obs_* in the ranges 170–250 nM). Esterification of thiazole in **27** to compound **28** increased affinity toward CAII, CAVA, CAVB, and CAXIV (*K_d_obs_* values of 45 nM, 50 µM, 46 nM, 29 nM of compound **28**, respectively). Of special interest was an increase in affinity toward CAVB in the direction **27→28**, with *K_d_obs_* value decreasing from 560 nM to 46 nM (Table 2). The hydrazide **29** interacted with all CAs slightly weaker than **28**. Hydrazone **30**, having by far the largest, hydrophobic, and inflexible –NHN=CPh group, showed a significantly decreased binding strength to all tested CA isozymes (*K_d_*s in the micromolar range). Compounds **31**–**34** were analogous to compounds **27**–**30** but had an additional ester substitute on the thiazole ring, which made compounds larger and less flexible than their counterparts (Figure 3). Ligands **31**–**33** showed similar or significantly weaker interactions with most CA isozymes than their analogous compounds without ethylester group on thiazole ring, **27**–**29**. However, of all tested compounds, the strongest interaction was measured between **32** and CA VB (*K_d_* = 30 nM). Binding affinities of compound **34** towards CAII, CAVB, and CAIX were in the range of 100 nM. Compounds **35** and **36** showed the strongest affinities to CAI (*K_d_obs_* 25 nM (Table 1) and 20 nM (Table 2), respectively). Furthermore, compound **36** interacted strongly with CAII, CAVII, CAIX, and CAXIII (*K_d_obs_* in the range of 23–40 nM), showing no selectivity toward CAs.

#### 2.3.3. Intrinsic Thermodynamics of Compounds **27**–**36** Binding to CAs

We calculated the intrinsic dissociation constants (*K_d_int_*, Table 3) and intrinsic Gibbs energy (Δ*G_int_*, Table S1) values of binding between CA isozymes and compounds **27**–**29**, **31**–**33**, and **36** and constructed a compound structure–affinity correlation map (Figure 4) which demonstrates how small changes in functional groups influence compound capability to bind a particular CA isozyme. The intrinsic binding parameters represent the actual binding reaction between the deprotonated sulfonamide and the Zn-bound water form of CA. To calculate the intrinsic binding parameters by Equations (4) and (5), the p*K_a_*s of tested sulfonamides were measured spectrophotometrically by measuring absorption at different pH values. The average p*K_a_* values of compounds **27**–**29**, **31**, **32**, **35**, and **36** were determined to be 10.1. Calculation of the intrinsic values has a significant advantage over the observed values, because the intrinsic values eliminate the pH and buffer effects that may be misleading in the structural origins of the altered affinity [5,42,43].

All compounds exhibited relatively weak binding to CAIII (*K_d_int_* at limit of detection for compounds **28**–**34** and *K_d_int_* values of 6 and 2.6 nM, respectively, for compounds **27** and **36**) and CAVA (*K_d_int_* at limit of detection for all tested compounds except compound **28**, which binds with *K_d_int_* of 13 nM (Table 3)). Figure 4 depicts some compounds in which minor structural changes had a significant effect on affinity for carbonic anhydrases. Esterification of carboxyl group (directions **27→28** and **31→32**) (Figure 4) was favorable or had negligible effect on the Gibbs energy for all CAs except for CAXII, which showed a slight decrease in affinity. Addition of methoxy group leads to a large gain in Δ*G_int_* for CAVB (ΔΔ*G_int_* (**27→28**) **=** -4.6 and ΔΔ*G_int_* (**31→32**) **=** −14.4 kJ/mol). Further modification of the methoxy with hydrazide group (directions **28→29** and **32→33**) decreased the Gibbs energy of binding to all CAs but did not change that to CAVB (ΔΔ*G_int_* (**28→29**) **=** 0.1 and ΔΔ*G_int_* (**32→33**) **=** 0.7 kJ/mol). Interestingly, addition of ethoxycarbonyl group on the thiazole ring (directions **27→31**, **28→32**, and **29→33**) resulted in the decrease of Δ*G_int_* or had negligible effect for all CAs, with the largest reduction of affinity for CAXII (ΔΔ*G_int_* (**28→32**) **=** 11.2 kJ/mol). In summary, the intrinsic binding data showed that esterification increased the binding affinity for the majority of CA isozymes (Table 3). Thus, it would be very interesting to investigate the influence of small and hydrophobic substitutes on the carboxyl group with the perspectives of finding possible new lead compounds for use as inhibitors for CAVB while binding weakly to CAVA isozyme and possibly having increased selectivity for CAVB isozyme.

#### 2.3.4. X-ray Crystallography of Human CAII Complexes with Inhibitors

The crystal structures of compounds **28**, **31**, and **36** in the complex with CAII were determined by X-ray crystallography (Figure 5). The electron density maps of the ligands bound in the active site of CAII are shown in Figure S2 in Supplementary Materials. The data collection and refinement statistics are presented in Table S2. In all three structures, the benzenesulfonamide ring occupies a similar position (Figure 5) due to the interaction with Leu198; only the positions of the substituents differed. The carboxy group of compounds **31** and **36** faces the hydrophilic side (colored cyan in Figure 5) of the active site of CAII and forms hydrogen bonds with Asn62, Asn67, or water molecules, while the methyl ester in compound **28** adheres to a more hydrophobic surface (colored brown in Figure 5). The 2D schemes of the interactions are shown as LigPlot schemes based on X-ray crystallography in Figure S3. The thiazole-based substituent interacts with Phe131 and was found on either side of the Phe131 side chain depending on the other substituents (Figure 5). Thus, the smallest thiazole substituent of compound **28** occupied the “right side” of the hydrophobic pocket, while the larger thiazole-based substituents of compounds **31** and **36** occupied the more spacious “left side” of the pocket, because they would not fit in the pocket on the “right side” without changing the position of the benzenesulfonamide due to steric hindrance.

## 3. Materials and Methods

### 3.1. Organic Synthesis

Reagents were purchased from Sigma-Aldrich (St. Louis, MO, USA). The melting points were determined on a MEL-TEMP (Electrothermal, Bibby Scientific Company, Burlington, NJ, USA) melting point apparatus and were uncorrected. The IR spectra (ν, cm^−1^) were recorded on a Perkin–Elmer Spectrum BX FT–IR spectrometer using KBr pellets. The ^1^H and ^13^C NMR spectra were recorded in DMSO-*d*_6_ medium on Brucker Avance III (400, 101 MHz) spectrometer (see Supplementary Materials, Figures S4–S103). The chemical shifts (δ) were reported in parts per million (ppm), calibrated from TMS (0 ppm) as an internal standard for ^1^H NMR and DMSO-*d*_6_ (39.43 ppm) for ^13^C NMR. Mass spectra were obtained on a Bruker maXis UHR-TOF mass spectrometer (Bruker Daltonics, Bremen, Germany) with ESI ionization. Elemental analysis was performed on a CE-440 elemental analyzer (Exeter Analytical Inc., North Chelmsford, MA, USA). The reaction course and purity of the synthesized compounds were monitored by TLC using aluminium plates pre-coated with the silica gel 60 F_254_ (MerckKGaA, Darmstadt, Germany). Single crystals of **4a** were investigated on a Rigaku, XtaLAB Synergy, Dualflex, HyPix diffractometer. The crystal was kept at 170.0(1) K during data collection. Using Olex2 [44], the structure was solved with the SIR2014 [45] structure solution program using Direct Methods and refined with the olex2.refine [46] refinement package using Gauss–Newton minimization.

#### 3.1.1. General Procedure for the Synthesis of Hydrazides **2a,b**

A mixture of carboxylic acid **1a** (3.70 g, 13 mmol) or **1b** (4.14 g, 13 mmol) and hydrazine monohydrate (6.3 mL, 130 mmol) was heated at reflux for 4 h. The reaction mixture was cooled down, diluted with propan-2-ol, and heated until reflux. Then, the suspension was left to cool down, and the precipitate was filtered off and recrystallized from propan-2-ol and water mixture.

##### 4-((2-(Hydrazinecarbonyl)-4-hydrazineyl-4-oxobutyl)amino)benzenesulfonamide (**2a**)

White solid, yield 3.58 g (84%); m.p. 164–165 °C; IR (KBr) (v, cm^−1^): 3399, 3301, 3213, 1607, 1603; ^1^H NMR (400 MHz, DMSO-*d*_6_) δ (ppm): 2.11–2.37 (m, 2H, CH_2_), 2.79–2.96 (m, 1H, CH), 2.99–3.33 (m, 2H, NCH_2_), 4.32 (br. s, 4H, 2NH_2_), 6.39 (t, 1H, *J =* 6.0 Hz, NH), 6.60–7.54 (m, 6H, H_Ar_, SO_2_NH_2_), 9.01, 9.11 (2s, 2H, 2NH); ^13^C NMR (101 MHz, DMSO-*d*_6_) (δ, ppm): 33.66, 44.40, 110.86, 127.29, 130.16, 151.13, 169.84, 172.00; HRMS (ESI) for C_11_H_18_N_6_O_4_S + H^+^, calcd. 330.1110, found 330.1115 [M + H^+^].

##### 3-Chloro-4-((2-(hydrazinecarbonyl)-4-hydrazineyl-4-oxobutyl)amino)benzenesulfonamide (**2b**)

White solid, yield 3.81 g (80%); m.p. 183–184 °C; IR (KBr) (v, cm^−1^): 3312, 3213, 1635, 1616; ^1^H NMR (400 MHz, DMSO-*d*_6_) δ (ppm): 2.16–2.41 (m, 2H, CH_2_), 2.90–3.07 (m, 1H, CH), 3.12–3.45 (m, 2H, NCH_2_), 4.29 (br. s, 4H, 2NH_2_), 6.05 (t, 1H, *J =* 5.7 Hz, NH), 6.81–7.72 (m, 5H, H_Ar_, SO_2_NH_2_), 9.03, 9.21 (2s, 2H, 2NH); ^13^C NMR (101 MHz, DMSO-*d*_6_) (δ, ppm): 33.46, 44.62, 110.26, 116.83, 126.13, 126.66, 131.25, 146.29, 169.79, 171.89; HRMS (ESI) for C_11_H_11_ClN_2_O_5_S + H^+^, calcd. 319.0155, found 319.0150 [M + H^+^].

#### 3.1.2. General Procedure for the Synthesis of Hydrazones **3a,b**

Hydrazide **2a** (0.33 g, 1 mmol) or **2b** (0.36 g, 1 mmol) was dissolved in acetone (15 mL), and the solution was heated at reflux for 2 h. The reaction mixture was cooled down, and the precipitate was filtered off.

##### 4-((4-Oxo-2-(2-(propan-2-ylidene)hydrazine-1-carbonyl)-4-(2-(propan-2-ylidene)hydrazineyl)butyl)amino)benzenesulfonamide (**3a**)

White solid, yield 0.28 g (68%); m.p. 79–80 °C; IR (KBr) (v, cm^−1^): 3391, 3371, 3250, 1687, 1660; ^1^H NMR (400 MHz, DMSO-*d*_6_) δ (ppm): 1.63–2.10 (m, 12H, 4CH_3_), 2.52–3.54 (m, 5H, CH_2_CO, CH, NCH_2_), 6.38–7.58 (m, 7H, H_ar_, NH, NH_2_), 9.84–10.19 (m, 2H, 2CONH); ^13^C NMR (101 MHz, DMSO-*d*_6_) (δ, ppm): 17.02, 17.12, 17.48, 17.60, 24.94, 25.11, 31.94, 33.08, 35.91, 43.51, 44.69, 110.88, 127.26, 130.10, 149.73, 150.31, 150.93, 151.21, 154.96, 156.07, 169.35, 173.19, 174.53; HRMS (ESI) for C_17_H_26_N_6_O_4_S + H^+^, calcd. 411.1814, found 411.1809 [M + H^+^].

##### 3-Chloro-4-((4-oxo-2-(2-(propan-2-ylidene)hydrazine-1-carbonyl)-4-(2-(propan-2-ylidene)hydrazineyl)butyl)amino)benzenesulfonamide (**3b**)

White solid, yield 0.31 g (70%); m.p. 133–134 °C; IR (KBr) (v, cm^−1^): 3361, 3227, 1652, 1594; ^1^H NMR (400 MHz, DMSO-*d*_6_) δ (ppm): 1.67–2.06 (m, 12H, 4CH_3_), 2.54–3.53 (m, 5H, CH_2_CO, CH, NCH_2_), 5.94–6.43 (m, 1H, NH,), 6.84–7.74 (m, 5H, H_ar_, NH_2_), 9.83–10.25 (m, 2H, 2CONH); ^13^C NMR (101 MHz, DMSO-*d*_6_*)* (δ, ppm): 17.04, 17.16, 17.50, 17.61, 24.93, 25.14, 35.26, 36.10, 43.99, 44.80, 110.34, 116.59, 126.10, 126.10, 131.25, 146.21, 149.88, 149.88, 151.10, 163.12, 173.04, 174.54; HRMS (ESI) for C_17_H_25_ClN_6_O_4_S + H^+^, calcd. 445.1425, found 445.1419 [M + H^+^].

#### 3.1.3. General Procedure for the Synthesis of Hydrazones **4a,b**

One mmol of hydrazide **2a** (0.33 g) or **2b** (0.36 g) was dissolved in propan-2-ol (15 mL), and methyl ethyl ketone (0.36 mL, 4 mmol) was added dropwise to the solution. The reaction mixture was heated at reflux for 3 h; then it was cooled down, and the precipitate was filtered off and washed with diethyl ether.

##### 4-(((1-(Butan-2-ylideneamino)-2,5-dioxopyrrolidin-3-yl)methyl)amino)benzenesulfonamide (**4a**)

White solid, yield 0.25 g (71%); m.p. 117–118 °C; IR (KBr) (v, cm^−1^): 3332, 3246, 1693, 1597; ^1^H NMR (400 MHz, DMSO-*d*_6_) δ (ppm): 0.82–1.24 (m, 3H, CH_3_), 1.70 (s, 3H, CH_2_CH_3_), 2.01–2.45 (m, 2H, CH_2_CH_3_), 2.58–3.14 (m, 2H, CH_2*pyr*_), 3.25–3.59 (m, 3H, CH, NCH_2_), 6.45–6.58 (m, 1H, NH), 6.68 (dd, 2H, *J =* 8.6, 5.7 Hz, H_ar_), 6.93 (s, 2H, NH_2_), 7.52 (dd, 2H, *J =* 8.9, 2.5 Hz, H_ar_); ^13^C NMR (101 MHz, DMSO-*d*_6_) (δ, ppm): 10.30, 18.48, 30.81, 31.41, 31.44, 37.79, 42.55, 42.71, 111.04, 111.09, 127.37, 127.40, 130.72, 151.01, 151.06, 171.70, 173.56, 174.33, 176.03, 182.62; HRMS (ESI) for C_15_H_20_N_4_O_4_S + H^+^, calcd. 353.1284, found 353.1289 [M + H^+^].

##### 4-(((1-(Butan-2-ylideneamino)-2,5-dioxopyrrolidin-3-yl)methyl)amino)-3-chlorobenzenesulfonamide (**4b**)

Brown solid, yield 0.29 g (74%); m.p. 92–93 °C; IR (KBr) (v, cm^−1^): 3323, 3247, 1694, 1592; ^1^H NMR (400 MHz, DMSO-*d*_6_) δ (ppm): 0.84–1.16 (m, 3H, CH_3_), 1.69 (s, 3H, CH_2_CH_3_), 2.05–2.47 (m, 2H, CH_2_CH_3_), 2.53–2.93 (m, 2H, CH_2*pyr*_), 3.11–3.79 (m, 3H, CH, NCH_2_), 6.32 (br. s, 1H, NH), 6.80–7.0 (m, 1H, H_ar_), 7.13 (s, 2H, NH_2_), 7.48–7.71 (m, 2H, H_ar_); ^13^C NMR (101 MHz, DMSO-*d*_6_*)* (δ, ppm): 10.29, 18.41, 21.90, 24.80, 25.50, 30.77, 31.40, 37.18, 38.14, 43.02, 43.29, 110.24, 110.39, 117.07, 126.14, 126.21, 126.88, 131.63, 146.12, 171.67, 173.62, 174.32, 176.12, 182.71; HRMS (ESI) for C_15_H_19_ClN_4_O_4_S + H^+^, calcd. 387.0894, found 387.0890 [M + H^+^].

#### 3.1.4. General Procedure for the Synthesis of Hydrazones **5a,b**

One mmol of hydrazide **2a** (0.33 g) or **2b** (0.36 g) was dissolved in propan-2-ol (15 mL), and acetophenone (0.35 mL, 3 mmol) was added dropwise to the solution. The reaction mixture was heated at reflux for 2 h; then it was cooled down, and the precipitate was filtered off and washed with diethyl ether and n-hexane.

##### 4-((4-Oxo-2-(2-(1-phenylethylidene)hydrazine-1-carbonyl)-4-(2-(1-phenylethylidene)hydrazineyl)butyl)amino)benzenesulfonamide (**5a**)

White solid, yield 0.38 g (72%); m.p. 162–163 °C; IR (KBr) (v, cm^−1^): 3378, 3244, 1652, 1600; ^1^H NMR (400 MHz, DMSO-*d*_6_) δ (ppm): 1.93–2.38 (m, 6H, 2CH_3_), 2.67–3.61 (m, 5H, CH_2_CO, CH, NCH_2_), 6.42–8.09 (m, 17H, H_ar_, NH, NH_2_), 10.18–10.90 (m, 2H, 2CONH); ^13^C NMR (101 MHz, DMSO-*d*_6_) (δ, ppm): 13.57, 13.92, 14.25, 14.76, 26.75, 30.82, 37.81, 42.75, 43.79, 44.72, 110.78, 110.95, 111.07, 118.89, 126.02, 126.13, 126.30, 126.48, 126.50, 127.37, 127.42, 128.17, 128.30, 128.39, 128.46, 128.71, 129.03, 129.79, 130.09, 130.74, 133.22, 138.23, 147.15, 147.82, 151.04, 151.10, 173.81, 174.36, 175.47, 176.06; HRMS (ESI) for C_27_H_30_N_6_O_4_S + H^+^, calcd. 535.2127, found 535.2123 [M + H^+^].

##### 3-Chloro-4-((4-oxo-2-(2-(1-phenylethylidene)hydrazine-1-carbonyl)-4-(2-(1-phenylethylidene)hydrazineyl)butyl)amino)benzenesulfonamide (**5b**)

White solid, yield 0.43 g (75%); m.p. 158–159 °C; IR (KBr) (v, cm^−1^): 3345, 3248, 1700, 1592; ^1^H NMR (400 MHz, DMSO-*d_6_*) δ (ppm): 2.10–2.32 (m, 6H, 2CH_3_), 2.54–3.24 (m, 5H, CH_2_CO, CH, NCH_2_), 6.23–6.42 (m, 1H, NH), 6.85–7.99 (m, 15H, H_ar_, NH_2_), 10.26–10.83 (m, 2H, 2CONH); HRMS (ESI) for C_27_H_29_ClN_6_O_4_S + H^+^, calcd. 569.1738, found 569.1735 [M + H^+^].

#### 3.1.5. General Procedure for the Synthesis of Hydrazones **6a,b**–**18a,b**

The mixture of hydrazide **2a** (0.17 g, 0.5 mmol) or **2b** (0.18 g, 0.5 mmol) with corresponding aldehyde (1.5 mmol) and propan-2-ol (15 mL) was heated at reflux for 2 h. Then, the reaction mixture was cooled down, and precipitate was filtered off and recrystallized from 1,4-dioxane.

##### 4-((2-(2-(Benzylidene)hydrazine-1-carbonyl)-4-(2-(benzylidene)hydrazineyl)-4-oxobutyl)amino)benzenesulfonamide (**6a**)

White solid, yield 0.21 g (84%); m.p. 208–209 °C; IR (KBr) (v, cm^−1^): 3364, 3239, 1655, 1601; ^1^H NMR (400 MHz, DMSO-*d_6_*) δ (ppm): 2.79–3.59 (m, 5H, CH_2_CO, CH, NCH_2_), 6.57–7.75 (m, 17H, H_ar_, NH, NH_2_), 7.94–8.23 (m, 2H, NCH), 11.21–11.65 (m, 2H, 2CONH); ^13^C NMR (101 MHz, DMSO-*d_6_*) (δ, ppm): 32.17, 32.68, 33.64, 36.07, 43.81, 44.70, 110.81, 110.90, 126.68, 126.71, 126.74, 126.92, 126.96, 127.33, 127.40, 128.78, 128.82, 129.69, 129.77, 130.23, 134.19, 134.26, 134.35, 142.68, 142.95, 143.23, 145.80, 146.26, 151.01, 151.12, 167.33, 169.60, 172.67, 172.96, 174.45, 174.62; HRMS (ESI) for C_25_H_26_N_6_O_4_S + H^+^, calcd. 507.1814, found 507.1809 [M + H^+^].

##### 4-((2-(2-(Benzylidene)hydrazine-1-carbonyl)-4-(2-(benzylidene)hydrazineyl)-4-oxobutyl)amino)-3-chlorobenzenesulfonamide (**6b**)

White solid, yield 0.24 g (89%); m.p. 212–213 °C; IR (KBr) (v, cm^−1^): 3406, 3243, 1662, 1598; ^1^H NMR (400 MHz, DMSO-*d_6_*) δ (ppm): ^1^H NMR (400 MHz, DMSO-*d_6_*) δ (ppm): 2.63–3.63 (m, 5H, CH_2_CO, CH, NCH_2_), 6.13–6.40 (m, 1H, NH), 6.92–7.74 (m, 15H, H_ar_, NH_2_), 7.91–8.27 (m, 2H, NCH), 11.22–11.67 (m, 2H, 2CONH); ^13^C NMR (101 MHz, DMSO-*d_6_*) (δ, ppm): 32.23, 33.65, 35.91, 44.27, 109.98, 116.89, 126.06, 126.07, 126.07, 126.67, 126.70, 126.98, 128.78, 129.69, 131.19, 134.14, 134.26, 142.72, 142.97, 143.43, 143.63, 146.22, 153.77, 172.88, 174.68; HRMS (ESI) for C_25_H_25_ClN_6_O_4_S + H^+^, calcd. 541.1425, found 541.1419 [M + H^+^].

##### 4-((2-(2-(2-Hydroxybenzylidene)hydrazine-1-carbonyl)-4-(2-(2-hydroxybenzylidene)hydrazineyl)-4-oxobutyl)amino)benzenesulfonamide (**7a**)

White solid, yield 0.20 g (74%); m.p. 177–178 °C; IR (KBr) (v, cm^−1^): 3290, 3252, 3163, 1668, 1602; ^1^H NMR (400 MHz, DMSO-*d*_6_) δ (ppm): 2.56–3.65 (m, 5H, CH_2_CO, CH, NCH_2_), 6.57–7.78 (m, 15H, H_ar_, NH, NH_2_), 8.20–8.47 (m, 2H, NCH), 9.81–10.36 (m, 1H, OH), 11.06–11.40 (m, 2H, 2CONH), 11.61–12.01 (m, 1H, OH); HRMS (ESI) for C_25_H_26_N_6_O_6_S + H^+^, calcd. 539.1713, found 539.1709 [M + H^+^].

##### 3-Chloro-4-((2-(2-(2-hydroxybenzylidene)hydrazine-1-carbonyl)-4-(2-(2-hydroxybenzylidene)hydrazineyl)-4-oxobutyl)amino)benzenesulfonamide (**7b**)

White solid, yield 0.22 g (76%); m.p. 204–205 °C; IR (KBr) (v, cm^−1^): 3294, 3247, 3190, 1667, 1593; ^1^H NMR (400 MHz, DMSO-*d*_6_) δ (ppm): ^1^H NMR (400 MHz, DMSO-*d*_6_) δ (ppm): 2.52–3.75 (m, 5H, CH_2_CO, CH, NCH_2_), 6.17–6.47 (m, 1H, NH), 6.57–8.47(m, 15H, H_ar_, NCH, NH_2_), 9.95–10.19 (m, 1H, OH), 11.04–11.43 (m, 2H, 2CONH), 11.66–11.94 (m, 1H, OH); ^13^C NMR (101 MHz, DMSO-*d*_6_) (δ, ppm): 31.41, 33.82, 35.36, 44.03, 44.60, 116.12, 116.33, 116.54, 116.99, 118.20, 118.57, 118.60, 119.32, 119.62, 120.09, 120.39, 126.10, 126.80, 129.35, 130.85, 131.36, 131.45, 133.25, 141.01, 146.21, 146.64, 147.19, 156.35, 157.28, 157.32, 158.65, 162.81, 166.77, 167.18, 169.03, 169.37, 172.22, 172.49, 174.01, 174.34; HRMS (ESI) for C_25_H_25_ClN_6_O_6_S + H^+^, calcd. 573.1323, found 573.1322 [M + H^+^].

##### 4-((2-(2-(3-Hydroxybenzylidene)hydrazine-1-carbonyl)-4-(2-(3-hydroxybenzylidene)hydrazineyl)-4-oxobutyl)amino)benzenesulfonamide (**8a**)

White solid, yield 0.22 g (81%); m.p. 192–193 °C; IR (KBr) (v, cm^−1^): 3444, 3373, 3283, 3172, 1657, 1597; ^1^H NMR (400 MHz, DMSO-*d*_6_) δ (ppm): 2.57–3.60 (m, 5H, CH_2_CO, CH, NCH_2_), 6.58–7.59 (m, 15H, H_ar_, NH, NH_2_), 7.85–8.15 (m, 2H, NCH), 9.62 (br. s,, 2H, OH), 11.16–11.62 (m, 2H, 2CONH); ^13^C NMR (101 MHz, DMSO-*d*_6_) (δ, ppm): 30.98, 31.87, 32.66, 32.67, 34.33, 36.15, 43.71, 44.71, 47.87, 110.93, 112.56, 112.63, 117.00, 117.29, 118.13, 118.37, 118.68, 127.35, 127.47, 129.83, 130.27, 135.54, 135.62, 142.98, 143.20, 146.43, 151.05, 151.14, 157.64, 157.72, 166.92, 169.31, 169.63, 172.56, 172.89, 174.52; HRMS (ESI) for C_25_H_26_N_6_O_6_S + H^+^, calcd. 539.1713, found 539.1707 [M + H^+^].

##### 3-Chloro-4-((2-(2-(3-hydroxybenzylidene)hydrazine-1-carbonyl)-4-(2-(3-hydroxybenzylidene)hydrazineyl)-4-oxobutyl)amino)benzenesulfonamide (**8b**)

White solid, yield 0.21 g (72%); m.p. 119–120 °C; IR (KBr) (v, cm^−1^): 3412, 3319, 3231, 3067, 1643, 1597; ^1^H NMR (400 MHz, DMSO-*d*_6_) δ (ppm): ^1^H NMR (400 MHz, DMSO-*d*_6_) δ (ppm): 2.61–3.62 (m, 5H, CH_2_CO, CH, NCH_2_), 6.09–6.32 (m, 1H, NH), 6.68–7.67 (m, 13H, H_ar_, NH_2_), 7.82–8.15 (m, 2H, NCH), 9.46–9.67 (m, 2H, OH), 11.12–11.63 (m, 2H, 2CONH); ^13^C NMR (101 MHz, DMSO-*d*_6_) (δ, ppm): 32.00, 32.58, 35.36, 36.18, 44.32, 44.84, 110.01, 110.46, 112.53, 112.74, 113.57, 116.88, 117.02, 117.55, 118.12, 118.74, 126.12, 126.22, 126.79, 126.87, 129.85, 131.31, 131.37, 135.43, 135.46, 135.56, 143.06, 143.27, 143.93, 146.20, 146.59, 157.64, 157.67, 166.93, 167.22, 169.22, 169.55, 172.58, 172.81, 174.29, 174.63; HRMS (ESI) for C_25_H_25_ClN_6_O_6_S + H^+^, calcd. 573.1323, found 573.1317 [M + H^+^].

##### 4-((2-(2-(4-Hydroxybenzylidene)hydrazine-1-carbonyl)-4-(2-(4-hydroxybenzylidene)hydrazineyl)-4-oxobutyl)amino)benzenesulfonamide (**9a**)

White solid, yield 0.25 g (93%); m.p. 186–187 °C; IR (KBr) (v, cm^−1^): 3442, 3356, 3288, 3190, 1657, 1602; ^1^H NMR (400 MHz, DMSO-*d*_6_) δ (ppm): 2.60–3.28 (m, 5H, CH_2_CO, CH, NCH_2_), 6.51–7.61 (m, 15H, H_ar_, NH, NH_2_), 7.83–8.12 (m, 2H, NCH), 9.90 (br. s, 2H, OH), 10.99–11.43 (m, 2H, 2CONH); ^13^C NMR (101 MHz, DMSO-*d*_6_) (δ, ppm): 32.38, 33.13, 36.47, 37.27, 44.15, 111.31, 116.12, 116.22, 125.66, 125.70, 125.78, 127.79, 127.86, 128.83, 128.88, 129.14, 129.21, 130.61, 130.65, 143.42, 143.72, 144.12, 146.56, 146.87, 147.02, 147.28, 151.48, 151.59, 151.61, 159.50, 159.57, 159.62, 159.71, 159.76, 167.47, 169.75, 172.77, 173.12, 174.44, 174.66; HRMS (ESI) for C_25_H_26_N_6_O_6_S + H^+^, calcd. 539.1713, found 539.1708 [M + H^+^].

##### 3-Chloro-4-((2-(2-(4-hydroxybenzylidene)hydrazine-1-carbonyl)-4-(2-(4-hydroxybenzylidene)hydrazineyl)-4-oxobutyl)amino)benzenesulfonamide (**9b**)

White solid, yield 0.24 g (83%); m.p. 226–227 °C; IR (KBr) (v, cm^−1^): 3434, 3327, 3236, 3079, 1633, 1593; ^1^H NMR (400 MHz, DMSO-*d*_6_) δ (ppm): ^1^H NMR (400 MHz, DMSO-*d*_6_) δ (ppm): 2.65–3.62 (m, 5H, CH_2_CO, CH, NCH_2_), 6.11–6.41 (m, 1H, NH), 6.72–7.75 (m, 13H, H_ar_, NH_2_), 7.80–8.14 (m, 2H, NCH), 9.74–9.99 (m, 2H, OH), 10.99–11.45 (m, 2H, 2CONH); ^13^C NMR (101 MHz, DMSO-*d*_6_) (δ, ppm): 32.06, 32.57, 35.31, 36.01, 44.28, 44.86, 109.99, 110.24, 115.67, 115.73, 116.88, 125.17, 125.34, 126.08, 126.77, 126.88, 128.38, 128.47, 128.67, 128.75, 131.18, 143.02, 143.94, 146.25, 159.06, 159.12, 159.21, 159.31, 169.20, 172.33, 172.58, 174.27; HRMS (ESI) for C_25_H_25_ClN_6_O_6_S + H^+^, calcd. 573.1323, found 573.1319 [M + H^+^].

##### 4-((2-(2-(2-Chlorobenzylidene)hydrazine-1-carbonyl)-4-(2-(2-chlorobenzylidene)hydrazineyl)-4-oxobutyl)amino)benzenesulfonamide (**10a**)

White solid, yield 0.25 g (86%); m.p. 174–175 °C; IR (KBr) (v, cm^−1^): 3452, 3328, 3081, 1694, 1598; ^1^H NMR (400 MHz, DMSO-*d*_6_) δ (ppm): 2.57–3.61 (m, 5H, CH_2_CO, CH, NCH_2_), 6.54–7.76 (m, 15H, H_ar_, NH, NH_2_), 8.31–8.68 (m, 2H, NCH), 11.43–11.91 (m, 2H, 2CONH); ^13^C NMR (101 MHz, DMSO-*d*_6_) (δ, ppm): 32.37, 35.98, 43.92, 110.81, 110.92, 126.58, 126.69, 127.39, 127.59, 127.77, 128.22, 129.89, 130.20, 130.24, 130.32, 130.52, 131.17, 131.42, 131.47, 131.49, 132.86, 133.06, 133.20, 134.69, 138.82, 139.04, 139.25, 142.21, 151.02, 151.10, 158.28, 169.75, 172.79, 173.05, 174.90; HRMS (ESI) for C_25_H_24_Cl_2_N_6_O_4_S + H^+^, calcd. 575.1035, found 575.1031 [M + H^+^].

##### 3-Chloro-4-((2-(2-(2-chlorobenzylidene)hydrazine-1-carbonyl)-4-(2-(2-chlorobenzylidene)hydrazineyl)-4-oxobutyl)amino)benzenesulfonamide (**10b**)

White solid, yield 0.26 g (85%); m.p. 203–204 °C; IR (KBr) (v, cm^−1^): 3438, 3337, 3077, 1688, 1602; ^1^H NMR (400 MHz, DMSO-*d*_6_) δ (ppm): ^1^H NMR (400 MHz, DMSO-*d*_6_) δ (ppm): 2.64–3.67 (m, 5H, CH_2_CO, CH, NCH_2_), 6.17–6.43 (m, 1H, NH), 6.98–8.00 (m, 13H, H_ar_, NH_2_), 8.29–8.64 (m, 2H, NCH), 11.44–11.92 (m, 2H, 2CONH); ^13^C NMR (101 MHz, DMSO-*d*_6_) (δ, ppm): 32.38, 32.53, 35.27, 35.84, 44.28, 110.01, 110.41, 116.85, 126.03, 126.42, 126.55, 126.79, 126.86, 127.41, 127.52, 127.76, 128.21, 129.85, 129.89, 130.19, 131.18, 131.37, 131.47, 132.85, 133.00, 133.07, 138.86, 139.40, 139.64, 142.35, 146.22, 158.26, 167.40, 169.64, 172.80, 172.99, 174.95; HRMS (ESI) for C_25_H_23_Cl_3_N_6_O_4_S + H^+^, calcd. 609.0645, found 609.0642 [M + H^+^].

##### 4-((2-(2-(3-Chlorobenzylidene)hydrazine-1-carbonyl)-4-(2-(3-chlorobenzylidene)hydrazineyl)-4-oxobutyl)amino)benzenesulfonamide (**11a**)

White solid, yield 0.24 g (83%); m.p. 190–191 °C; IR (KBr) (v, cm^−1^): 3356, 3240, 3193, 1638, 1600; ^1^H NMR (400 MHz, DMSO-*d*_6_) δ (ppm): 2.59–3.64 (m, 5H, CH_2_CO, CH, NCH_2_), 6.56–8.25 (m, 17H, H_ar_, NH, NCH, NH_2_), 11.33–11.83 (m, 2H, 2CONH); ^13^C NMR (101 MHz, DMSO-*d*_6_) (δ, ppm): 32.09, 32.61, 36.16, 36.87, 43.77, 44.64, 110.74, 110.93, 125.22, 125.45, 125.57, 125.93, 126.00, 126.16, 126.93, 127.34, 127.43, 127.87, 129.41, 130.26, 130.30, 130.65, 130.88, 131.17, 133.59, 133.65, 133.72, 135.78, 136.44, 136.61, 141.19, 141.41, 141.71, 144.10, 144.56, 151.02, 151.08, 160.61, 167.18, 167.49, 169.80, 172.81, 173.08, 174.78; HRMS (ESI) C_25_H_24_Cl_2_N_6_O_4_S + H^+^, calcd. 575.1035, found 575.1033 [M + H^+^].

##### 3-Chloro-4-((2-(2-(3-chlorobenzylidene)hydrazine-1-carbonyl)-4-(2-(3-chlorobenzylidene)hydrazineyl)-4-oxobutyl)amino)benzenesulfonamide (**11b**)

Light-brown solid, yield 0.24 g (79%); m.p. 175–176 °C; IR (KBr) (v, cm^−1^): 3347, 3244, 3192, 1642, 1593; ^1^H NMR (400 MHz, DMSO-*d*_6_) δ (ppm): ^1^H NMR (400 MHz, DMSO-*d*_6_) δ (ppm): 2.61–3.75 (m, 5H, CH_2_CO, CH, NCH_2_), 6.15–6.44 (m, 1H, NH), 6.90–8.06 (m, 15H, H_ar_, NH_2_, NCH), 11.23–11.86 (m, 2H, 2CONH); ^13^C NMR (101 MHz, DMSO-*d*_6_) (δ, ppm): 31.33, 35.41, 37.52, 43.21, 44.22, 109.85, 110.24, 116.86, 117.14, 125.20, 125.55, 125.85, 126.08, 126.20, 126.28, 126.78, 126.92, 127.40, 127.86, 129.35, 130.65, 130.87, 131.04, 131.16, 131.24, 131.66, 131.79, 133.58, 133.66, 133.71, 133.87, 135.11, 135.78, 136.48, 141.21, 141.90, 146.11, 146.20, 160.22, 160.61, 172.60, 172.82, 173.00, 174.46, 174.90; HRMS (ESI) for C_25_H_23_Cl_3_N_6_O_4_S + H^+^, calcd. 609.0645, found 609.0641 [M + H^+^].

##### 4-((2-(2-(4-Chlorobenzylidene)hydrazine-1-carbonyl)-4-(2-(4-chlorobenzylidene)hydrazineyl)-4-oxobutyl)amino)benzenesulfonamide (**12a**)

Light-yellow solid, yield 0.25 g (86%); m.p. 179–180 °C; IR (KBr) (v, cm^−1^): 3401, 3243, 3073, 1663, 1600; ^1^H NMR (400 MHz, DMSO-*d*_6_) δ (ppm): 2.58–3.57 (m, 5H, CH_2_CO, CH, NCH_2_), 6.58–7.73 (m, 15H, H_ar_, NH, NH_2_), 7.86–8.04 (m, 2H, NCH), 11.14–11.76 (m, 2H, 2CONH); HRMS (ESI) C_25_H_24_Cl_2_N_6_O_4_S + H^+^, calcd. 575.1035, found 575.1030 [M + H^+^].

##### 3-Chloro-4-((2-(2-(4-chlorobenzylidene)hydrazine-1-carbonyl)-4-(2-(4-chlorobenzylidene)hydrazineyl)-4-oxobutyl)amino)benzenesulfonamide (**12b**)

White solid, yield 0.25 g (83%); m.p. 211–212 °C; IR (KBr) (v, cm^−1^): 3404, 3242, 3071, 1661, 1595; ^1^H NMR (400 MHz, DMSO-*d*_6_) δ (ppm): ^1^H NMR (400 MHz, DMSO-*d*_6_) δ (ppm): 2.62–3.66 (m, 5H, CH_2_CO, CH, NCH_2_), 6.09–6.38 (m, 1H, NH), 6.92–7.76 (m, 13H, H_ar_, NH_2_), 7.87–8.28 (m, 2H, NCH), 11.20–11.83 (m, 2H, 2CONH); ^13^C NMR (101 MHz, DMSO-*d*_6_) (δ, ppm): 32.36, 32.50, 35.19, 35.79, 44.34, 44.72, 110.01, 110.43, 116.85, 126.06, 126.85, 128.24, 128.28, 128.35, 128.54, 128.61, 128.80, 128.86, 131.19, 131.36, 133.05, 133.13, 133.18, 133.29, 134.13, 134.19, 134.26, 134.37, 141.50, 141.73, 142.07, 142.29, 144.55, 145.09, 146.25, 167.04, 167.31, 169.60, 172.72, 172.87, 174.62, 174.87; HRMS (ESI) for C_25_H_23_Cl_3_N_6_O_4_S + H^+^, calcd. 609.0645, found 609.0640 [M + H^+^].

##### 4-((2-(2-(4-Fluorobenzylidene)hydrazine-1-carbonyl)-4-(2-(4-fluorobenzylidene)hydrazineyl)-4-oxobutyl)amino)benzenesulfonamide (**13a**)

White solid, yield 0.20 g (74%); m.p. 153–154 °C; IR (KBr) (v, cm^−1^): 3297, 3214, 3071, 1639, 1601; ^1^H NMR (400 MHz, DMSO-*d*_6_) δ (ppm): 2.08–2.34 (m, 2H, CH_2_CO), 2.79–3.30 (m, 3H, CH, NCH_2_), 6.39 (t, 1H, *J =* 6.0 Hz, NH), 6.61–7.76 (m, 14H, H_ar_, NH_2_), 7.90–8.21 (m, 2H, NCH), 11.10–11.69 (m, 2H, 2CONH); HRMS (ESI) C_25_H_24_F_2_N_6_O_4_S + H^+^, calcd. 543.1626, found 543.1621 [M + H^+^].

##### 3-Chloro-4-((2-(2-(4-fluorobenzylidene)hydrazine-1-carbonyl)-4-(2-(4-fluorobenzylidene)hydrazineyl)-4-oxobutyl)amino)benzenesulfonamide (**13b**)

White solid, yield 0.23 g (79%); m.p. 207–208 °C; IR (KBr) (v, cm^−1^): 3410, 3244, 3076, 1662, 1597; ^1^H NMR (400 MHz, DMSO-*d*_6_) δ (ppm): ^1^H NMR (400 MHz, DMSO-*d*_6_) δ (ppm): 2.62–3.67 (m, 5H, CH_2_CO, CH, NCH_2_), 6.15–6.36 (m, 1H, NH), 6.90–7.80 (m, 13H, H_ar_, NH_2_), 7.88–8.26 (m, 2H, NCH), 11.24–11.70 (m, 2H, 2CONH); ^13^C NMR (101 MHz, DMSO-*d*_6_) (δ, ppm): 32.37, 33.74, 35.19, 44.35, 44.75, 110.02, 110.42, 115.72, 115.94, 116.84, 116.91, 126.06, 126.77, 126.85, 128.73, 128.81, 129.02, 129.10, 129.18, 130.89, 131.16, 141.61, 141.85, 142.16, 142.39, 144.74, 145.30, 146.27, 161.64, 161.80, 164.09, 167.25, 169.52, 172.54, 172.67, 172.83, 174.56, 174.80; HRMS (ESI) for C_25_H_23_ClF_2_N_6_O_4_S + H^+^, calcd. 577.1236, found 577.1231 [M + H^+^].

##### 4-((2-(2-(4-Methoxybenzylidene)hydrazine-1-carbonyl)-4-(2-(4-methoxybenzylidene)hydrazineyl)-4-oxobutyl)amino)benzenesulfonamide (**14a**)

White solid, yield 0.21 g (75%); m.p. 168–169 °C; IR (KBr) (v, cm^−1^): 3344, 3188, 3076, 1651, 1603; ^1^H NMR (400 MHz, DMSO-*d*_6_) δ (ppm): 2.77–3.52 (m, 5H, CH_2_CO, CH, NCH_2_), 3.79 (s, 6H, 2CH_3_), 6.58–7.67 (m, 15H, H_ar_, NH, NH_2_), 7.89–8.20 (m, 2H, NCH), 11.02–11.57 (m, 2H, 2CONH); ^13^C NMR (101 MHz, DMSO-*d*_6_) (δ, ppm): 32.24, 33.65, 35.98, 43.88, 44.39, 55.24, 55.29, 55.29, 110.86, 114.28, 126.81, 126.90, 127.29, 127.43, 128.25, 128.52, 130.17, 142.54, 143.00, 151.05, 151.12, 160.54, 160.73, 166.70, 169.83, 172.00, 172.36, 172.72, 174.41; HRMS (ESI) C_27_H_30_N_6_O_6_S + H^+^, calcd. 567.2026, found 567.2021 [M + H^+^].

##### 3-Chloro-4-((2-(2-(4-methoxybenzylidene)hydrazine-1-carbonyl)-4-(2-(4-methoxybenzylidene)hydrazineyl)-4-oxobutyl)amino)benzenesulfonamide (**14b**)

Light-yellow solid, yield 0.22 g (73%); m.p. 198–199 °C; IR (KBr) (v, cm^−1^): 3355, 3243, 3072, 1660, 1595; ^1^H NMR (400 MHz, DMSO-*d*_6_) δ (ppm): ^1^H NMR (400 MHz, DMSO-*d*_6_) δ (ppm): 2.60–3.61 (m, 5H, CH_2_CO, CH, NCH_2_), 3.79, 3.82 (2s, 6H, 2CH_3_), 6.09–6.44 (m, 1H, NH), 6.88–8.20 (m, 15H, H_ar_, NCH, NH_2_), 11.01–11.55 (m, 2H, 2CONH); ^13^C NMR (101 MHz, DMSO-*d*_6_) (δ, ppm): 32.30, 32.57, 35.22, 44.33, 55.21, 55.28, 55.39, 109.97, 114.27, 114.39, 126.11, 126.57, 126.71, 126.87, 128.24, 128.49, 128.57, 129.97, 131.12, 131.31, 142.60, 143.22, 146.28, 160.49, 160.72, 161.66, 172.42, 172.64, 174.23, 174.45; HRMS (ESI) for C_27_H_29_ClN_6_O_6_S + H^+^, calcd. 601.1636, found 601.1631 [M + H^+^].

##### 4-((2-(2-(2-Nitrobenzylidene)hydrazine-1-carbonyl)-4-(2-(2-nitrobenzylidene)hydrazineyl)-4-oxobutyl)amino)benzenesulfonamide (**15a**)

Yellow solid, yield 0.24 g (80%); m.p. 197–198 °C; IR (KBr) (v, cm^−1^): 3361, 3194, 3058, 1641, 1598; ^1^H NMR (400 MHz, DMSO-*d*_6_) δ (ppm): 2.60–3.57 (m, 5H, CH_2_CO, CH, NCH_2_), 6.59–8.22 (m, 15H, H_ar_, NH, NH_2_), 8.33–8.66 (m, 2H, NCH), 11.50–12.07 (m, 2H, 2CONH); ^13^C NMR (101 MHz, DMSO-*d*_6_) (δ, ppm): 32.38, 32.63, 33.81, 35.88, 44.03, 44.65, 110.82, 110.94, 124.62, 124.70, 124.79, 127.36, 127.58, 127.67, 127.75, 127.82, 127.91, 128.45, 128.50, 128.60, 128.74, 128.81, 129.43, 130.22, 130.34, 130.52, 132.16, 133.57, 133.69, 133.93, 138.23, 138.45, 138.60, 141.29, 141.57, 141.93, 147.97, 148.00, 148.02, 148.14, 148.88, 151.06, 151.10, 158.67, 167.37, 167.60, 169.93, 172.96, 173.16, 174.96, 175.15; HRMS (ESI) C_25_H_24_N_8_O_8_S + H^+^, calcd. 597.1516, found 597.1513 [M + H^+^].

##### 3-Chloro-4-((2-(2-(2-nitrobenzylidene)hydrazine-1-carbonyl)-4-(2-(2-nitrobenzylidene)hydrazineyl)-4-oxobutyl)amino)benzenesulfonamide (**15b**)

Yellow solid, yield 0.29 g (94%); m.p. 178–179 °C; IR (KBr) (v, cm^−1^): 3358, 3243, 3073, 1646, 1591; ^1^H NMR (400 MHz, DMSO-*d*_6_) δ (ppm): ^1^H NMR (400 MHz, DMSO-*d*_6_) δ (ppm): 2.68–3.69 (m, 5H, CH_2_CO, CH, NCH_2_), 6.17–6.39 (m, 1H, NH), 6.92–7.76 (m, 13H, H_ar_, NH_2_), 8.32–8.68 (m, 2H, NCH), 11.59–12.03 (m, 2H, 2CONH); ^13^C NMR (101 MHz, DMSO-*d*_6_) (δ, ppm): 32.38, 33.78, 35.26, 35.83, 44.31, 44.70, 110.07, 116.84, 116.95, 124.62, 124.69, 124.78, 126.03, 126.80, 127.43, 127.82, 127.88, 128.45, 128.48, 129.43, 130.35, 131.17, 131.40, 132.16, 133.53, 133.71, 133.92, 138.27, 138.67, 138.98, 146.23, 147.96, 148.00, 148.14, 148.88, 158.67, 167.58, 169.84, 172.95, 173.11, 174.90, 175.16; HRMS (ESI) for C_25_H_23_ClN_8_O_8_S + H^+^, calcd. 631.1126, found 631.1121 [M + H^+^].

##### 4-((2-(2-(3-Nitrobenzylidene)hydrazine-1-carbonyl)-4-(2-(3-nitrobenzylidene)hydrazineyl)-4-oxobutyl)amino)benzenesulfonamide (**16a**)

Light-yellow solid, yield 0.26 g (87%); m.p. 218–219 °C; IR (KBr) (v, cm^−1^): 3352, 3260, 3074, 1667, 1595; ^1^H NMR (400 MHz, DMSO-*d*_6_) δ (ppm): 2.61–3.58 (m, 5H, CH_2_CO, CH, NCH_2_), 6.58–8.59 (m, 17H, H_ar_, NH, NCH, NH_2_), 11.29–11.99 (m, 2H, 2CONH); ^13^C NMR (101 MHz, DMSO-*d*_6_) (δ, ppm): 32.40, 32.63, 34.03, 34.30, 36.10, 36.78, 36.82, 43.96, 44.62, 110.73, 110.95, 120.89, 121.06, 121.34, 122.66, 124.00, 125.83, 127.34, 130.17, 130.37, 130.66, 132.10, 132.68, 133.18, 133.25, 134.44, 135.24, 136.03, 136.23, 140.64, 140.85, 143.84, 148.16, 148.21, 151.11, 160.50, 167.36, 167.60, 169.76, 169.98, 172.88, 173.10, 174.85, 175.09; HRMS (ESI) C_25_H_24_N_8_O_8_S + H^+^, calcd. 597.1516, found 597.1514 [M + H^+^].

##### 3-Chloro-4-((2-(2-(3-nitrobenzylidene)hydrazine-1-carbonyl)-4-(2-(3-nitrobenzylidene)hydrazineyl)-4-oxobutyl)amino)benzenesulfonamide (**16b**)

White solid, yield 0.28 g (90%); m.p. 199–200 °C; IR (KBr) (v, cm^−1^): 3368, 3242, 3071, 1659, 1598; ^1^H NMR (400 MHz, DMSO-*d*_6_) δ (ppm): ^1^H NMR (400 MHz, DMSO-*d*_6_) δ (ppm): 2.66–3.69 (m, 5H, CH_2_CO, CH, NCH_2_), 6.14–6.33 (m, 1H, NH), 6.83–8.61 (m, 15H, H_ar_, NCH, NH_2_), 11.48–12.02 (m, 2H, 2CONH); ^13^C NMR (101 MHz, DMSO-*d*_6_) (δ, ppm): 32.44, 35.31, 35.92, 44.33, 44.66, 109.93, 116.76, 116.97, 120.83, 120.93, 121.23, 121.37, 123.93, 124.14, 126.07, 126.76, 127.35, 130.11, 130.36, 131.11, 131.40, 131.47, 132.12, 132.72, 133.20, 135.90, 136.06, 136.18, 140.69, 141.00, 143.44, 143.73, 144.01, 146.24, 148.08, 148.17, 167.35, 167.57, 169.92, 172.89, 173.03, 174.93, 175.21; HRMS (ESI) for C_25_H_23_ClN_8_O_8_S + H^+^, calcd. 631.1126, found 631.1122 [M + H^+^].

##### 4-((2-(2-(4-Nitrobenzylidene)hydrazine-1-carbonyl)-4-(2-(4-nitrobenzylidene)hydrazineyl)-4-oxobutyl)amino)benzenesulfonamide (**17a**)

Yellow solid, yield 0.28 g (93%); m.p. 256–257 °C; IR (KBr) (v, cm^−1^): 3413, 3239, 3066, 1665, 1601; ^1^H NMR (400 MHz, DMSO-*d*_6_) δ (ppm): 2.60–3.60 (m, 5H, CH_2_CO, CH, NCH_2_), 6.56–8.36 (m, 17H, H_ar_, NH, NCH, NH_2_), 11.51–12.00 (m, 2H, 2CONH); HRMS (ESI) C_25_H_24_N_8_O_8_S + H^+^, calcd. 597.1516, found 597.1511 [M + H^+^].

##### 3-Chloro-4-((2-(2-(4-nitrobenzylidene)hydrazine-1-carbonyl)-4-(2-(4-nitrobenzylidene)hydrazineyl)-4-oxobutyl)amino)benzenesulfonamide (**17b**)

Yellow solid, yield 0.25 g (81%); m.p. 220–221 °C; IR (KBr) (v, cm^−1^): 3390, 3247, 3072, 1662, 1596; ^1^H NMR (400 MHz, DMSO-*d*_6_) δ (ppm): ^1^H NMR (400 MHz, DMSO-*d*_6_) δ (ppm): 2.66–3.67 (m, 5H, CH_2_CO, CH, NCH_2_), 6.17–6.37 (m, 1H, NH), 6.92–8.37 (m, 15H, H_ar_, NCH, NH_2_), 11.45–12.14 (m, 2H, 2CONH); ^13^C NMR (101 MHz, DMSO-*d*_6_) (δ, ppm): 32.49, 35.15, 35.75, 44.38, 44.65, 110.01, 110.45, 116.80, 116.96, 123.87, 124.01, 124.01, 126.06, 126.77, 127.34, 127.57, 127.64, 127.81, 127.90, 131.18, 140.32, 140.52, 140.57, 140.88, 141.11, 143.44, 143.97, 146.24, 147.59, 147.77, 167.64, 173.04, 173.16, 175.37; HRMS (ESI) for C_25_H_23_ClN_8_O_8_S + H^+^, calcd. 631.1126, found 631.1121 [M + H^+^].

##### 4,4′-(((2-(((4-Sulfamoylphenyl)amino)methyl)succinyl)bis(hydrazin-2-yl-1-ylidene))bis(methaneylylidene))dibenzoic acid (**18a**)

Light-yellow solid, yield 0.27 g (91%); m.p. 308–309 °C; IR (KBr) (v, cm^−1^): 3319, 3191, 3074, 1667, 1626, 1601; ^1^H NMR (400 MHz, DMSO-*d*_6_) δ (ppm): ^1^H NMR (400 MHz, DMSO-*d*_6_) δ (ppm): 2.70–3.54 (m, 5H, CH_2_CO, CH, NCH_2_), 6.45–8.51 (m, 17H, H_ar_, NH, NCH, NH_2_), 11.32–11.94 (m, 2H, 2CONH), 13.03 (br. s, 2H, 2OH); ^13^C NMR (101 MHz, DMSO-*d*_6_) (δ, ppm): 32.76, 35.07, 35.75, 49.98, 118.91, 126.49, 126.71, 126.98, 127.13, 127.39, 128.53, 129.55, 129.78, 129.82, 130.32, 131.64, 133.15, 137.48, 138.23, 139.01, 141.87, 142.73, 161.01, 166.86, 166.94, 172.79, 173.65; HRMS (ESI) C_27_H_26_N_6_O_8_S + H^+^, calcd. 595.1611, found 595.1606 [M + H^+^].

##### 4,4′-(((2-(((2-Chloro-4-sulfamoylphenyl)amino)methyl)succinyl)bis(hydrazin-2-yl-1-ylidene))bis(methaneylylidene))dibenzoic acid (**18b**)

Light-brown solid, yield 0.28 g (90%); m.p. 294–295 °C; IR (KBr) (v, cm^−1^): 3348, 3245, 3194, 3064, 1671, 1643, 1597; ^1^H NMR (400 MHz, DMSO-*d*_6_) δ (ppm): ^1^H NMR (400 MHz, DMSO-*d*_6_) δ (ppm): 2.65–3.73 (m, 5H, CH_2_CO, CH, NCH_2_), 6.13–6.40 (m, 1H, NH), 6.84–8.33 (m, 15H, H_ar_, NCH, NH_2_), 11.36–11.87 (m, 2H, 2CONH), 13.09 (br. s, 2H, 2OH); HRMS (ESI) for C_27_H_25_ClN_6_O_8_S + H^+^, calcd. 629.1221, found 629.1216 [M + H^+^].

#### 3.1.6. General Procedure for the Synthesis of Pyrroles **19a,b**

A mixture of hydrazide **2a** (0.46 g, 1.4 mmol) or **2b** (0.51 g, 1.4 mmol), hexane-2,5-dione (0.8 mL, 7 mmol), and 30 mL of propan-2-ol was heated under reflux for 5h. Then, the reaction mixture was filtered, and the liquid fractions were evaporated under reduced pressure. The residue was diluted with water (20 mL), and the precipitate was filtered off, washed with water, dried, and recrystallized from propan-2-ol.

##### N^1^,N^4^-bis(2,5-Dimethyl-1*H*-pyrrol-1-yl)-2-(((4-sulfamoylphenyl)amino)methyl)succinamide (**19a**)

Light-brown solid, yield 0.51 g (75%); m.p. 195–196 °C; IR (KBr) (v, cm^−1^): 3399, 3362, 3248, 1667, 1598; ^1^H NMR (400 MHz, DMSO-*d*_6_) δ (ppm): 1.86–2.02 (m, 12H, 4CH_3_), 2.55–2.82 (m, 2H, CH_2_), 3.15–3.31 (m, 2H, NCH_2_), 3.39–3.56 (m, 1H, CH), 5.60, 5.63 (2s, 4H, 4CH_pyr_), 6.61–6.77 (m, 3H, H_ar_, NH), 6.95 (br. s, 2H, NH_2_), 7.48–7.57 (m, 2H, H_ar_), 10.70, 10.81 (2s, 2H, 2NNH); ^13^C NMR (101 MHz, DMSO-*d*_6_) (δ, ppm): 10.88, 10.92, 10.94, 11.04, 32.83, 44.55, 102.79, 102.89, 102.95, 110.88, 126.62, 126.85, 126.99, 130.56, 150.86, 169.93, 172.10; HRMS (ESI) for C_23_H_30_N_6_O_4_S + H^+^, calcd. 487.2127, found 487.2122 [M + H^+^].

##### 2-(((2-Chloro-4-sulfamoylphenyl)amino)methyl)-N^1^,N^4^-bis(2,5-dimethyl-1*H*-pyrrol-1-yl)succinamide (**19b**)

Light-brown solid, yield 0.59 g (81%); m.p. 232–233 °C; IR (KBr) (v, cm^−1^): 3401, 3356, 3244, 1660, 1594; ^1^H NMR (400 MHz, DMSO-*d*_6_) δ (ppm): 1.91, 1.94, 1.98 (3s, 12H, 4CH_3_), 2.58–2.84 (m, 2H, CH_2_), 3.36–3.43 (m, 2H, NCH_2_), 3.58–3.68 (m, 1H, CH), 5.61, 5.63 (2s, 4H, 4CH_pyr_), 6.24 (t, 1H, *J =* 5.5 Hz, NH), 7.00 (d, *J =* 8.6 Hz, 1H, H_ar_), 7.16 (br. s, 2H, NH_2_), 7.52–7.71 (m, 2H, H_ar_), 10.72, 10.87 (2s, 2H, 2NNH); ^13^C NMR (101 MHz, DMSO-*d*_6_) (δ, ppm): 10.89, 10.93, 11.00, 11.04, 32.76, 44.69, 102.84, 102.90, 102.96, 110.33, 117.03, 126.15, 126.63, 126.79, 126.84, 126.97, 131.62, 146.12, 169.91, 172.04; HRMS (ESI) for C_23_H_29_ClN_6_O_4_S + H^+^, calcd. 521.1738, found 521.1732 [M + H^+^].

#### 3.1.7. 3-Chloro-4-((4-(3,5-dimethyl-1*H*-pyrazol-1-yl)-2-(3,5-dimethyl-1*H*-pyrazole-1-carbonyl)-4-oxobutyl)amino)benzenesulfonamide (**20**)

A mixture of hydrazide **2b** (0.51 g, 1.4 mmol), pentane-2,4-dione (0.6 mL, 6 mmol) and 20 mL of propan-2-ol was heated at reflux for 5 h. Then it was cooled down and the precipitate was filtered off, washed with diethyl ether and recrystallized from propan-2-ol to afford light-brown solid, yield 0.54 g (78%); m.p. 201–202 °C; IR (KBr) (v, cm^−1^): 3371, 1723, 1595; ^1^H NMR (400 MHz, DMSO-*d*_6_) δ (ppm): 2.18, 2.25, 2.35, 2.43 (4s, 12H, 4CH_3_), 3.34–3.44 (m, 3H, CH, CH_2_), 3.61–3.75 (m, 2H, NCH_2_), 6.16, 6.24 (2s, 2H, 2CH_pyr_), 6.52 (t, 1H, *J =* 5.9 Hz, NH), 7.13 (br. s, 2H, NH_2_), 7.25 (d, 1H, *J =* 8.7 Hz, H_ar_), 7.56–7.65 (m, 2H, H_ar_); ^13^C NMR (101 MHz, DMSO-*d*_6_) (δ, ppm): 13.56, 13.70, 14.07, 14.20, 35.39, 44.32, 110.76, 111.40, 111.71, 116.99, 126.04, 126.85, 131.50, 143.51, 143.81, 146.19, 151.93, 152.24, 172.07, 173.76; HRMS (ESI) for C_21_H_25_ClN_6_O_4_S + H^+^, calcd. 493.1425, found 493.1419 [M + H^+^].

#### 3.1.8. General Procedure for the Synthesis of Compounds **22a,b** and **23a,b**

Hydrazide **2a** (1 g, 3 mmol) or **2b** (1.10 g, 3 mmol) was dissolved in dimethylformamide (5 mL), and methyl isothiocyanate (0.58 g, 8 mmol) or phenyl isothiocyanate (0.9 mL, 7.5 mmol) was added dropwise, respectively. Reaction mixtures were stirred at room temperature for 4–6 h and then diluted with water (20 mL). The precipitate was filtered off, washed with water, dried, and recrystallized from 1,4-dioxane.

##### 2,2′-(2-(((4-Sulfamoylphenyl)amino)methyl)succinyl)bis(*N*-methylhydrazine-1-carbothioamide) (**22a**)

White solid, yield 1.13 g (79%); m.p. 192–193 °C; IR (KBr) (v, cm^−1^): 3381, 3299, 3196, 1657, 1601; ^1^H NMR (400 MHz, DMSO-*d_6_*) δ (ppm): 2.81–2.89 (m, 6H, 2CH_3_), 2.91–3.32 (m, 5H, CH, NCH_2_, CH_2_), 6.44 (t, 1H, *J =* 6.0 Hz, ArNH), 6.67 (d, 2H, *J =* 8.5 Hz, H_ar_,), 6.94 (br. s, 2H, NH_2_), 7.52 (d, 2H, *J =* 8.5 Hz, H_ar_,), 7.62–7.97 (m, 2H, 2NH), 9.19 (br. s, 2H, 2NH), 9.91, 10.05 (2s, 2H, 2NH); ^13^C NMR (101 MHz, DMSO-*d_6_*) (δ, ppm): 30.87, 33.44, 35.83, 44.32, 111.07, 127.37, 130.64, 150.98, 171.19, 172.71, 182.17; HRMS (ESI) for C_15_H_24_N_8_O_4_S_3_ + H^+^, calcd. 477.1161, found 477.1155 [M + H^+^].

##### 2,2′-(2-(((2-Chloro-4-sulfamoylphenyl)amino)methyl)succinyl)bis(*N*-methylhydrazine-1-carbothioamide (**22b**)

Light-brown solid, yield 1.19 g (78%); m.p. 212–213 °C; IR (KBr) (v, cm^−1^): 3386, 3300, 3175, 1660, 1594; ^1^H NMR (400 MHz, DMSO-*d*_6_) δ (ppm): 2.84, 2.85 (2s, 6H, 2CH_3_), 2.93–3.51 (m, 5H, CH, CH_2_, NCH_2_), 6.17 (t, 1H, *J =* 5.8 Hz, ArNH), 6.90 (d, 1H, *J =* 8.8 Hz, H_ar_), 7.14 (br. s, 2H, NH_2_), 7.57 (d, 1H, *J =* 8.6 Hz, H_ar_), 7.67–7.97 (m, 2H, 2NH), 9.09–9.38 (m, 2H, 2NH), 9.94, 10.10 (2s, 2H, NH); ^13^C NMR (101 MHz, DMSO-*d*_6_) (δ, ppm): 30.88, 33.22, 44.24, 110.38, 117.05, 126.13, 126.77, 131.50, 146.24, 171.29, 172.69, 182.04, 182.17, 182.26; HRMS (ESI) for C_15_H_23_ClN_8_O_4_S_3_+H^+^, calcd. 511.0771, found 511.0773 [M + H^+^].

##### 2,2′-(2-(((4-Sulfamoylphenyl)amino)methyl)succinyl)bis(*N*-phenylhydrazine-1-carbothioamide) (**23a**)

White solid, yield 1.64 g (91%); m.p. 136–137 °C; IR (KBr) (v, cm^−1^): 3212, 1660, 1597; ^1^H NMR (400 MHz, DMSO-*d_6_*) δ (ppm): 2.54–3.27 (m, 5H, NCH_2_, CH, CH_2_), 6.35–7.85 (m, 17H, NH, NH_2_, H_ar_), 9.32–10.50 (m, 6H, 6NH); ^13^C NMR (101 MHz, DMSO-*d_6_*) (δ, ppm): 30.80, 33.35, 35.81, 44.28, 111.09, 116.79, 121.03, 123.66, 123.98, 124.45, 125.96, 127.38, 128.14, 128.45, 128.98, 129.93, 130.66, 139.00, 139.03, 141.19, 150.94, 162.32; HRMS (ESI) for C_25_H_28_N_8_O_4_S_3_ + H^+^, calcd. 601.1474, found 601.1468 [M + H^+^].

##### 2,2′-(2-(((2-Chloro-4-sulfamoylphenyl)amino)methyl)succinyl)bis(*N*-phenylhydrazine-1-carbothioamide) (**23b**)

White solid, yield 1.71 g (90%); m.p. 158–159 °C; IR (KBr) (v, cm^−1^): 3244, 1683, 1593; ^1^H NMR (400 MHz, DMSO-*d_6_*) δ (ppm): 2.40–4.00 (m, 5H, CH_2_, CH, NCH_2_), 6.17 (br. s, 1H, NH), 6.83–7.87 (m, 15H, NH_2_, H_ar_), 9.38–10.55 (m, 6H, 6NH); ^13^C NMR (101 MHz, DMSO-*d_6_*) (δ, ppm): 30.79, 30.97, 33.17, 35.80, 44.17, 110.34, 116.80, 117.05, 123.82, 124.40, 126.13, 126.24, 126.77, 128.11, 128.51, 131.09, 131.53, 139.02, 146.19, 158.23, 162.31; HRMS (ESI) for C_25_H_27_ClN_8_O_4_S_3_ + H^+^, calcd. 635.1084, found 635.1079 [M + H^+^].

#### 3.1.9. General Procedure for the Synthesis of Compounds **24a,b** and **25a,b**

A mixture of thiosemicarbazide **22a**,**b** (0.5 mmol) or **23a**,**b** (0.5 mmol) and 2% aqueous KOH solution (20 mL) was stirred at room temperature for 8 h. Afterwards, it was acidified with glacial acetic acid to pH 6. The formed precipitate was filtered off, washed with water, and recrystallized from propan-2-ol.

##### 4-((2,3-bis(4-Methyl-5-thioxo-4,5-dihydro-1*H*-1,2,4-triazol-3-yl)propyl)amino)benzenesulfonamide (**24a**)

Brown solid, yield 0.16 g (73%); m.p. 170–171 °C; IR (KBr) (v, cm^−1^): 3332, 3215, 3087; ^1^H NMR (400 MHz, DMSO-*d_6_*) δ (ppm): 3.13–3.23 (m, 2H, CH_2_), 3.33, 3.39 (2s, 6H, 2CH_3_), 3.49–3.61 (m, 2H, NCH_2_), 3.61–3.71 (m, 1H, CH), 6.57–6.73 (m, 3H, ArNH, H_ar_), 6.95 (br. s, 2H, NH_2_), 7.52 (d, *J =* 8.4 Hz, 2H, H_ar_), 13.52, 13.64 (2s, 2H, NH); ^13^C NMR (101 MHz, DMSO-*d_6_*) (δ, ppm): 26.50, 29.73, 29.99, 32.58, 45.84, 110.92, 127.52, 130.77, 150.71, 150.84, 153.36, 166.49, 166.74; HRMS (ESI) for C_15_H_20_N_8_O_2_S_3_ + H^+^, calcd. 441.0950, found 441.0944 [M + H^+^].

##### 4-((2,3-bis(4-Methyl-5-thioxo-4,5-dihydro-1*H*-1,2,4-triazol-3-yl)propyl)amino)-3-chlorobenzenesulfonamide (**24b**)

Light-brown solid, yield 0.17 g (71%); m.p. 151–152 °C; IR (KBr) (v, cm^−1^): 3308, 3241, 3078; ^1^H NMR (400 MHz, DMSO-*d_6_*) δ (ppm): 3.15–3.22 (m, 2H, CH_2_), 3.36, 3.40 (2s, 6H, 2CH_3_), 3.57–3.67 (m, 2H, NCH_2_), 3.71–3.83 (m, 1H, CH), 6.39 (t, 1H, *J =* 6.1 Hz, ArNH), 6.95 (d, *J =* 8.7 Hz, 1H, H_ar_), 7.13 (br. s, 2H, NH_2_), 7.54 (d, *J =* 8.7 Hz, 1H, H_ar_), 7.65 (s, 1H, H_ar_), 13.51, 13.63 (2s, 2H, NH); ^13^C NMR (101 MHz, DMSO-*d_6_*) (δ, ppm): 26.32, 29.73, 30.00, 32.36, 45.69, 110.20, 116.94, 126.17, 126.98, 131.61, 146.13, 150.68, 153.15, 166.52, 166.74; HRMS (ESI) for C_15_H_19_ClN_8_O_2_S_3_+H^+^, calcd. 475.0560, found 475.0557 [M + H^+^].

##### 4-((2,3-bis(4-Phenyl-5-thioxo-4,5-dihydro-1*H*-1,2,4-triazol-3-yl)propyl)amino)benzenesulfonamide (**25a**)

White solid, yield 0,19 g (68%); m.p. 258–259 °C; IR (KBr) (v, cm^−1^): 3381, 3201, 3074; ^1^H NMR (400 MHz, DMSO-*d_6_*) δ (ppm): 2.79–2.87 (m, 2H, CH_2_), 3.03–3.21 (m, 2H, NCH_2_), 3.25–3.38 (m, 1H, CH), 6.09 (d, *J =* 8.4 Hz, 2H, H_ar_), 6.59 (t, 1H, *J =* 6.2 Hz, ArNH), 6.86–7.68 (m, 14H, NH_2_, H_ar_), 13.44 (br. s, 2H, 2NH); ^13^C NMR (101 MHz, DMSO-*d_6_*) (δ, ppm): 26.94, 32.43, 45.31, 110.49, 127.20, 127.94, 129.42, 129.56, 130.63, 133.14, 133.17, 149.96, 149.98, 152.45, 167.62, 167.76; HRMS (ESI) for C_25_H_24_N_8_O_2_S_3_+H^+^, calcd. 565.1263, found 565.1257 [M + H^+^].

##### 4-((2,3-bis(4-Phenyl-5-thioxo-4,5-dihydro-1*H*-1,2,4-triazol-3-yl)propyl)amino)-3-chlorobenzenesulfonamide (**25b**)

White solid, yield 0,22 g (73%); m.p. 175–176 °C; IR (KBr) (v, cm^−1^): 3351, 3192, 3065; ^1^H NMR (400 MHz, DMSO-*d_6_*) δ (ppm): 2.76–3.79 (m, 5H, CH_2,_ CH, NCH_2,_), 5.90 (d, *J =* 8.8 Hz, H, H_ar_), 6.34 (t, 1H, *J =* 6.2 Hz, ArNH), 6.92–7.75 (m, 15H, NH_2_, H_ar_), 12.82 (br. s, 2H, 2NH); ^13^C NMR (101 MHz, DMSO-*d_6_*) (δ, ppm): 26.98, 31.92, 45.22, 109.03, 116.84, 117.01, 125.60, 126.86, 127.77, 128.07, 129.06, 129.23, 129.44, 129.49, 131.39, 133.04, 133.27, 145.15, 150.01, 152.32, 167.52, 167.78; HRMS (ESI) for C_25_H_23_ClN_8_O_2_S_3_+H^+^, calcd. 599.0873, found 599.0867 [M + H^+^].

#### 3.1.10. 3-((4-Methylthiazol-2-yl)(4-sulfamoylphenyl)amino)propanoic acid (**27**)

Carboxylic acid 26 (0.45 g, 1.5 mmol) and NaOAc (0.20 g, 2.5 mmol) were dissolved into 10 mL of hot water. Then, chloroacetone (0.20 mL, 2.5 mmol) was added, and reaction mixture was heated at reflux for 4h. Then, it was cooled down, and the precipitate was filtered off and washed with diethyl ether and n-hexane to afford light-pinkish solid, yield 0.50 g (98%); m.p. 162–163 °C; IR (KBr) (v, cm^−1^): 3300, 1680, 1497; ^1^H NMR (400 MHz, DMSO-*d_6_*) δ (ppm): 2.19 (s, 3H, CH_3_), 2.63 (t, 2H, *J =* 7.2 Hz, CH_2_), 4.14 (t, 2H, *J =* 7.3 Hz, CH_2_), 6.44 (s, 1H, CH), 7.39 (s, 2H, NH_2_), 7.62 (d, 2H, *J =* 8.3 Hz, H_ar_), 7.85 (d, 2H, *J =* 8.3 Hz, H_ar_), 12.36 (br. s, 1H, OH); ^13^C NMR (101 MHz, DMSO-*d_6_*) (δ, ppm): 17.39, 32.25, 48.53, 103.51, 125.15, 127.33, 140.99, 147.39, 148.45, 167.23, 172.48; Anal. Calcd. for C_13_H_15_N_3_O_4_S_2_: C 45.74; H 4.43; N 12.31%. Found: C 45.82; H 4.48; N 12.30%.

#### 3.1.11. Methyl 3-((4-Methylthiazol-2-yl)(4-sulfamoylphenyl)amino)propanoate (**28**)

A mixture of thiazole 27 (0.41 g, 1.2 mmol), H_2_SO_4_ (0.1 mL), and 15 mL of methanol were heated under reflux for 6 h. Then, the reaction mixture was filtered, and the liquid fractions were evaporated under reduced pressure. The residue was diluted with 5% aqueous Na_2_CO_3_ solution (20 mL), and the precipitate was filtered off, washed with water, dried, and recrystallized from propan-2-ol to afford white solid, yield 0,32 g (74%); m.p. 72–73 °C; IR (KBr) (v, cm^−1^): 1679, 1507; ^1^H NMR (400 MHz, DMSO-*d_6_*) δ (ppm): 2.19 (s, 3H, CH_3_), 2.72 (t, 2H, *J =* 7.0 Hz, CH_2_), 3.51 (s, 3H, CH_3_), 4.18 (t, 2H, *J =* 7.0 Hz, CH_2_), 6.43 (s, 1H, CH), 7.40 (s, 2H, NH_2_), 7.61 (d, 2H, *J =* 8.3 Hz, H_ar_), 7.85 (d, 2H, *J =* 8.3 Hz, H_ar_); ^13^C NMR (101 MHz, DMSO-*d_6_*) (δ, ppm): 17.37, 32.12, 48.48, 51.43, 103.52, 125.35, 127.36, 141.17, 147.31, 148.44, 167.22, 171.39; Anal. Calcd. for C_14_H_17_N_3_O_4_S_2_: C 47.31; H 4.82; N 11.82%. Found: C 47.56; H 4.83; N 11.81%.

#### 3.1.12. 4-((3-Hydrazineyl-3-oxopropyl)(4-methylthiazol-2-yl)amino)benzenesulfonamide (**29**)

Ester 28 (0.53 g, 1.5 mmol) and hydrazine monohydrate (0.15 g, 3 mmol) were dissolved into 5 mL of propan-2-ol, and the reaction mixture was heated under reflux for 5 h. Then, it was cooled down, and the precipitate was filtered off, washed with diethyl ether, and n-hexane to afford white solid, yield 0.47 g (89%); m.p. 167–168 °C; IR (KBr) (v, cm^−1^): 3382, 3260, 1644, 1498; ^1^H NMR (400 MHz, DMSO-*d_6_*) δ (ppm): 2.19 (s, 3H, CH_3_), 2.44 (t, 2H, *J =* 7.3 Hz, CH_2_), 3.92–4.72 (m, 4H, NH_2_, CH_2_), 6.45 (s, 1H, CH), 7.44–7.96 (m, 6H, NH_2_, H_ar_), 9.06 (s, 1H, NH); ^13^C NMR (101 MHz, DMSO-*d_6_*) (δ, ppm): 17.40, 31.77, 49.21, 103.58, 124.93, 127.22, 127.26, 140.77, 147.41, 148.43, 167.18, 169.14; Anal. Calcd. for C_13_H_17_N_5_O_3_S_2_: C 43.93; H 4.82; N 19.70%. Found: C 44.04; H 4.75; N 19.50%.

#### 3.1.13. 4-((3-(2-Benzylidenehydrazineyl)-3-oxopropyl)(4-methylthiazol-2-yl)amino)benzenesulfonamide (**30**)

Hydrazide 29 (0.53 g, 1.5 mmol), benzaldehyde (0.19 g, 1.8 mmol), and 10 mL of propan-2-ol were mixed under reflux for 1 h. Then, the reaction mixture was cooled down, and the precipitate was filtered off, washed with diethyl ether, and recrystallized from propan-2-ol to afford white solid, yield 0.58 g (87%); m.p. 170–171 °C; IR (KBr) (v, cm^−1^): 3247, 1671, 1595, 1499; ^1^H NMR (400 MHz, DMSO-*d_6_*) δ (ppm): 2.08 (s, 3H, CH_3_), 2.58 (t, 0.70H, *J =* 7.2 Hz, CH_2_), 3.00 (t, 1.30H, *J =* 7.4 Hz, CH_2_) 4.06–4.36 (m, 2H, NCH_2_), 7.13–8.14 (m, 13H, NH_2_, CH, N=CH, H_Ar_), 11.35, 11.38, 11.41, 11.44 (4s, 1H, NH); Anal. Calcd. for C_20_H_21_N_5_O_3_S_2_: C 54.16; H 4.77; N 15.79%. Found: C 54.36; H 4.65; N 15.90%.

#### 3.1.14. 3-((5-(Ethoxycarbonyl)-4-methylthiazol-2-yl)(4-sulfamoylphenyl)amino)propanoic acid (**31**)

Carboxylic acid 26 (1.00 g, 3 mmol) and NaOAc (0.30 g, 3.6 mmol) were dissolved into 10 mL of hot water. Then, ethyl 2-chloroacetoacetate (0.70 g, 4.25 mmol) was added, and the reaction mixture was heated at reflux for 12h. Then, it was cooled down, and the precipitate was filtered off and washed with water and n-hexane to afford white solid, yield 1.05 g (85%); m.p. 221–222 °C; IR (KBr) (v, cm^−1^): 3546, 1708, 1677, 1509; ^1^H NMR (400 MHz, DMSO-*d_6_*) δ (ppm): 1.20 (t, 3H, *J =* 7.1 Hz, CH_3_), 2.52 (s, 3H, CH_3_), 2.65 (t, 2H, *J =* 7.1 Hz, CH_2_), 4.08–4.28 (m, 4H, 2CH_2_), 7.48 (s, 2H, NH_2_), 7.70 (d, 2H, *J =* 8.4 Hz, H_ar_), 7.95 (d, 2H, *J =* 8.4 Hz, H_ar_), 12.38 (s, 1H, OH); ^13^C NMR (101 MHz, DMSO-*d_6_*) (δ, ppm): 14.23, 17.31, 32.14, 48.34, 60.22, 109.47, 127.19, 127.69, 143.05, 146.02, 159.13, 161.56, 169.25, 172.23; Anal. Calcd. for C_16_H_19_N_3_O_6_S_2_: C 46.48; H 4.63; N 10.16%. Found: C 46.29; H 4.56; N 10.10%.

#### 3.1.15. Ethyl 2-((3-Methoxy-3-oxopropyl)(4-sulfamoylphenyl)amino)-4-methylthiazole-5-carboxylate (**32**)

A mixture of thiazole 31 (0.42 g, 1 mmol), H_2_SO_4_ (0.1 mL) and 15 mL of methanol were heated under reflux for 5 h. Then, the reaction mixture was filtered, and the liquid fractions were evaporated under reduced pressure. The residue was diluted with 5% aqueous Na_2_CO_3_ solution (20 mL); the precipitate was filtered off, washed with water, dried, and recrystallized from propan-2-ol to afford white solid, yield 0.32 g (74%); m.p. 140–141 °C; IR (KBr) (v, cm^−1^): 3371, 1732, 1678, 1520; ^1^H NMR (400 MHz, DMSO-*d_6_*) δ (ppm): 1.19 (t, 3H, *J =* 7.1 Hz, CH_3_), 2.48 (s, 3H, CH_3_), 2.72 (t, 2H, *J =* 6.9 Hz, CH_2_), 3.51 (s, 3H, CH_3_), 4.13 (q, 2H, *J =* 7.1 Hz, CH_2_), 4.21 (t, 2H, *J =* 6.9 Hz, CH_2_), 7.47 (s, 2H, NH_2_), 7.68 (d, 2H, *J =* 8.3 Hz, H_ar_), 7.94 (d, 2H, *J =* 8.2 Hz, H_ar_); ^13^C NMR (101 MHz, DMSO-*d_6_*) (δ, ppm): 14.21, 17.28, 32.01, 48.28, 51.48, 60.22, 109.54, 127.24, 127.70, 143.14, 145.94, 159.08, 161.53, 169.21, 171.15; Anal. Calcd. for C_17_H_21_N_3_O_6_S_2_: C 47.76; H 4.95; N 9.83%. Found: C 47.77; H 4.94; N 9.99%.

#### 3.1.16. Ethyl 2-((3-(Hydrazineyloxy)-3-oxopropyl)(4-sulfamoylphenyl)amino)-4-methylthiazole-5-carboxylate (**33**)

A mixture of ester 32 (0.43 g, 1 mmol), hydrazine monohydrate (0.15 g, 3 mmol), and 15 mL of propan-2-ol were heated under reflux for 12 h. Then, the reaction mixture was cooled down, and the precipitate was filtered off and recrystallized from propan-2-ol to afford white solid, yield 0.38 g (88%); m.p. 206–207 °C; IR (KBr) (v, cm^−1^): 3325, 3223, 1712, 1679, 1507; ^1^H NMR (400 MHz, DMSO-*d_6_*) δ (ppm): 1.19 (t, 3H, *J =* 7.1 Hz, CH_3_), 2.43 (t, 2H, *J =* 7.1 Hz, CH_2_), 2.48 (s, 3H, CH_3_), 4.06–4.24 (m, 6H, NH_2_, 2CH_2_), 7.47 (s, 2H, SO_2_NH_2_), 7.67 (d, 2H, *J =* 8.1 Hz, H_ar_), 7.93 (d, 2H, *J =* 8.2 Hz, H_ar_), 9.07 (s, 1H, NH); ^13^C NMR (101 MHz, DMSO-*d_6_*) (δ, ppm): 14.25, 17.33, 31.71, 49.20, 60.21, 109.45, 127.14, 127.63, 142.92, 146.11, 159.17, 161.58, 168.86, 169.21; Anal. Calcd. for C_16_H_21_N_5_O_5_S_2_: C 44.95; H 4.95; N 16.38%. Found: C 45.20; H 4.90; N 16.23%.

#### 3.1.17. Ethyl 2-((3-(2-Benzylidenehydrazineyl)-3-oxopropyl)(4-sulfamoylphenyl)amino)-4-methylthiazole-5-carboxylate (**34**)

A mixture of hydrazide 33 (0.43 g, 1 mmol), benzaldehyde (0.13 g, 1.2 mmol), and 15 mL of propan-2-ol were heated under reflux for 3 h. Then, the reaction mixture was cooled down and diluted with diethyl ether. The precipitate was filtered off to afford white solid, yield 0.44 g (85%); m.p. 195–196 °C; IR (KBr) (v, cm^−1^): 3269, 1678, 1655, 1553 1513; ^1^H NMR (400 MHz, DMSO-*d_6_*) δ (ppm): 1.11–1.27 (m, 3H, CH_3_), 2.51 (s, 3H, CH_3_), 2.64 (t, 0.75H, *J =* 7.1 Hz, CH_2_), 3.07 (t, 1.25H, *J =* 7.3 Hz, CH_2_), 4.01–4.50 (m, 4H, 2CH_2_), 7.15–8.15 (m, 12H, H_ar_, CH_,_ NH_2_), 11.39 (s, 0.7H, NH), 11.45 (s, 0.3H, NH); Anal. Calcd. for C_23_H_25_N_5_O_5_S_2_: C 53.58; H 4.89; N 13.58%. Found: C 53.23; H 4.67; N 13.34%.

#### 3.1.18. 3-((5-Chloro-4-(4-chlorophenyl)thiazol-2-yl)(4-sulfamoylphenyl)amino)propanoic acid (**36**)

Thiazole 35 (0.70 g, 1.6 mmol) and N-chlorosuccinimide (0.60 g, 4.5 mmol) were dissolved into 3 mL of DMF, and the reaction mixture was stirred at 0 °C for 6 h. Then, it was diluted with cold water, and the precipitate was filtered off and dissolved into 20 mL of 5% aqueous NaOH solution. The mixture was acidified with glacial acetic acid to pH 6 to afford light-yellowish solid, yield 0.53 g (71%); m.p. 109–110 °C; IR (KBr) (v, cm^−1^): 3353, 1727, 1510; ^1^H NMR (400 MHz, DMSO-*d_6_*) δ (ppm): 2.67 (t, 2H, *J =* 7.1 Hz, CH_2_), 4.20 (t, 2H, *J =* 7.1 Hz, CH_2_), 7.46 (s, 2H, NH_2_), 7.54 (d, 2H, *J =* 8.4 Hz, H_ar_), 7.72 (d, 2H, *J =* 8.4 Hz, H_ar_), 7.91 (d, 4H, *J =* 8.4 Hz, H_ar_), 12.32 (br. s, 1H, OH); ^13^C NMR (101 MHz, DMSO-*d_6_*) (δ, ppm): 32.30, 48.13, 107.25, 126.10, 127.65, 128.60, 129.44, 131.42, 132.97, 142.31, 144.05, 146.30, 163.41, 172.46; Anal. Calcd. for C_19_H_16_Cl_2_N_2_O_4_S_2_: C 48.41; H 3.42; N 5.94%. Found: C 48.29; H 3.35; N 5.99%.

### 3.2. Determination of Compounds Binding to Human CA Isozymes

#### 3.2.1. Protein Preparation

Human CA isozymes were expressed in *E. coli* (CAI, II, III, IV, VA, VB, VII, XII, XIII, XIV) or mammalian cells (CAVI and CAIX), chromatographically purified following the published protocols [47], and stored at −80 °C. The protein concentration was determined by UV absorption at 280 nm using Beer–Lambert law. The CAII used for crystallization was additionally purified by affinity chromatography.

#### 3.2.2. Fluorescent Thermal Shift Assay (FTSA)

The fluorescent thermal shift assay was performed to obtain the *observed* binding constants (inversely proportional to the *observed* dissociation constants (*K_d_obs_*s)) between CA isozymes and synthesized compounds. The QIAGEN Rotor-Gene Q was used to carry out the experiments, with a blue channel used for excitation (365 nm) and detection (460 nm). FTSA in this case relies on an extrinsic probe, 8-anilino-1-naphthalenesulfonate (ANS), to detect the fluorescence increase during protein denaturation, from which protein melting temperature (*T_m_*) can be determined. Since the binding compounds usually thermally stabilize a protein, an increase in *T_m_* is observed when increasing the compound concentration. The observed affinity of ligands for CAs was determined by plotting the *T_m_* as a function of ligand concentration and then fitting a curve as described previously [48]. Samples consisted of constant concentration of CAs (5 µM for all proteins except 7.5 or 10 µM of CAIV) and varied concentrations of ligand ((0–200) µM) in 50 mM sodium phosphate buffer at pH 7.0 containing 100 mM NaCl, 50 µM ANS, and 2% (*v*/*v*) DMSO. Samples were heated from 25 °C to 99 °C at the speed of 1 °C/min. An example of an experiment is given in Figure 2.

#### 3.2.3. Isothermal Titration Calorimetry (ITC)

ITC determined the observed standard enthalpy changes upon binding as well as the *K_d_obs_* values [41] that were compared to ones obtained by FTSA. The ITC experiments were performed by adding the proteins to the calorimeter cell and the compound solutions to the syringe at 37 ºC. The CAII binding with compounds **28**, **31**, and **36** and CAVB with compound **32** (Figure S104) were determined using a MicroCal PEAQ-ITC calorimeter (Northampton, MA, USA). Experiments consisted of 19 injections, the first injection of 0.4 μL over 0.8 s, with the remaining injections of 2 μL over 4 s with spacing of 150 s between injections, at temperature 37 °C, reference power 10 μcal/s, and stirring speed of 750 rpm. The protein concentrations for CAII and CAVB were 10 μM and 8 μM, respectively; the compound concentration was 10 times higher than the protein (100 μM for compounds tested with CAII, 80 μM for compound tested with CAVB). Proteins and compounds were diluted in sodium phosphate 50 mM buffer at pH 7.0, containing 100 mM NaCl and 2% (*v*/*v*) DMSO. Data were analyzed using NITPIC (version 1.2.7) [49,50] and Sedphat (v. 14.0) [51] software; the heat of dilution was subtracted from the titration curves.

#### 3.2.4. Determination of Compound Sulfonamide Group p*K_a_*

To calculate the intrinsic thermodynamic parameters of compound binding to CA isozymes, the p*K_a_* values of the sulfonamide group and the water molecule bound to the Zn(II) ion at the active site of CA proteins had to be determined. The p*K_a_* values for CA isozyme-bound water molecules were taken from [40], CAI—8.1; CAII—6.9; CAIII—6.5; CAIV—6.6; CAVA—6.7; CAVB—7.0; CAVI—6.0; CAVII—6.8; CAIX—6.6; CAXII—6.8; CAXIII—8.0; CAXIV—6.1. Compound p*K_a_* values were measured by obtaining UV-Vis spectra at 37 °C at different pH values (from 7.0 to 12.0 with 0.5 pH increment) using BMG Labtech CLARIOstar^Plus^ plate reading spectrophotometer. Compounds were diluted to a constant concentration of 50–200 µM (concentration was chosen depending on compound solubility and absorbance peak) in a universal buffer, consisting of 50 mM sodium phosphate, 50 mM sodium acetate, 25 mM sodium borate, and 50 mM sodium chloride, and also contained 2% (*v*/*v*) DMSO. The p*K_a_* values were calculated as described previously in [28,52] by normalizing the absorbance, plotting it as a function of pH, then fitting to the Henderson–Hasselbalch equation using the least square method (Figure S105).

The p*K_a_*s of compounds **27**, **28**, **29**, **31**, **32**, **35**, and **36** were measured, with an average p*K_a_* value of 10.1 for all compounds. Since the p*K_a_* values were only able to be measured for compound group **26**–**36**, due to universal buffer pH limitations, intrinsic thermodynamic parameters were not calculated for compounds **2**(**a**,**b**)–**25**(**a**,**b**), only for compounds **26**–**36** using experimentally obtained p*K_a_* values when possible and using the average p*K_a_* value mentioned above for compounds not measured.

#### 3.2.5. Calculation of the Intrinsic Thermodynamic Parameters

The intrinsic thermodynamic parameters were calculated as described in [40]. The intrinsic reaction of inhibitor binding to CA is shown in Equation (1). The *observed* reaction includes the associated protonation–deprotonation reactions of protein, ligand, and buffer and thus has to be accounted for in order to make correct conclusions about compound binding affinities. It is known that CA proteins bind sulfonamides only when water molecules are coordinated to the Zn(II) ion, and sulfonamides only bind to CA proteins when their amino group is deprotonated (confirmed by neutron crystallography [53,54,55]):

(1) $${\mathrm{CA}\;\mathrm{Zn}}^{\mathrm{II}}-H_{2}O+{\mathrm{RSO}}_{2}{\mathrm{NH}}^{-}⇄{\;\mathrm{CA}\;\mathrm{Zn}}^{\mathrm{II}}-{\mathrm{NH}\;\mathrm{SO}}_{2}R+H_{2}O$$

When calculating *K_d_int_* values, these protein and ligand fractions from Equation (1) have to be accounted for (Equations (2) and (3)). The intrinsic dissociation constant is calculated by multiplying *K_d_obs_* value by the corresponding fractions of protein and ligand (Equation (4)).

(2) $$f_{RSO_{2}NH^{-}}=\frac{10^{pH-pK_{a\_RSO_{2}NH_{2}}}}{1+10^{pH-pK_{a\_RSO_{2}NH_{2}}}}$$

(3) $$f_{CAZnH_{2}O}=1-\frac{10^{pH-pK_{a\_CAZnH_{2}O}}}{1+10^{pH-pK_{a\_CAZnH_{2}O}}}$$

(4) $$K_{d\_int}=K_{d\_obs}\times f_{RSO_{2}NH^{-}}\times f_{CAZnH_{2}O}$$

(5) $$\Delta G_{int}^{0}=RTln\left(K_{d\_int}\right)$$

The intrinsic standard Gibbs energy changes upon binding were calculated using Equation (5). Here, *R*—universal ideal gas constant (8.3144 J/mol·K), *T*—absolute temperature (310.15 K).

#### 3.2.6. X-ray Crystallography: Crystallization, Data Collection, and Structure Determination

CAII was concentrated by ultrafiltration to 31 mg/mL and mixed with the crystallization solution (0.1 M sodium bicine (pH 9.0), 0.2 M ammonium sulfate, and 2 M sodium malonate (pH 7.0)) at a ratio 1:1 and crystallized by the sitting drop method. One μL of 50 mM compound solution in DMSO was combined with 50 μL of reservoir solution for crystals soaking.

X-ray diffraction data were collected at the EMBL beamline P13 at PETRA III ring of DESY synchrotron in Hamburg. Crystals were flash cooled to 100 K without additional cryo-protection. The data were processed and scaled using XDS [56], MOSFLM [57], and SCALA [58]. MOLREP [59] was used for molecular replacement, with an initial model of 4HT0. The model was refined with REFMAC [60] and fitted in the electron density map using COOT [61]. The 3D models of compounds were constructed by the AVOGADRO [62] program, while ligand parameter files were created using LIBCHECK [63,64]. The PDB IDs, data collection, and refinement statistics are listed in Table S2 in Supplementary Materials. The 2D schemes based on X-ray crystallography are shown as LigPlot+ [65] schemes in Figure S3.

## 4. Conclusions

A series of benzenesulfonamides bearing *para*-*N* β,γ-amino acid or *para*-*N* β-amino acid and thiazole moieties were synthesized, and the binding affinities for the entire family of 12 catalytically active human CA isozymes were determined by the fluorescent thermal shift assay. Selected compound affinities were also determined by isothermal titration calorimetry, while X-ray crystallography helped to determine the structural arrangement of the inhibitors in the active site of CAII. The intrinsic affinities that are independent of protonation reactions were determined according to the described model. The analysis showed that the chlorine atom in the *meta*-position of *para*-*N* β-amino acid bearing benzenesulfonamide derivatives increases the observed affinity for CA over non-chlorinated compounds, while the substituents located further away from this group of benzenesulfonamides have lower influence on the affinity. The most potent compound, **36**, bearing *para*-*N* β-amino acid and thiazole moieties bound CAI and CAIX with 20 nM *K_d_obs_*. Correlation of the chemical structures of the compounds with the intrinsic affinities showed that small changes in the chemical structure of the compound can have significant effects on the affinity.

## Figures and Tables

**Table 1:** The *observed* dissociation constants of CAI, CAII, CAIX, CAXII, and CAXIII with tested compounds at pH 7.0 and 37 °C by the fluorescent thermal shift assay. ND–*K_d_obs_* value was not determined. *K_d_obs_* data of compounds **21 [30]**, **26 [31]**, and **35 [31]** were taken from previous publications, as well as compound laboratory names marked with *. The standard deviation of the FTSA measurements is ±1.6-fold in *K_d_obs_*.

Cmpd.	Lab. Name	Observed Dissociation Constant (*K_d_obs_*), nM				
		CAI	CAII	CAIX	CAXII	CAXIII
**2a**	2a	78,000	14,000	4000	71,000	130,000
**2b**	2b	6700	1300	830	14,000	6700
**3a**	BB2.2-7	170,000	13,000	6700	110,000	100,000
**3b**	BB2.1-7	10,000	1800	670	14,000	6700
**4a**	BB2.2-8	18,000	40,000	2000	18,000	20,000
**4b**	BB2.1-8	2900	330	250	4000	1300
**5a**	BB2.2-19	8300	1300	590	500	5000
**5b**	BB2.1-19	4000	250	180	6700	400
**6a**	3a	14,000	1800	560	25,000	7700
**7a**	BB2.2-20	6700	2000	500	20,000	10,000
**7b**	BB2.1-20	1000	170	67	5000	170
**8a**	BB2.2-21	10,000	2000	670	33,000	5000
**8b**	BB2.1-21	1000	250	100	20,000	200
**9a**	BB2.2-22	5900	1300	500	25,000	4300
**10a**	BB2.2-24	17,000	2500	500	50,000	1700
**10b**	BB2.1-24	1700	330	130	50,000	130
**11a**	BB2.2-25	20,000	3300	1000	100,000	5000
**11b**	BB2.1-25	2500	330	130	33,000	330
**12a**	4a	36,000	5000	1000	66,000	17,000
**12b**	4b	6700	1100	670	57,000	1500
**13a**	5a	22,000	2900	1400	23,000	20,000
**13b**	5b	2600	390	330	23,000	340
**14a**	6a	32,000	3400	1400	38,000	23,000
**14b**	6b	510	59	290	10,000	66
**15a**	BB2.2-17	13,000	3300	1000	50,000	6700
**15b**	BB2.1-17	2500	500	140	50,000	400
**16a**	BB2.2-18	20,000	4000	1400	50,000	14,000
**16b**	BB2.1-18	3300	500	130	130,000	1000
**17a**	7a	36,000	5400	3300	79,000	19,000
**17b**	7b	2600	570	1400	58,000	1400
**19a**	BB2.2-9	10,000	1700	560	10,000	4000
**21**	10 *	250	66	220	1400	ND
**23a**	BB2.2-11	4000	2000	130	2200	10,000
**24a**	BB2.2-31	43,000	6700	910	3300	19,000
**24b**	BB2.1-31	2900	250	59	1700	1300
**25a**	BB2.2-12	100,000	3300	100	4000	25,000
**26**	19 *	10,000	5000	10,000	33,000	ND
**35**	31 *	25	100	560	200	530

**Table 2:** The *observed* dissociation constants of all 12 catalytically active CAs with tested compounds at pH 7.0 and 37 °C by the fluorescent thermal shift assay. The value ≥ 200,000 is the upper limit of *K_d_obs_* detection; ND–*K_d_obs_* value was not determined. The standard deviation of the FTSA measurements is ± 1.6-fold in *K_d_obs_*. The ITC data are shown in the brackets (pH 7.0, 37 °C).

Cmpd.	Lab. Name	Observed Dissociation Constants (*K_d_obs_*), nM											
		CAI	CAII	CAIII	CAIV	CAVA	CAVB	CAVI	CAVII	CAIX	CAXII	CAXIII	CAXIV
**6b**	3b	1400	240	≥200,000	4200	20,000	130	670	290	100	7900	180	140
**9b**	BB2.1-22	670	200	≥200,000	1000	2900	830	670	250	100	14,000	250	130
**18a**	BB2.2-23	20,000	5000	≥200,000	33,000	20,000	14,000	33,000	6700	1400	20,000	25,000	5000
**18b**	BB2.1-23	5000	1300	≥200,000	13,000	6700	6700	20,000	330	250	13,000	1800	1000
**19b**	BB2.1-9	290	50	≥200,000	1800	20,000	1400	5000	200	56	1300	130	25
**20**	BB2.1-10	5000	500	≥200,000	2000	22,000	3300	20,000	10,000	100	13,000	2900	330
**22a**	BB2.2-30	50,000	2500	≥200,000	6700	33,000	25,000	13,000	10,000	330	11,000	33,000	3300
**22b**	BB2.1-30	3300	400	≥200,000	2000	6700	≥200,000	3300	670	100	2200	1700	130
**23b**	BB2.1-11	67	50	15,000	1000	20,000	1100	1000	110	33	50	400	50
**25b**	BB2.1-12	170	25	5000	1400	38,000	1400	200	170	25	630	400	25
**27**	BB8.1-7	67	220	63,000	1400	≥200,000	560	18,000	250	48	180	870	170
**28**	BB8.1-12	37	45 (59)	≥200,000	1100	50,000	46	7100	77	33	250	250	29
**29**	BB8.1-14	150	380	≥200,000	1500	≥200,000	77	20,000	670	80	830	1700	130
**30**	BB8.1-15	3300	4000	≥200,000	27,000	≥200,000	11000	67,000	100,000	290	110,000	8300	6700
**31**	BB8.1-4	2000	910 (860)	≥200,000	6700	≥200,000	20,000	24,000	1700	360	3800	450	2000
**32**	BB8.1-5	830	53	≥200,000	2000	≥200,000	30 (41)	10,000	130	190	7700	330	770
**33**	BB8.1-10	3100	500	≥200,000	2800	≥200,000	100	25,000	1100	630	26,000	830	910
**34**	BB8.1-13	670	150	≥200,000	1000	≥200,000	120	10,000	250	170	14,000	220	1000
**36**	BB8.3-3	20	40 (130)	17,000	3300	≥200,000	400	6300	33	23	120	40	130

**Table 3:** The intrinsic dissociation constants (*K_d_int_*) for compounds binding to CAs at 37 °C. ND—not determined. *—the p*K_a_* value was not determined and is an average of all measured compounds. Experimental data and statistics of p*K_a_* values are provided in Supplementary Materials.

Cmpd.	Lab. Name	Sulfonamide p*K_a_*	Intrinsic Dissociation Constants (*K_d_int_*), nM											
			CAI	CAII	CAIII	CAIV	CAVA	CAVB	CAVI	CAVII	CAIX	CAXII	CAXIII	CAXIV
**27**	BB8.1-7	10.4	0.025	0.039	6.0	0.16	≥27	0.11	0.65	0.038	0.0054	0.028	0.31	0.0076
**28**	BB8.1-12	10.1	0.027	0.016	≥38	0.25	13	0.018	0.51	0.024	0.0075	0.077	0.18	0.0026
**29**	BB8.1-14	10.3	0.070	0.084	≥24	0.21	≥33	0.019	0.91	0.13	0.011	0.16	0.77	0.0073
**31**	BB8.1-4	10.1	1.5	0.32	≥38	1.5	≥53	7.9	1.7	0.52	0.081	1.2	0.32	0.18
**32**	BB8.1-5	9.7	1.5	0.047	≥96	1.1	≥130	0.030	1.8	0.10	0.11	5.9	0.60	0.17
**33**	BB8.1-10	10.1 *	2.3	0.18	≥38	0.63	≥53	0.040	1.8	0.34	0.14	8.0	0.60	0.081
**36**	BB8.3-3	10.2	0.012	0.011	2.6	0.59	≥42	0.13	0.36	0.0081	0.0041	0.029	0.023	0.0092

## Data Availability

Data is contained within the article and Supplementary Materials.

## Supplementary Materials

The following supporting information can be downloaded at: https://www.mdpi.com/article/10.3390/ph15040477/s1, Table S1: Intrinsic standard Gibbs energy change values for compounds binding to CAs; Table S2: X-ray crystallography data collection and refinement statistics; Figure S1: Determination of compound **32** binding to human CAs by the fluorescent thermal shift assay; Figure S2: The electron densities of the ligands in the active site of CAII; Figure S3: 2D schemes of interaction of compound **28** (PDB ID: 7QGZ), compound **31** (PDB ID: 7QGY), and compound **36** (PDB ID: 7QGX) with the active site of CAII; Figures S4–S103: ^1^H or ^13^C NMR of compounds at 400 MHz (DMSO-*d_6_*) or at 101 MHz (DMSO-*d_6_*), respectively; Figure S104: Isothermal titration calorimetry data of CAII binding with compounds **31**, **28** and **36**; Figure S105: p*K_a_* of sulfonamide amino group.
