# Supplementary material for: Beta and Gamma Amino Acid-Substituted Benzenesulfonamides as Inhibitors of Human Carbonic Anhydrases
Source: Pharmaceuticals (Basel). 2022 Apr 13;15(4):477. doi: 10.3390/ph15040477 (PMC9033141; doi:10.3390/ph15040477)
Supplement: Supplementary file 1 [file pharmaceuticals-15-00477-s001.zip › pharmaceuticals-1614677-supplementary.pdf]

Supplementary material for the manuscript:

Beta and Gamma Amino Acid-Substituted Benzenesulfonamides as Inhibitors of Human Carbonic Anhydrases

Benas Balandis [1], Tomas Šimkūnas [2], Vaida Paketurytė-Latvė [2], Vilma Michailovienė [2], Aurelija Mickevičiūtė [2], Elena Manakova [3], Saulius Gražulis [3], Sergey Belyakov [4], Visvaldas Kairys [5], Vytautas Mickevičius [1], Asta Zubrienė [2] and Daumantas Matulis [2,*]

[1] Department of Organic Chemistry, Kaunas University of Technology, LT-50254, Radvilėnų pl. 19,
Kaunas LT-50254, Lithuania; benas.balandis@ktu.edu (B.B.); vytautas.mickevicius@ktu.lt (V.M.)
[2] Department of Biothermodynamics and Drug Design, Institute of Biotechnology, Life Sciences Center,
Vilnius University, Saulėtekio 7, Vilnius LT-10257, Lithuania; tomas.simkunas@gmc.stud.vu.lt (T.Š.); vaida.paketuryte@gmc.vu.lt (V.P.-L.); vilma.michailoviene@bti.vu.lt (V.M.); aurelija.mickeviciute@bti.vu.lt (A.M.); asta.zubriene@bti.vu.lt (A.Z.)
[3] Department of Protein–DNA Interactions, Institute of Biotechnology, Life Sciences Center,
Vilnius University, Saulėtekio al. 7, Vilnius LT-10257, Lithuania; elena.manakova@bti.vu.lt (E.M.); saulius.grazulis@bti.vu.lt (S.G.)
[4] Laboratory of Physical Organic Chemistry, Latvian Institute of Organic Synthesis, Aizkraukles 21,
Riga LV-1006, Latvia; serg@osi.lv (S.B.)
[5] Department of Bioinformatics, Institute of Biotechnology, Life Sciences Center, Vilnius University, Saulėtekio al. 7, Vilnius LT-10257, Lithuania; visvaldas.kairys@bti.vu.lt (V.K)
* Correspondence: daumantas.matulis@bti.vu.lt (D.M); Tel.: +370-5-223-4364; Fax: +370-5-223-4367

Table of Contents

Fluorescent thermal shift assay data 2

Intrinsic Gibbs energy changes 3

X-ray crystallography of human CAII complexes with inhibitors........ 4

NMR spectra of synthesized compounds 8

Isothermal titration calorimetry data 62

pK_a values of sulfonamide amino group 63

References........ 64

Fluorescent thermal shift assay data

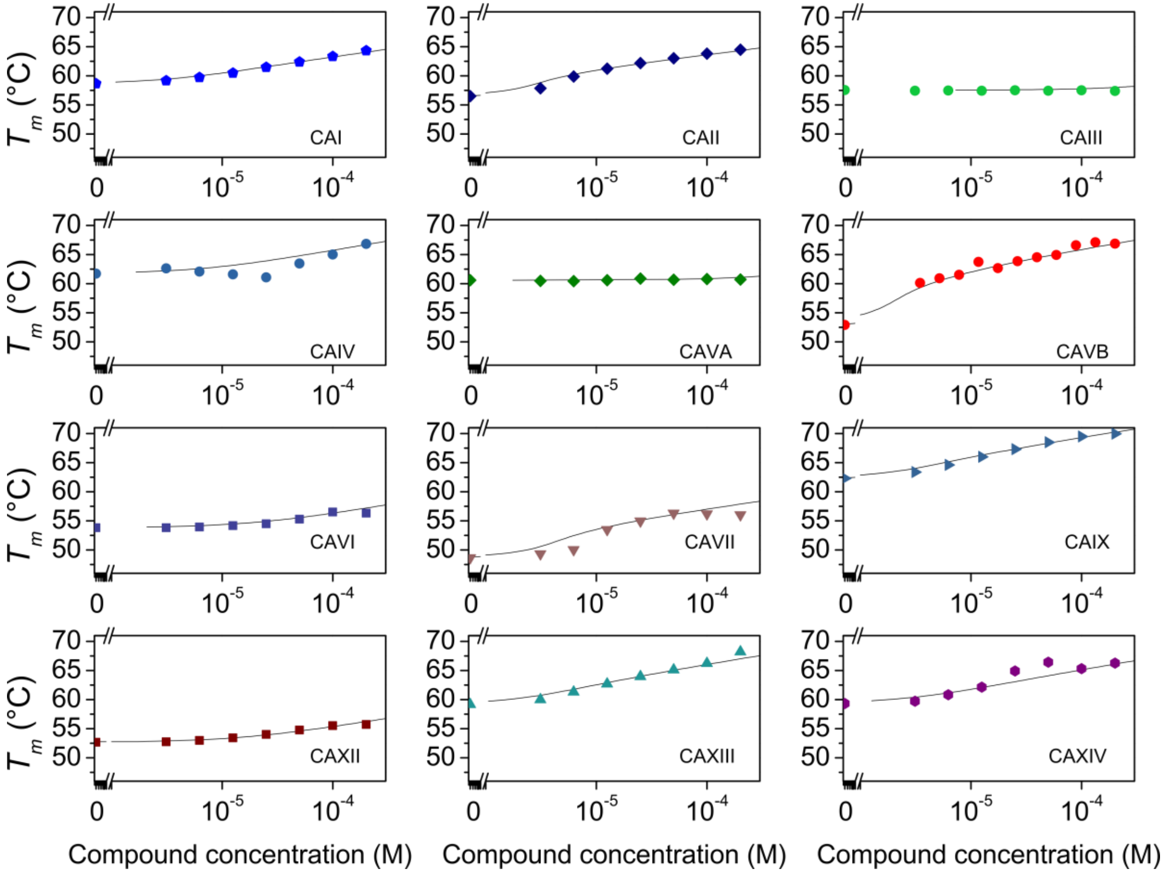

Figure S1. Determination of compound **32** binding to human CAs by the fluorescent thermal shift assay (FTSA) in 50 mM sodium phosphate buffer containing 100 mM NaCl at pH 7.0. The melting temperatures plotted as a function of added compound concentration (data points) and fitted to a model for 37 °C (solid lines). K_{d_obs} values are listed in Table 2.

Intrinsic Gibbs energy changes

Table S1. Intrinsic standard Gibbs energy change (ΔG_{int}) values for compounds binding to CAs at 37 °C. ND – not determined.

Cmpd.	Lab. name	Intrinsic Gibbs energy change (ΔG_{int}), kJ/mol											
		CAI	CAII	CAIII	CAIV	CAVA	CAVB	CAVI	CAVII	CAIX	CAXII	CAXIII	CAXIV
**27**	*BB8.1-7*	-62.9	-61.7	-48.7	-58.1	≤-44.9	-59.0	-54.5	-61.7	-66.8	-62.6	-56.3	-65.9
**28**	*BB8.1-12*	-62.6	-64.0	≤-44.0	-56.9	-46.7	-63.6	-55.1	-63.0	-66.0	-60.0	-57.8	-68.7
**29**	*BB8.1-14*	-60.2	-59.7	≤-45.2	-57.3	≤-44.3	-63.5	-53.6	-58.6	-64.9	-58.1	-54.0	-66.0
**31**	*BB8.1-4*	-52.4	-56.3	≤-44.0	-52.3	≤-43.1	-48.0	-51.9	-55.0	-59.8	-52.9	-56.2	-57.8
**32**	*BB8.1-5*	-52.3	-61.2	≤-41.6	-53.0	≤-40.8	-62.4	-51.8	-59.3	-59.1	-48.8	-54.7	-57.9
**33**	*BB8.1-10*	-51.2	-57.8	≤-44.0	-54.5	≤-43.1	-61.7	-51.8	-56.1	-58.4	-48.0	-54.7	-59.8
**36**	*BB8.3-3*	-64.8	-64.9	-50.9	-54.7	≤-43.7	-58.7	-56.0	-65.8	-67.5	-62.4	-63.1	-65.4

X-ray crystallography of human CAII complexes with inhibitors

Table S2. X-ray crystallography data collection and refinement statistics of human CAII complexes with inhibitors. All datasets were collected at 100 K, test set size was 10%.

Isoform-ligand	CAII –**31**(*BB8.1-4*)	CAII – **28** (*BB8.1-12*)	CAII – **36**(*BB8.3-3*)
PDB ID	7QGY	7QGZ	7QGX
Data-collection statistics			
Space group	$P12_11$	$P12_11$	$P12_11$
Unit-cell parameters (Å)	a=42.3,<br>b=41.1,<br>c=72.0,<br>β=104.2°	a=42.3,<br>b=41.2,<br>c=72.2,<br>β=104.3°	a=42.2,<br>b=41.0,<br>c=72.0,<br>β=104.3°
Resolution range (Å)	1.5-41.1	1.1-70	1.2-40.9
Wavelength (Å)	1.54187	0.976300	0.976300
Radiation source	Rigaku MicroMax™-007 HF	EMBL, P13	EMBL, P13
Unique reflections number	35659	85901	67066
R_{merge}, overall (outer shell)	0.024 (0.061)	0.090 (0.276)	0.100 (0.332)
I/σ overall (outer shell)	35.0 (8.6)	13.7 (5.8)	4.2 (2.5)
Multiplicity overall (outer shell)	4.8 (1.5)	6.4 (4.9)	2.4 (2.5)
Completeness (%) overall (outer shell)	92.5 (53.7)	95.4 (76.4)	87.1 (91.8)
Wilson B-factor	10.8	9.2	10.9
Refinement statistics			
R_{work}	0.1549	0.1983	0.1859
R_{free}	0.1943	0.2173	0.2111
RMSD bond lengths, (Å)	0.0221	0.0257	0.0212
RMSD bond angles (°)	2.2310	2.2748	2.2332
Average B factors (Å^2)			

All	14.09	13.51	14.86
main-chain	10.99	10.82	11.88
side-chain	14.14	13.60	14.33
Inhibitors	16.22	15.06	15.61
Waters	25.27	23.90	25.84
Zinc	6.32	5.39	6.68
Other molecules	-	-	36.59
Number of atoms			
All	2453	2473	2550
Protein	2129	2153	2153
Inhibitor	27	23	29
Water	296	296	360
Zinc	1	1	1
Other molecules	0	0	7
Ramachandran statistics (%)			
most favored regions	95	97	97
additionally allowed regions	5	3	3
Outliers	0	0	0

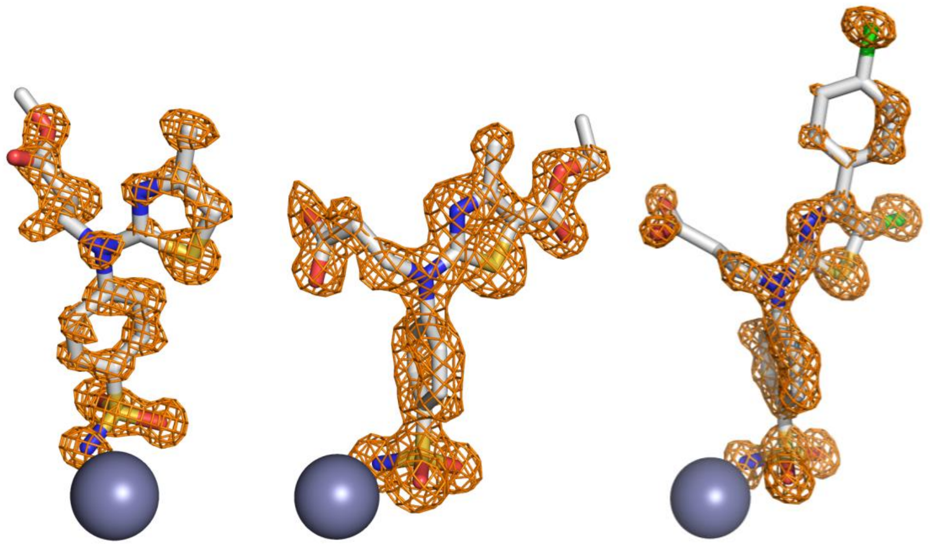

Figure S2. The electron densities |F(o)-F(c)| of the ligands calculated in the absence of ligand and contoured at 3σ. Zinc ion is shown as a gray sphere. Right panel: Compound **28** (*BB8.1-12*) (PDB ID: 7QGZ); middle panel: Compound **31** (*BB8.1-4*) (PDB ID: 7QGY); left panel: Compound **36** (*BB8.3-3*) (PDB ID: 7QGX) in the active site of CAII.

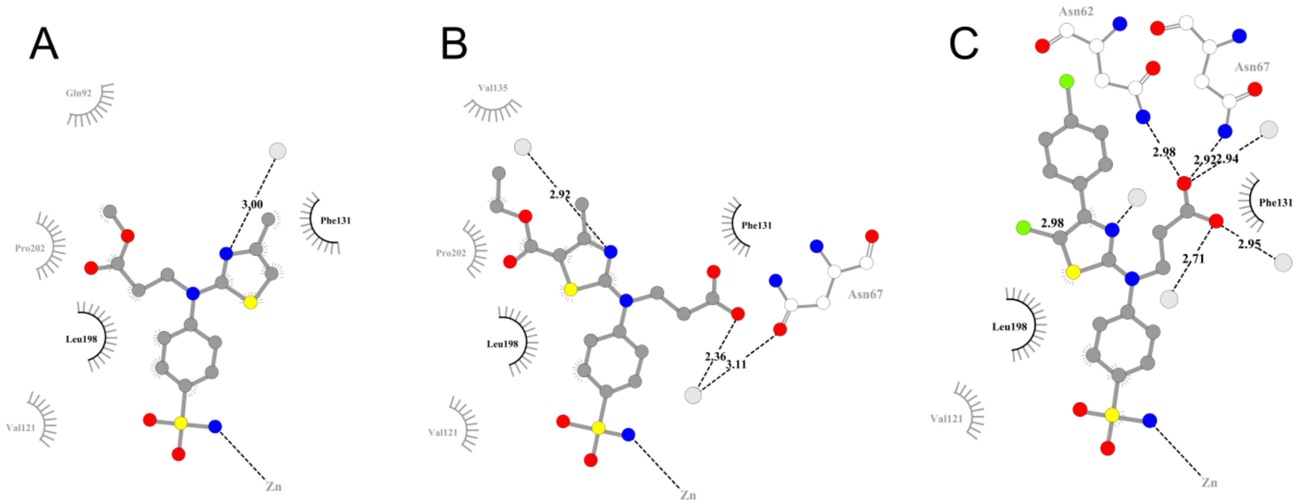

Figure S3. 2D schemes of interaction of (**A**) compound **28** (*BB8.1-12*) (PDB ID: 7QGZ), (**B**) compound **31** (*BB8.1-4*) (PDB ID: 7QGY) and (**C**) compound **36** (*BB8.3-3*) (PDB ID: 7QGX) with the active site of CAII. The lengths of the hydrogen bonds are given in angstroms and represented in dotted lines, while hydrophobic contacts are shown by an arc with spokes. The sulfonamide group bonds with Thr199 are not shown for simplicity. Water molecules are depicted as white spheres. Schemes were created using LigPlot+[1].

NMR spectra of synthesized compounds

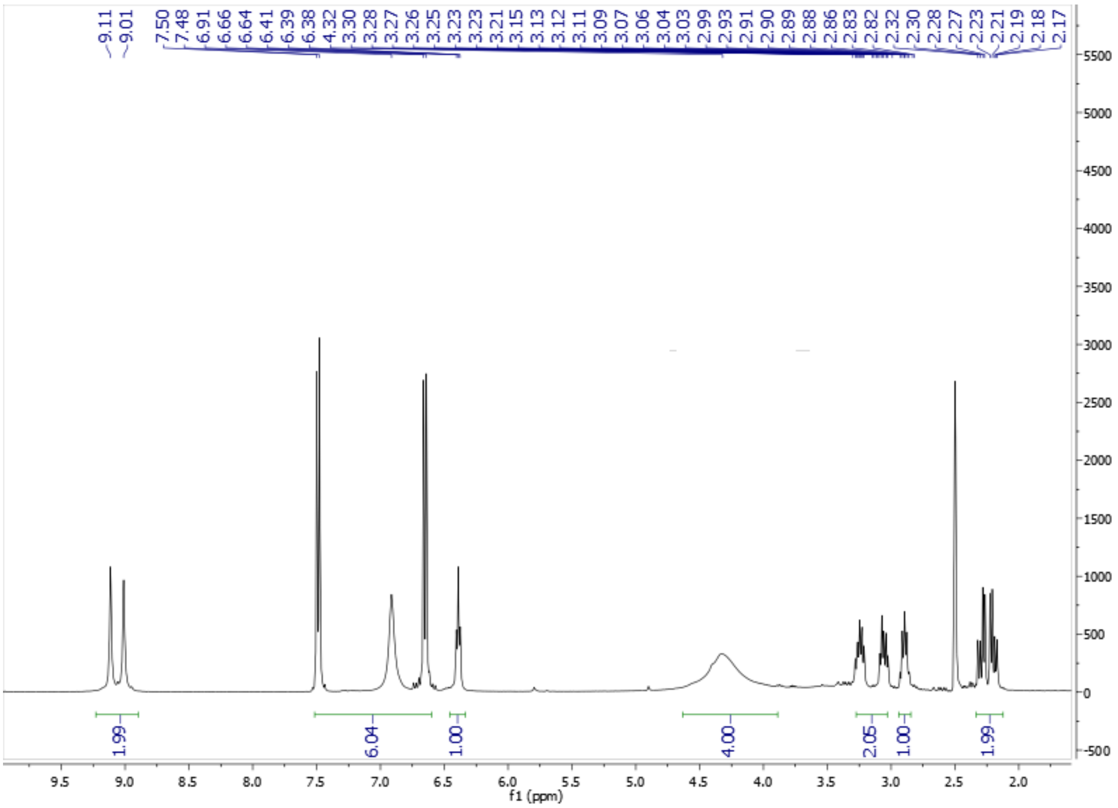

Figure S4. ^{1}H NMR of compound **2a** at 400 MHz (DMSO-*d*6)

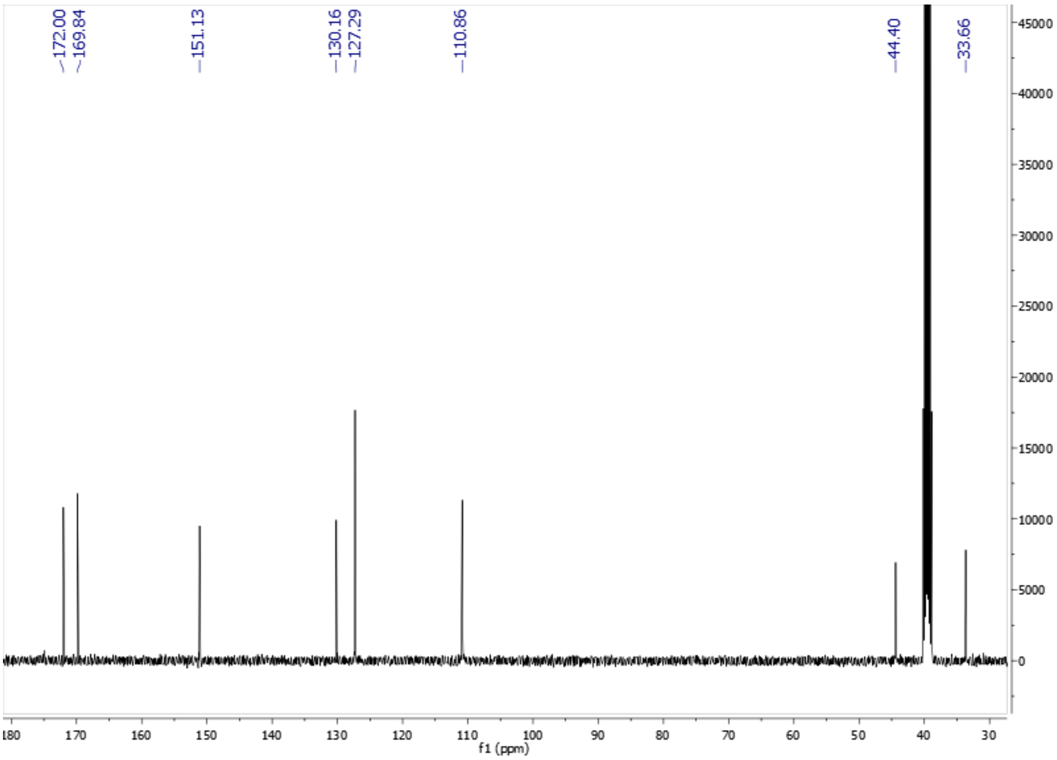

Figure S5. ^{13}C NMR spectrum of compound **2a** at 101 MHz (DMSO-*d*6)

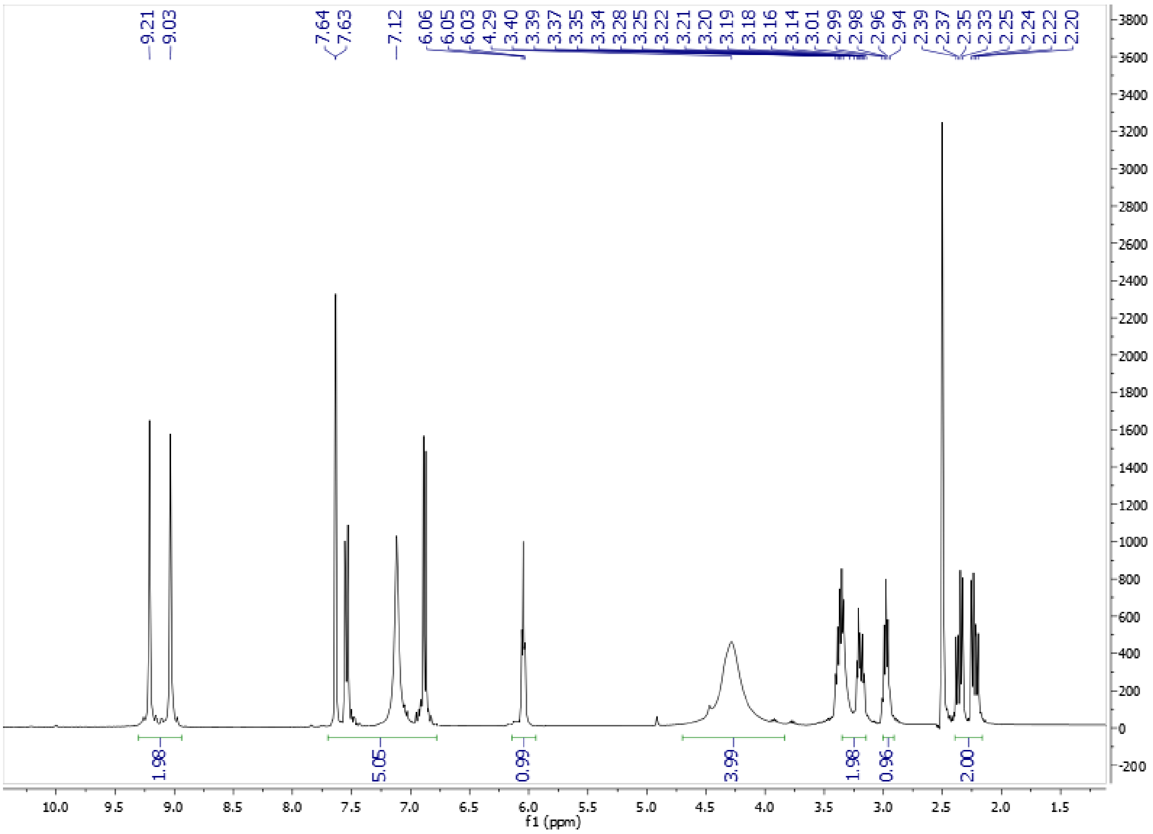

Figure S6. ^{1}H NMR of compound **2b** at 400 MHz (DMSO-d_6)

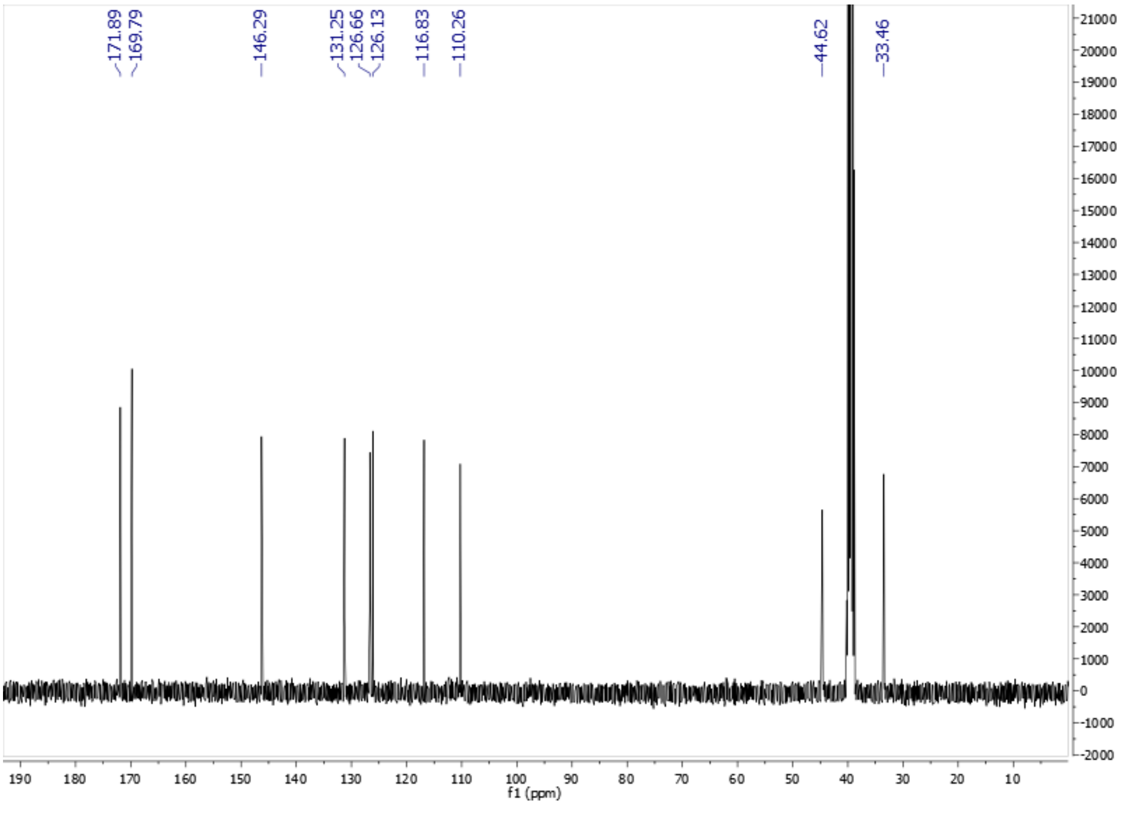

Figure S7. ^{13}C NMR spectrum of compound **2b** at 101 MHz (DMSO-d_6)

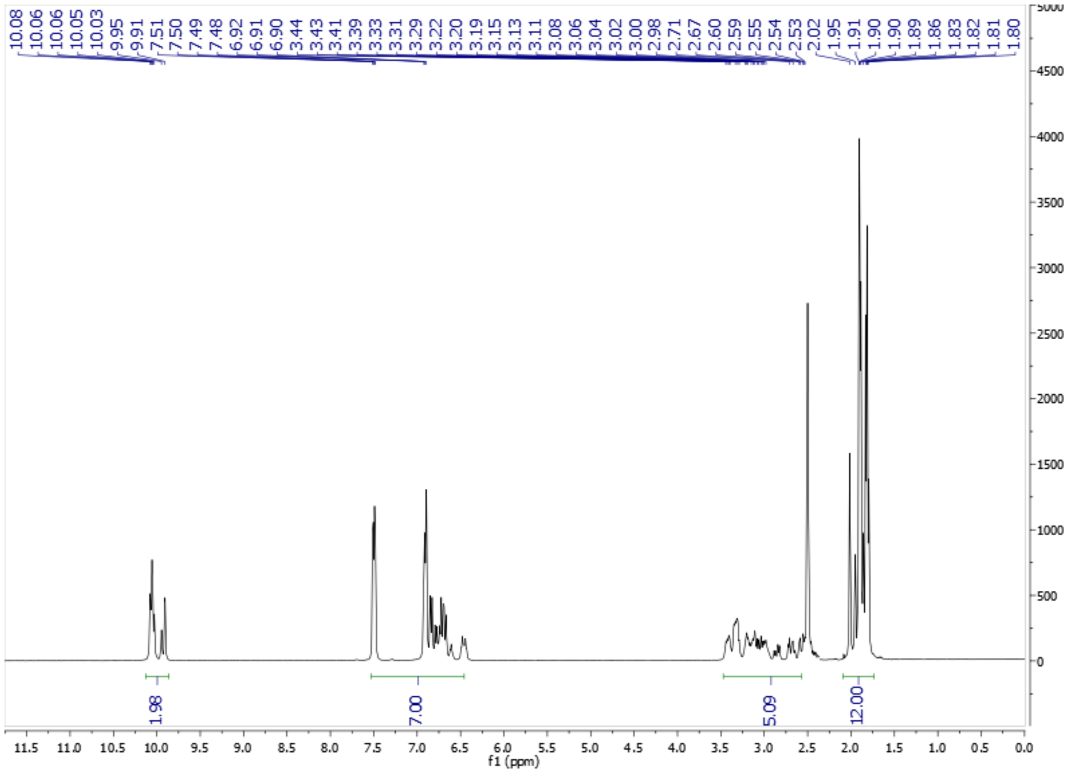

Figure S8. ^{1}H NMR of compound **3a** at 400 MHz (DMSO-d_6)

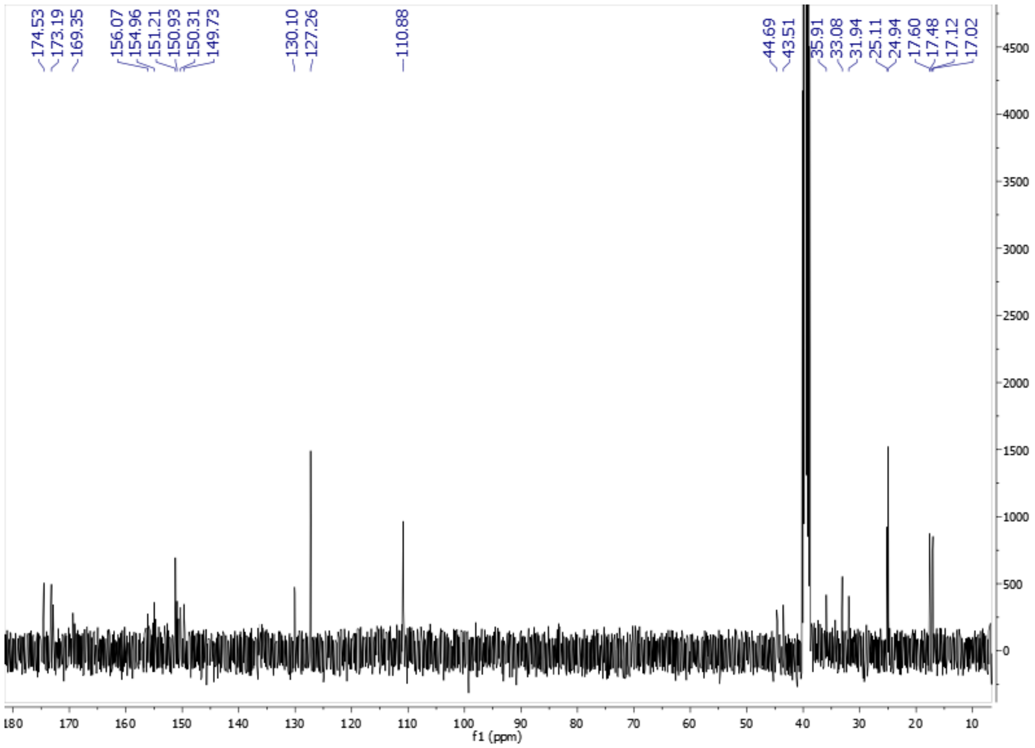

Figure S9. ^{13}C NMR spectrum of compound **3a** at 101 MHz (DMSO-d_6)

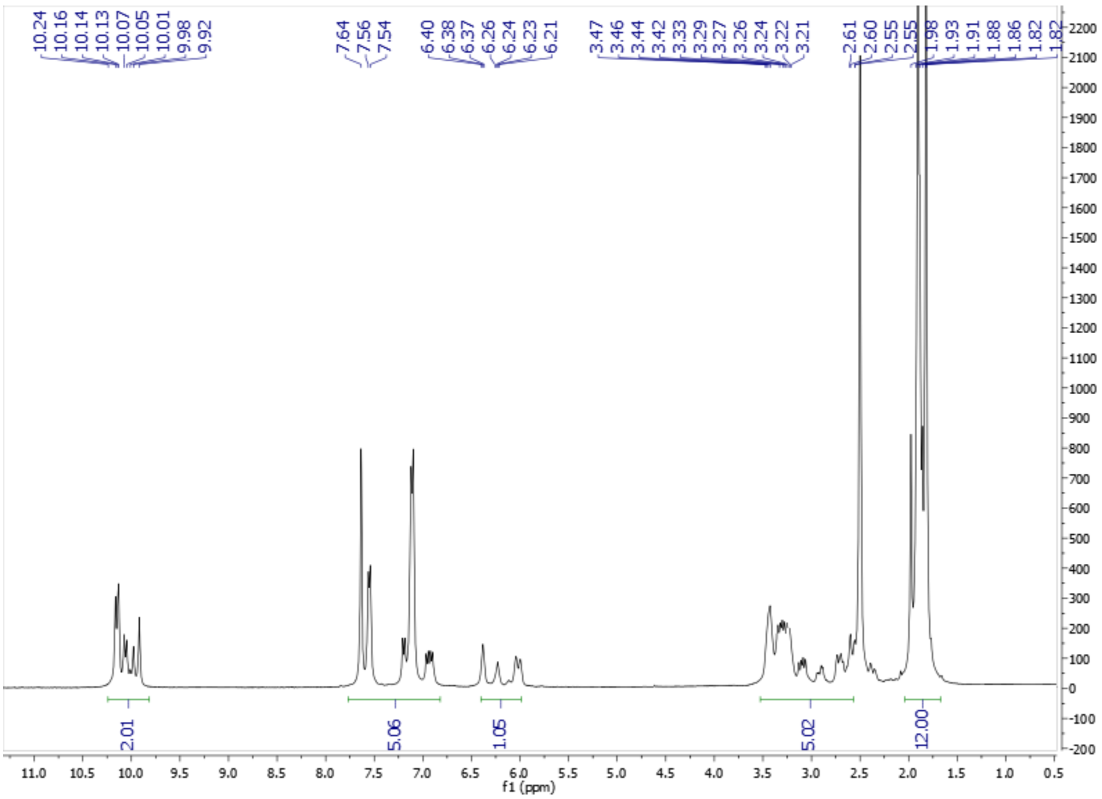

Figure S10. ^{1}H NMR of compound **3b** at 400 MHz (DMSO-d_6)

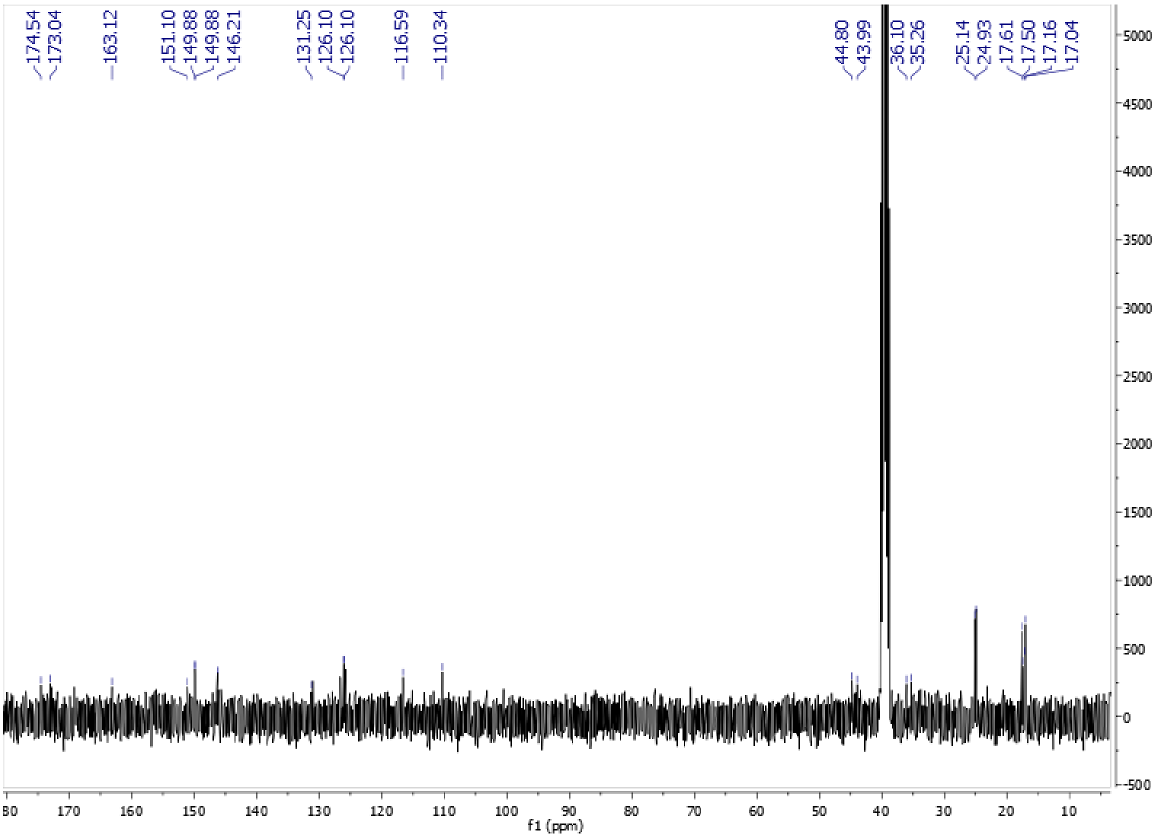

Figure S11. ^{13}C NMR spectrum of compound **3b** at 101 MHz (DMSO-d_6)

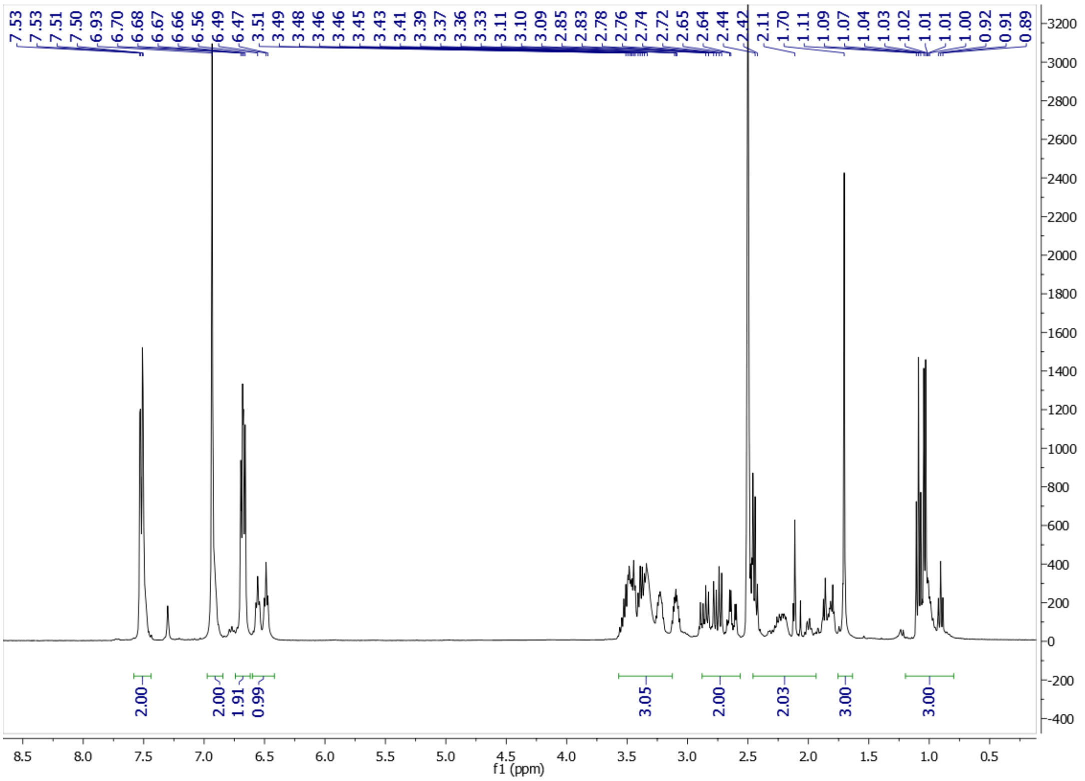

Figure S12. ^{1}H NMR of compound **4a** at 400 MHz (DMSO-d_6)

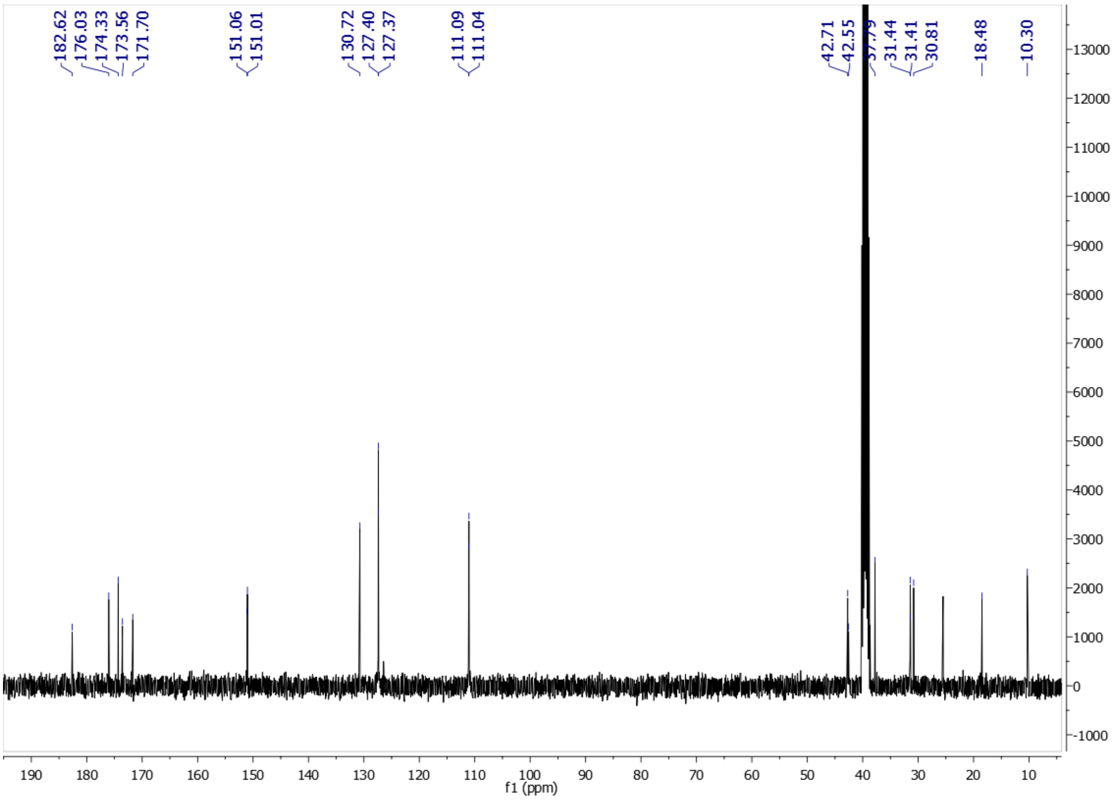

Figure S13. ^{13}C NMR of compound **4a** at 101 MHz (DMSO-d_6)

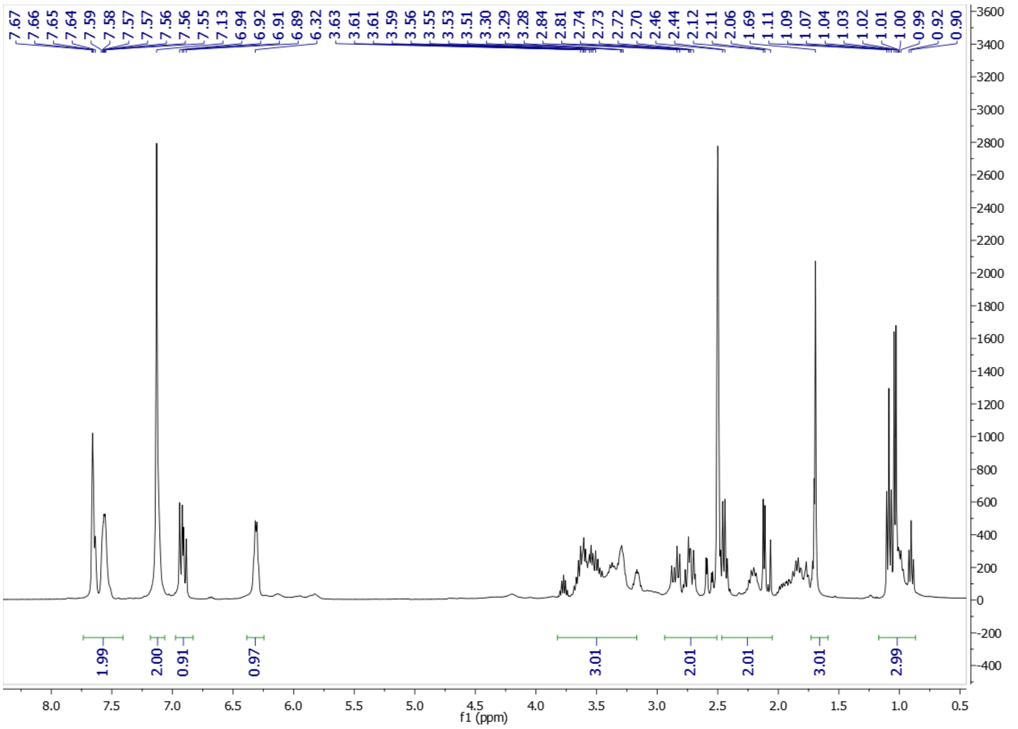

Figure S14. ^{1}H NMR of compound **4b** at 400 MHz (DMSO-d_6)

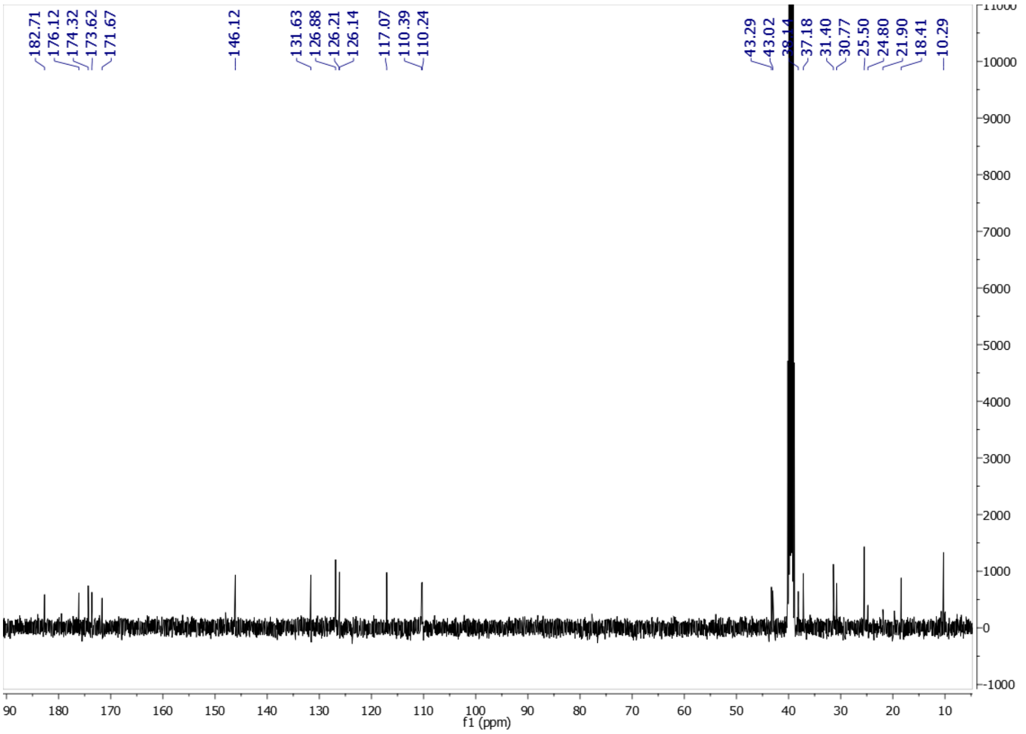

Figure S15. ^{13}C NMR spectrum of compound **4b** at 101 MHz (DMSO-d_6)

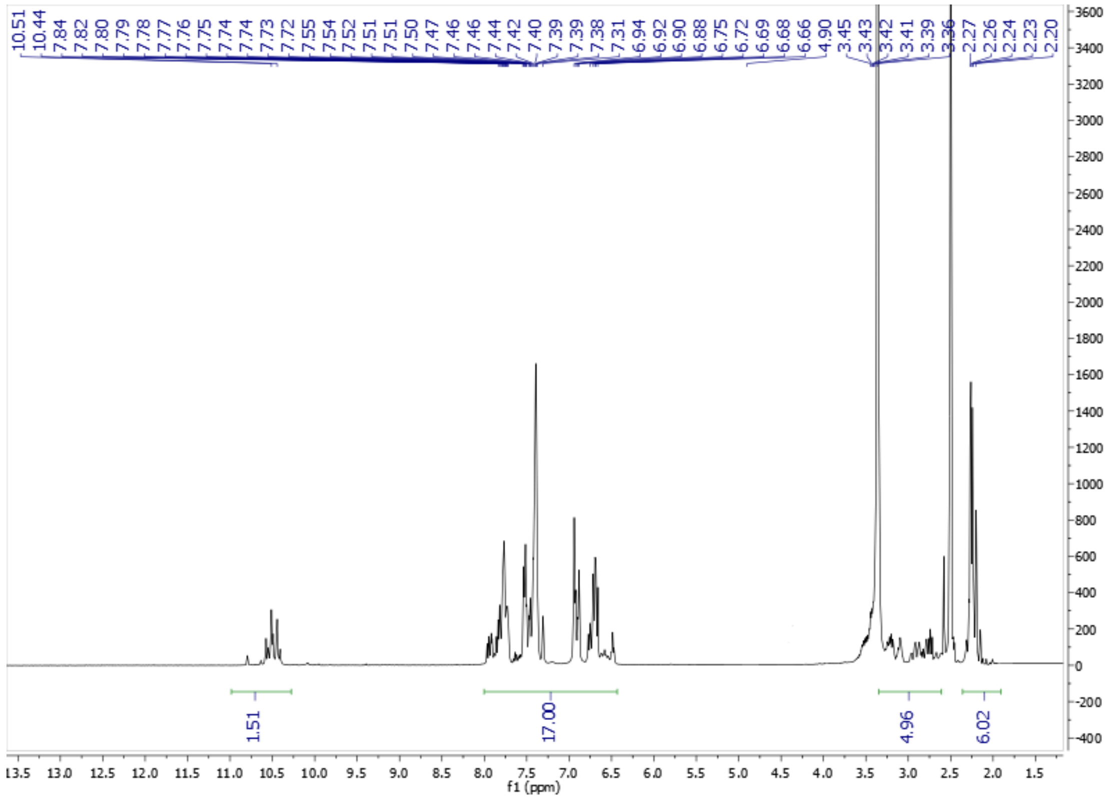

Figure S16. ^{1}H NMR of compound **5a** at 400 MHz (DMSO-d_6)

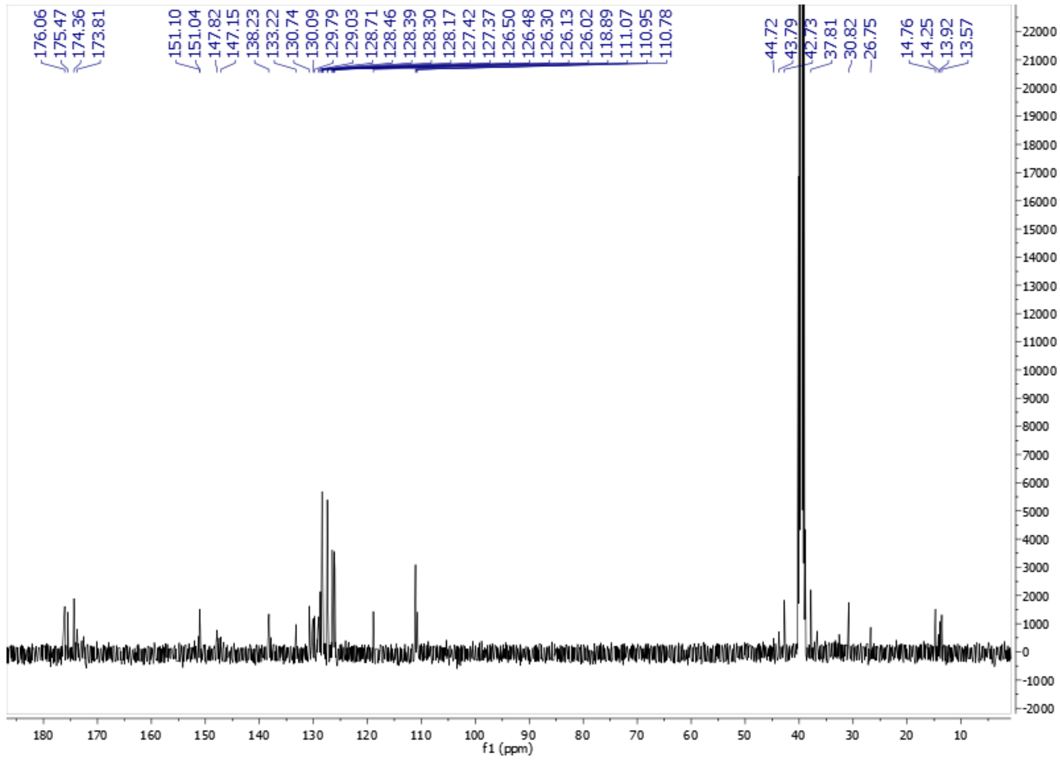

Figure S17. ^{13}C NMR spectrum of compound **5a** at 101 MHz (DMSO-d_6)

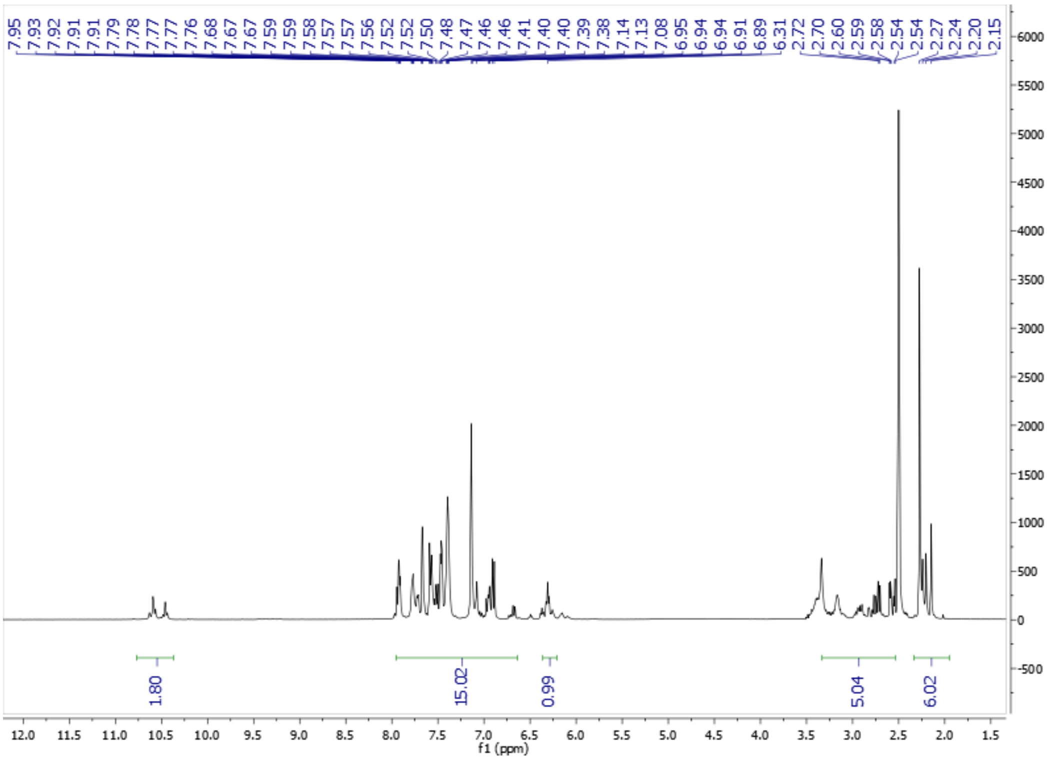

Figure S18. ^{1}H NMR of compound **5b** at 400 MHz (DMSO-d_6)

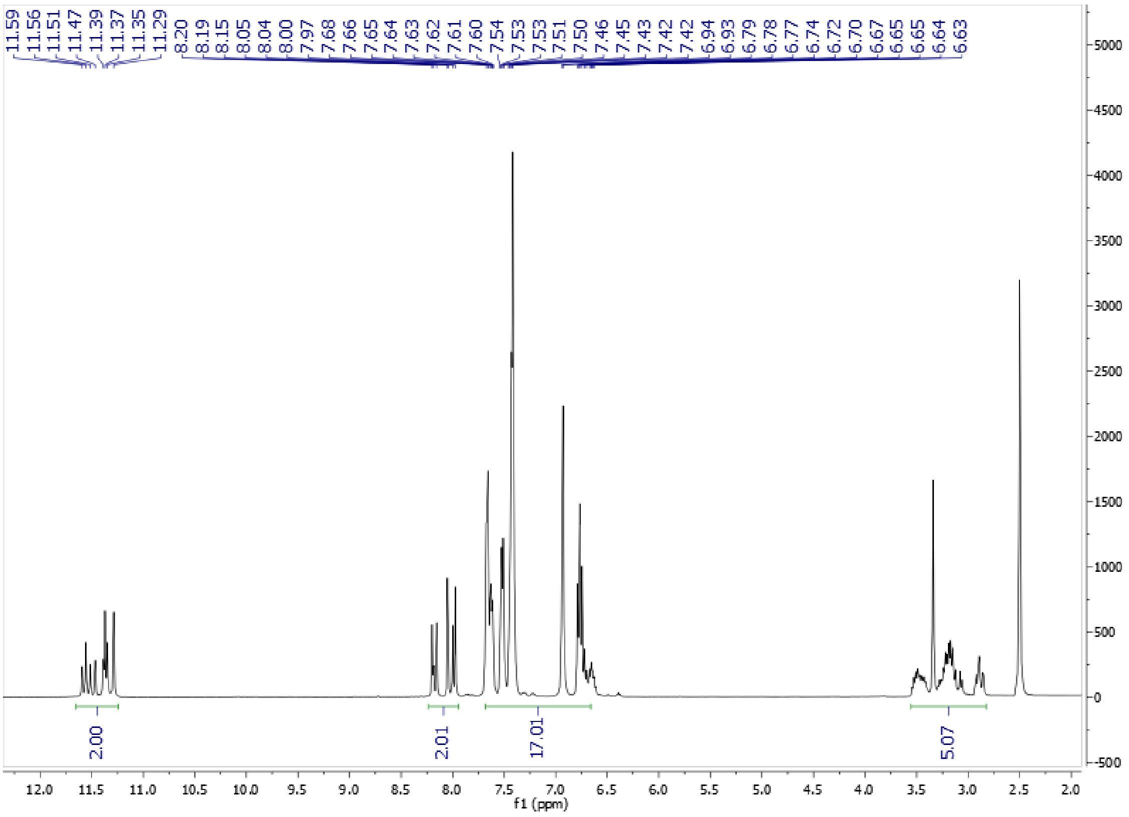

Figure S19. ^{1}H NMR of compound **6a** at 400 MHz (DMSO-*d*6)

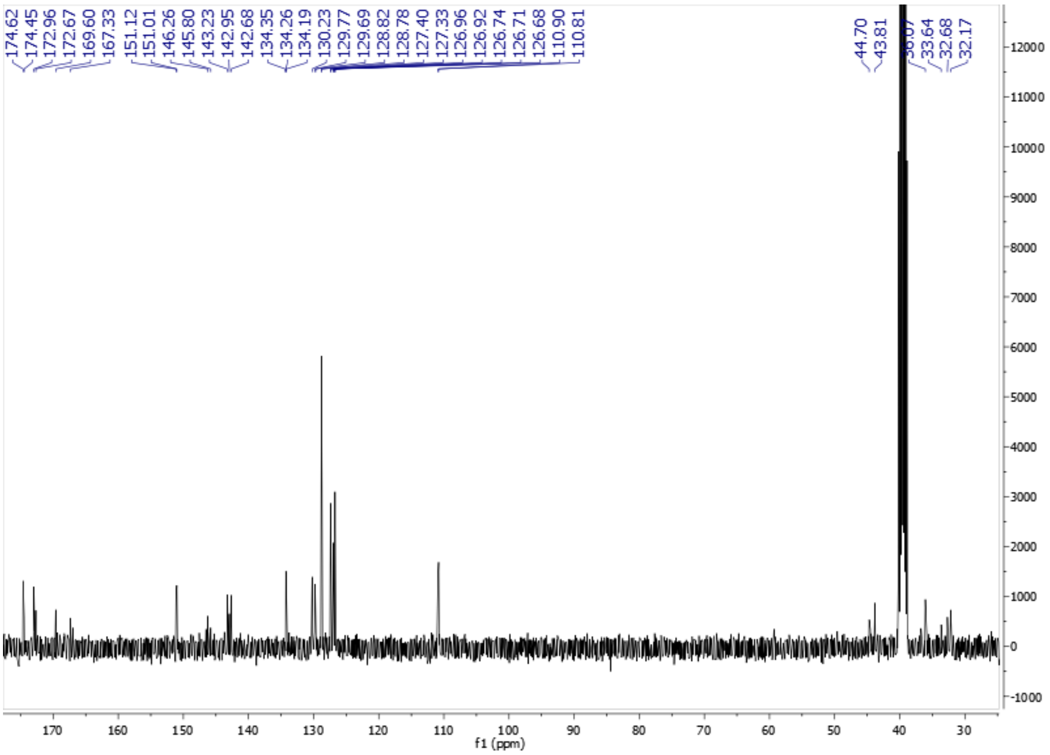

Figure S20. ^{13}C NMR spectrum of compound **6a** at 101 MHz (DMSO-*d*6)

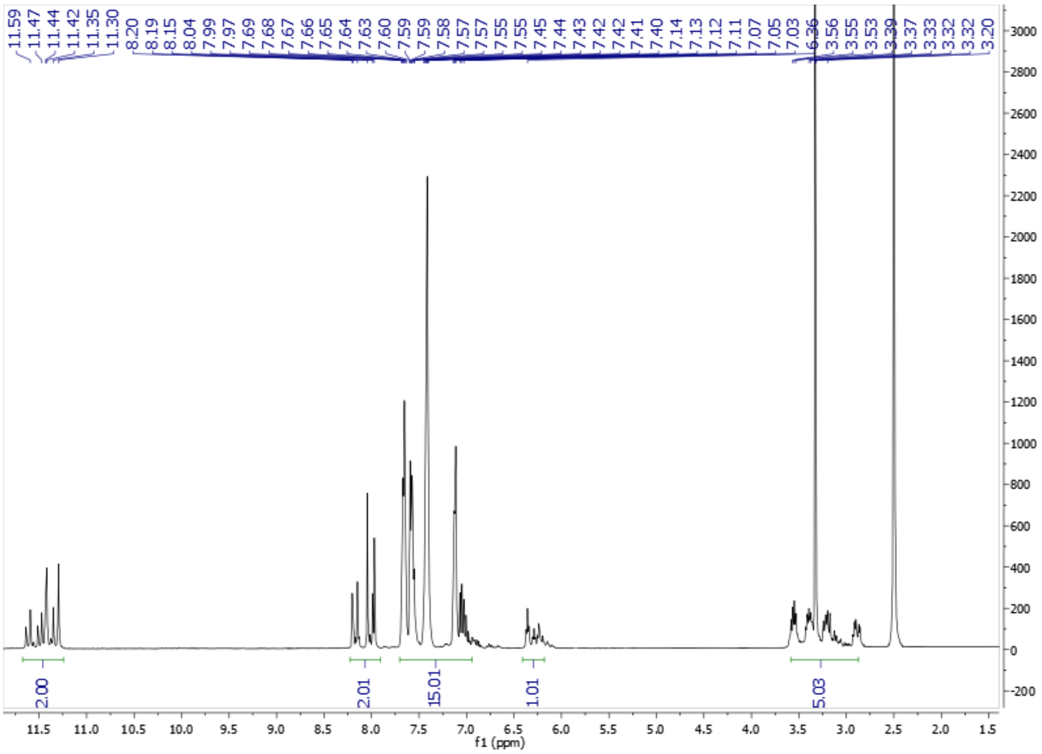

Figure S21. ^{1}H NMR of compound **6b** at 400 MHz (DMSO-*d*₆)

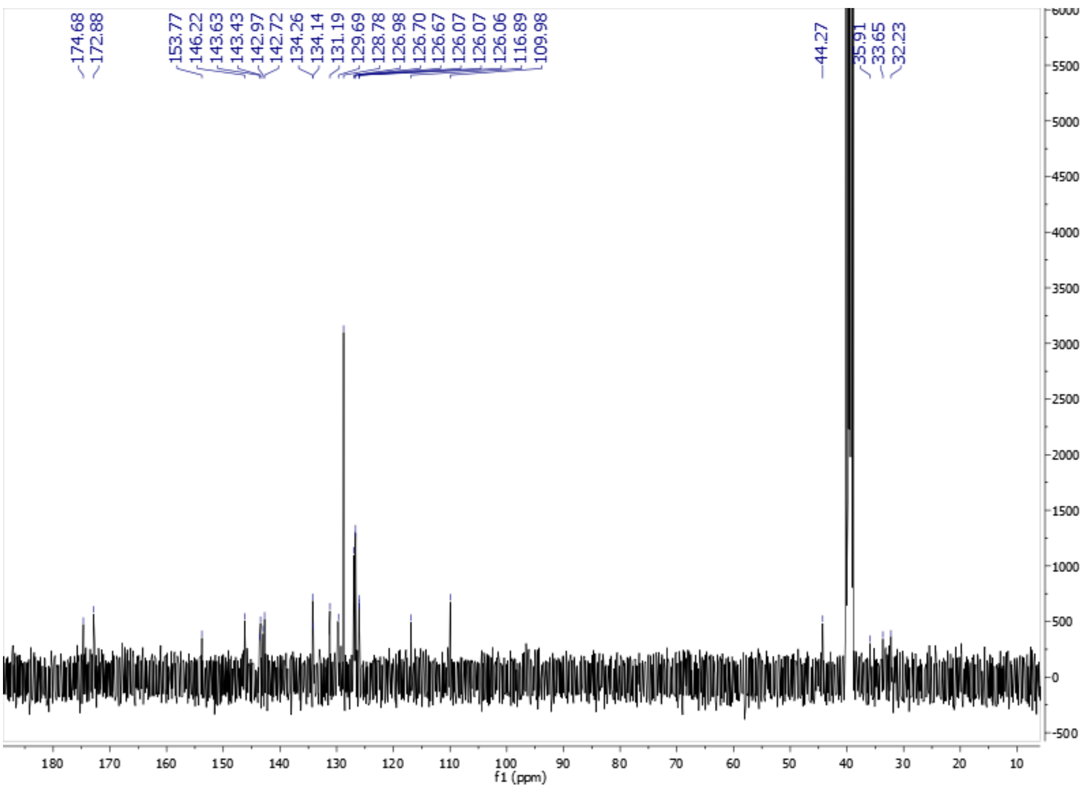

Figure S22. ^{13}C NMR spectrum of compound **6b** at 101 MHz (DMSO-*d*₆)

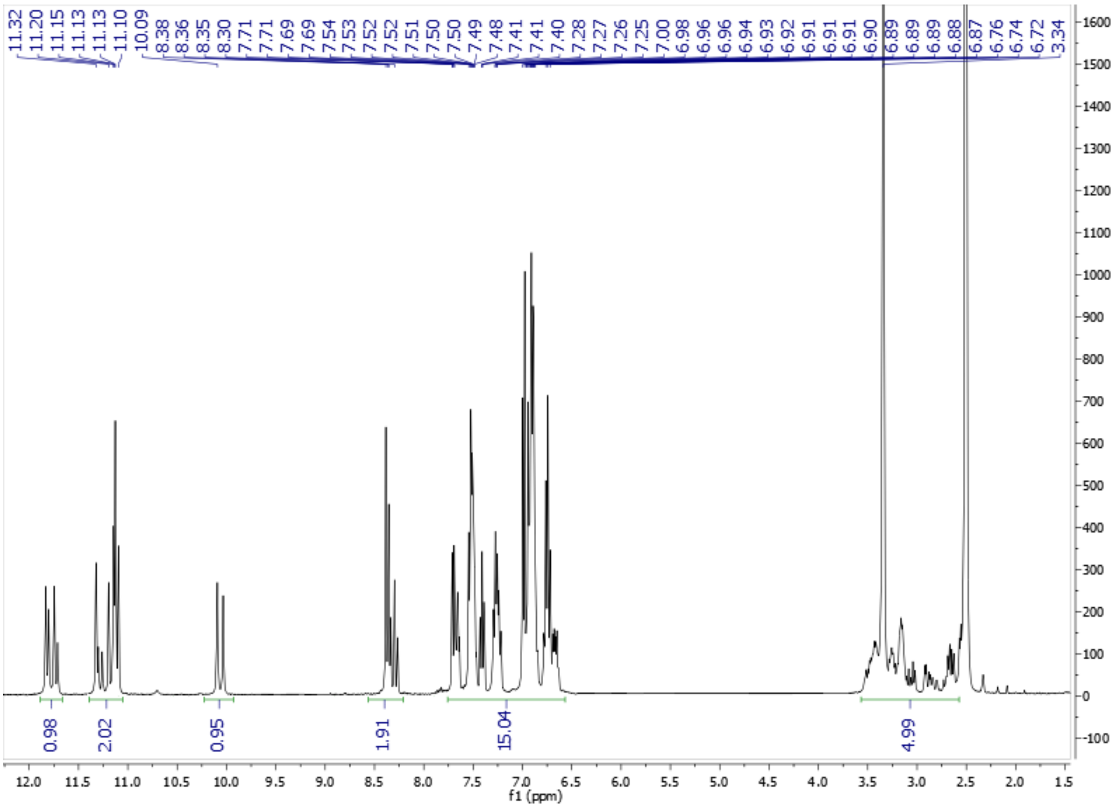

Figure S23. ^{1}H NMR of compound **7a** at 400 MHz (DMSO-d_6)

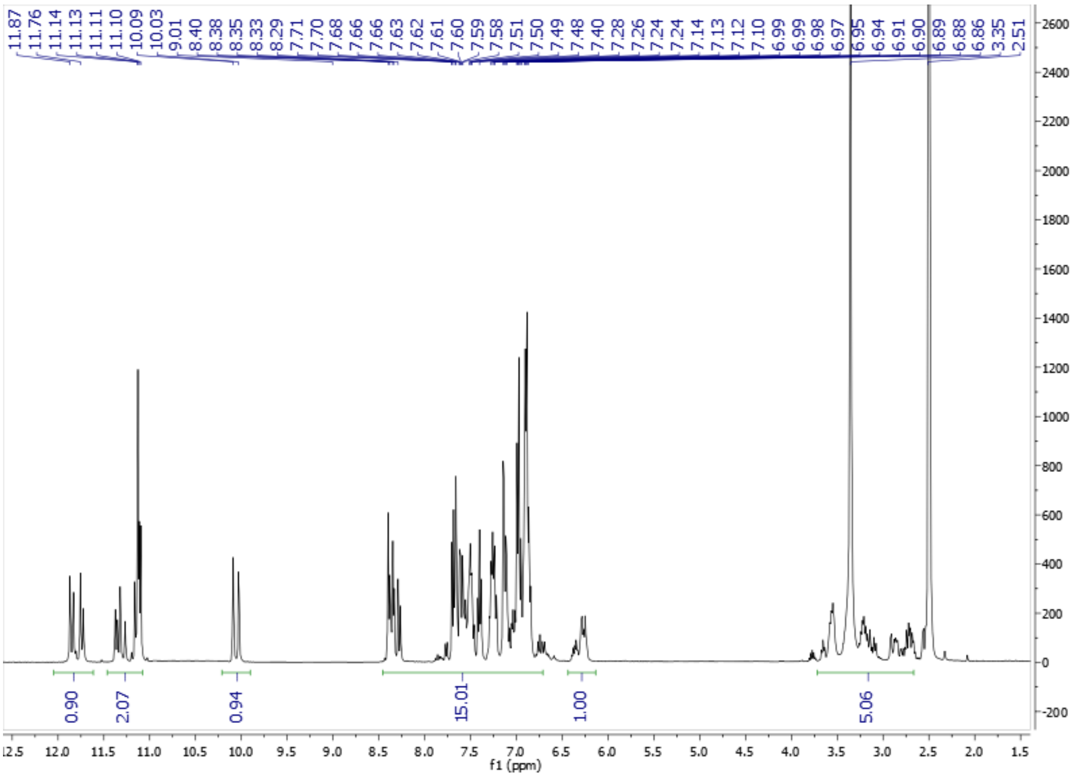

Figure S24. ^{1}H NMR of compound **7b** at 400 MHz (DMSO-d_6)

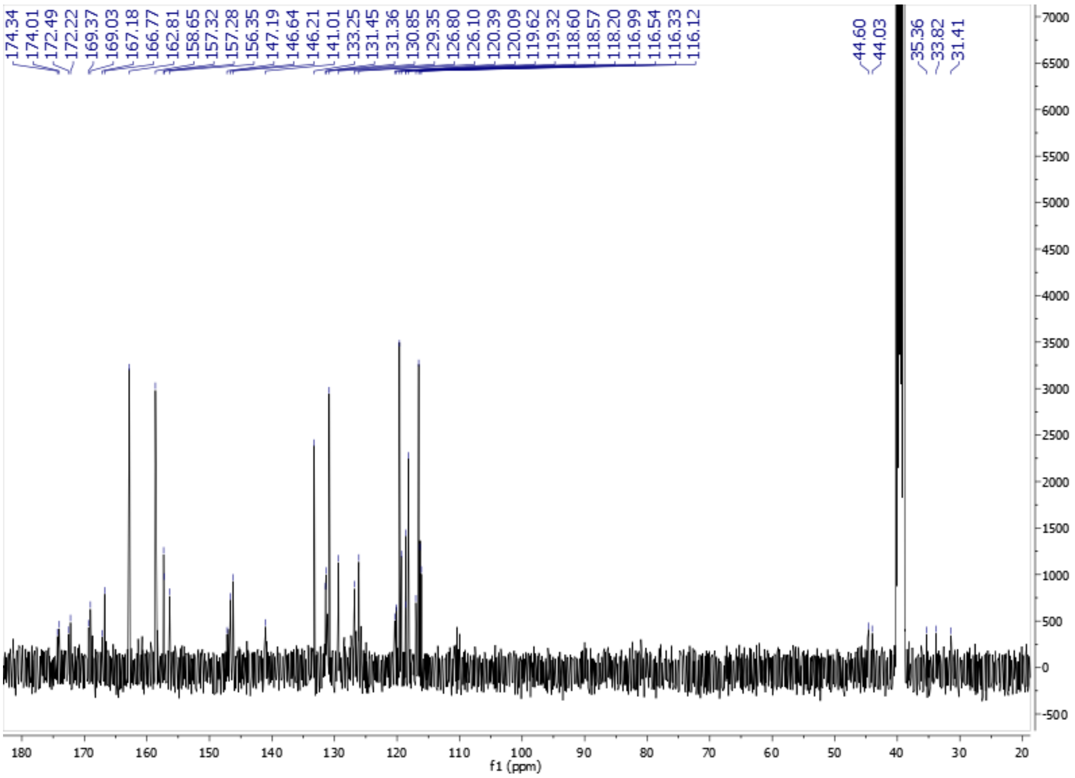

Figure S25. ^{13}C NMR spectrum of compound **7b** at 101 MHz (DMSO-d_6)

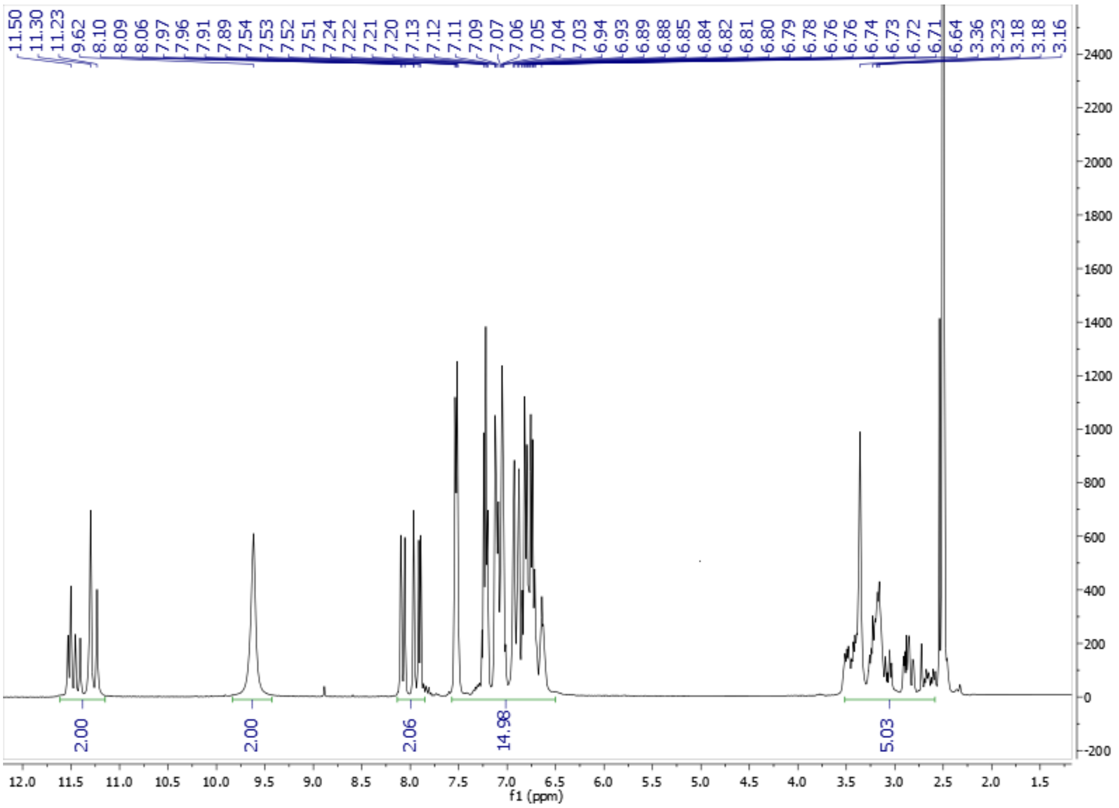

Figure S26. ^{1}H NMR of compound **8a** at 400 MHz (DMSO-*d*$_6$)

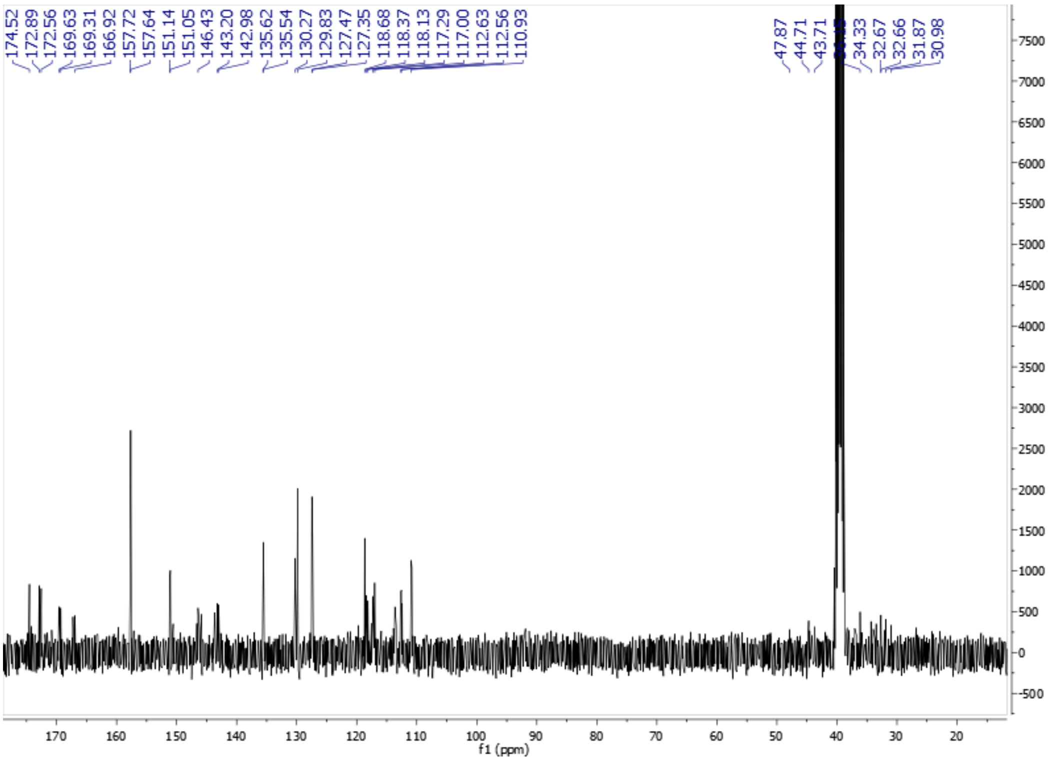

Figure S27. ^{13}C NMR spectrum of compound **8a** at 101 MHz (DMSO-*d*$_6$)

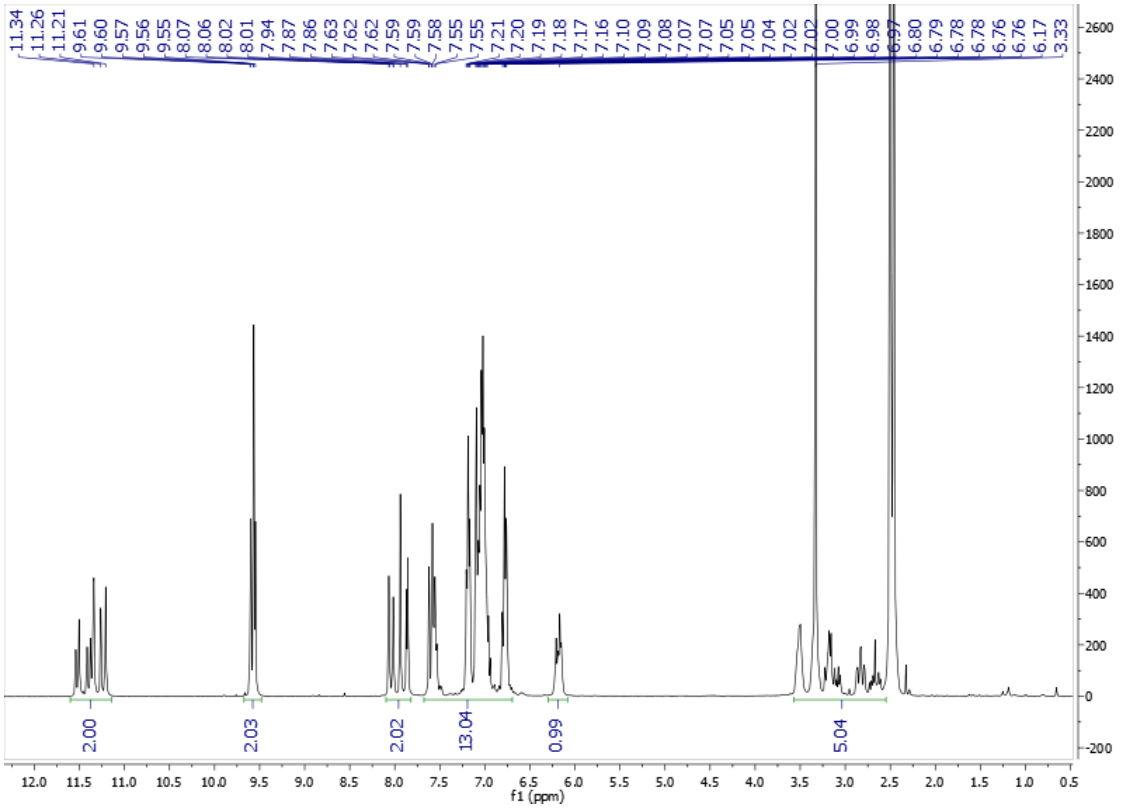

Figure S28. ^{1}H NMR of compound **8b** at 400 MHz (DMSO-d_6)

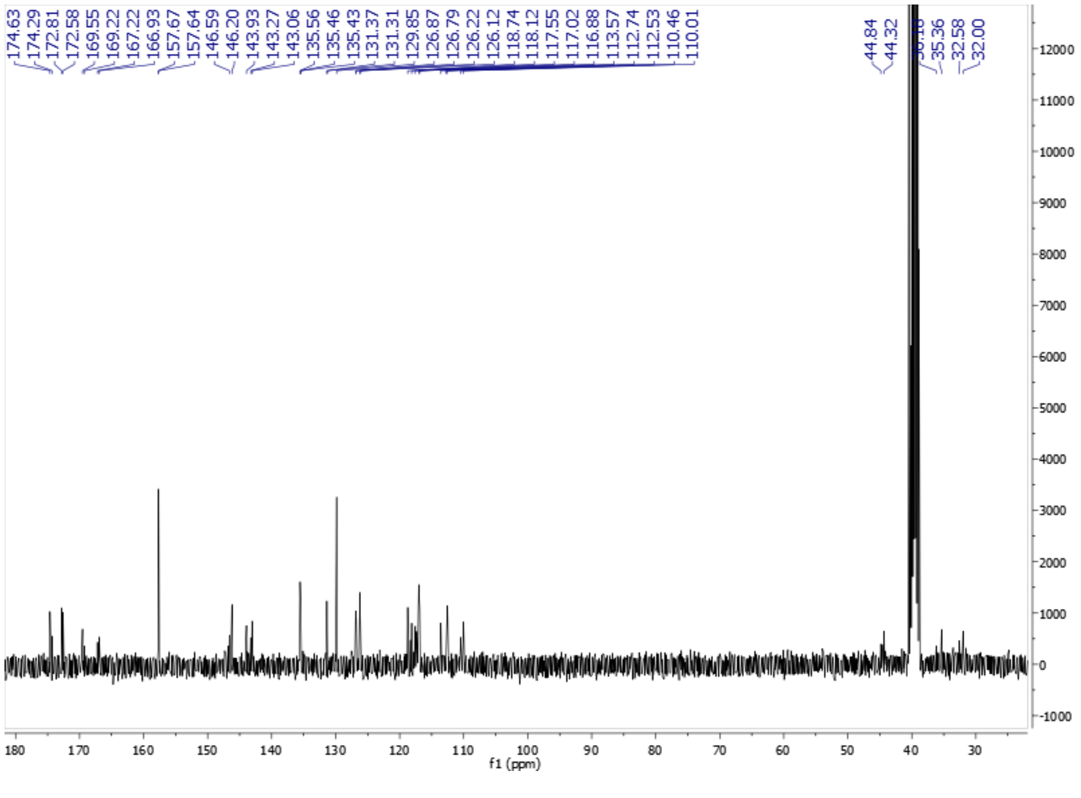

Figure S29. ^{13}C NMR spectrum of compound **8b** at 101 MHz (DMSO-d_6)

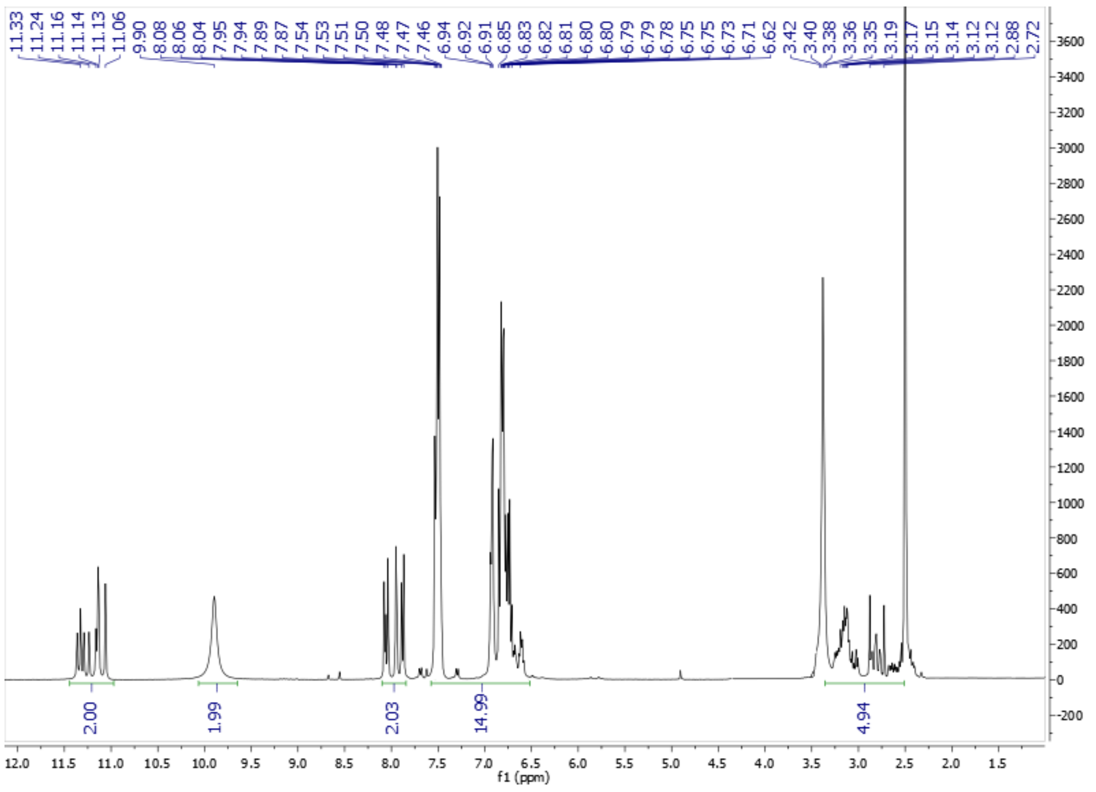

Figure S30. ^{1}H NMR of compound **9a** at 400 MHz (DMSO-*d*6)

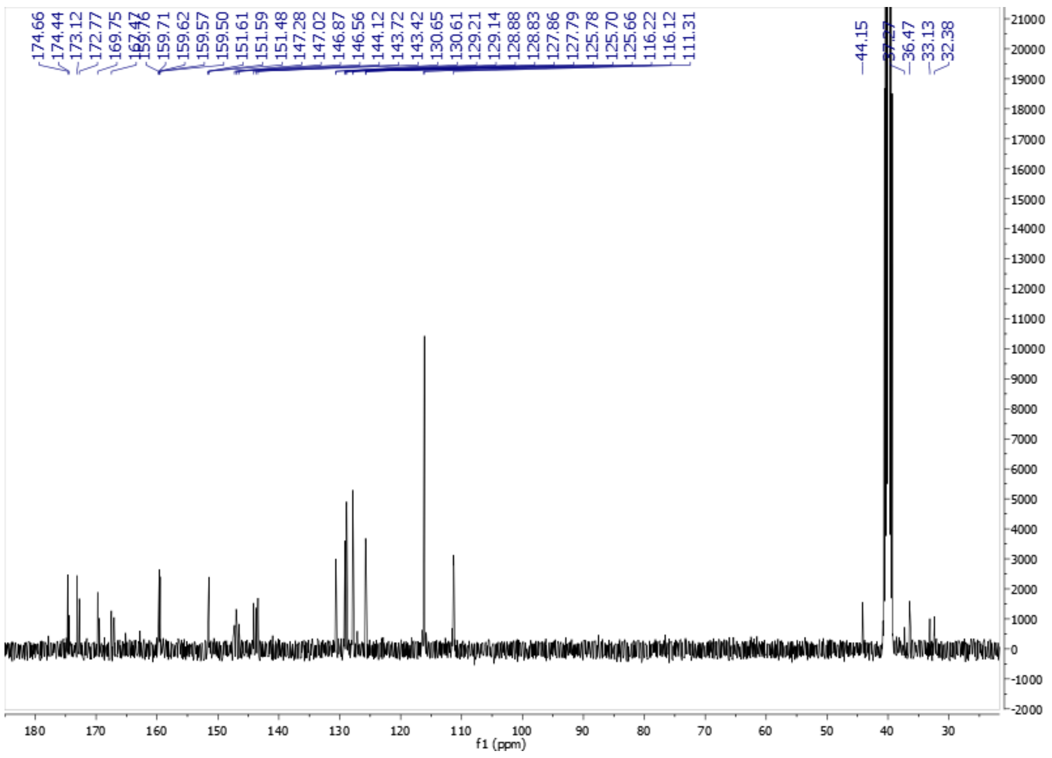

Figure S31. ^{13}C NMR spectrum of compound **9a** at 101 MHz (DMSO-*d*6)

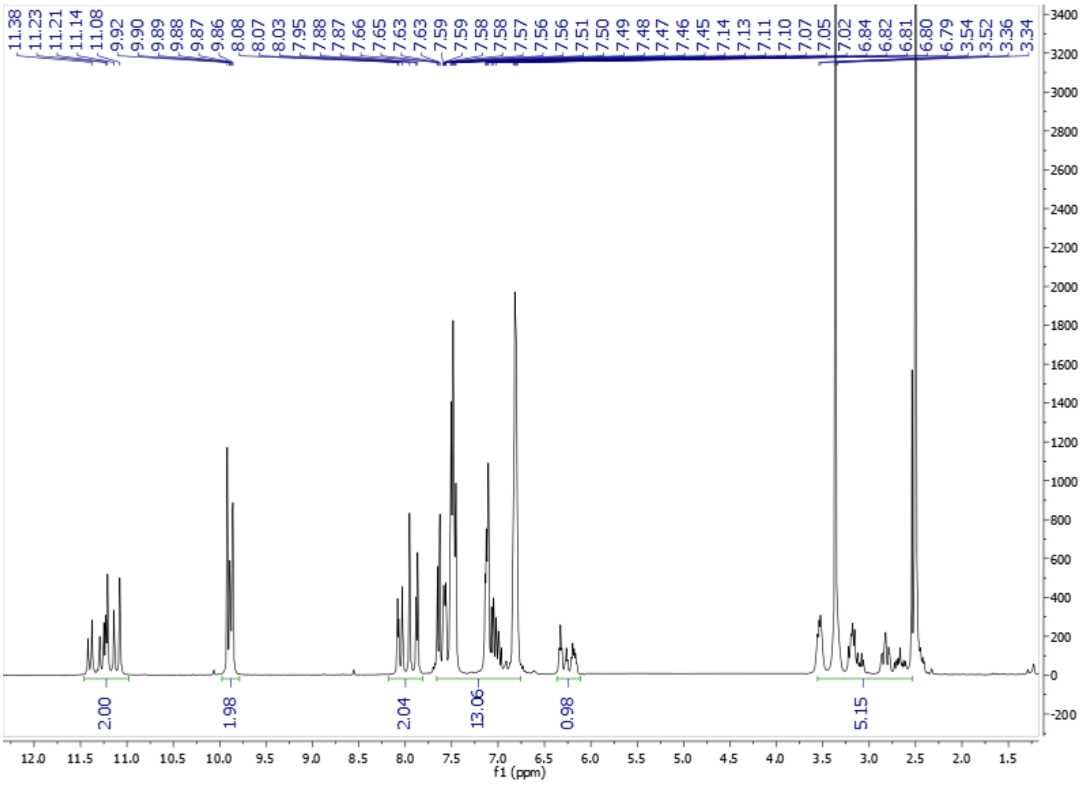

Figure S32. ^{1}H NMR of compound **9b** at 400 MHz (DMSO-d_6)

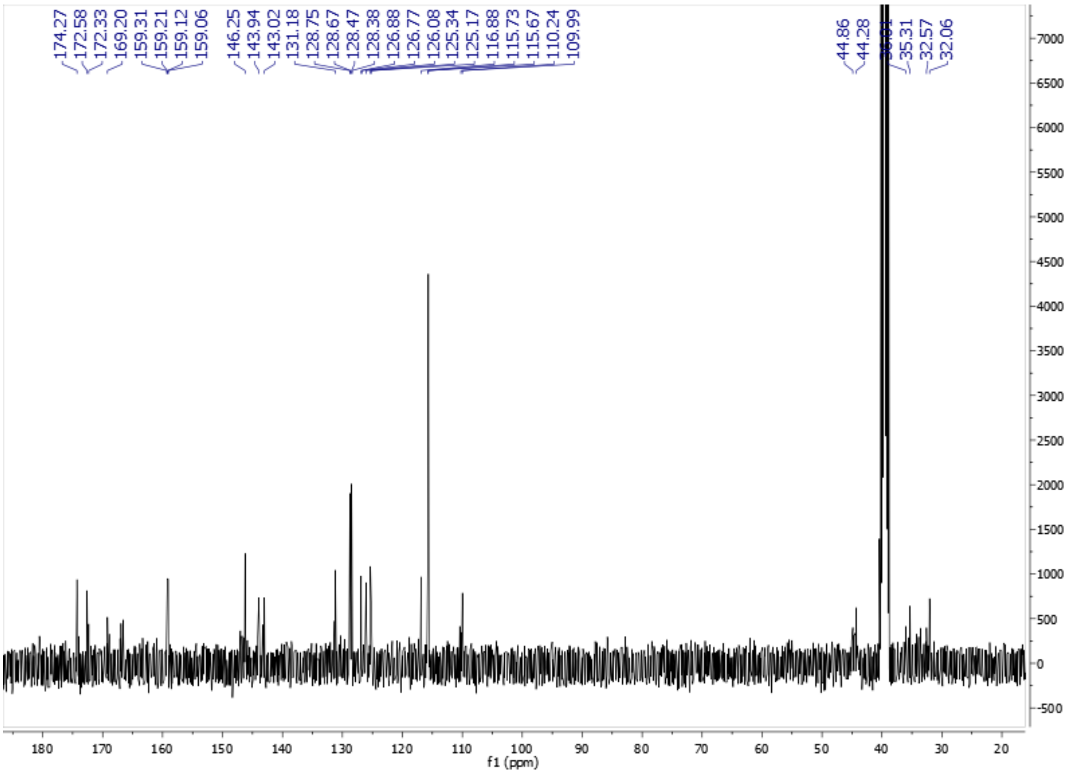

Figure S33. ^{13}C NMR spectrum of compound **9b** at 101 MHz (DMSO-d_6)

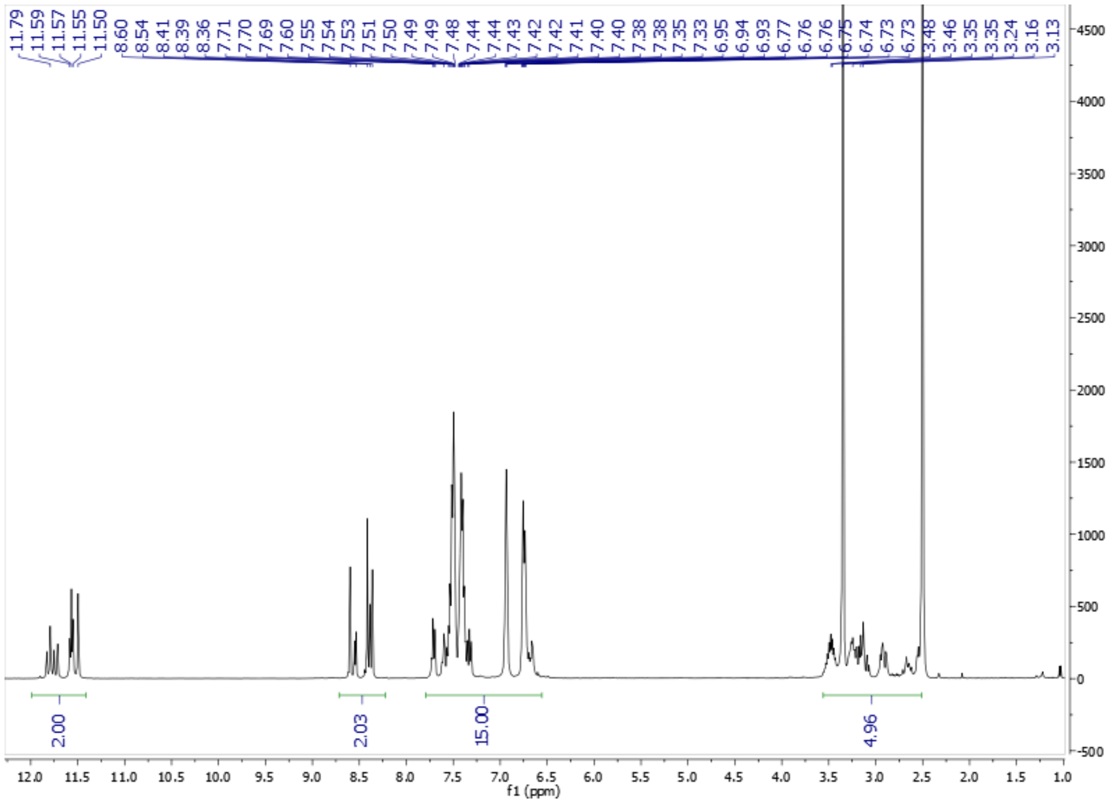

Figure S34. ^{1}H NMR of compound **10a** at 400 MHz (DMSO-d_6)

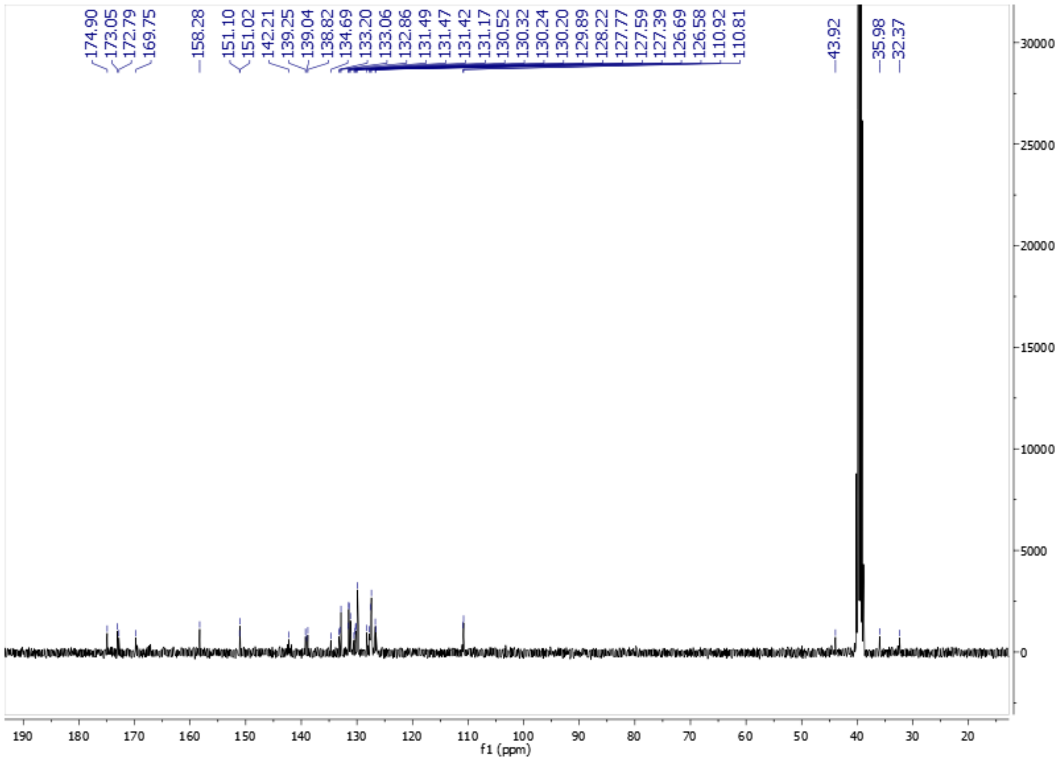

Figure S35. ^{13}C NMR spectrum of compound **10a** at 101 MHz (DMSO-d_6)

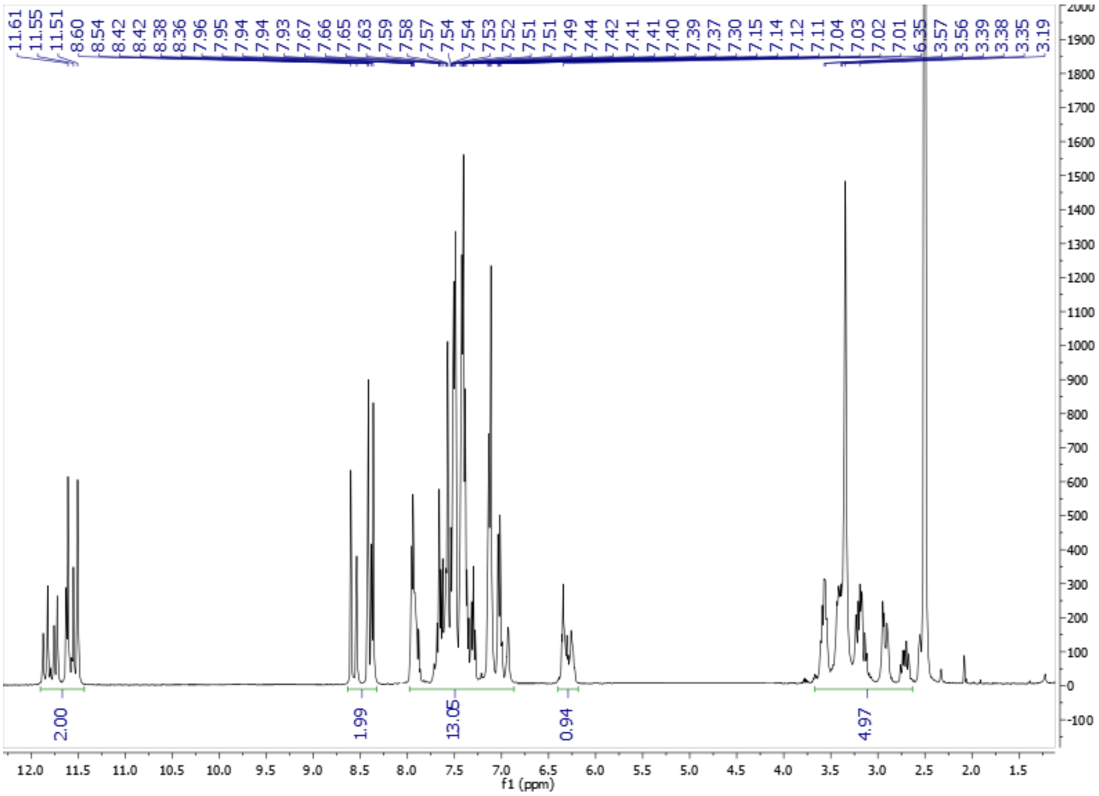

Figure S36. ^{1}H NMR of compound **10b** at 400 MHz (DMSO-d_6)

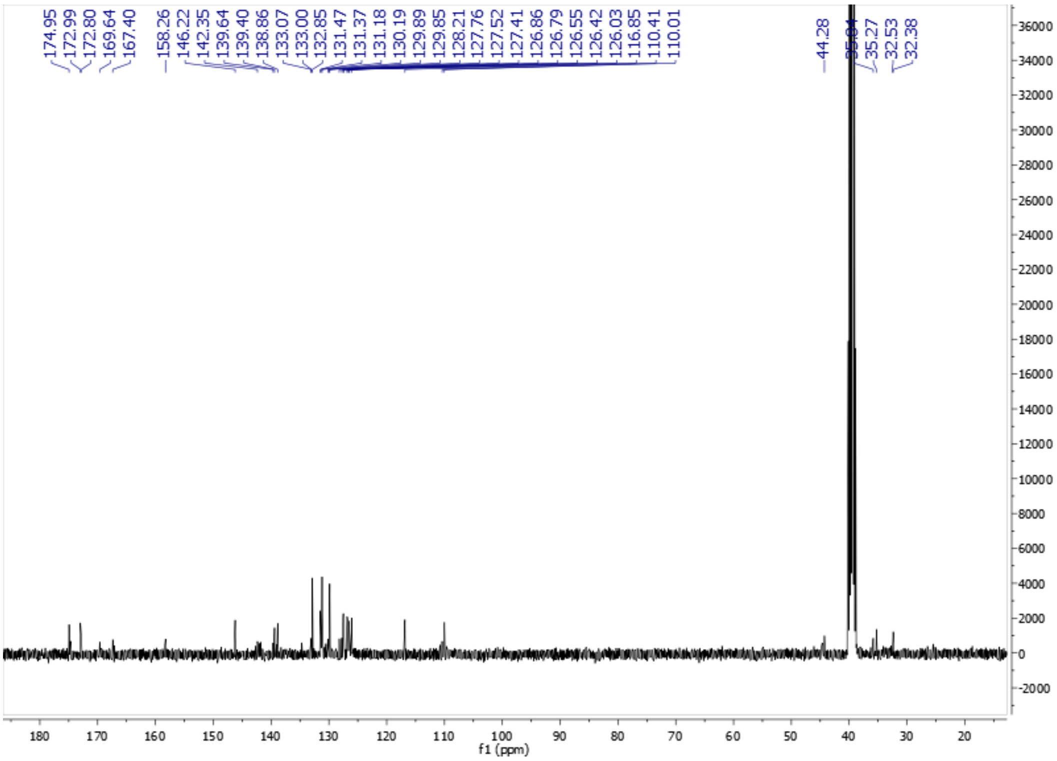

Figure S37. ^{13}C NMR spectrum of compound **10b** at 101 MHz (DMSO-d_6)

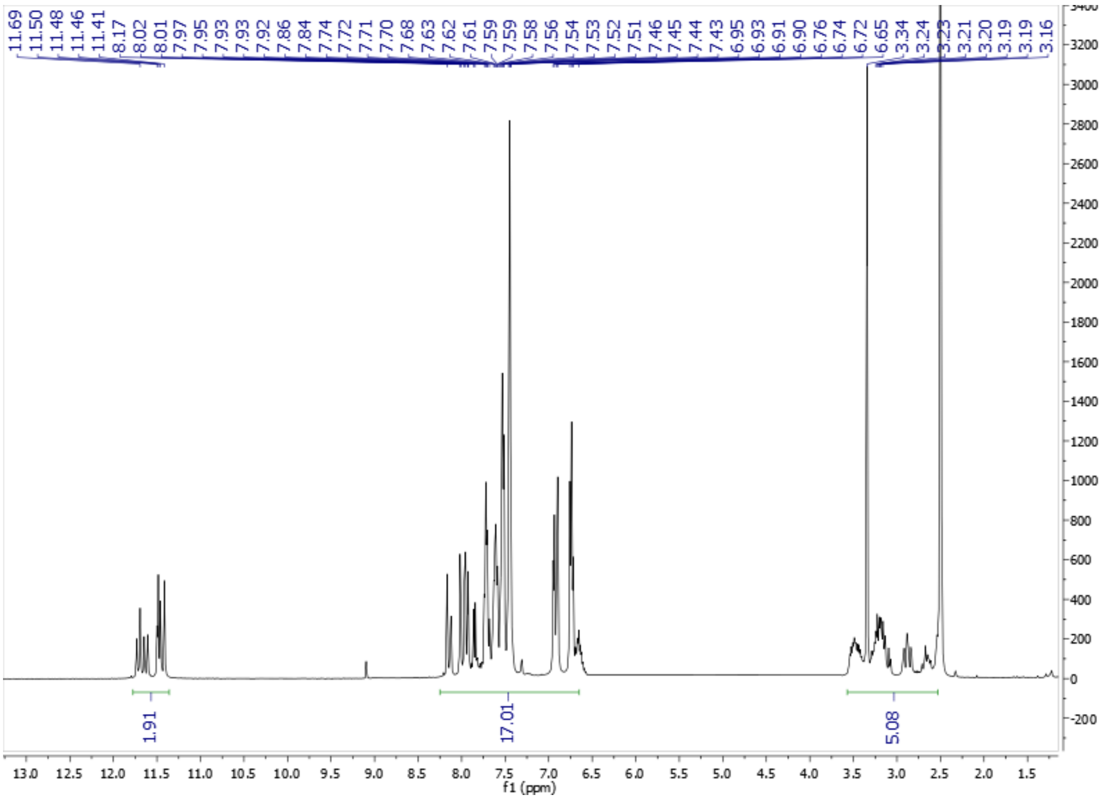

Figure S38. ^{1}H NMR of compound **11a** at 400 MHz (DMSO-d_6)

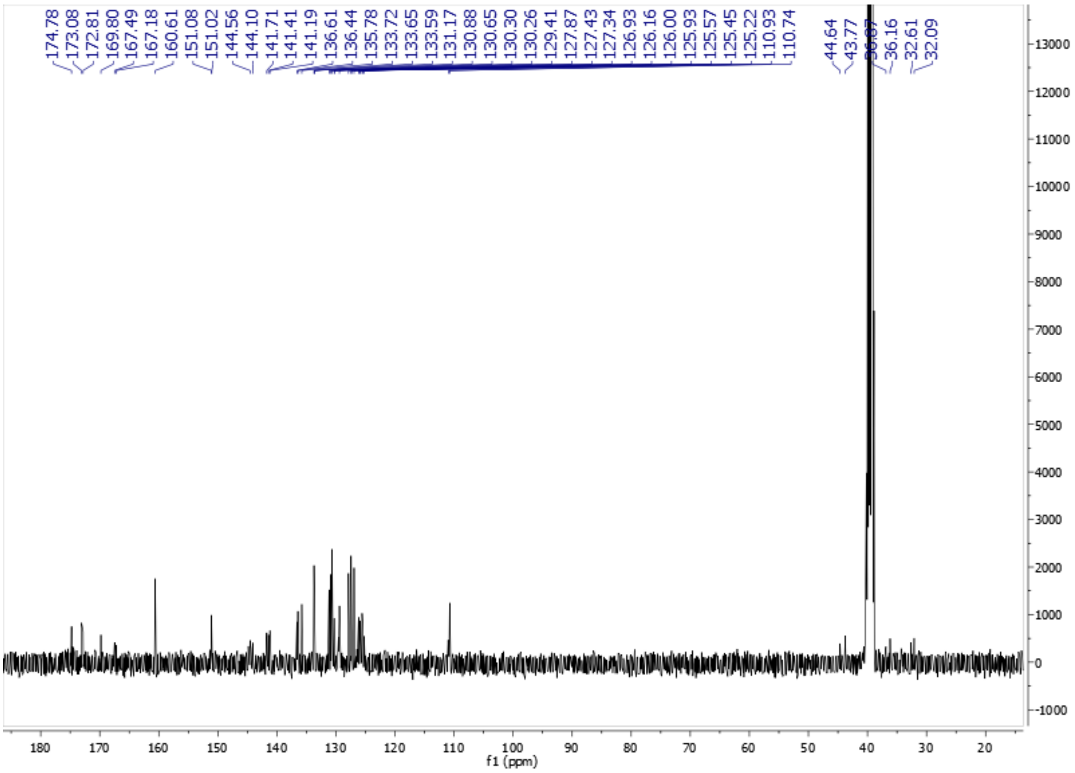

Figure S39. ^{13}C NMR spectrum of compound **11a** at 101 MHz (DMSO-d_6)

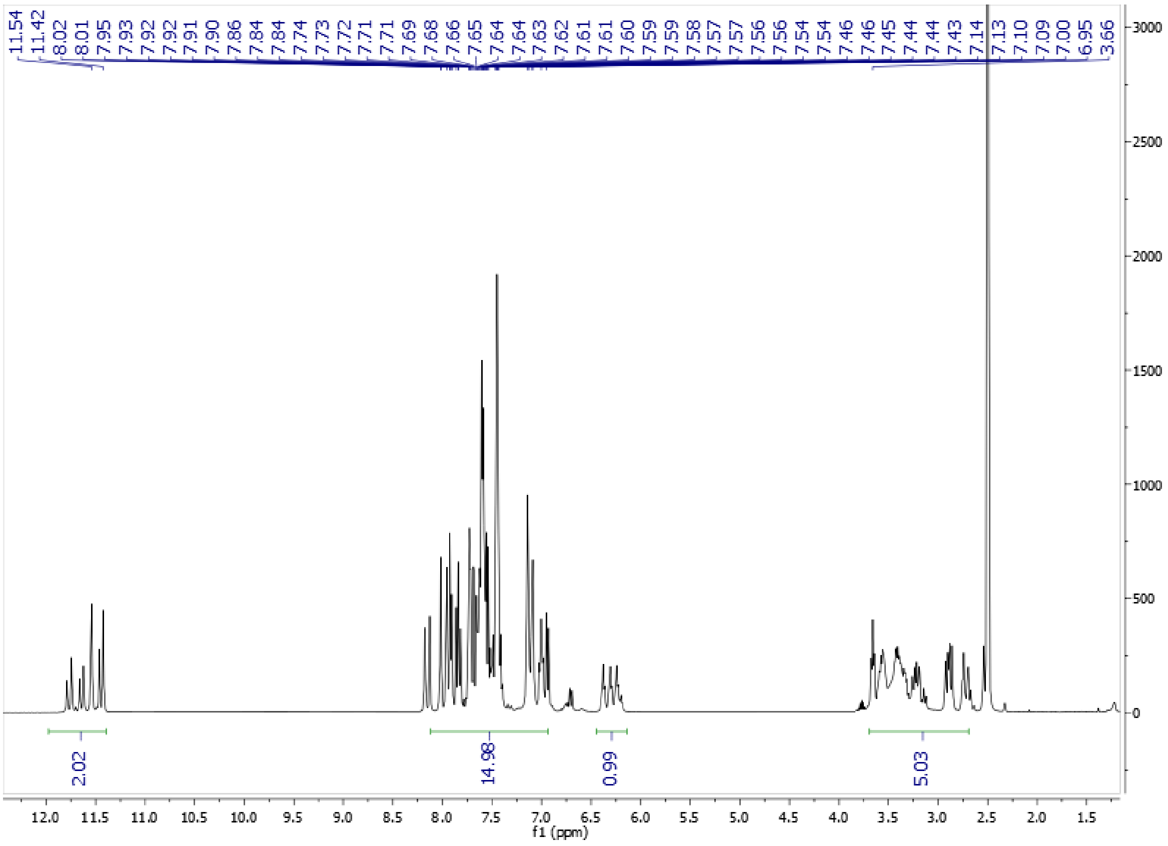

Figure S40. ^{1}H NMR of compound **11b** at 400 MHz (DMSO-d_6)

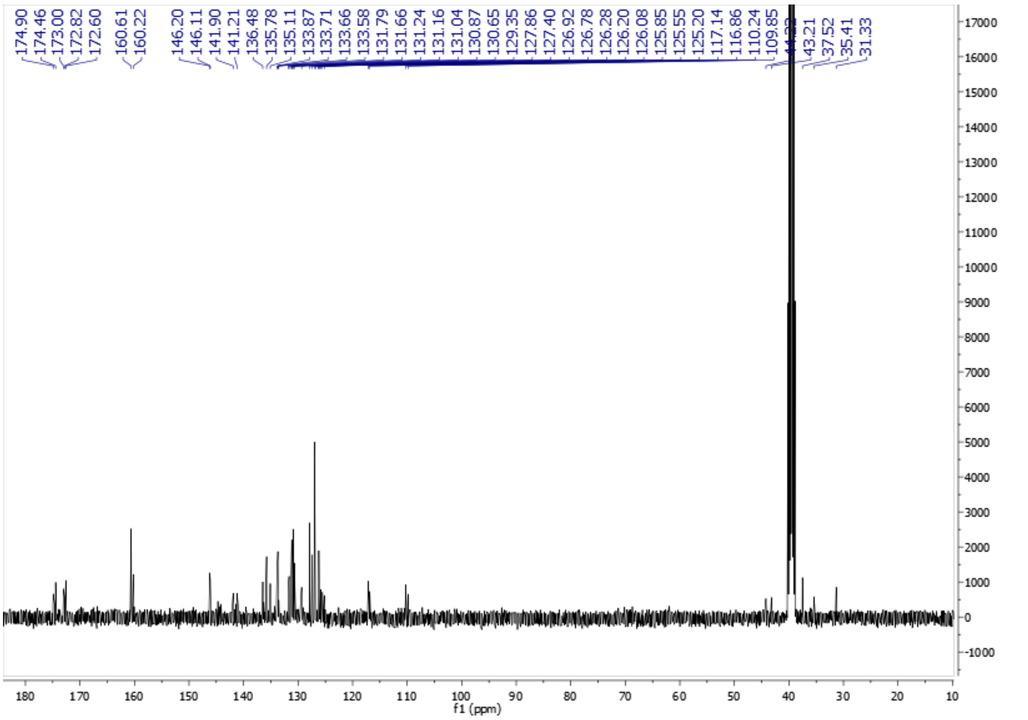

Figure S41. ^{13}C NMR spectrum of compound **11b** at 101 MHz (DMSO-d_6)

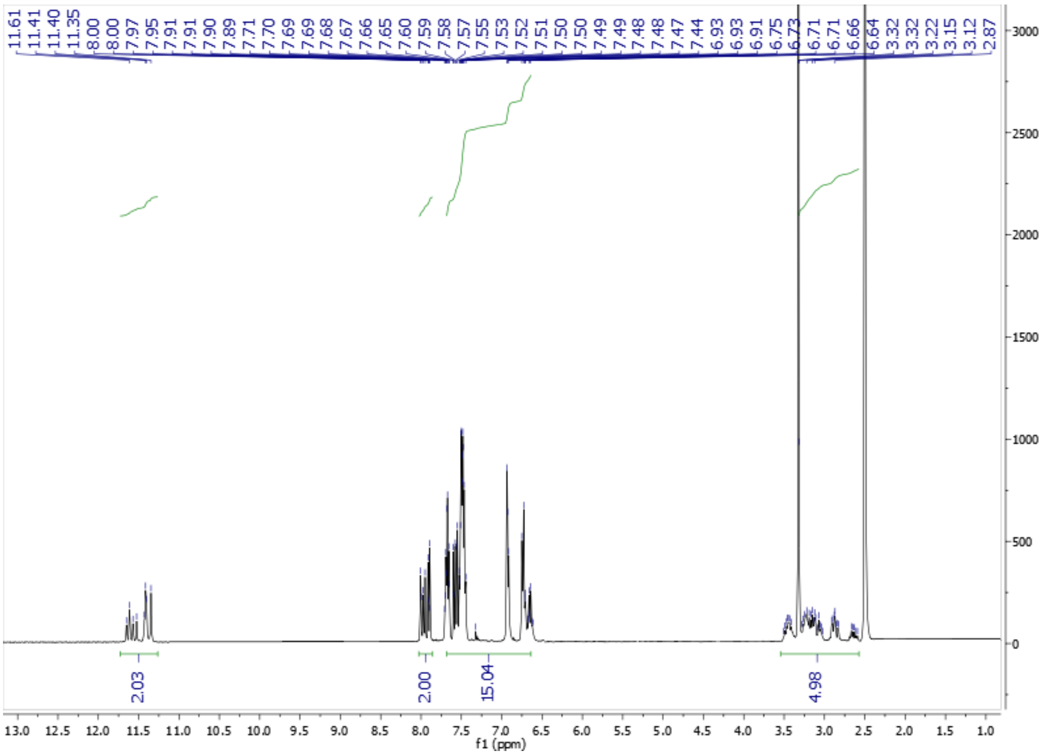

Figure S42. ^{1}H NMR of compound **12a** at 400 MHz (DMSO-d_6)

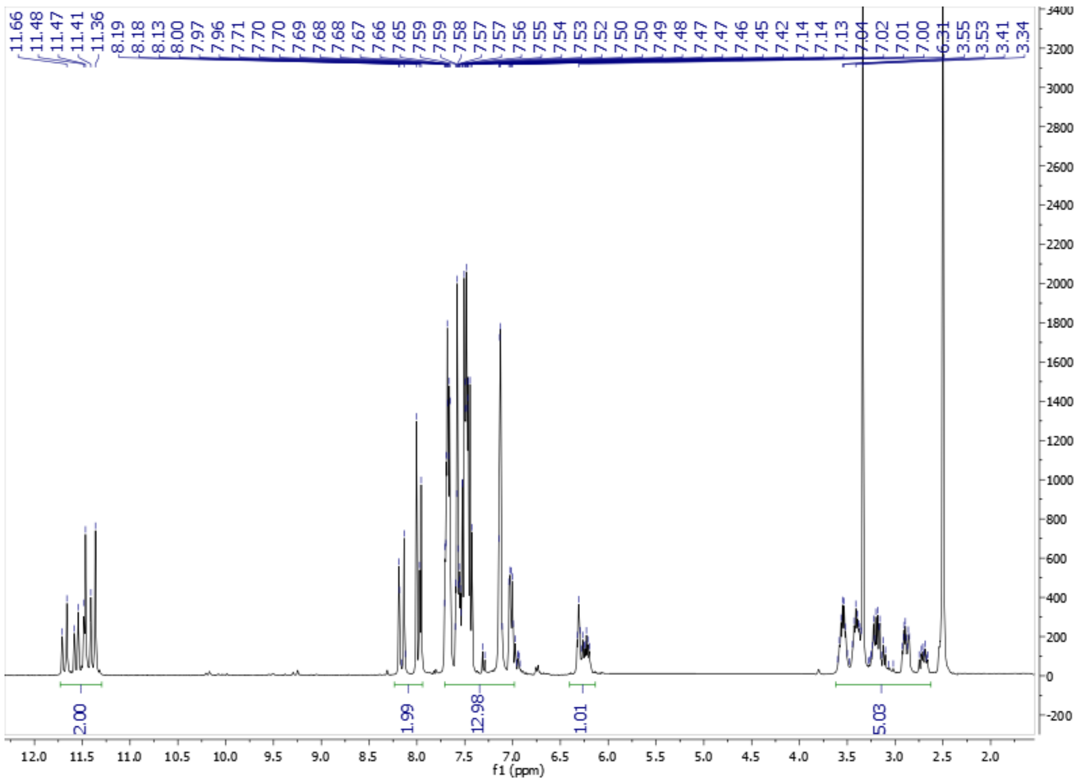

Figure S43. ^{1}H NMR of compound **12b** at 400 MHz (DMSO-d_6)

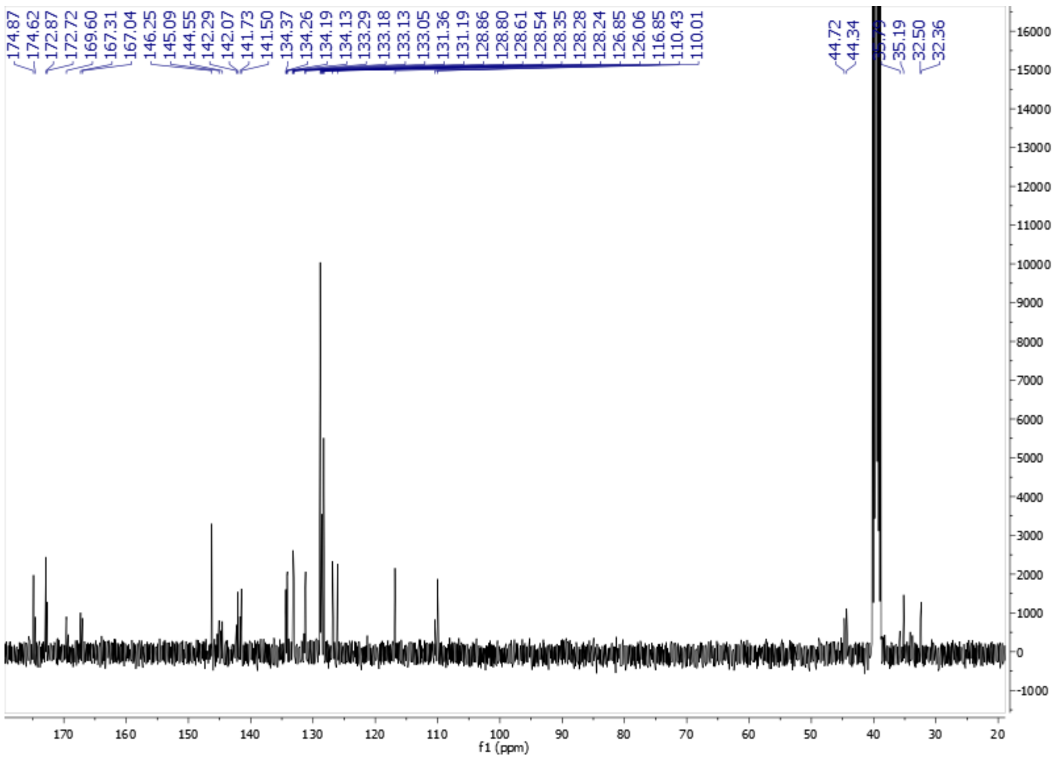

Figure S44. ^{13}C NMR spectrum of compound **12b** at 101 MHz (DMSO-d_6)

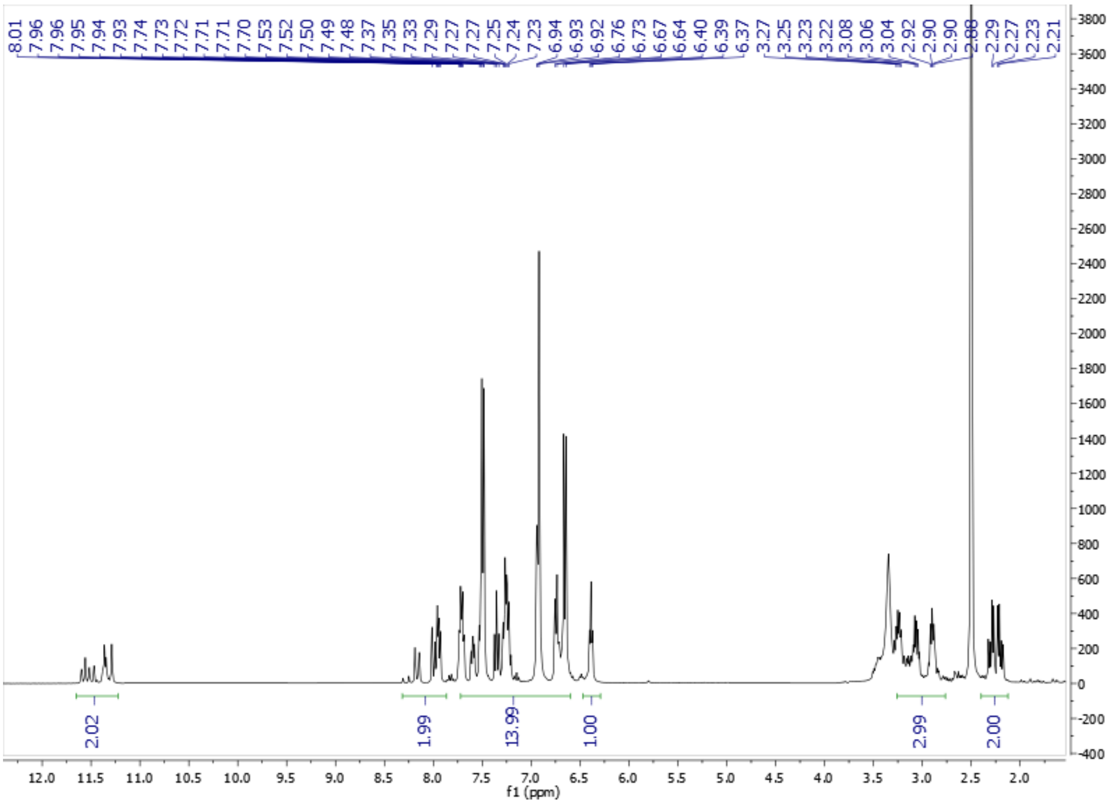

Figure S45. ^{1}H NMR of compound **13a** at 400 MHz (DMSO-d_6)

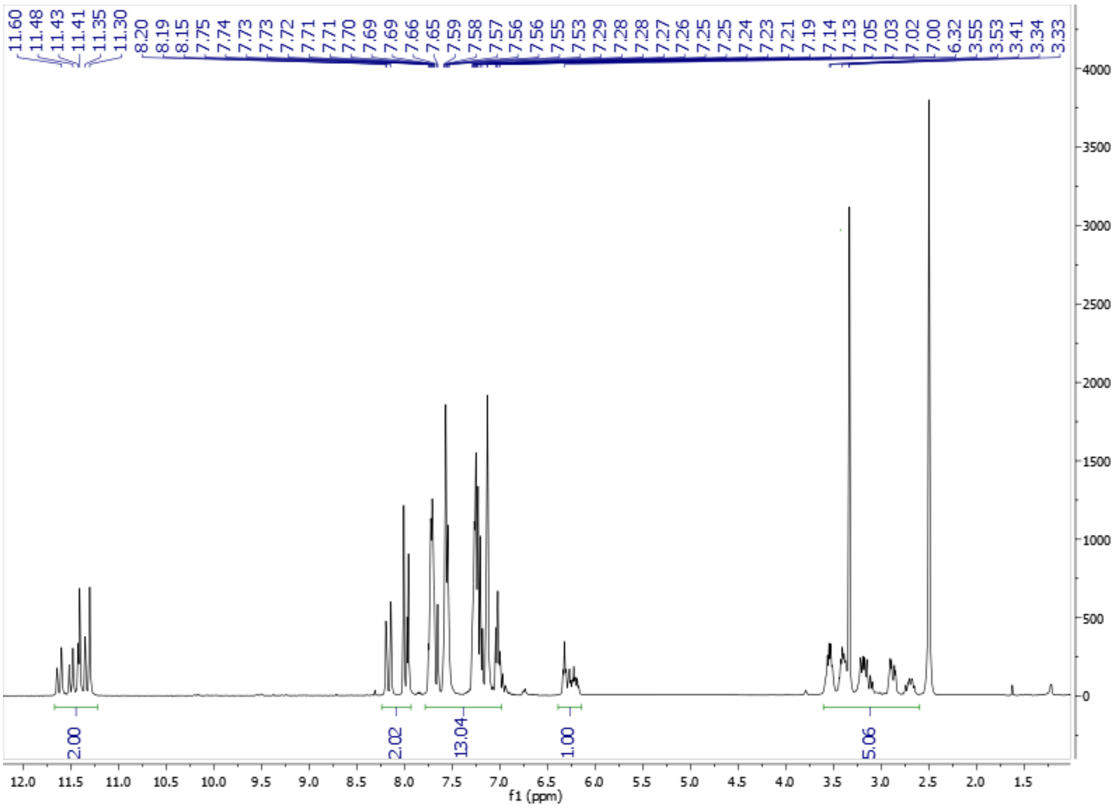

Figure S46. ^{1}H NMR of compound **13b** at 400 MHz (DMSO-d_6)

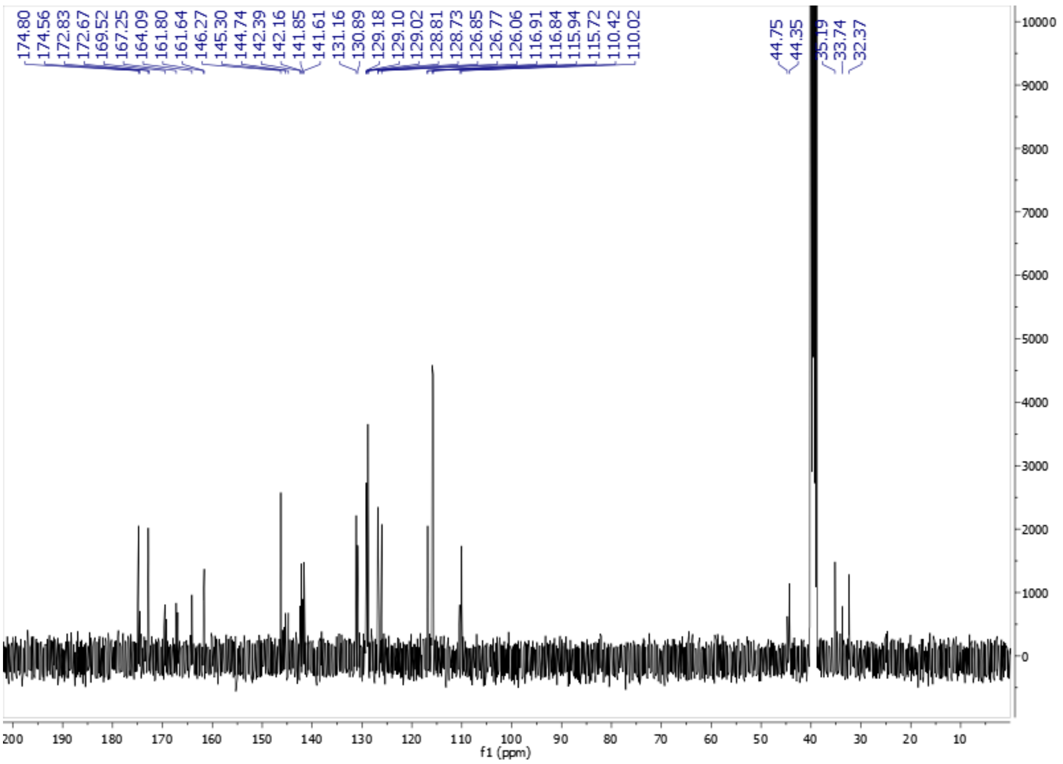

Figure S47. ^{13}C NMR spectrum of compound **13b** at 101 MHz (DMSO-d_6)

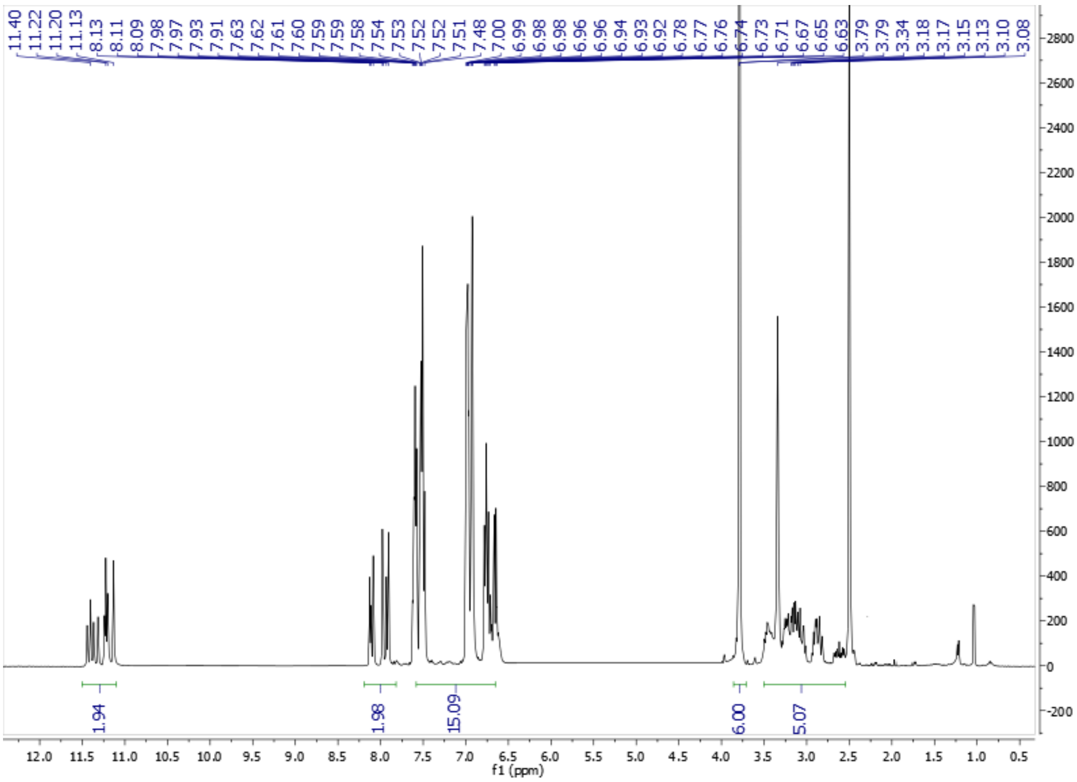

Figure S48. ^{1}H NMR of compound **14a** at 400 MHz (DMSO-*d*6)

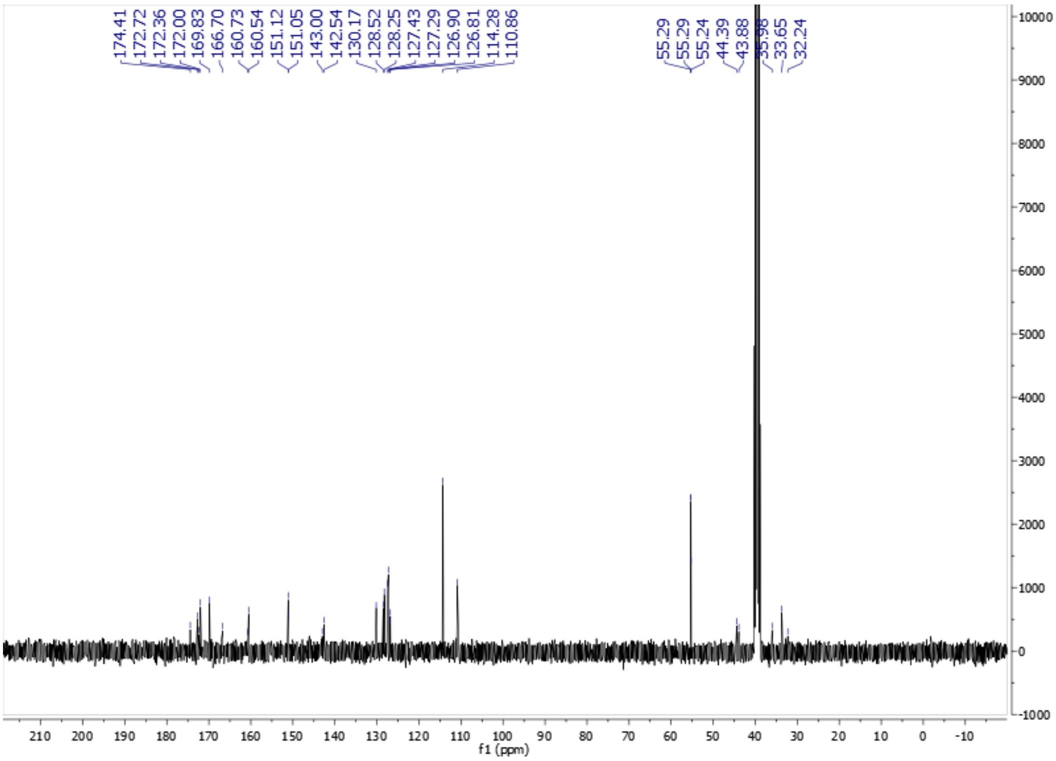

Figure S49. ^{13}C NMR spectrum of compound **14a** at 101 MHz (DMSO-*d*6)

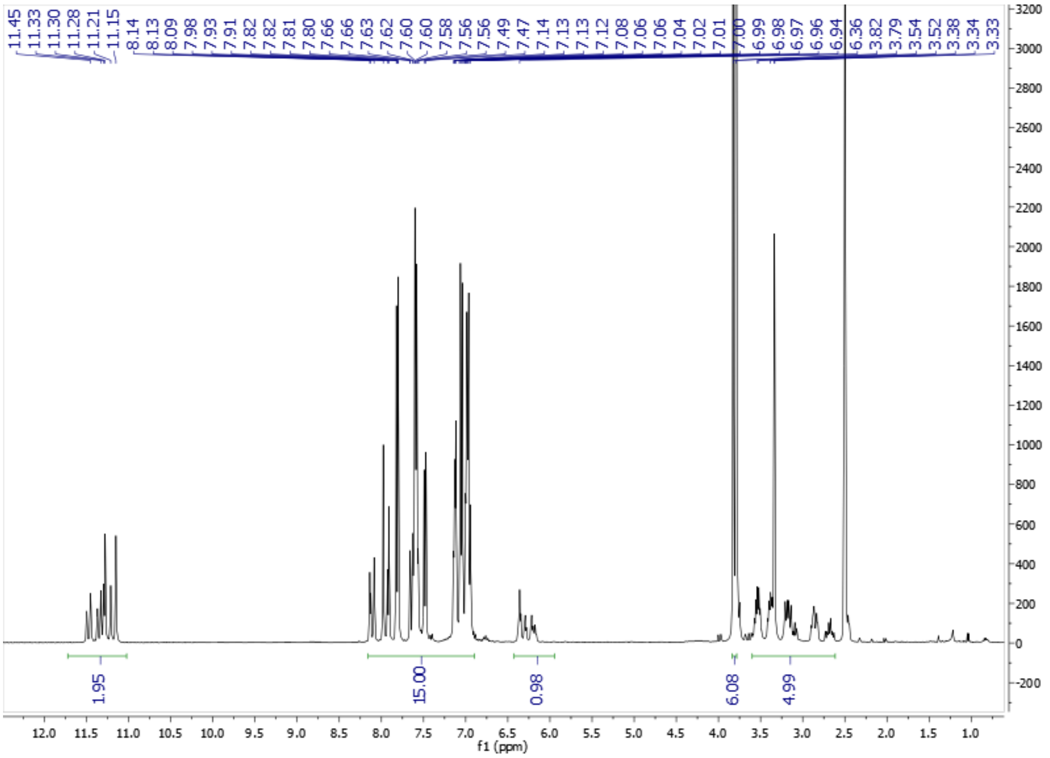

Figure S50. ^{1}H NMR of compound **14b** at 400 MHz (DMSO-d_6)

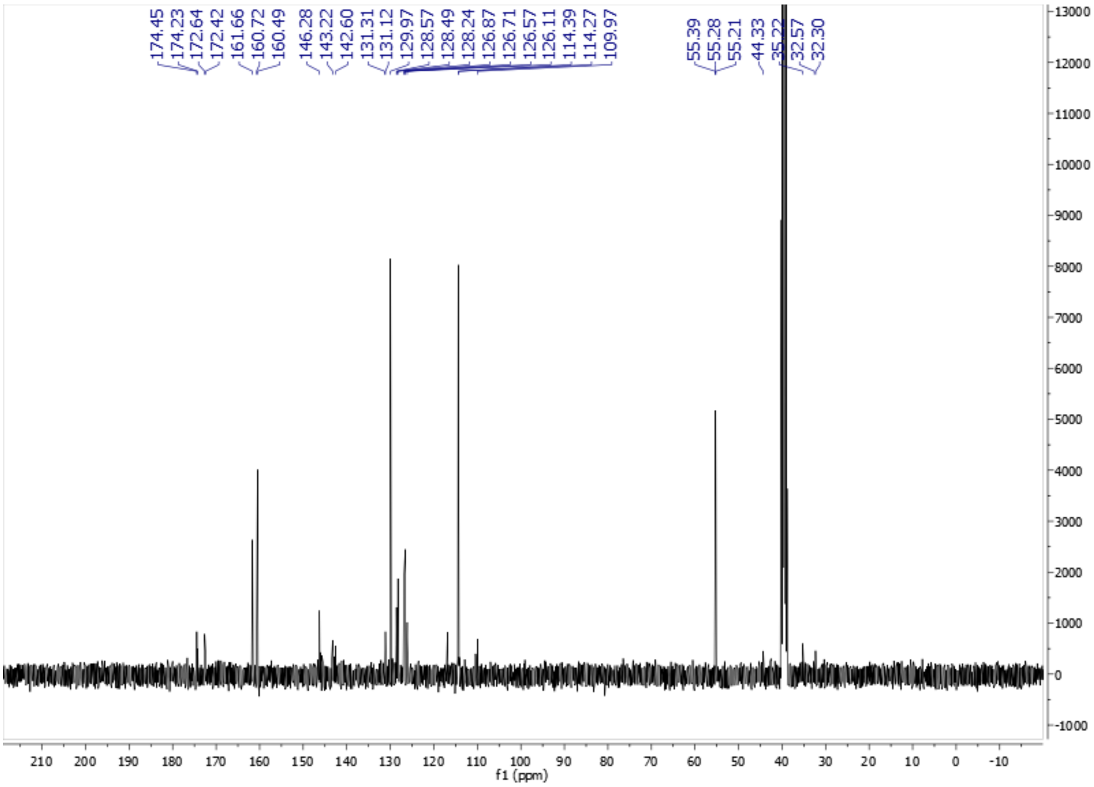

Figure S51. ^{13}C NMR spectrum of compound 1**4b** at 101 MHz (DMSO-d_6)

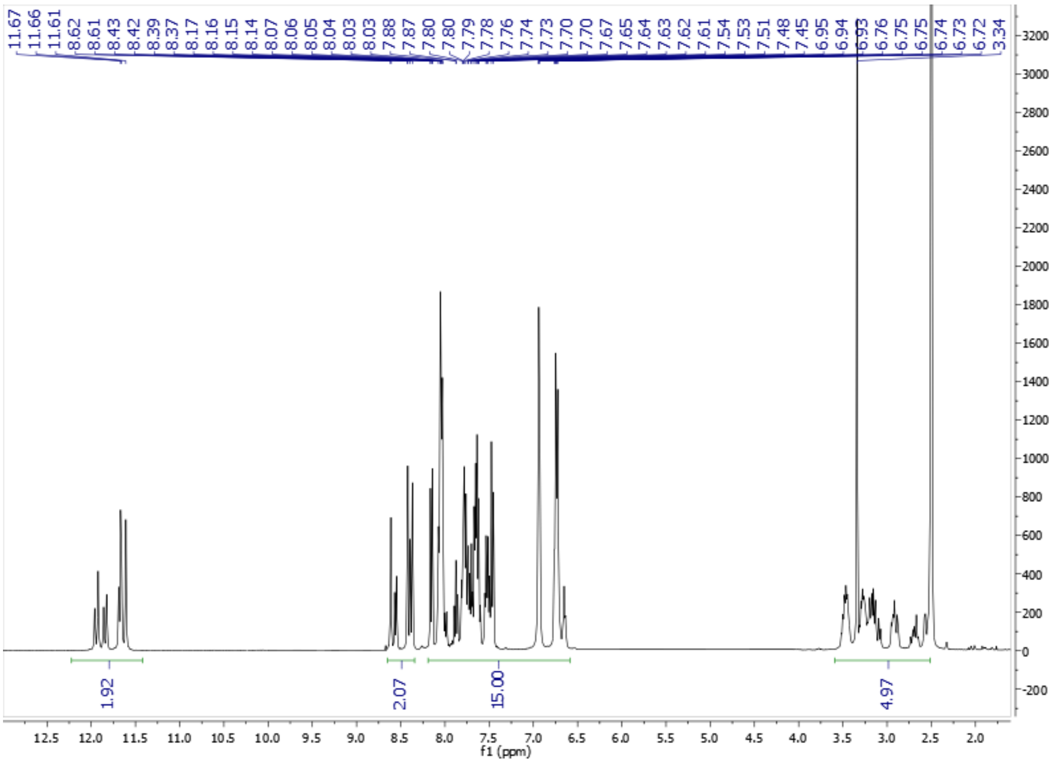

Figure S52. ¹H NMR of compound **15a** at 400 MHz (DMSO-*d*₆)

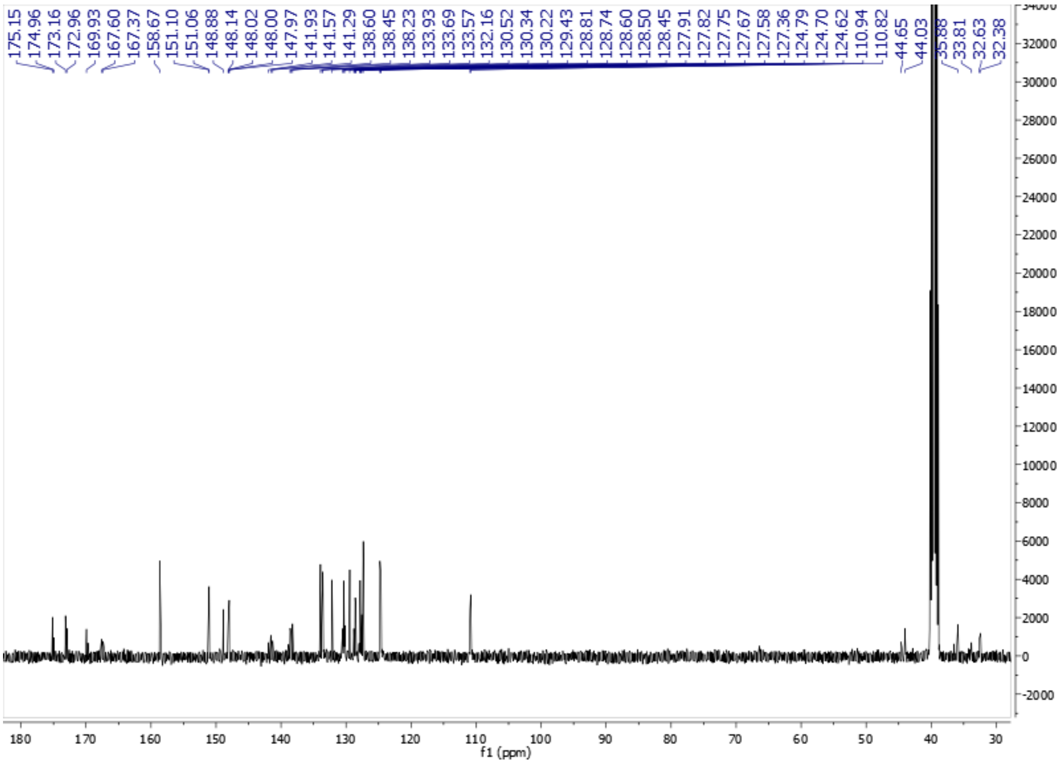

Figure S53. ¹³C NMR spectrum of compound **15a** at 101 MHz (DMSO-*d*₆)

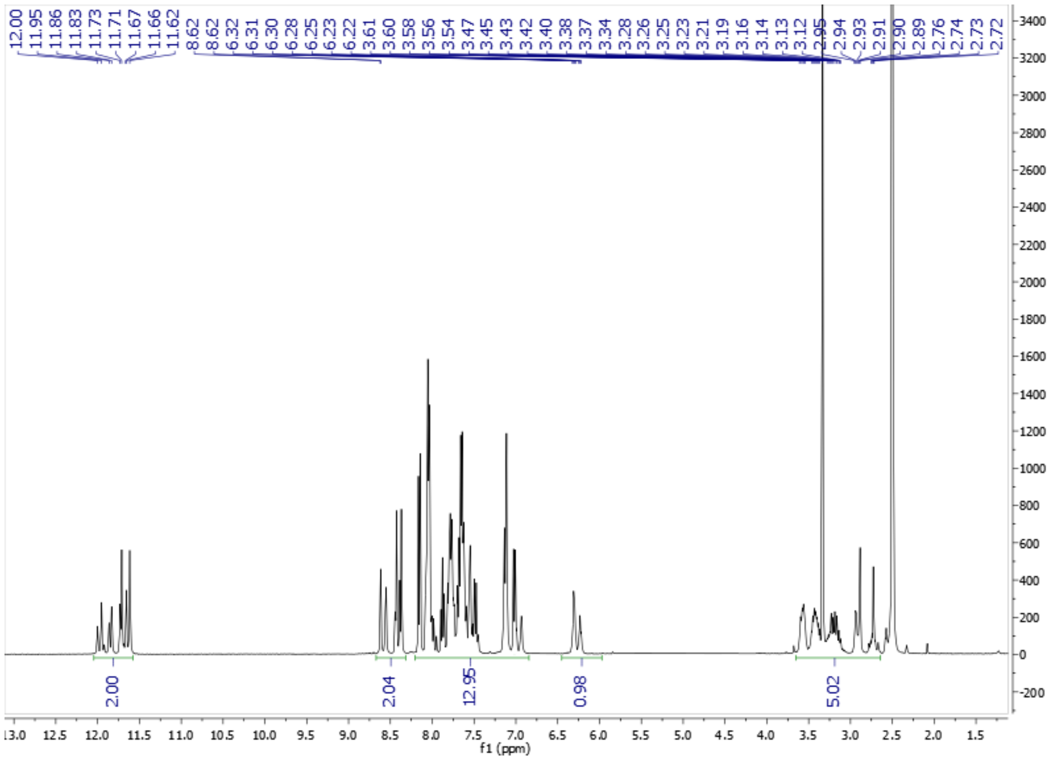

Figure S54. ^{1}H NMR of compound **15b** at 400 MHz (DMSO-d_6)

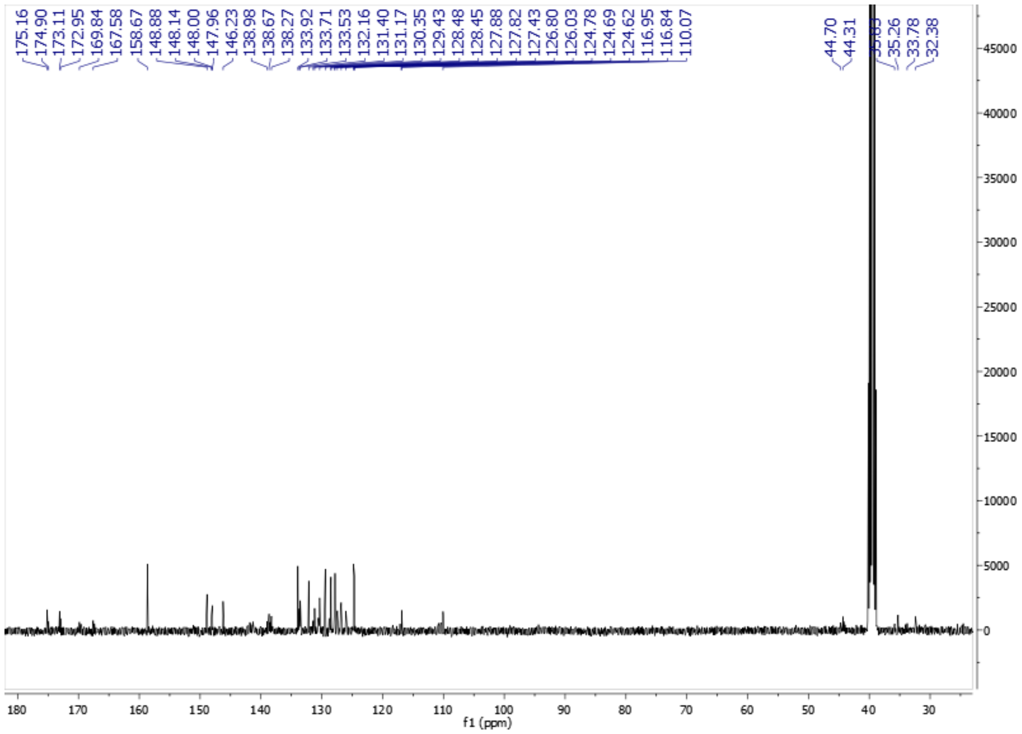

Figure S55. ^{13}C NMR spectrum of compound **15b** at 101 MHz (DMSO-d_6)

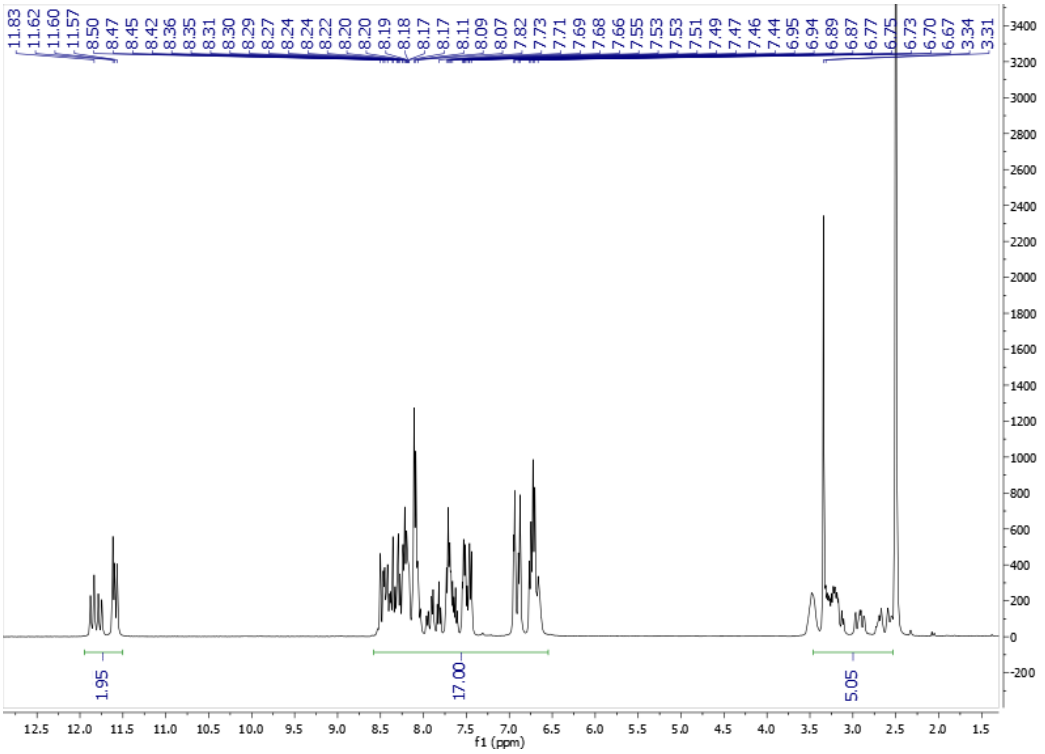

Figure S56. ^{1}H NMR of compound **16a** at 400 MHz (DMSO-d_6)

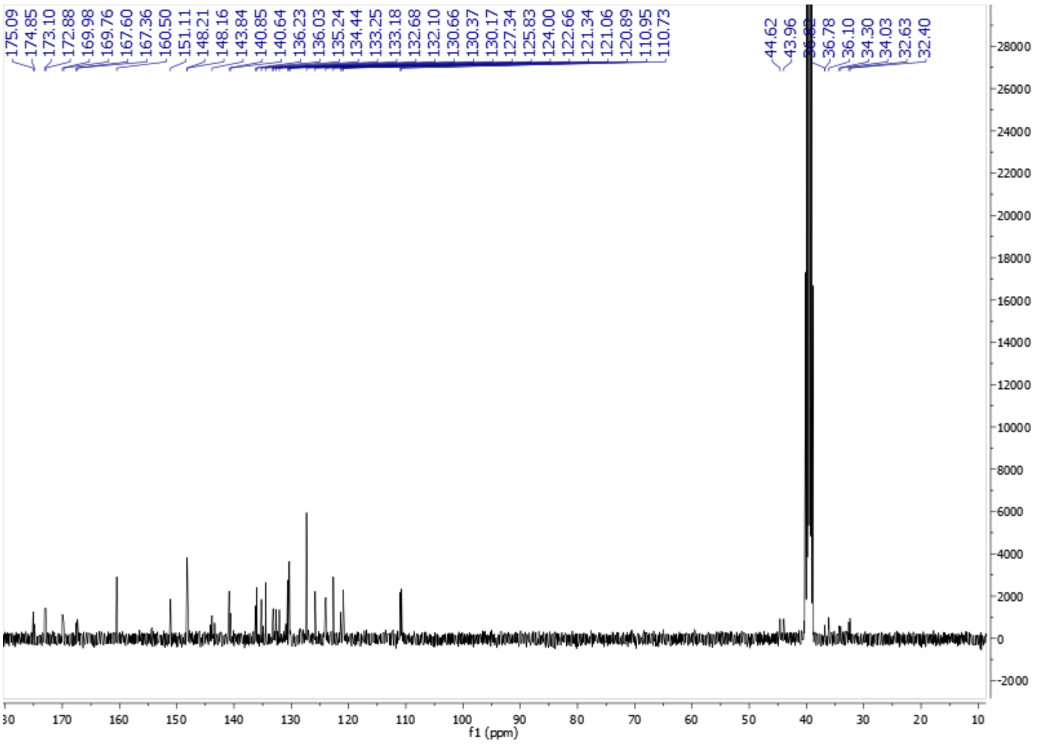

Figure S57. ^{13}C NMR spectrum of compound **16a** at 101 MHz (DMSO-d_6)

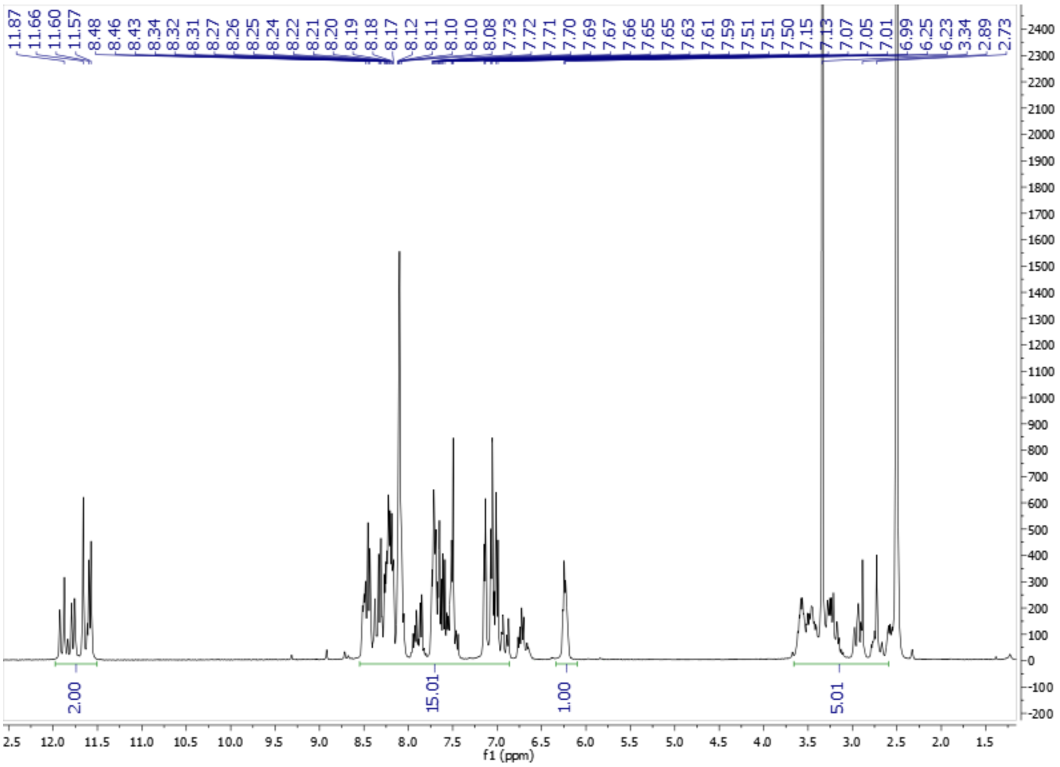

Figure S58. ^{1}H NMR of compound **16b** at 400 MHz (DMSO-*d*6)

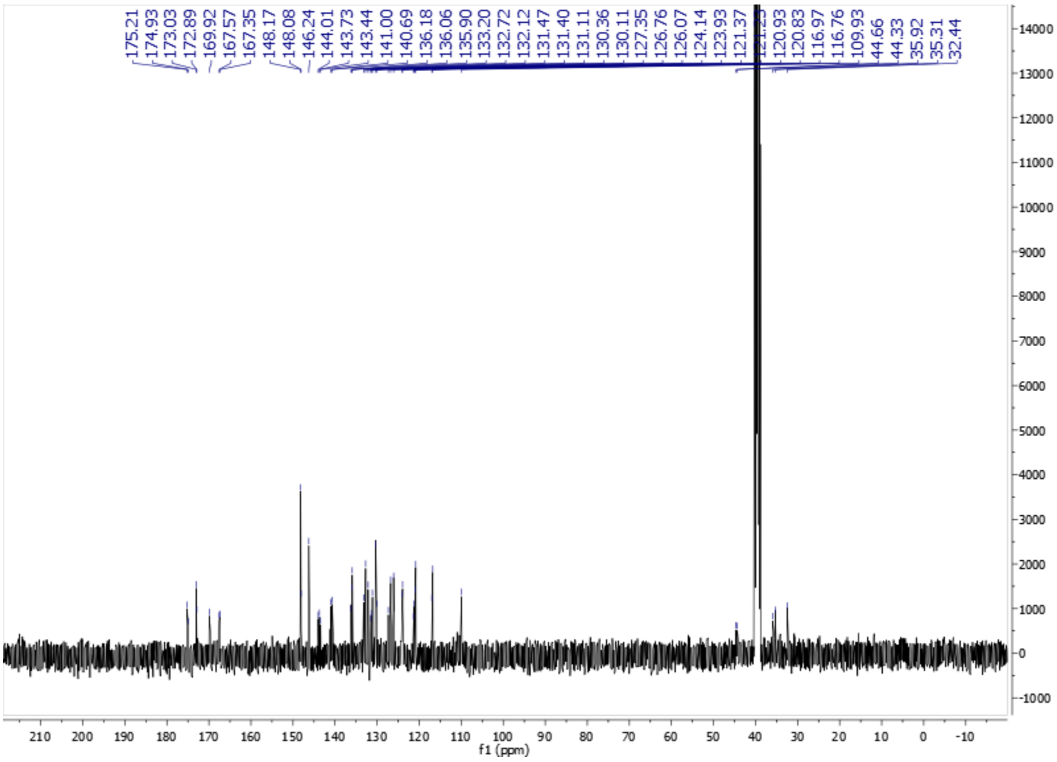

Figure S59. ^{13}C NMR spectrum of compound **16b** at 101 MHz (DMSO-*d*6)

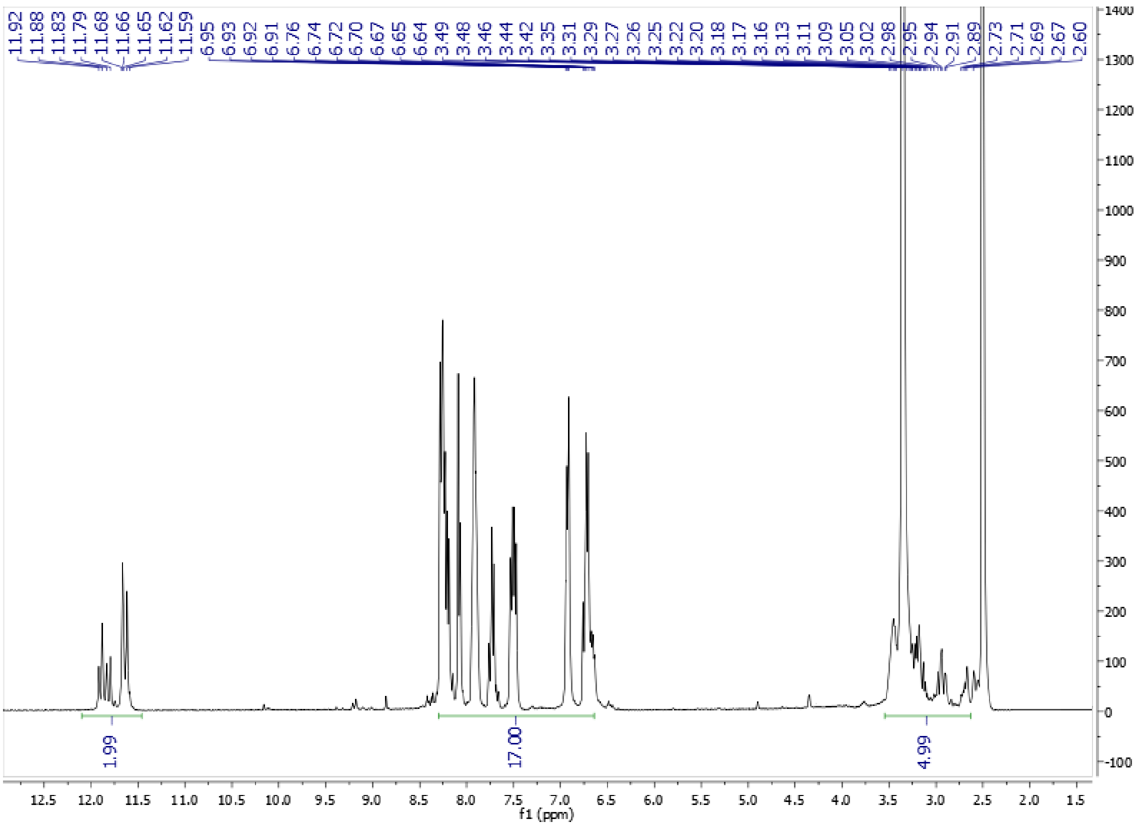

Figure S60. ^{1}H NMR of compound **17a** at 400 MHz (DMSO-d_6)

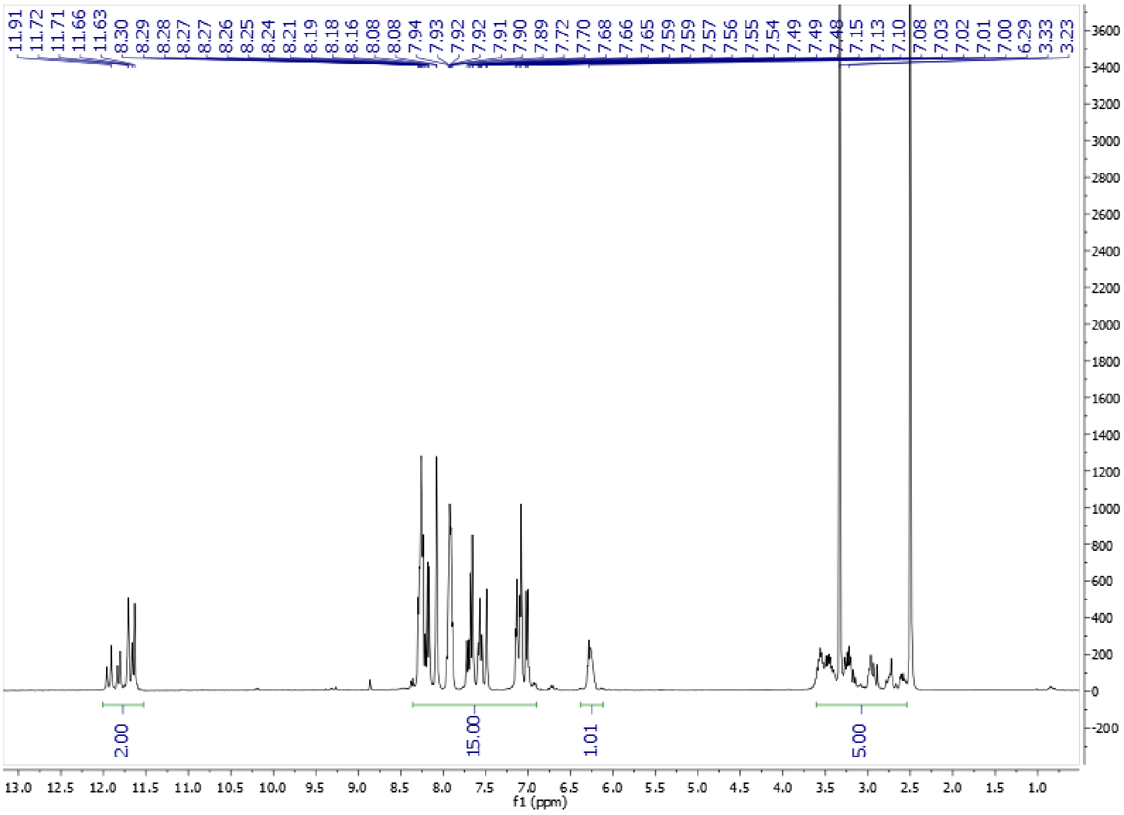

Figure S61. ^{1}H NMR of compound **17b** at 400 MHz (DMSO-d_6)

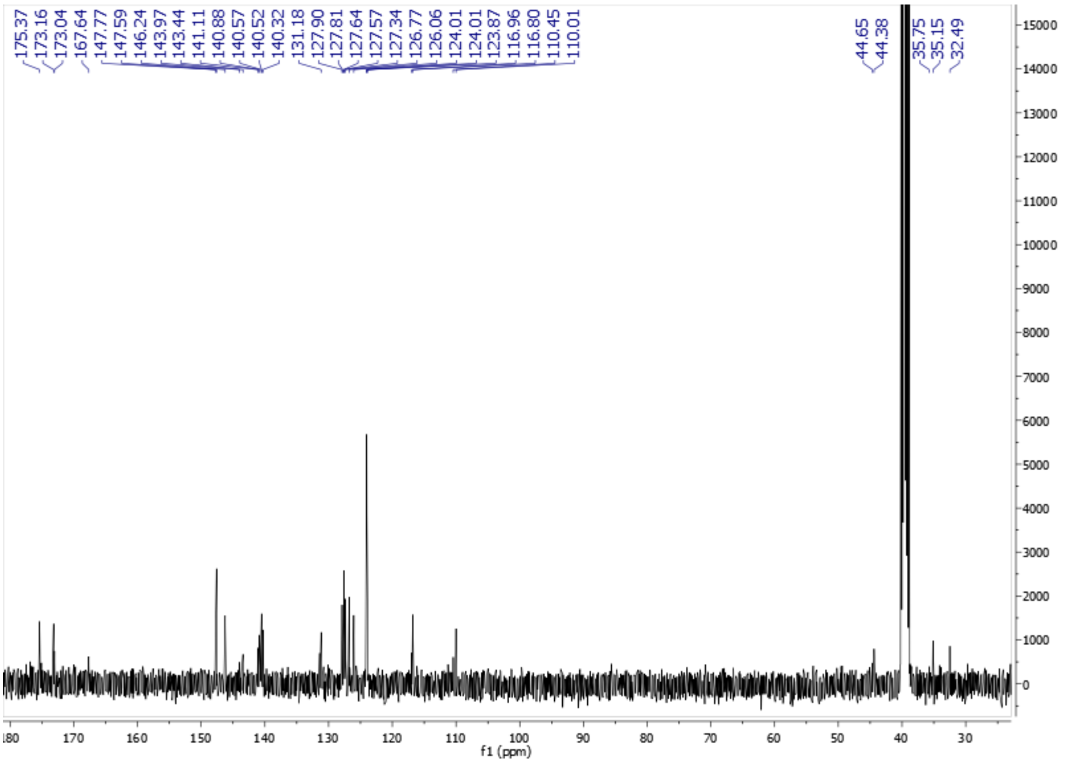

Figure S62. ^{13}C NMR spectrum of compound **17b** at 101 MHz (DMSO-d_6)

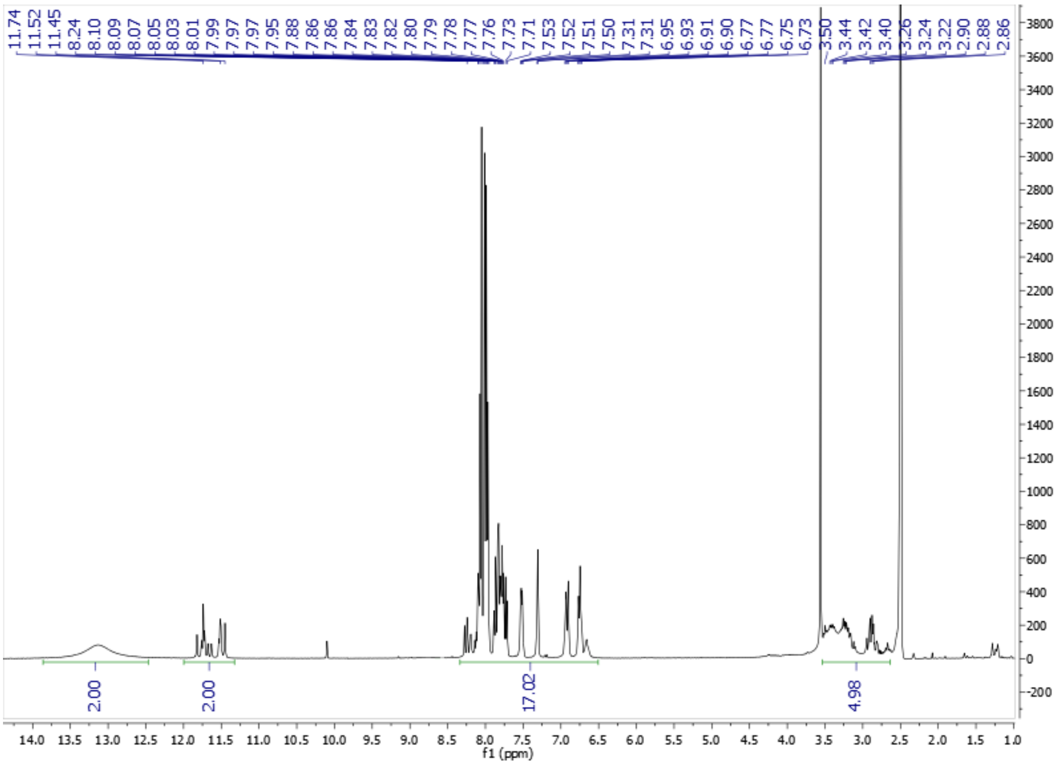

Figure S63. ^{1}H NMR of compound **18a** at 400 MHz (DMSO-d_6)

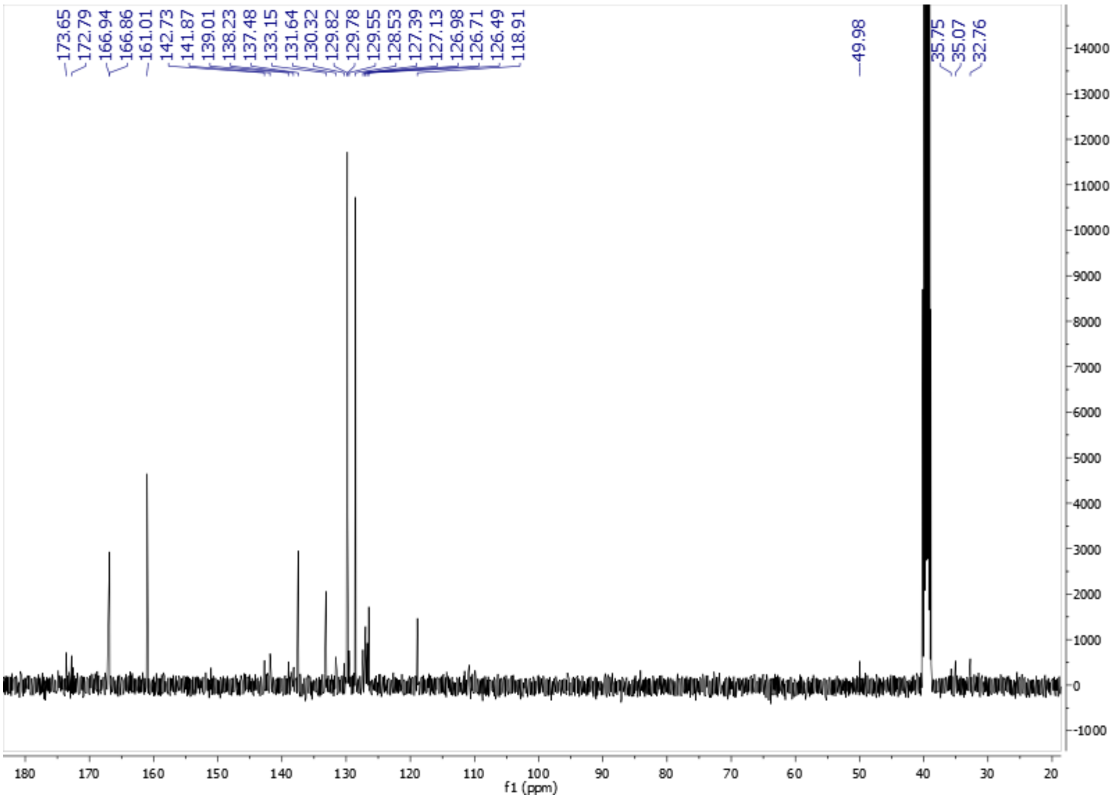

Figure S64. ^{13}C NMR spectrum of compound **18a** at 101 MHz (DMSO-d_6)

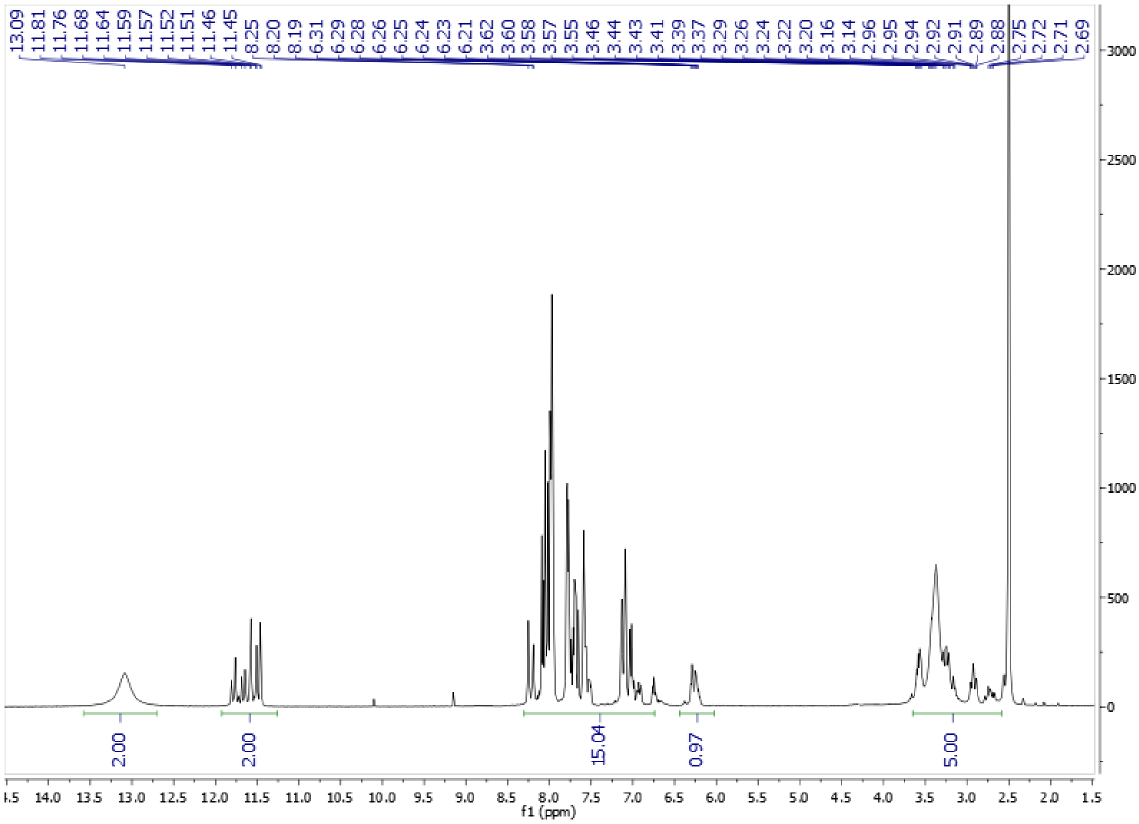

Figure S65. ^{1}H NMR of compound **18b** at 400 MHz (DMSO-d_6)

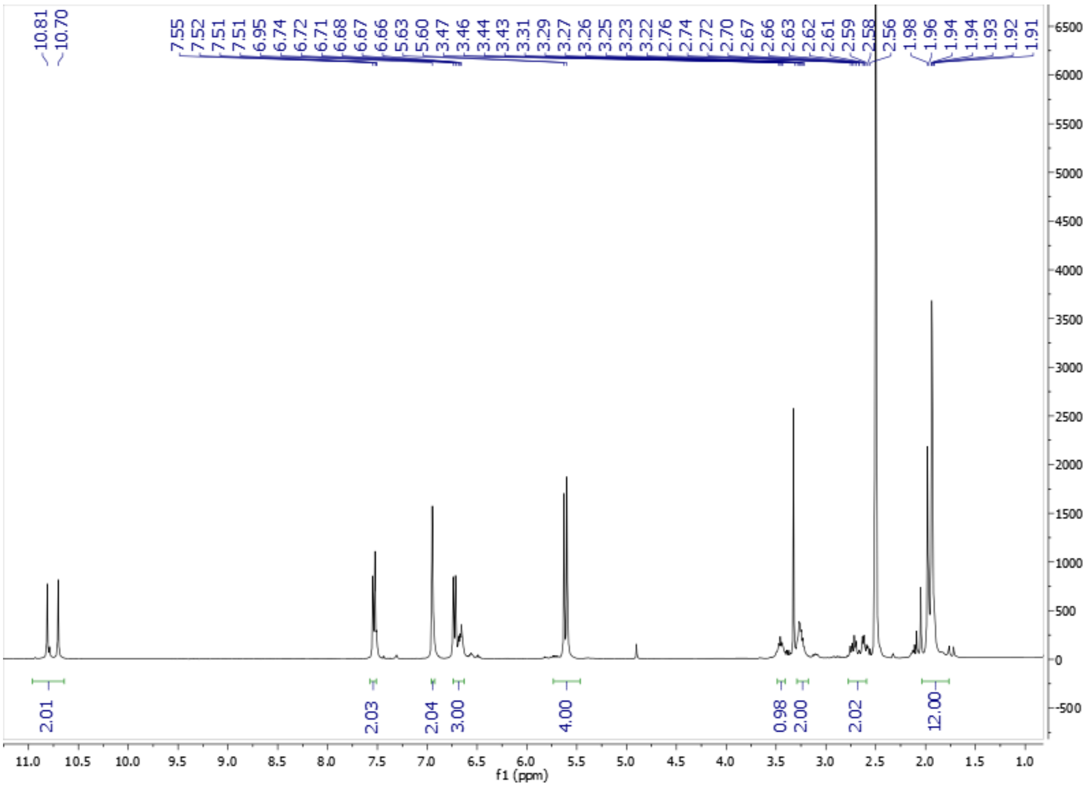

Figure S66. ^{1}H NMR of compound **19a** at 400 MHz (DMSO-d_6)

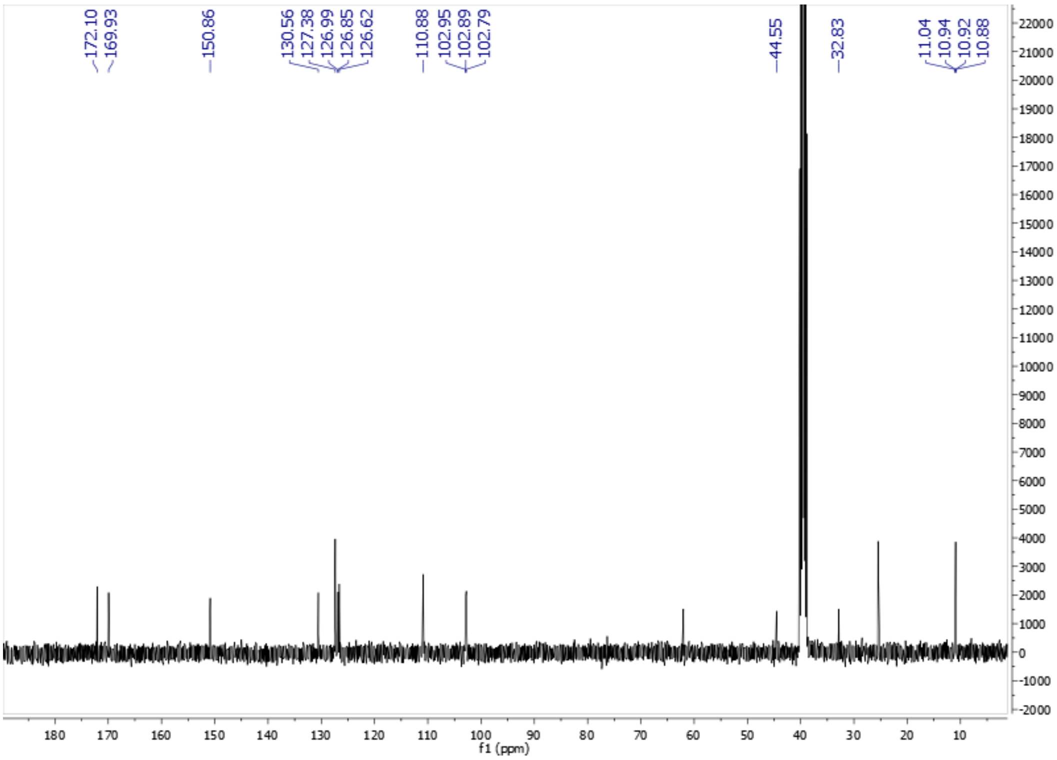

Figure S67. ^{13}C NMR spectrum of compound **19a** at 101 MHz (DMSO-d_6)

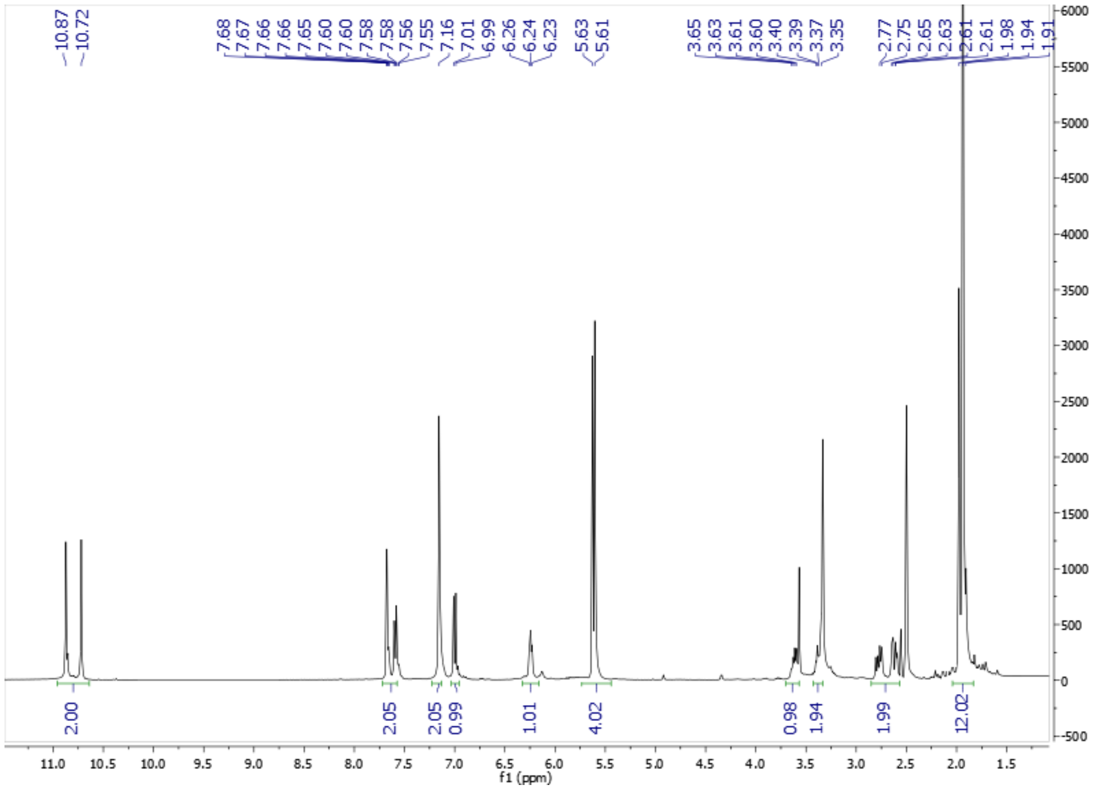

Figure S68. ^{1}H NMR of compound **19b** at 400 MHz (DMSO-d_6)

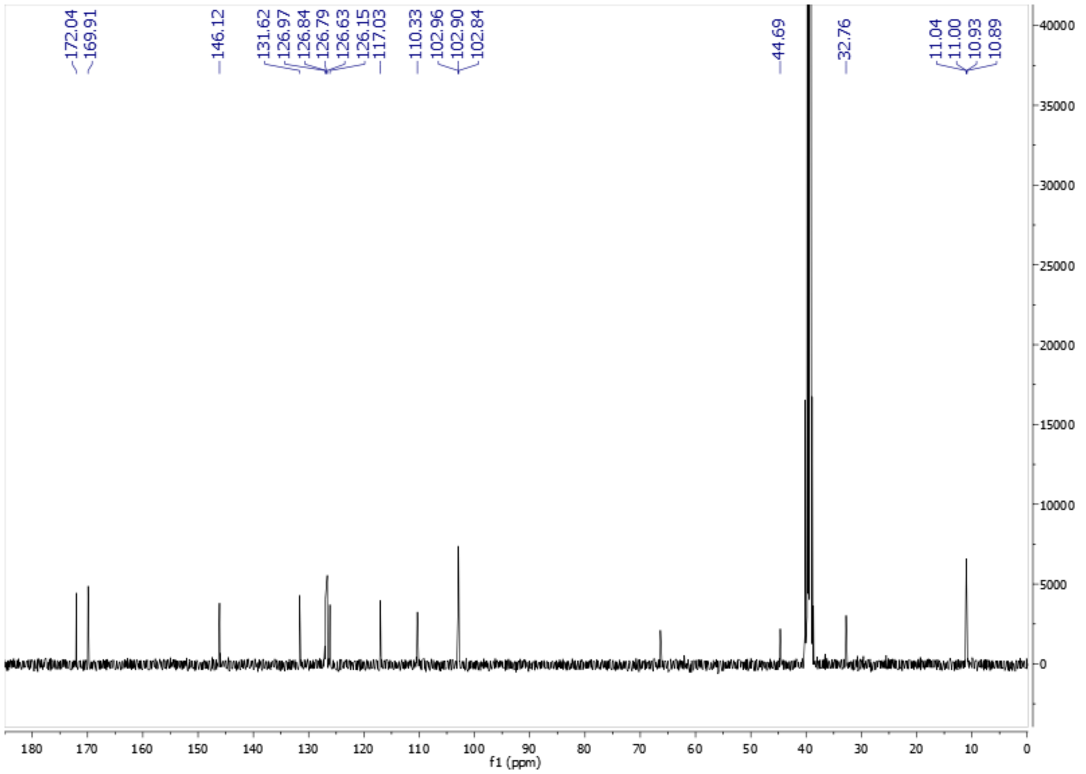

Figure S69. ^{13}C NMR spectrum of compound **19b** at 101 MHz (DMSO-d_6)

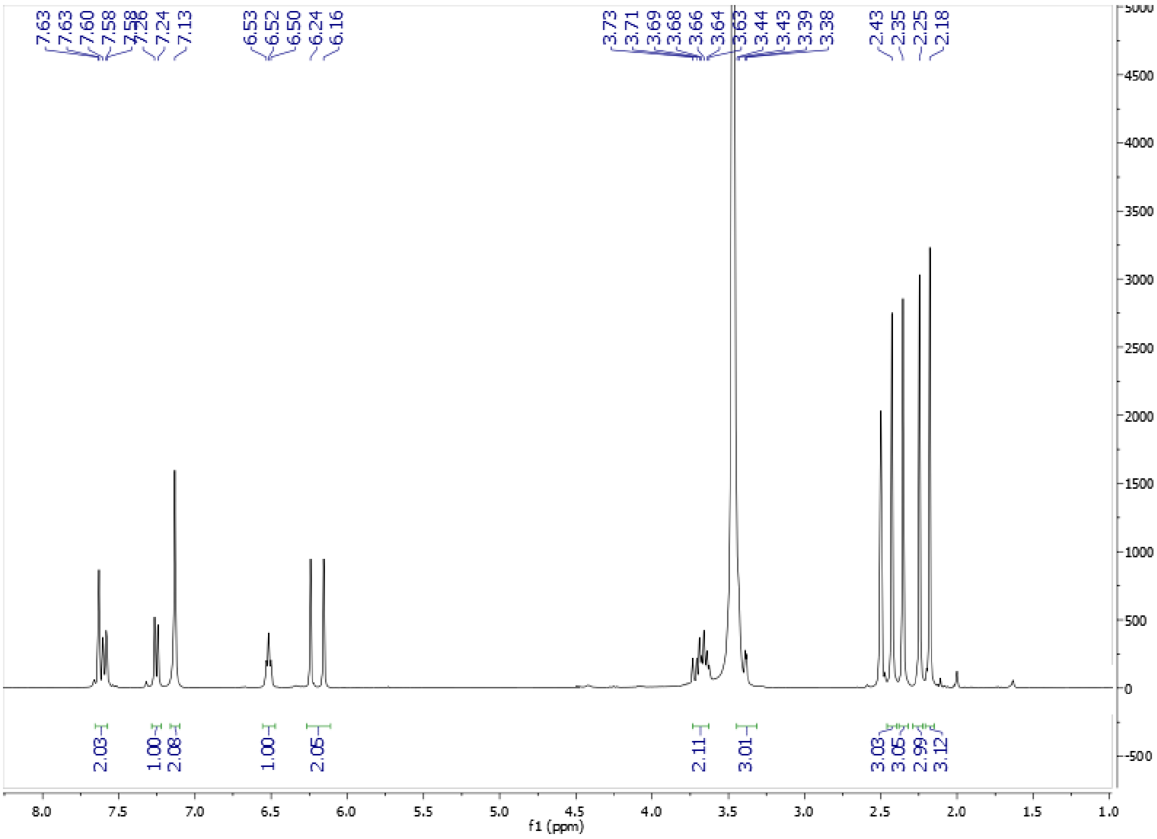

Figure S70. ^{1}H NMR of compound **20** at 400 MHz (DMSO-*d*$_6$)

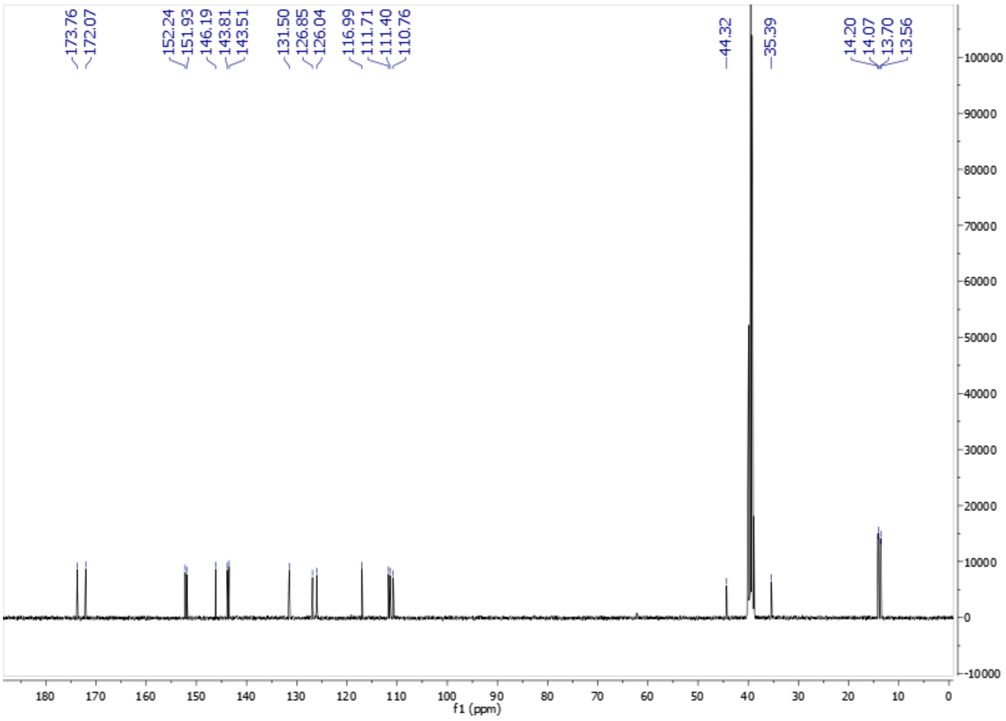

Figure S71. ^{13}C NMR spectrum of compound **20** at 101 MHz (DMSO-*d*$_6$)

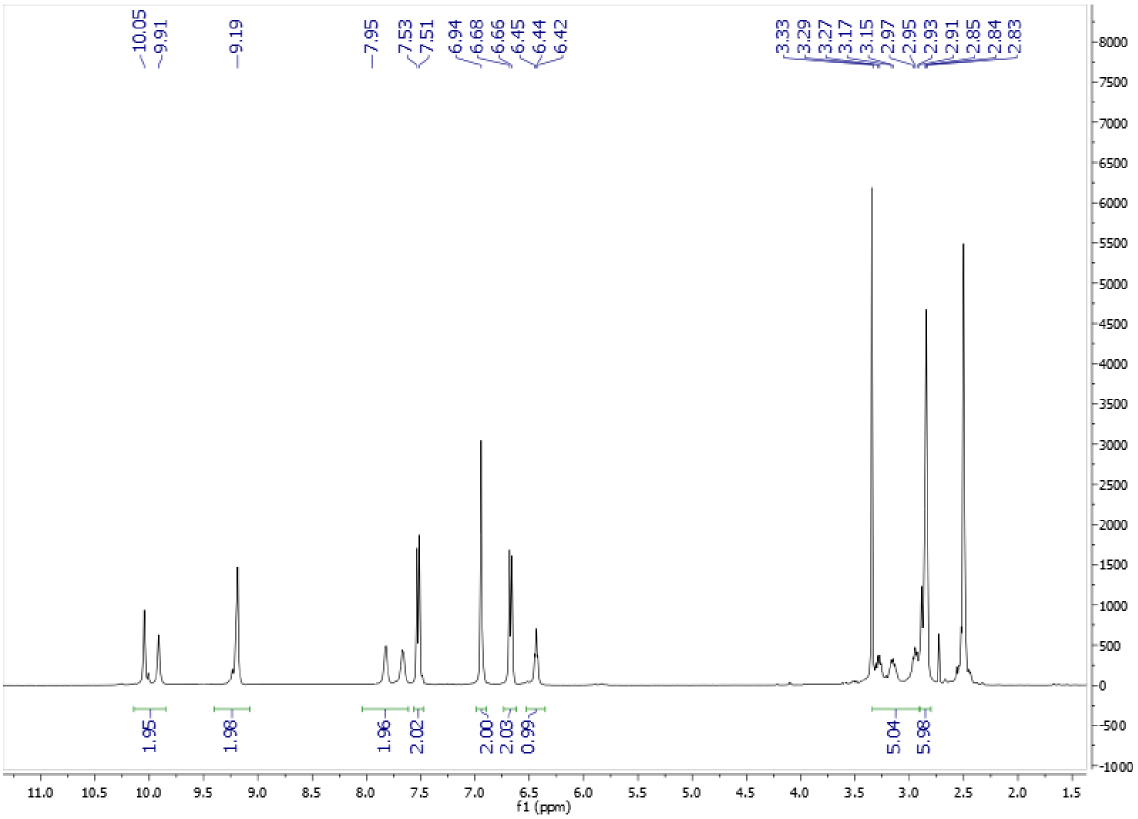

Figure S72. ^{1}H NMR of compound **22a** at 400 MHz (DMSO-d_6)

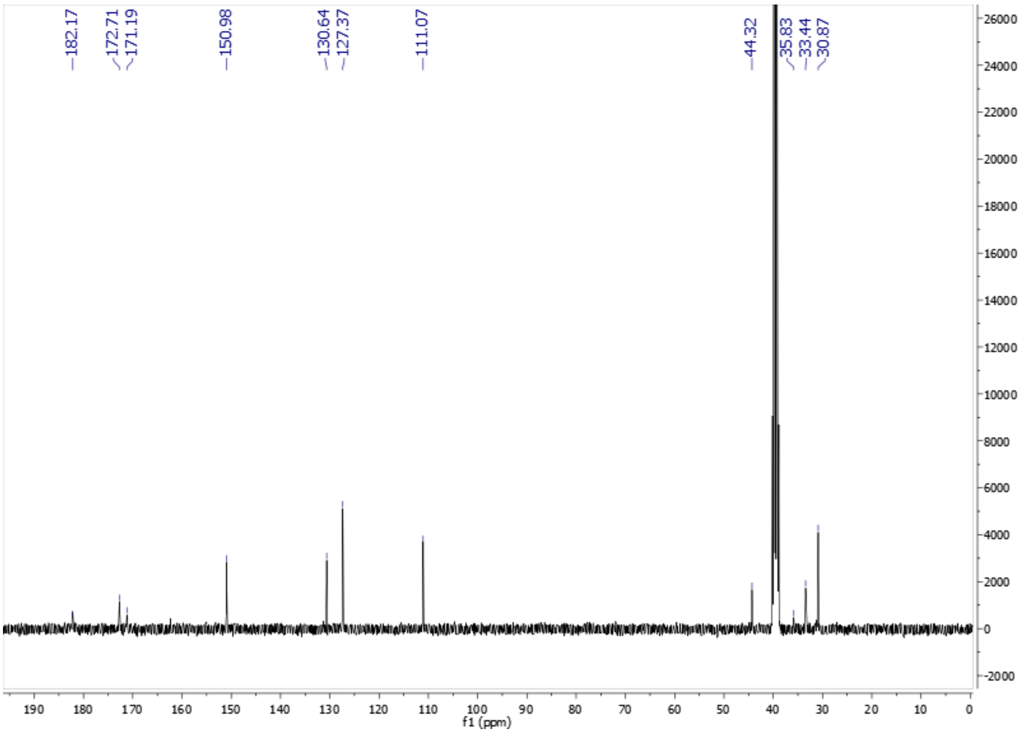

Figure S73. ^{13}C NMR spectrum of compound **22a** at 101 MHz (DMSO-d_6)

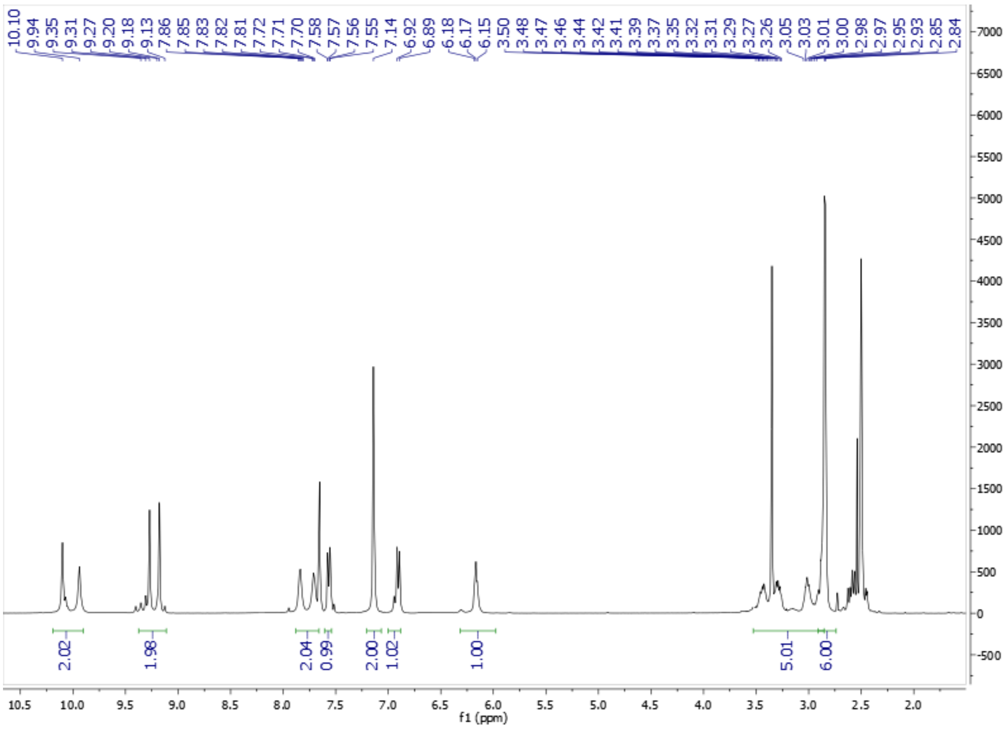

Figure S74. ^{1}H NMR of compound **22b** at 400 MHz (DMSO-d_6)

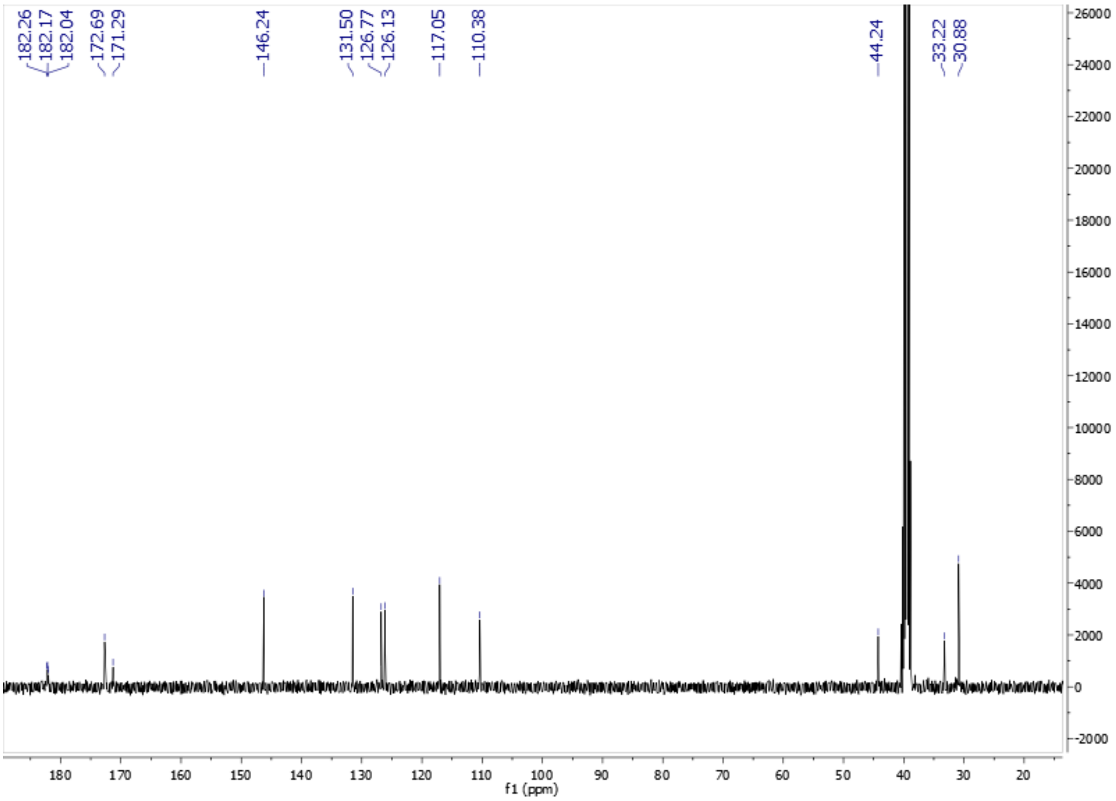

Figure S75. ^{13}C NMR spectrum of compound **22b** at 101 MHz (DMSO-d_6)

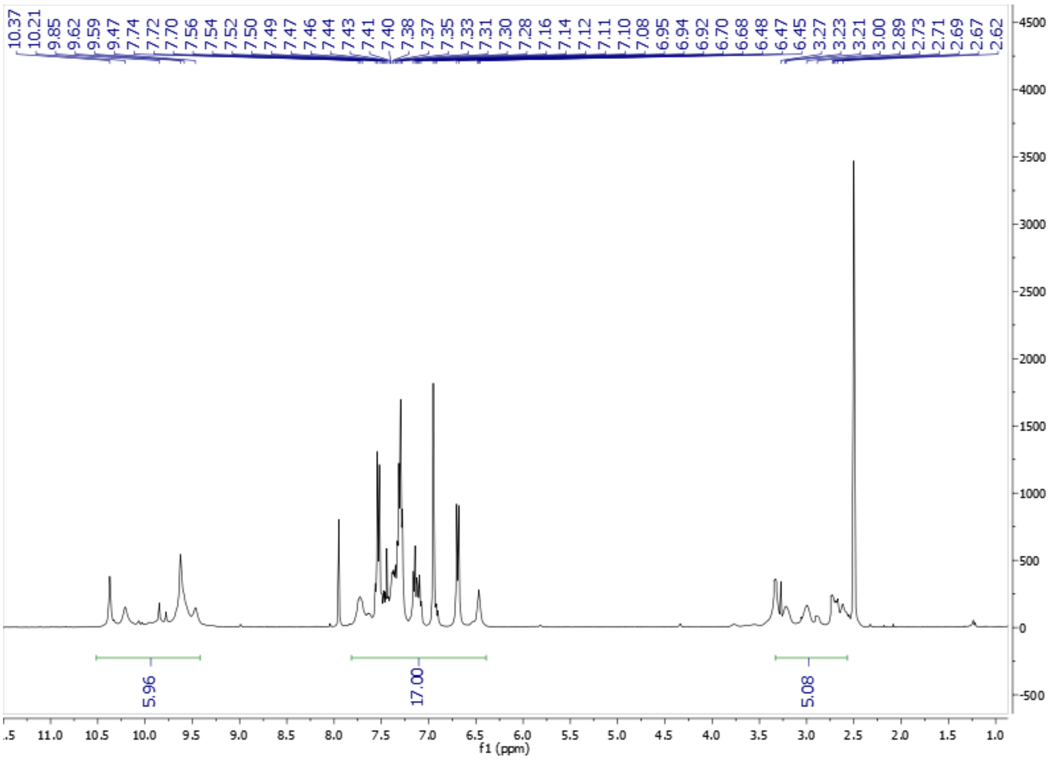

Figure S76. ^{1}H NMR of compound **23a** at 400 MHz (DMSO-d_6)

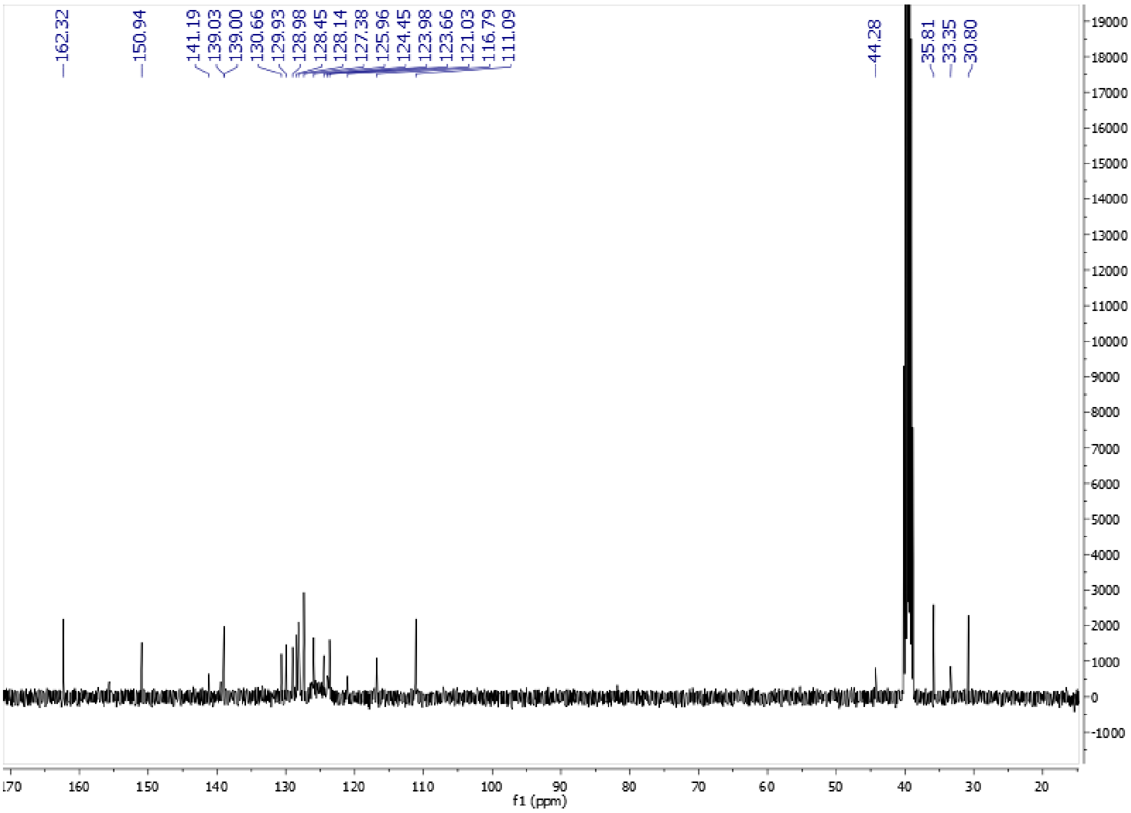

Figure S77. ^{13}C NMR spectrum of compound **23a** at 101 MHz (DMSO-d_6)

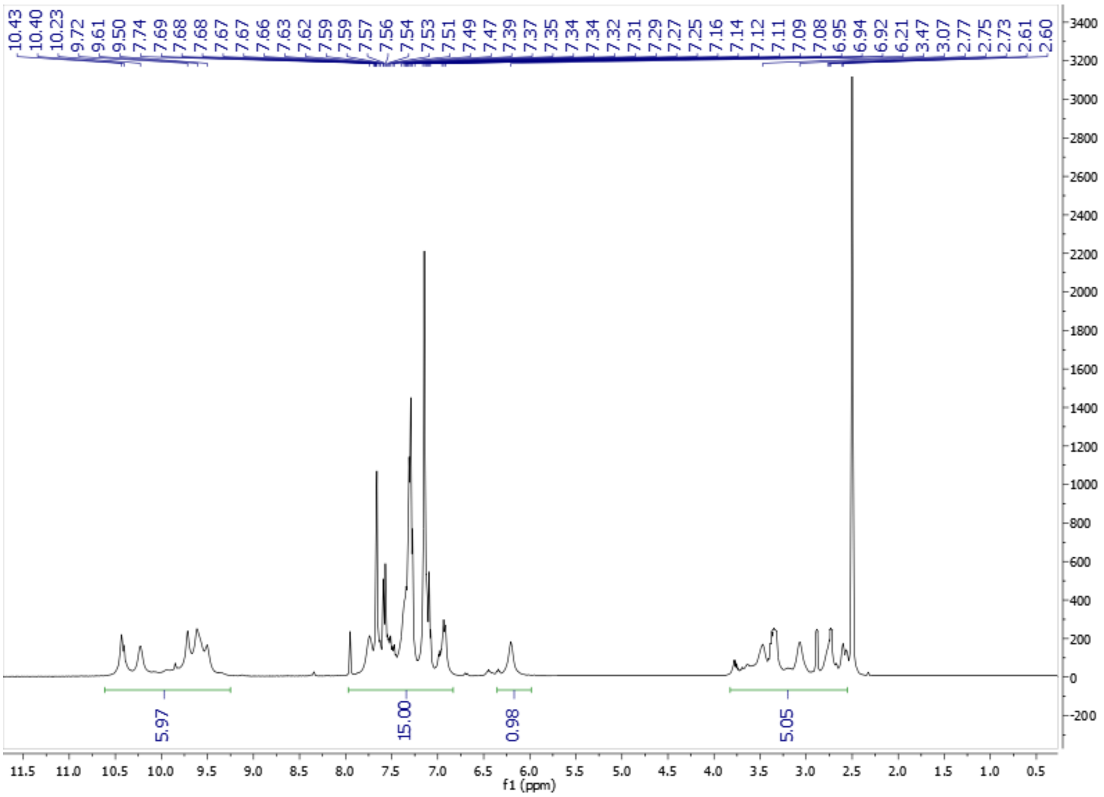

Figure S78. ^{1}H NMR of compound **23b** at 400 MHz (DMSO-d_6)

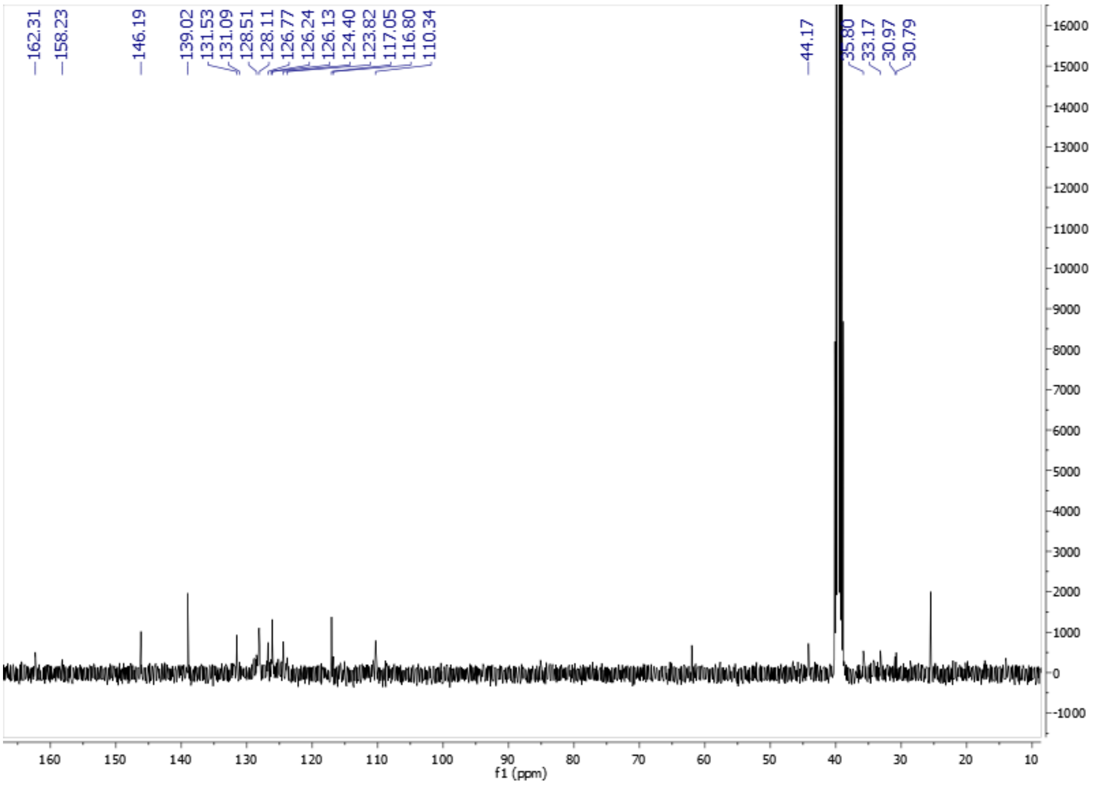

Figure S79. ^{13}C NMR spectrum of compound **23b** at 101 MHz (DMSO-d_6)

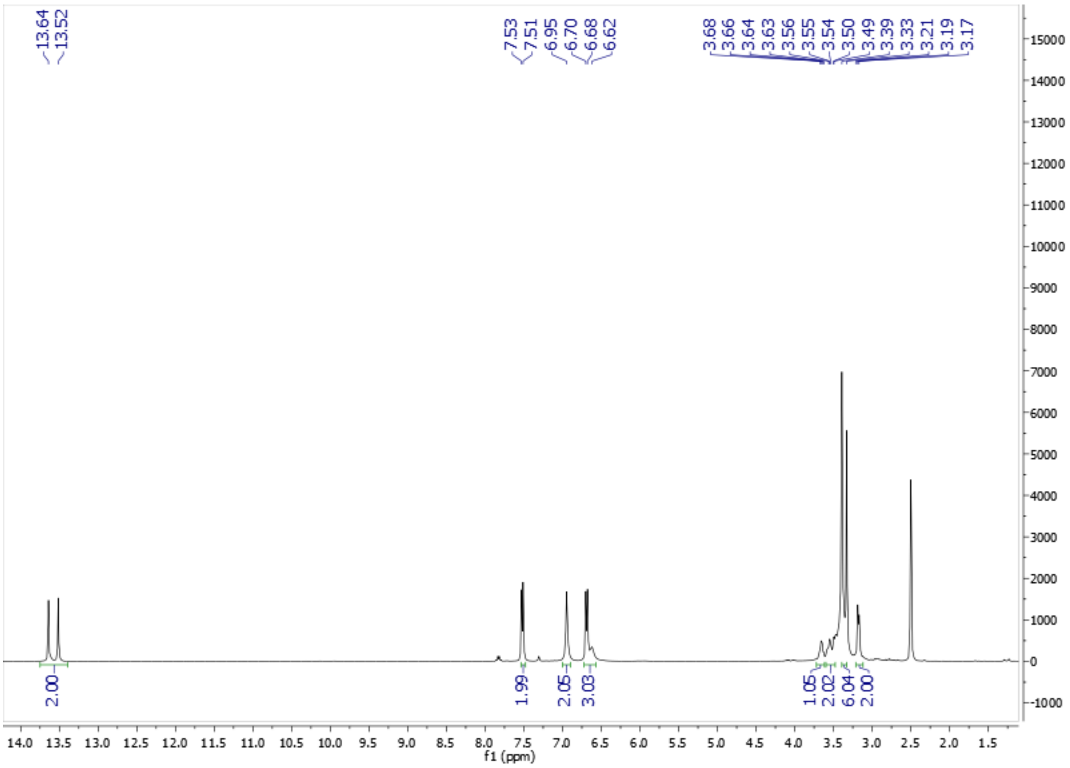

Figure S80. ^{1}H NMR of compound **24a** at 400 MHz (DMSO-d_6)

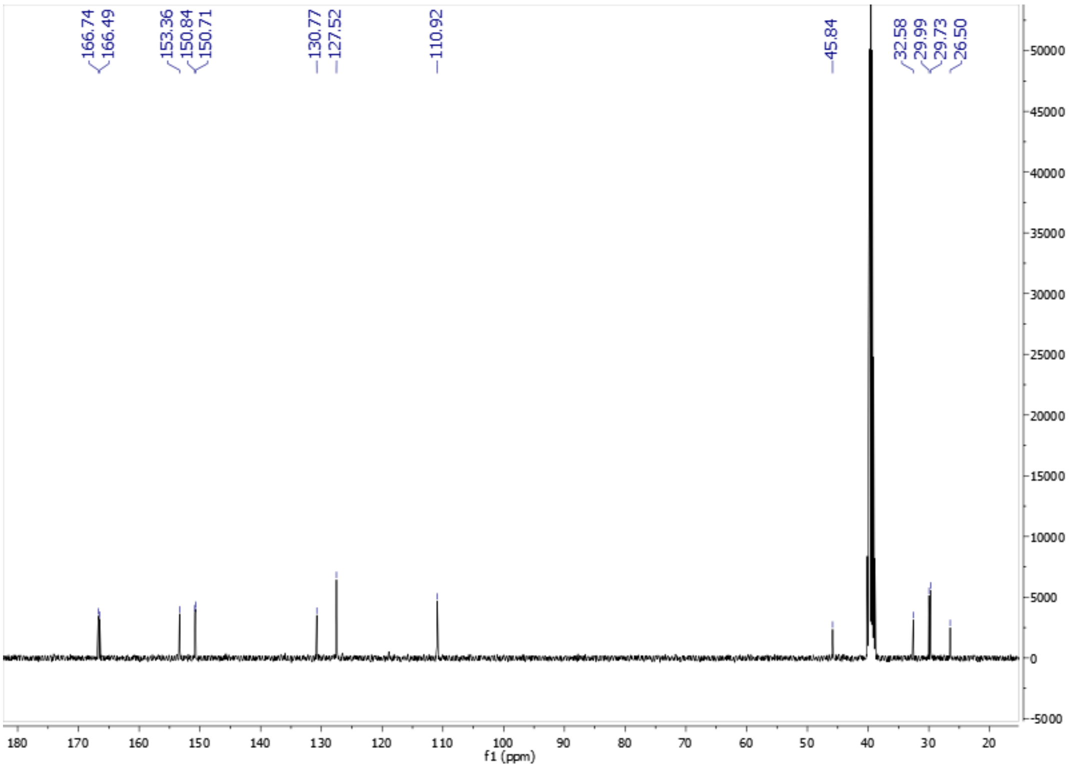

Figure S81. ^{13}C NMR spectrum of compound **24a** at 101 MHz (DMSO-d_6)

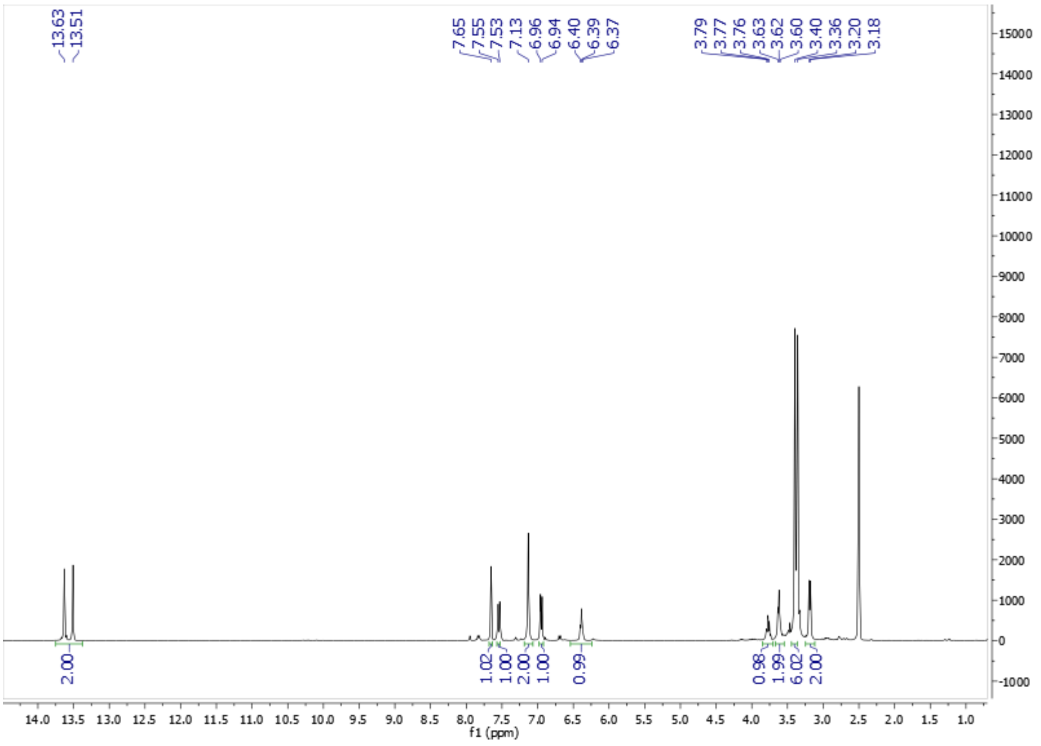

Figure S82. ^{1}H NMR of compound **24b** at 400 MHz (DMSO-d_6)

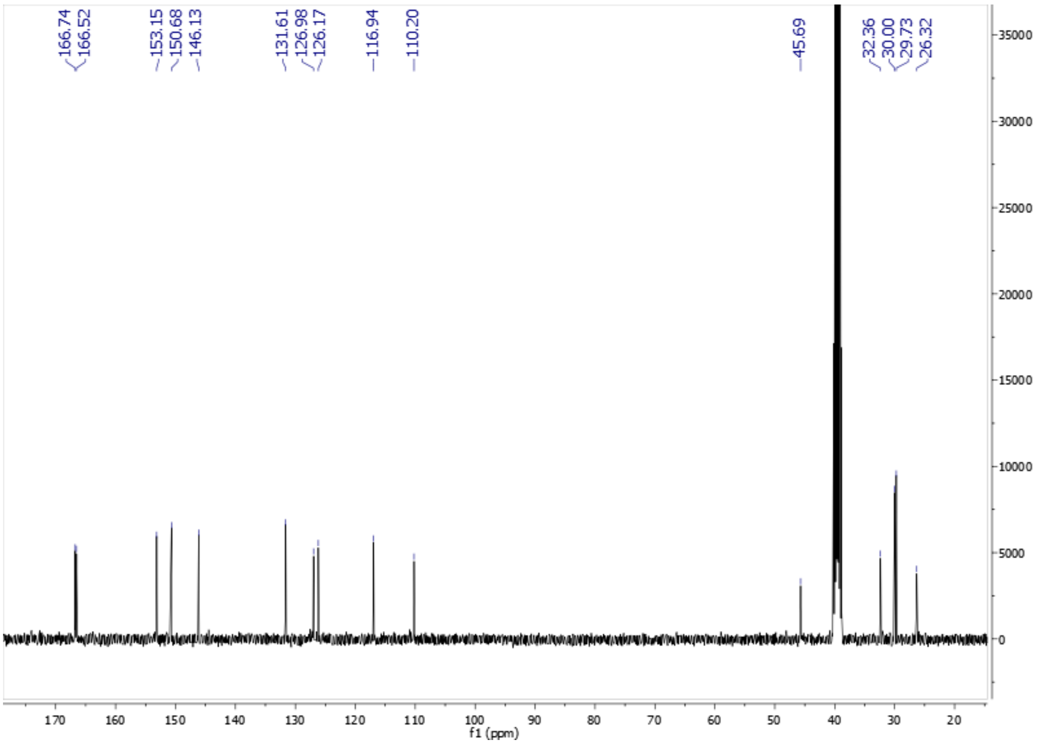

Figure S83. ^{13}C NMR spectrum of compound **24b** at 101 MHz (DMSO-d_6)

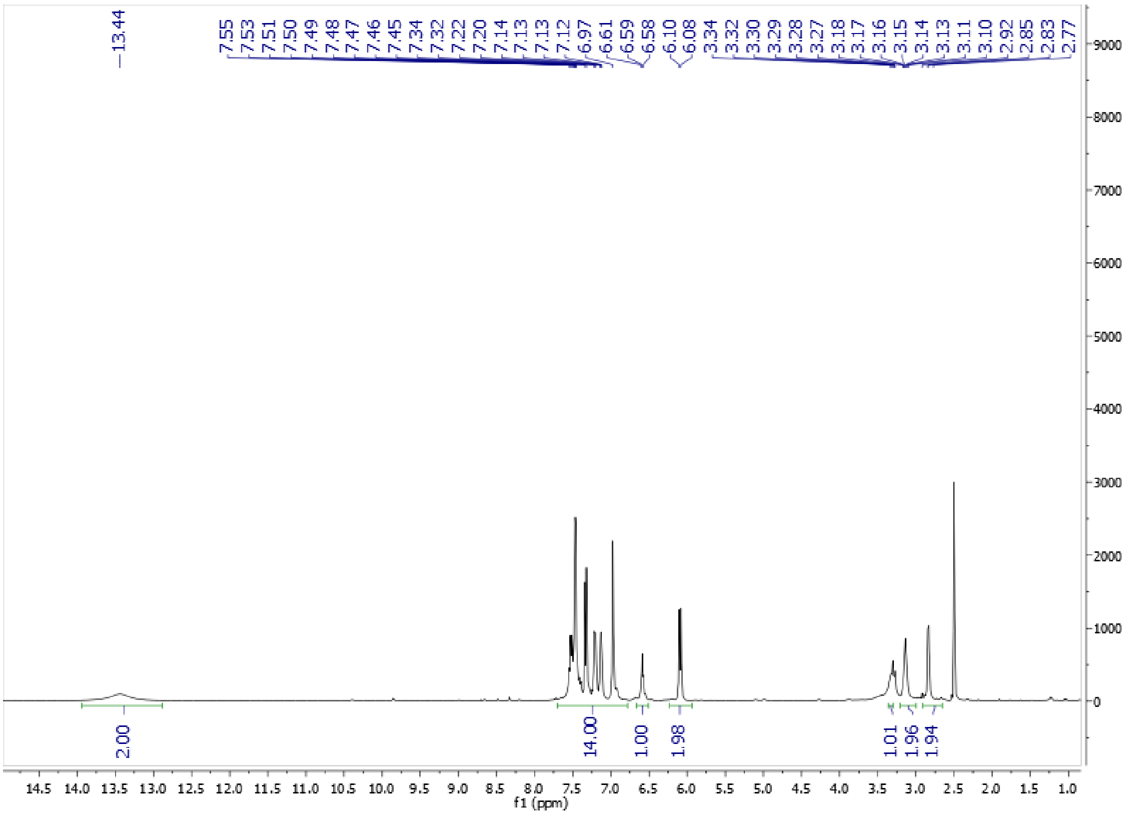

Figure S84. ^{1}H NMR of compound **25a** at 400 MHz (DMSO-d_6)

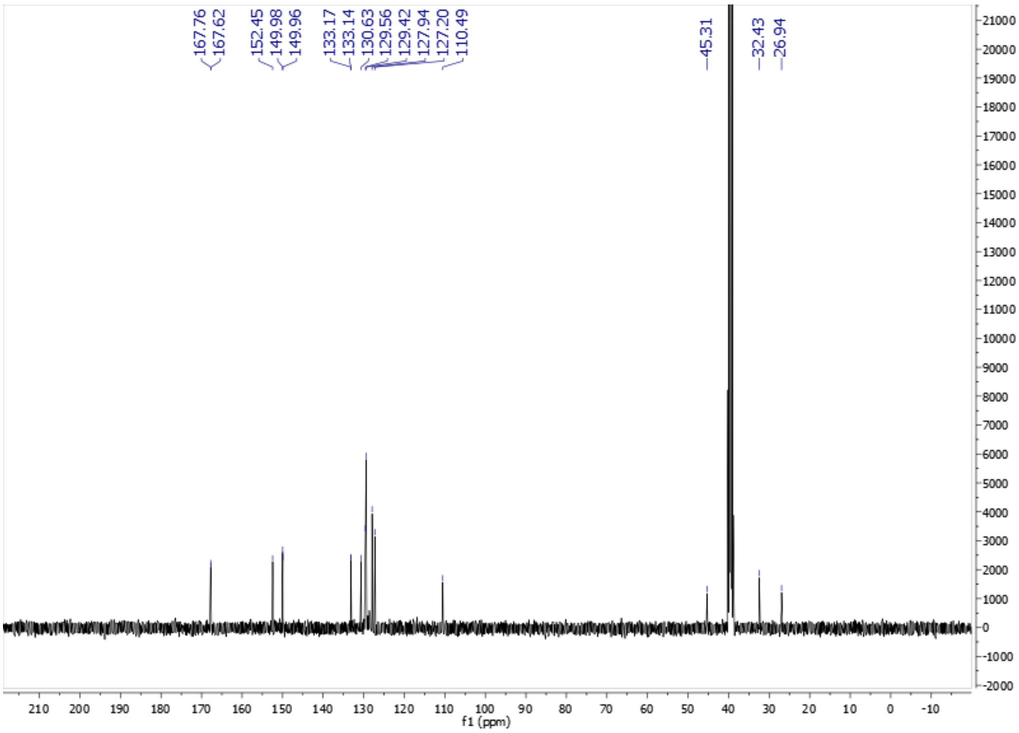

Figure S85. ^{13}C NMR spectrum of compound **25a** at 101 MHz (DMSO-d_6)

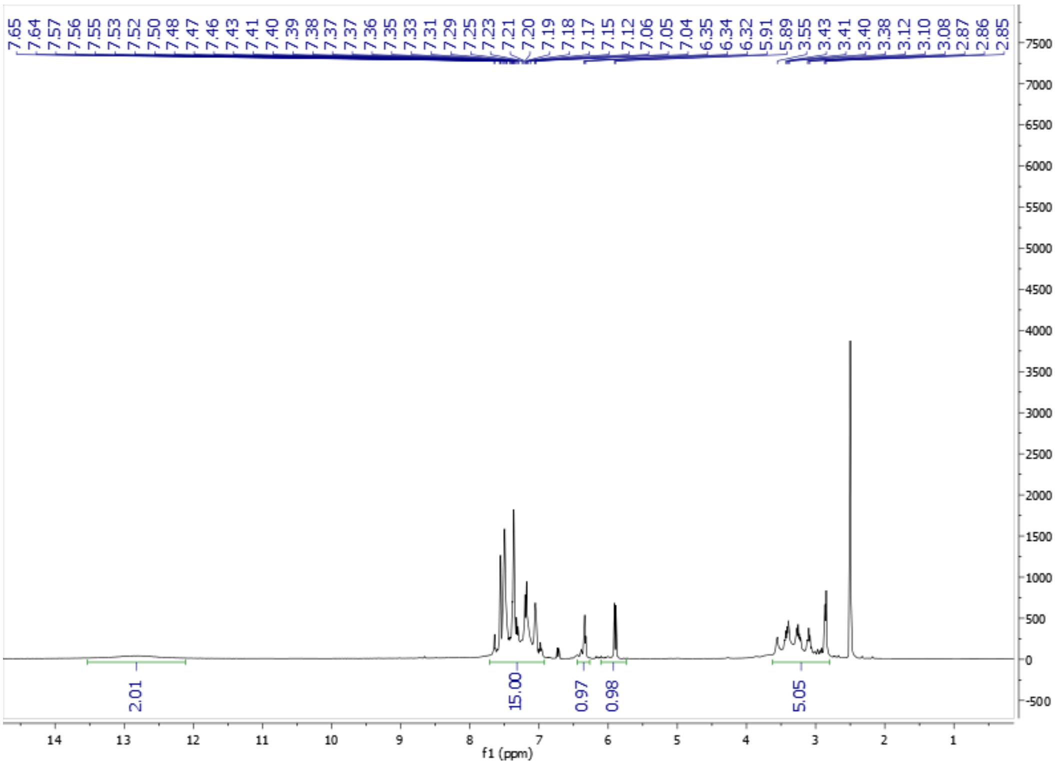

Figure S86. ^{1}H NMR of compound **25b** at 400 MHz (DMSO-*d*₆)

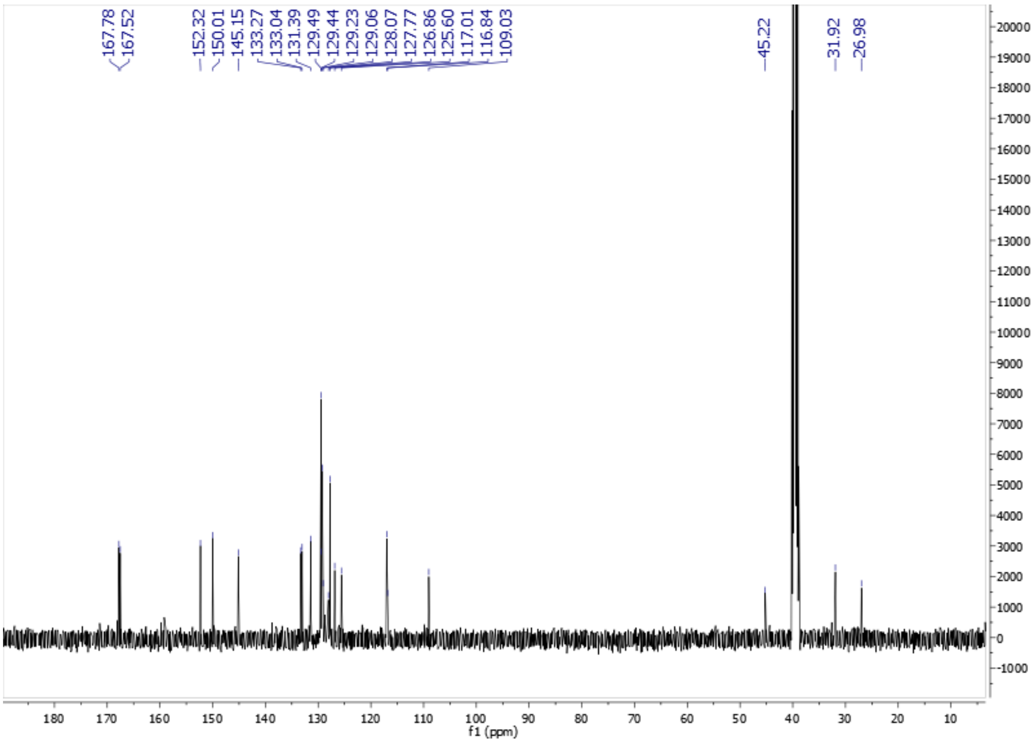

Figure S87. ^{13}C NMR spectrum of compound **25b** at 101 MHz (DMSO-*d*₆)

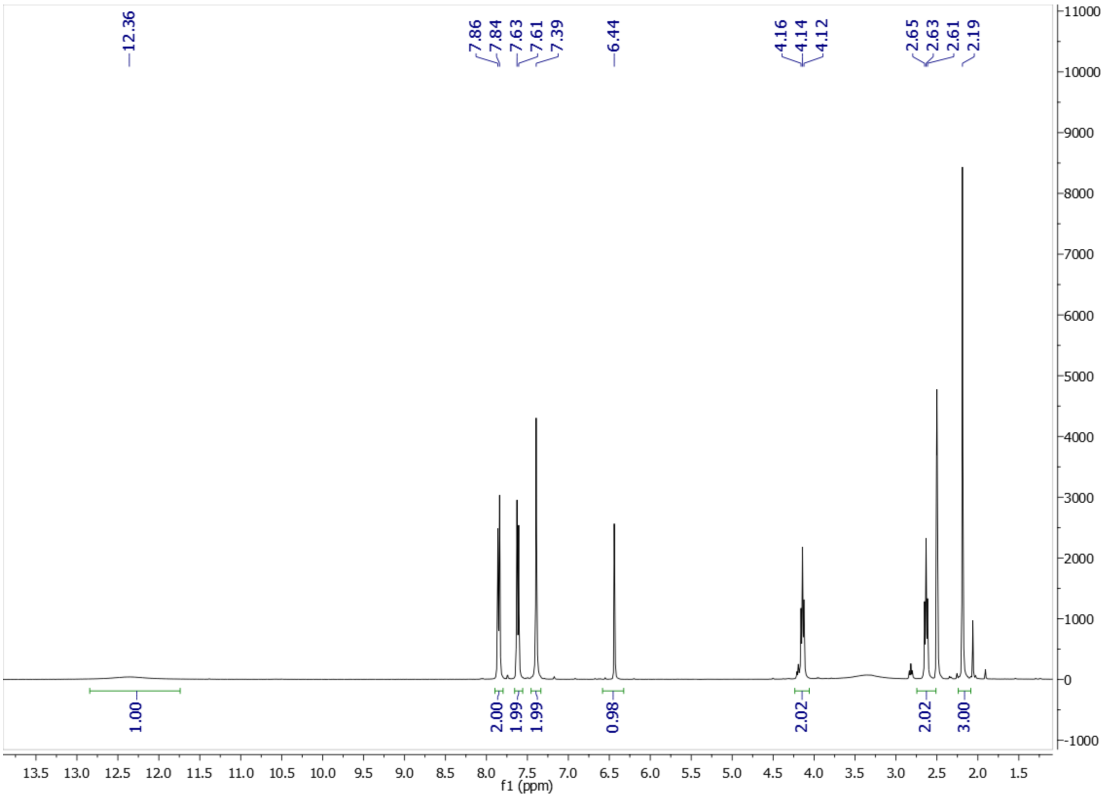

Figure S88. ^{1}H NMR of compound **27** at 400 MHz (DMSO-d_6)

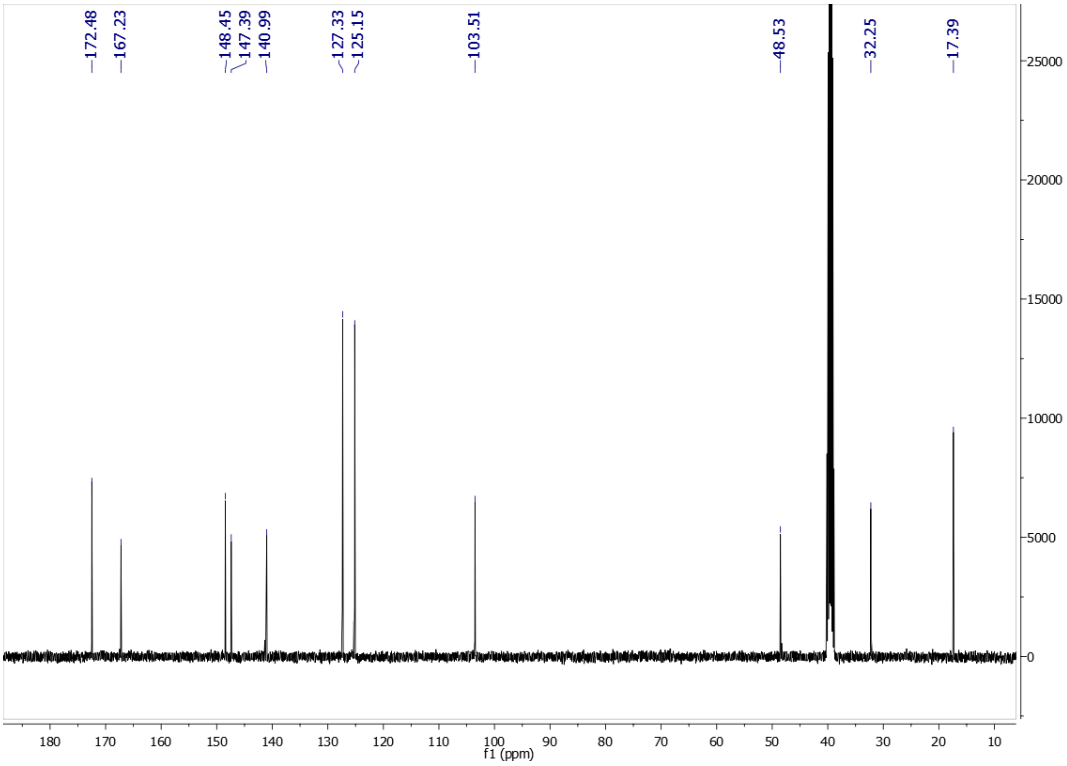

Figure S89. ^{13}C NMR spectrum of compound **27** at 101 MHz (DMSO-d_6)

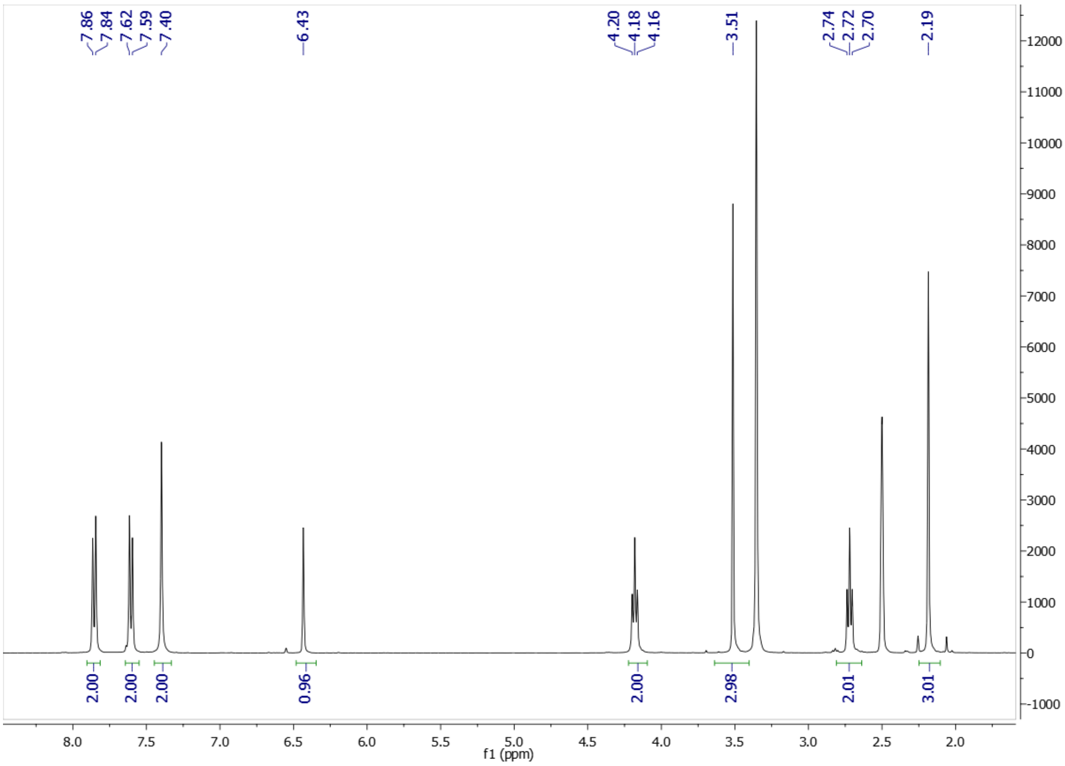

Figure S90. ^{1}H NMR of compound **28** at 400 MHz (DMSO-*d*6)

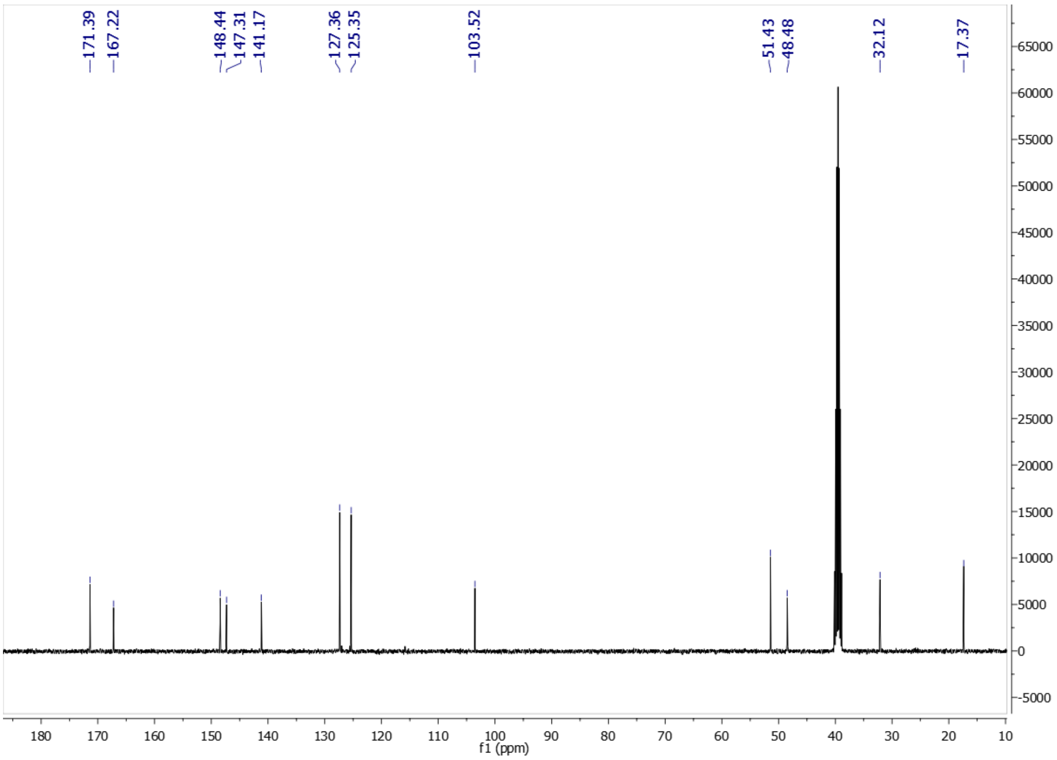

Figure S91. ^{13}C NMR spectrum of compound **28** at 101 MHz (DMSO-*d*6)

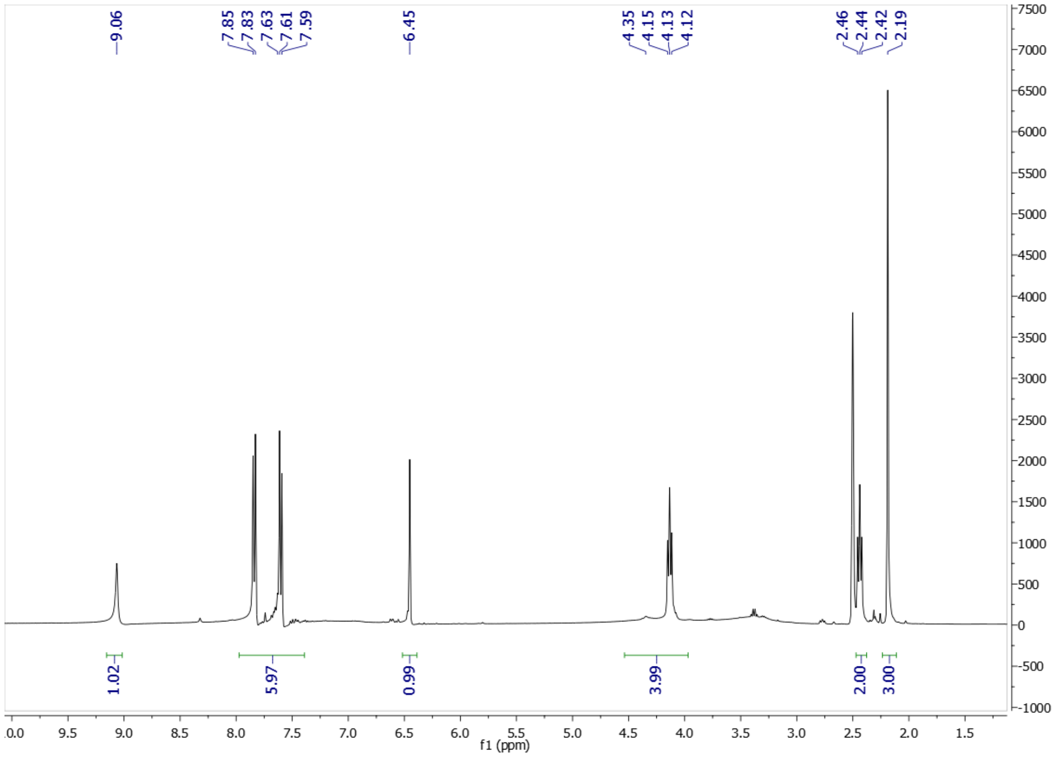

Figure S92. ^{1}H NMR of compound **29** at 400 MHz (DMSO-*d*6)

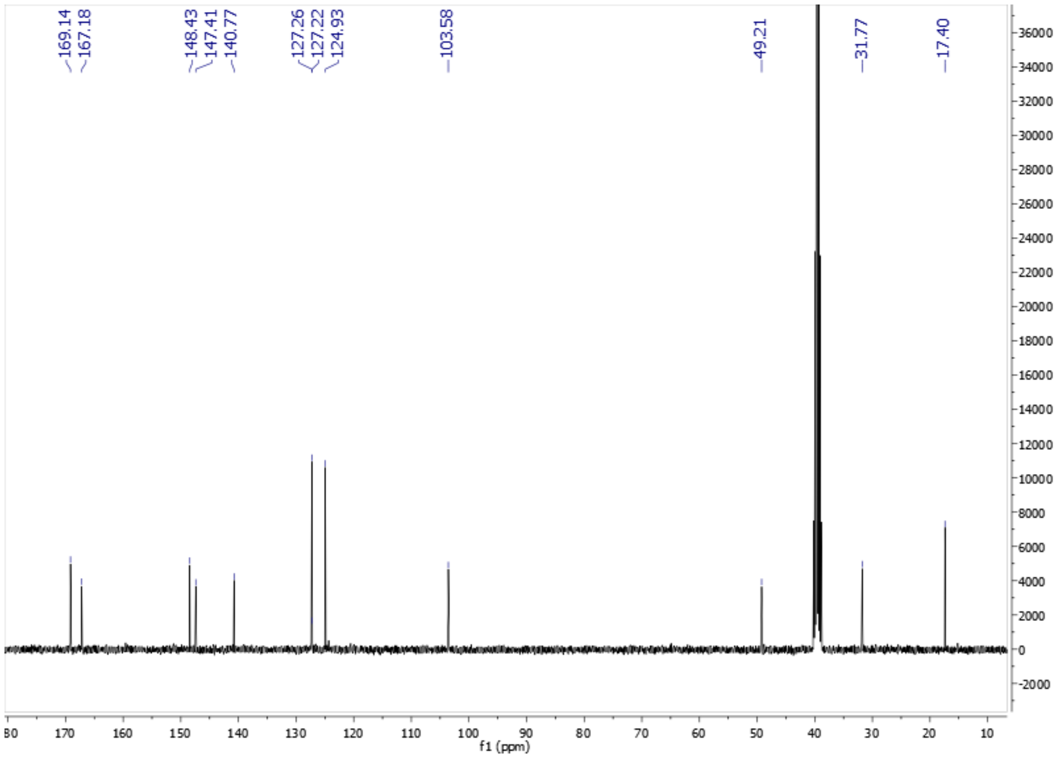

Figure S93. ^{13}C NMR spectrum of compound **29** at 101 MHz (DMSO-*d*6)

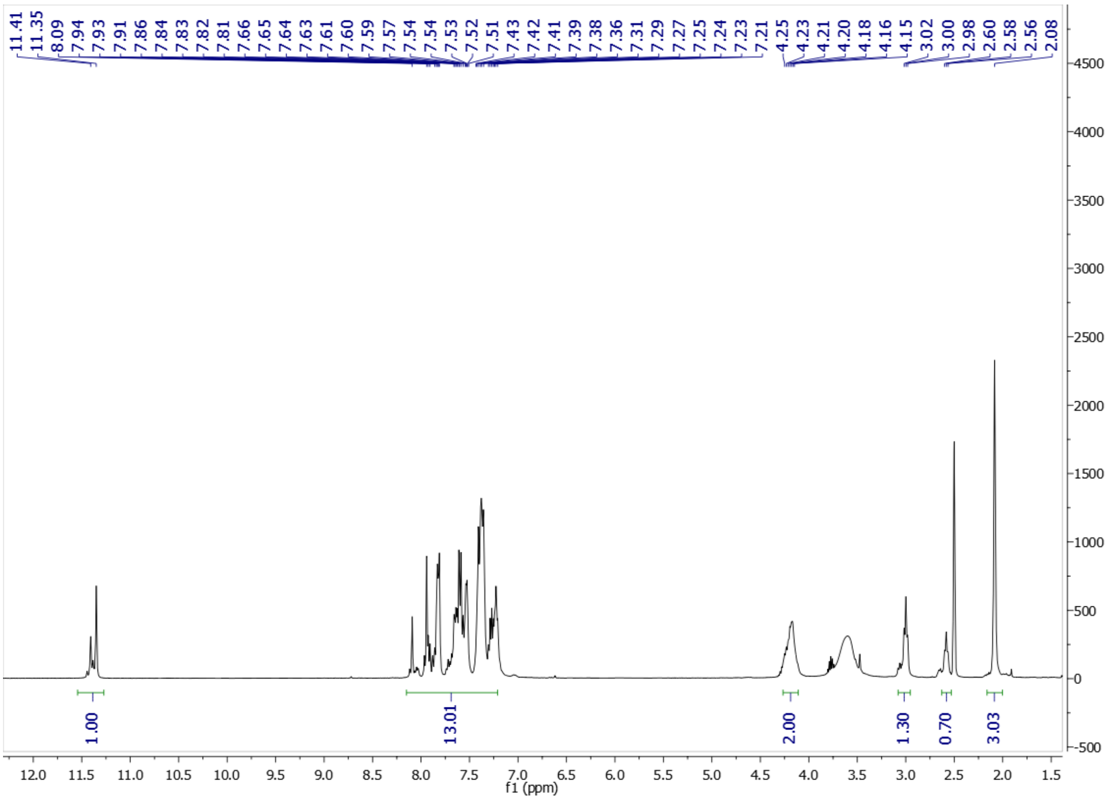

Figure S94. ^{1}H NMR of compound **30** at 400 MHz (DMSO-*d*6)

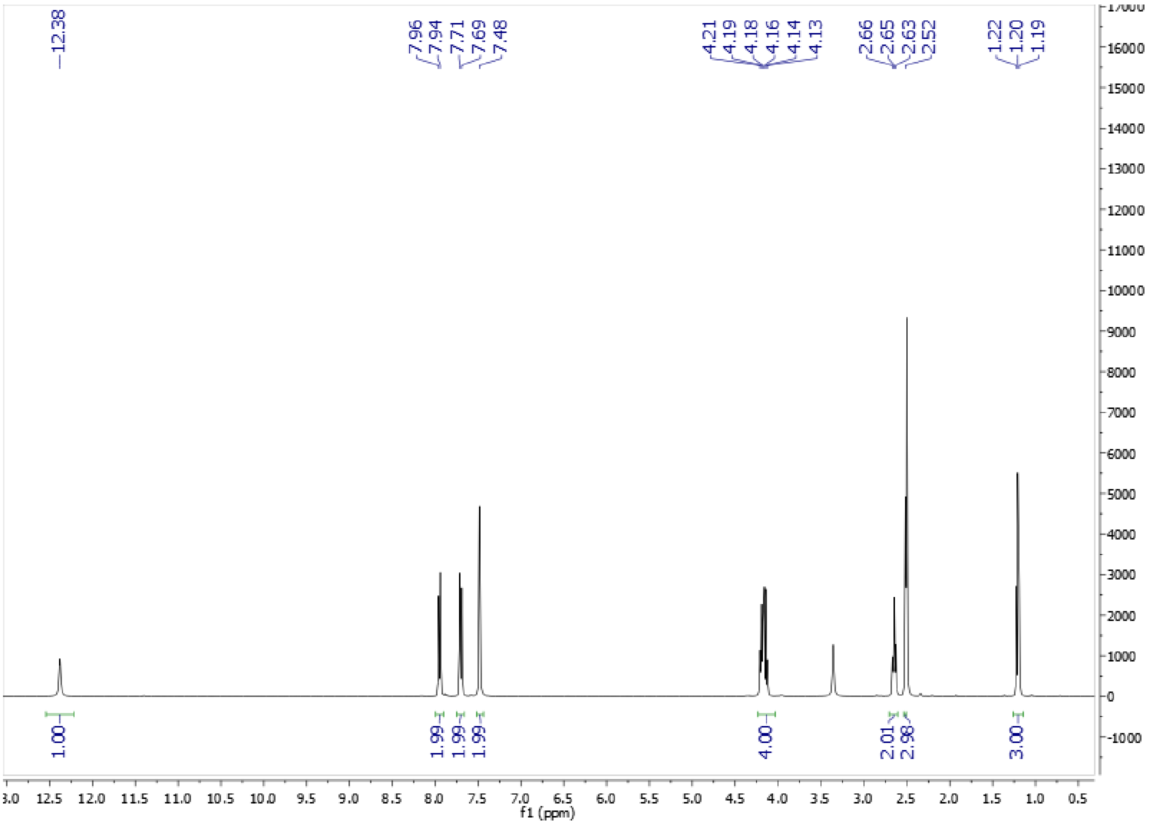

Figure S95. ^{1}H NMR of compound **31** at 400 MHz (DMSO-d_6)

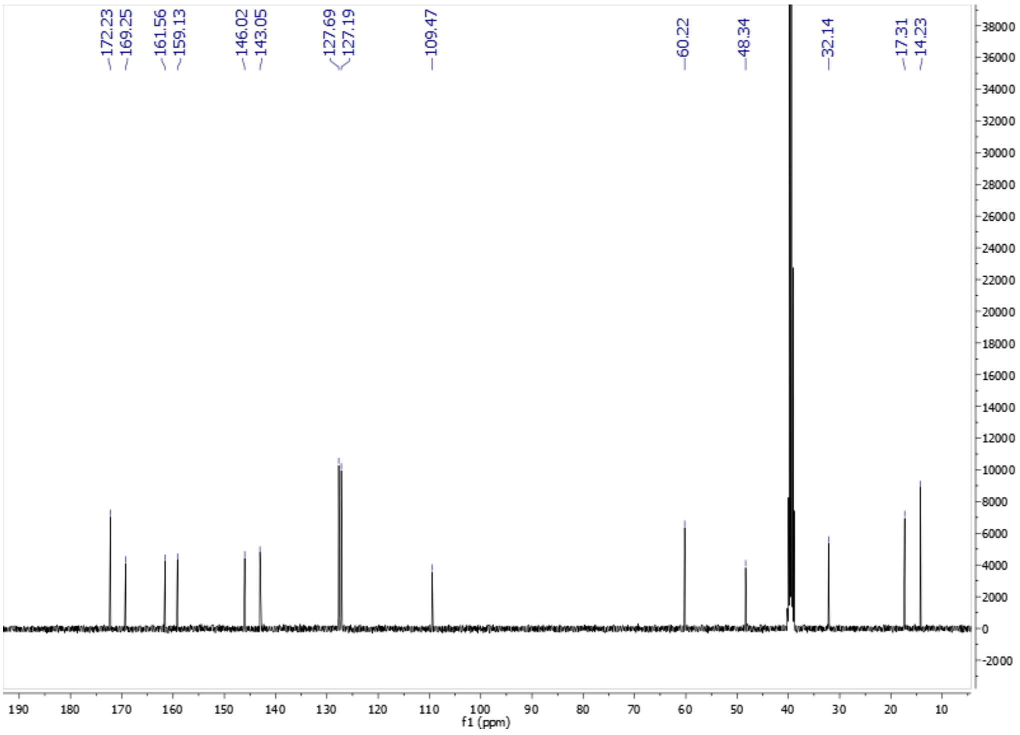

Figure S96. ^{13}C NMR spectrum of compound **31** at 101 MHz (DMSO-d_6)

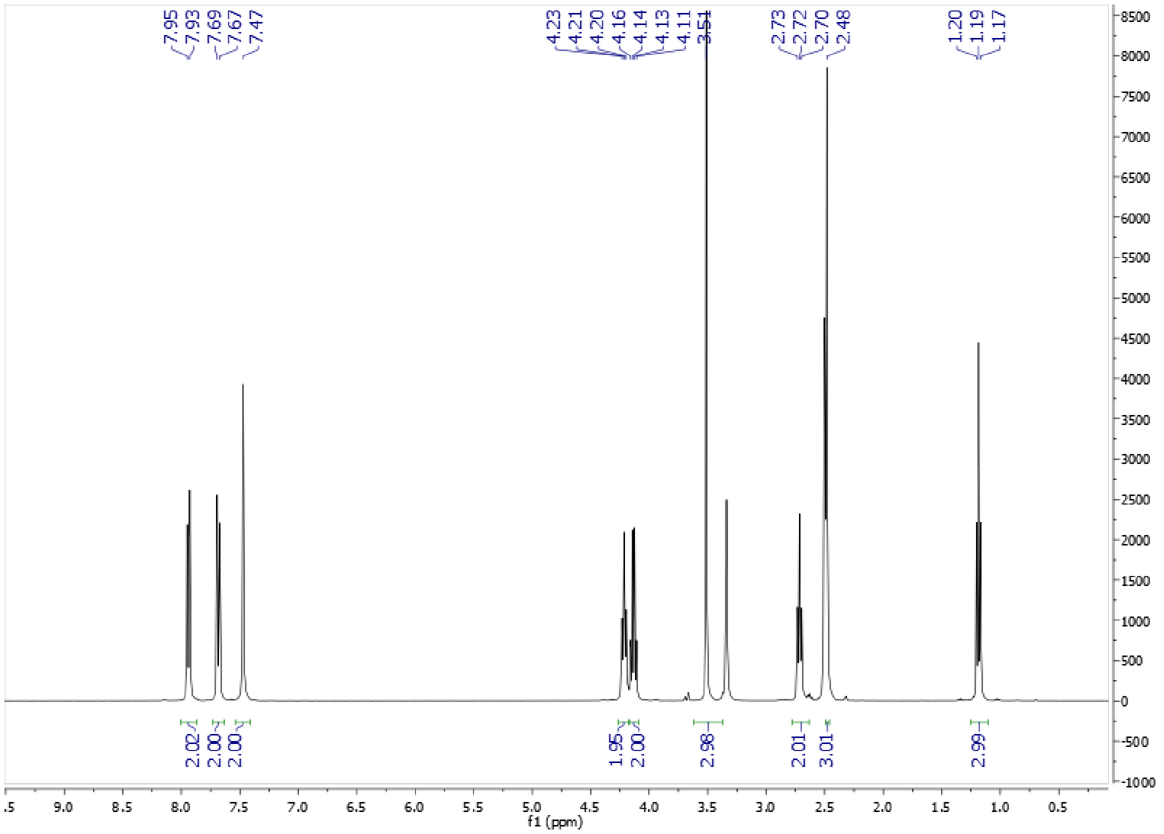

Figure S97. ^{1}H NMR of compound **32** at 400 MHz (DMSO-*d*6)

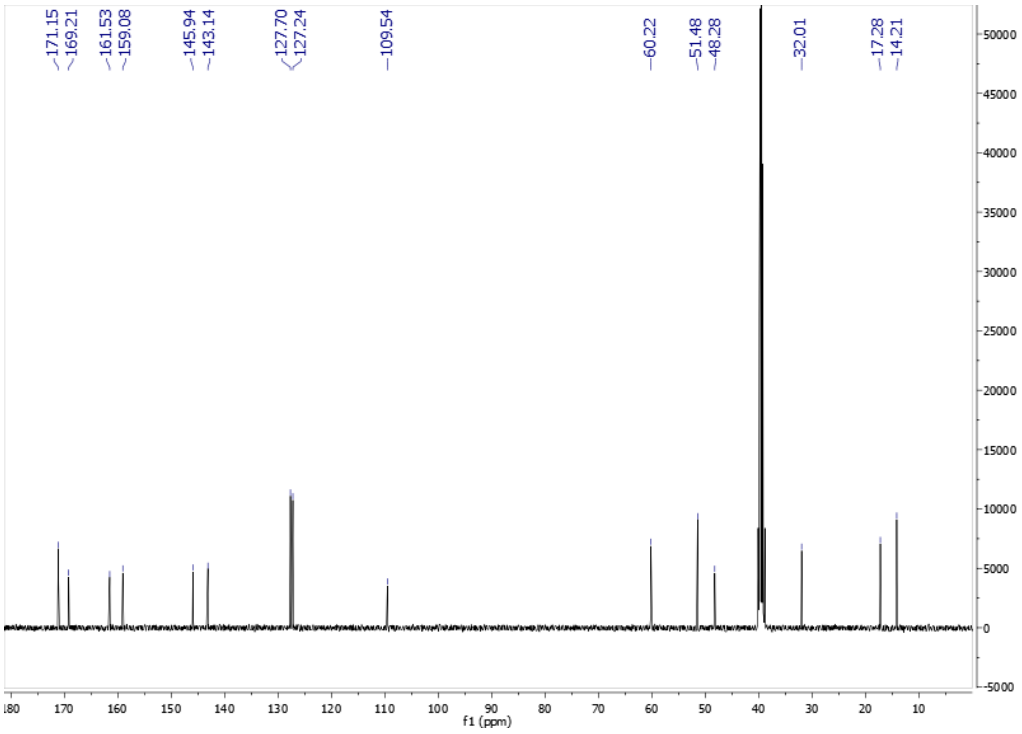

Figure S98. ^{13}C NMR spectrum of compound **32** at 101 MHz (DMSO-*d*6)

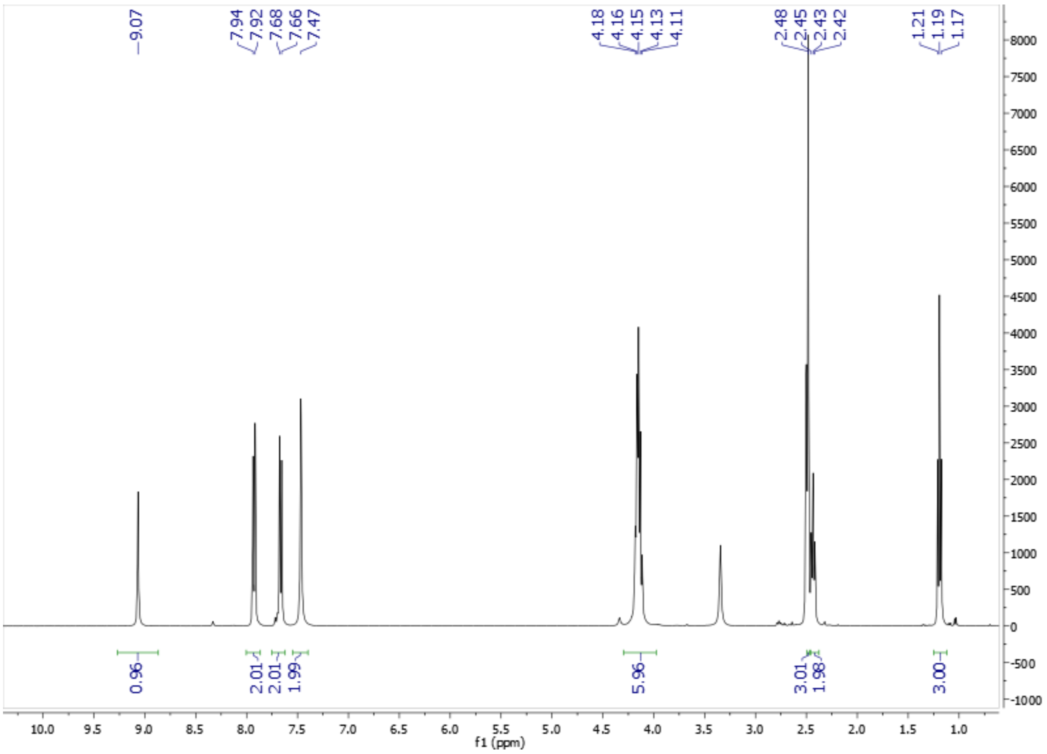

Figure S99. ^{1}H NMR of compound **33** at 400 MHz (DMSO-d_6)

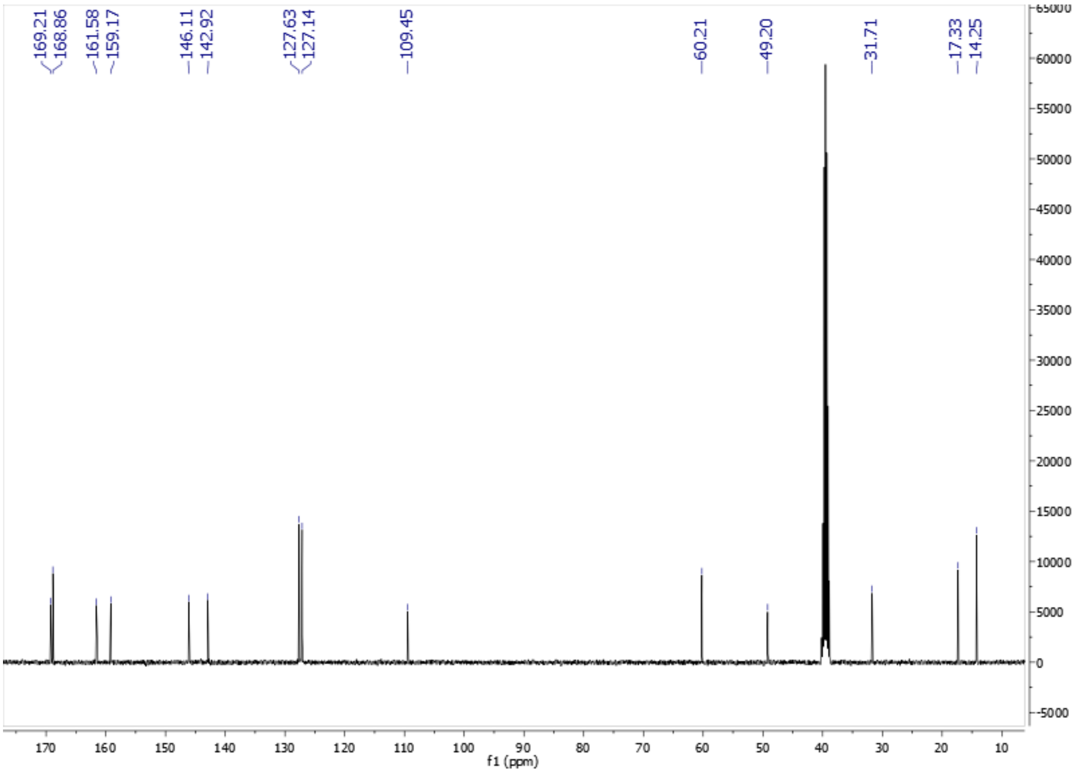

Figure S100. ^{13}C NMR spectrum of compound **33** at 101 MHz (DMSO-d_6)

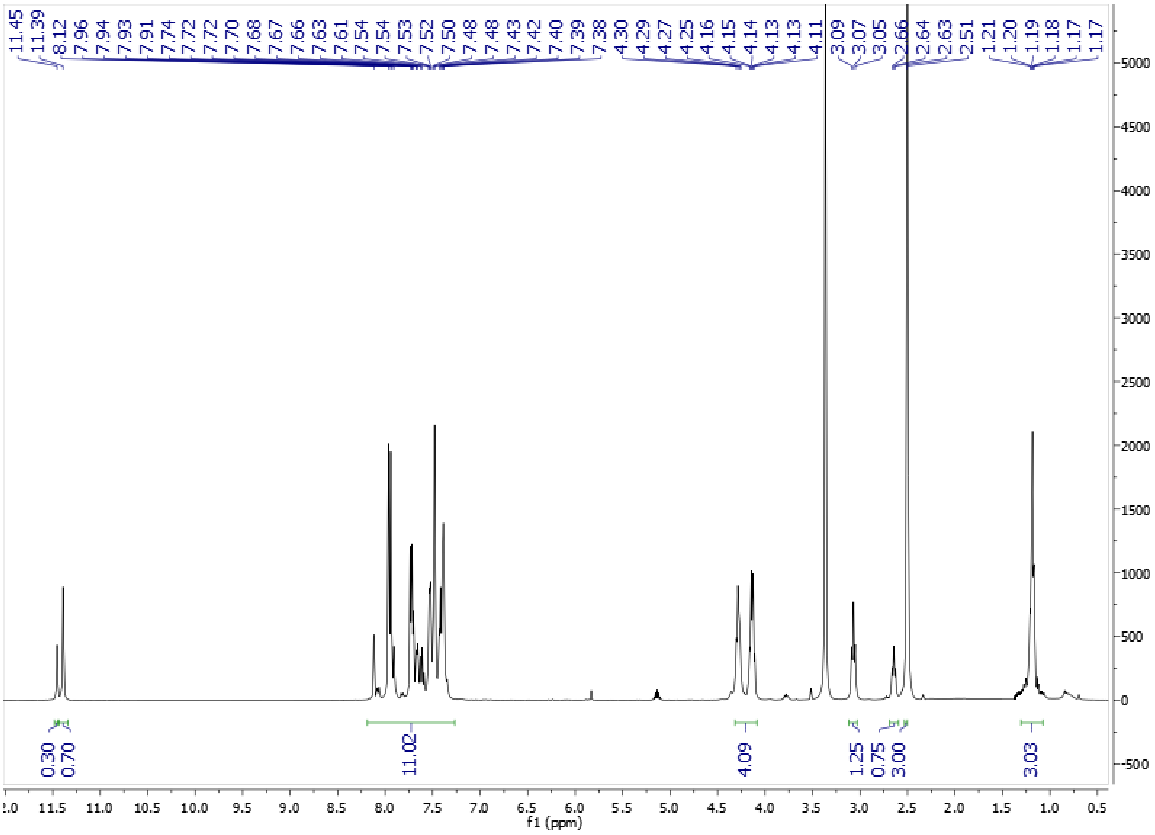

Figure S101. ^{1}H NMR of compound **34** at 400 MHz (DMSO-d_6)

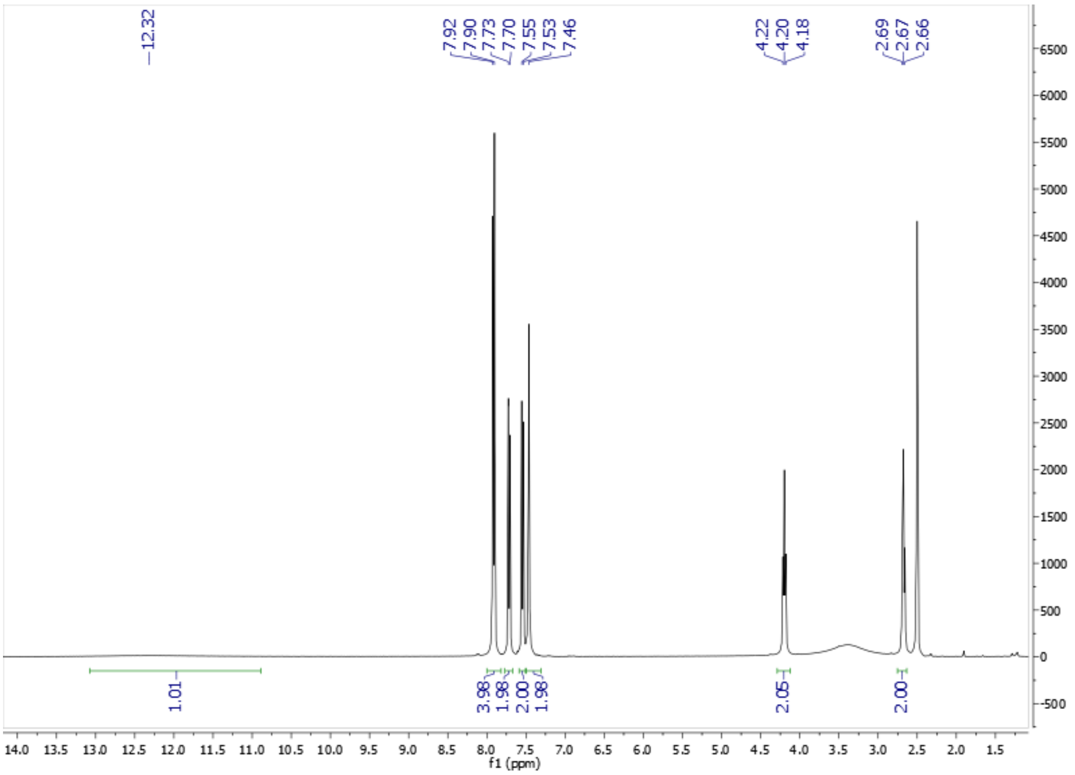

Figure S102. ^{1}H NMR of compound **36** at 400 MHz (DMSO-d_6)

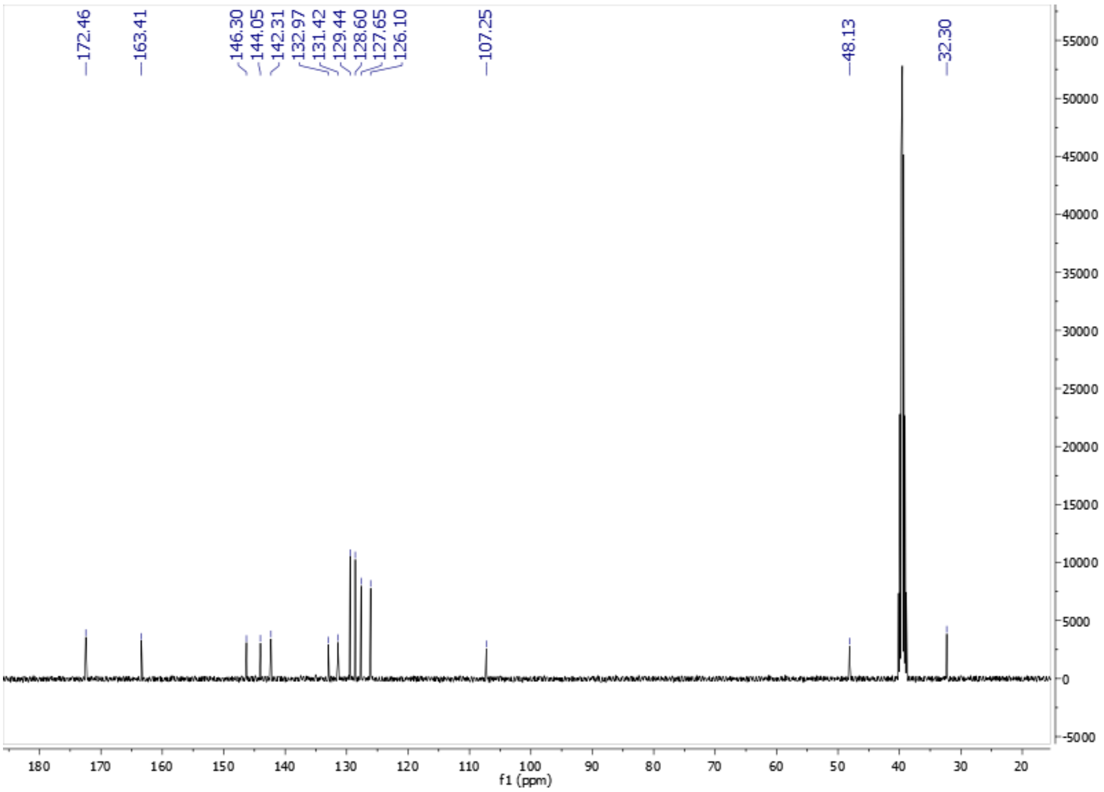

Figure S103. ^{13}C NMR spectrum of compound **36** at 101 MHz (DMSO-d_6)

Isothermal titration calorimetry data

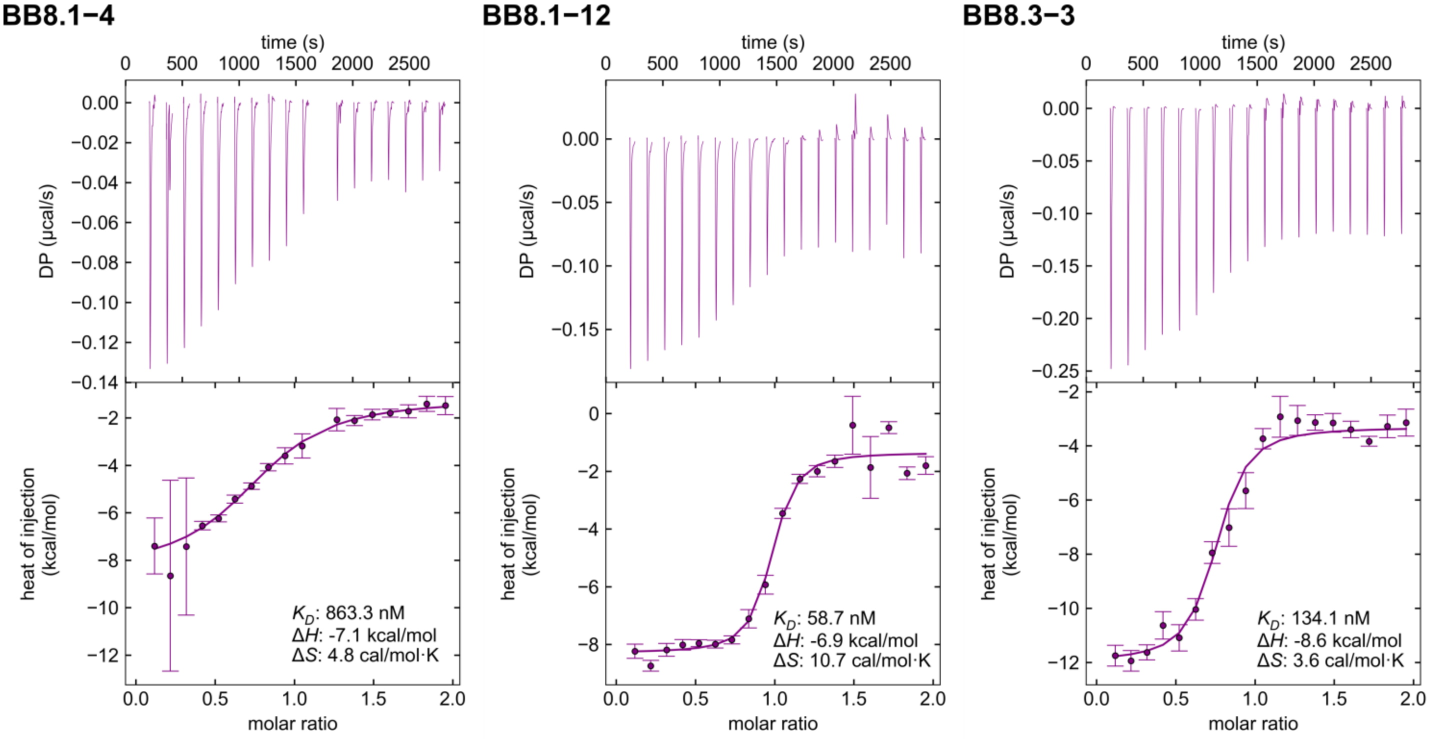

Figure S104. Isothermal titration calorimetry data of CAII binding with compounds **31** (*BB8.1-4*), **28** (*BB8.1-12*) and **36** (*BB8.3-3*) at 37 ˚C and pH 7.0 in 50 mM sodium phosphate buffer containing 100 mM NaCl and 2% (v/v) of DMSO. Experiments were performed on MicroCal PEAQ-ITC calorimeter (Malvern) and analyzed using NITPIC[2,3], SEDPHAT[4].

pK_a values of sulfonamide amino group

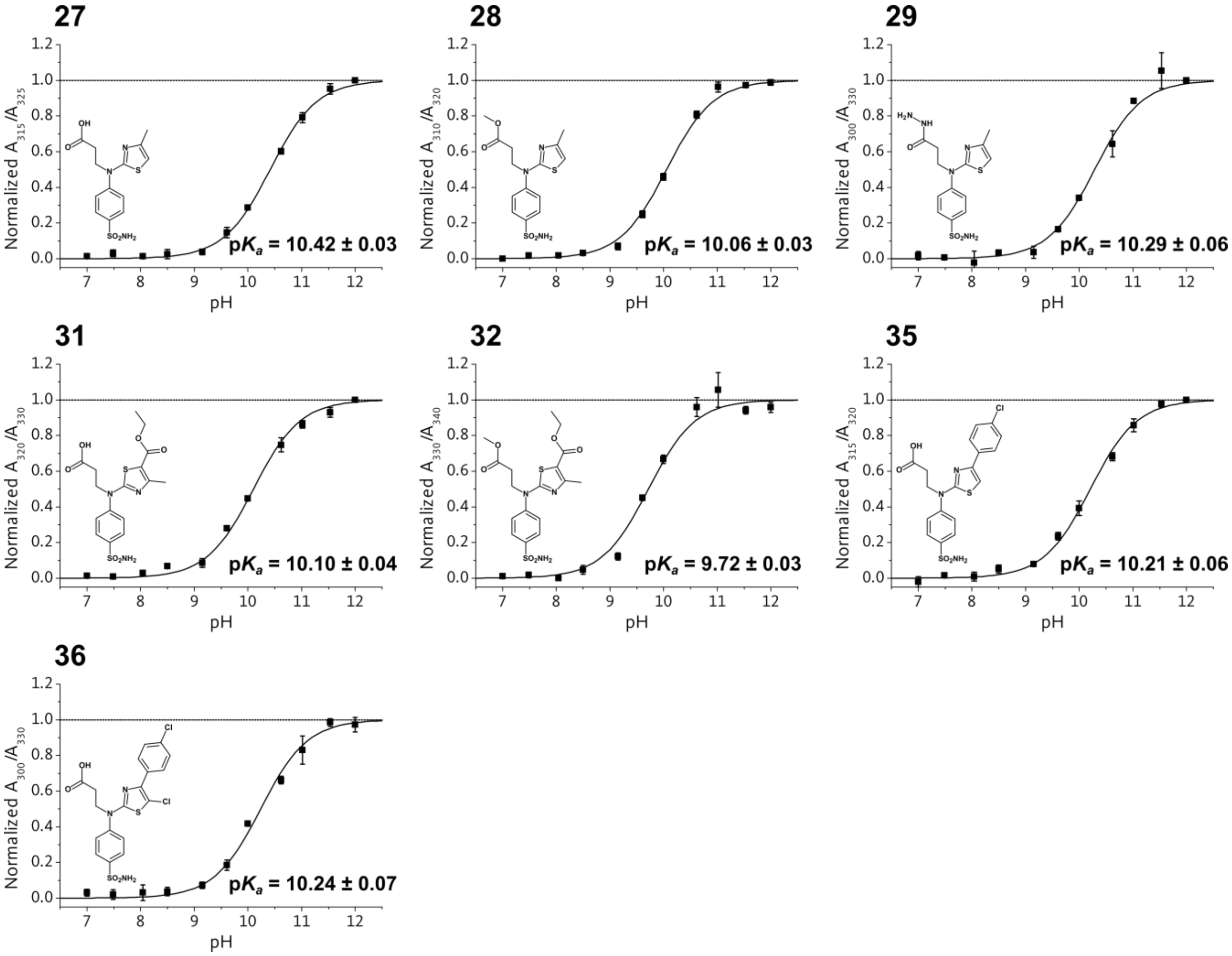

Figure S105. pK_a of sulfonamide amino group measurement. Average normalized ratio of absorbances (black squares) with corresponding error margins plotted as function of pH and fitted to Henderson–Hasselbalch curve (black line) using least square method for compounds **27**, **28**, **29**, **31**, **32**, **35** and **36**. pK_a values and compound structures are shown near corresponding graphs. pK_a values were measured twice for compounds **29** and **36**, three times for compounds **28**, **31**, **32** and **35** and four times for compound **27**. Only average pK_a value plus-minus standard deviation is given in the figure. Averages of pK_a values are used in calculations of intrinsic thermodynamic parameters for compounds whose pK_a values were not measured.

References

1. Laskowski, R.A.; Swindells, M.B. LigPlot+: multiple ligand-protein interaction diagrams for drug discovery. *J. Chem. Inf. Model*. **2011**, 51(10), 2778–2786. https://doi.org/10.1021/ci200227u

2. Keller, S.; Vargas, C.; Zhao, H.; Piszczek, G.; Brautigam, C.A.; Schuck, P. High-Precision Isothermal Titration Calorimetry with Automated Peak Shape Analysis. *Anal. Chem.* **2012**, *84*, 5066–5073. https://doi.org/10.1021/ac3007522.

3. Brautigam, C.A.; Zhao, H.; Vargas, C.; Keller, S.; Schuck, P. Integration and global analysis of isothermal titration calorimetry data for studying macromolecular interactions. *Nat. Protocols.* **2016**, *11*, 882–894. https://doi.org/10.1038/nprot.2016.044.

4. Zhao, H.; Piszczek, G.; Schuck, P. SEDPHAT—A platform for global ITC analysis and global multi-method analysis of molecular interactions. *Methods* **2015**, *76*, 137–148. https://doi.org/10.1016/j.ymeth.2014.11.012.
